# Structure-Based Design, Docking and Binding Free Energy Calculations of A366 Derivatives as Spindlin1 Inhibitors

**DOI:** 10.3390/ijms22115910

**Published:** 2021-05-31

**Authors:** Chiara Luise, Dina Robaa, Pierre Regenass, David Maurer, Dmytro Ostrovskyi, Ludwig Seifert, Johannes Bacher, Teresa Burgahn, Tobias Wagner, Johannes Seitz, Holger Greschik, Kwang-Su Park, Yan Xiong, Jian Jin, Roland Schüle, Bernhard Breit, Manfred Jung, Wolfgang Sippl

**Affiliations:** 1 Institute of Pharmacy, Martin Luther University of Halle-Wittenberg, Kurt-Mothes-Str.3, 06120 Halle/Saale, Germany; luisechi@hotmail.it (C.L.); dina.robaa@pharmazie.uni-halle.de (D.R.); 2 Institute for Organic Chemistry, University of Freiburg, Albertstr. 21, 79104 Freiburg, Germany; pm.regenass@gmail.com (P.R.); DM.Maurer@web.de (D.M.); dmytroostrovskyi@googlemail.com (D.O.); bernhard.breit@chemie.uni-freiburg.de (B.B.); 3 Institute of Pharmaceutical Sciences, University of Freiburg, Albertstr. 25, 79104 Freiburg, Germany; ludwig.seifert@pharmazie.uni-freiburg.de (L.S.); johannes.bacher@pharmazie.uni-freiburg.de (J.B.); teresa.burgahn@web.de (T.B.); dr.wagner.tobias@gmail.com (T.W.); johannes.seifert@pharmazie.uni-freiburg.de (J.S.); manfred.jung@pharmazie.uni-freiburg.de (M.J.); 4 Department of Urology and Center for Clinical Research, University Freiburg Medical Center, Breisacherstr. 66, 79106 Freiburg, Germany; holger.greschik@uniklinik-freiburg.de (H.G.); roland.schuele@uniklinik-freiburg.de (R.S.); 5 Mount Sinai Center for Therapeutics Discovery, Departments of Pharmacological Sciences and Oncological Sciences, Tisch Cancer Institute, Icahn School of Medicine at Mount Sinai, New York, NY 10029, USA; kwang-su.park@mssm.edu (K.-S.P.); yan.xiong@mssm.edu (Y.X.); jian.jin@mssm.edu (J.J.); 6 CIBSS—Centre for Integrative Biological Signalling Studies, University of Freiburg, 79104 Freiburg, Germany

**Keywords:** Spindlin1, structure–activity relationship (SAR), docking, molecular dynamics (MD) simulations, MM-GBSA

## Abstract

The chromatin reader protein Spindlin1 plays an important role in epigenetic regulation, through which it has been linked to several types of malignant tumors. In the current work, we report on the development of novel analogs of the previously published lead inhibitor **A366**. In an effort to improve the activity and explore the structure–activity relationship (SAR), a series of 21 derivatives was synthesized, tested in vitro, and investigated by means of molecular modeling tools. Docking studies and molecular dynamics (MD) simulations were performed to analyze and rationalize the structural differences responsible for the Spindlin1 activity. The analysis of MD simulations shed light on the important interactions. Our study highlighted the main structural features that are required for Spindlin1 inhibitory activity, which include a positively charged pyrrolidine moiety embedded into the aromatic cage connected via a propyloxy linker to the 2-aminoindole core. Of the latter, the amidine group anchor the compounds into the pocket through salt bridge interactions with Asp184. Different protocols were tested to identify a fast in silico method that could help to discriminate between active and inactive compounds within the **A366** series. Rescoring the docking poses with MM-GBSA calculations was successful in this regard. Because **A366** is known to be a G9a inhibitor, the most active developed Spindlin1 inhibitors were also tested over G9a and GLP to verify the selectivity profile of the **A366** analogs. This resulted in the discovery of diverse selective compounds, among which **1s** and **1t** showed Spindlin1 activity in the nanomolar range and selectivity over G9a and GLP. Finally, future design hypotheses were suggested based on our findings.

## 1. Introduction

Spindlin1 is a chromatin reader protein that plays an important role in epigenetic regulation through the recognition and interpretation of histone modifications. It consists of three Tudor domains, with the second domain binding to the H3K4me3 (H3 trimethylated at lysine 4) mark [1,2,3]. More recently, its interaction with H4K20me3 (H4 trimethylated at lysine 20) was also described [4,5]. Since the binding of the latter histone mark displayed a weaker affinity than that of H3K4me3, it has been hypothesized that the H4K20me3 mark acts as a secondary binding partner for Spindlin1 [5]. Interestingly, the additional binding of the first domain with asymmetrically dimethylated arginine residues (Rme2a) has been shown to have different effects on the histone peptide affinity. Indeed, the H3K4me3R8me2a methylation pattern increases the affinity of H3K4me3, whereas H4K20me3R23me2a shows a lower binding affinity than H4K20me3 [3,5]. Very recently, another bivalent methylation pattern has been reported, namely H3K4me3K9me3/2, which has been described to enhance the histone binding affinity of H3K4me3 [6].

H3K4 is one of the most studied histone modifications, and it is well known to be tightly associated with the promoters of active genes that lead to transcriptional activation [7]. In contrast, H4K20 has been reported to have different functions based on the methylation states; H4K20me1 is associated with transcriptional activation, while H4K20me3 is linked with repression of transcription when present at promoters [8]. However, further studies are needed to investigate the biological relevance of the H4K20me3 mark and its functions correlated with Spindlin1 interactions.

Spindlin1 has been linked to several types of malignant tumors including ovarian cancer, breast cancer and triple negative breast cancer, non-small-cell lung cancers, liposarcoma, and very recently, liver cancer [9,10,11,12,13,14]. Moreover, it was found to be associated with drug-resistant breast cancer, as a study established that Spindlin1, which is negatively regulated by the miR-148/152 family, enhances Adriamycin resistance [15]. The literature also reports that *Spindlin1* may play a role in tumorigenesis, and it was additionally suggested that *Spindlin1* is a proto-oncogene [16,17,18,19,20]. Some of the above-mentioned Spindlin1 functions have been related to diverse signaling pathways, such as Wnt (β-catenin and TCF-4) [3,21], RET [13], PI3K/Akt [10], and uL18-MDM2-p53 [19] pathways and SREBP1c-triggered FASN signaling [14]. In addition, Spindlin1 was found to control skeletal muscle development in mice and to play an important role in the first meiotic division of mammalian oocytes, thus providing potential links of Spindlin1 to human skeletal muscle diseases and human infertility, respectively [22,23]. Therefore, Spindlin1 inhibitors might be useful chemical tools to further investigate the physiological and pathological roles of this relatively new target.

To date, 15 crystal structures of Spindlin1 have been deposited in the RCSB Protein Data Bank (PDB) [24], both in apo form [25] and in complex with histone peptides [2,3,5], bivalent inhibitors, and small molecule inhibitors [26,27,28]. The crystal structure analysis and mutagenesis studies of some residues present in the first and second Tudor domains clearly indicated, as mentioned above, that the second domain is responsible for the recognition of the trimethylated lysine [2,3,5,13]. Specifically, four residues (Phe141, Trp151, Tyr170, and Tyr177) form the so-called aromatic cage in which the trimethylated lysine is embedded and stabilized by cation–pi interactions and hydrophobic van der Waals contacts.

**Figure 1:**
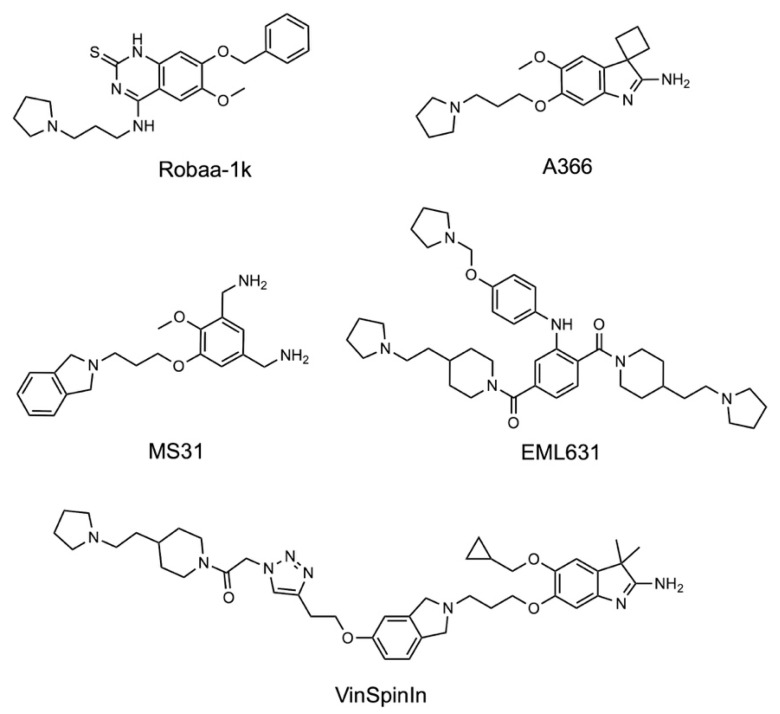
Structures of reported Spindlin1 inhibitors.

In recent years, there has been growing interest in Spindlin1, which has led to the identification of several inhibitors blocking the Spindlin–H3K4me3 interaction. Through in silico studies coupled with in vitro testing, we identified novel Spindlin1 inhibitors active in the low µM range; as an example, compound Robaa-1k is illustrated in Figure 1 [29]. Using a screening platform, diverse compounds were identified, leading to the discovery of **A366**, a previously described G9a inhibitor (IC_50_: 3.3 nM, [30]), as a nanomolar Spindlin1 inhibitor (IC_50_: 182.6 nM, [31]) (Figure 1). Recently, applying the same approach to the screening of an epigenetic compound library, another hit was discovered, which was then simplified to a fragment-like inhibitor (MS31, Figure 1) [28]. Two different studies have identified bivalent inhibitors of Spindlin1 that simultaneously interact with its Tudor domain I and II (EML631 and VinSpinIn, Figure 1) [26,27].

Herein we report on the development of novel analogs of the **A366** inhibitor. In an attempt to improve the activity of **A366** and explore the structure–activity relationship (SAR), a congeneric series containing 21 **A366** derivatives was synthesized and tested in vitro for their inhibitory activity against Spindlin1. This was accompanied by various computational approaches in order to investigate the possible binding mode of the synthesized derivatives, to explore the impact of the structural variations on the Spindlin1 inhibitory activity, and to identify a fast in silico method that could help to discriminate between active and inactive compounds within the **A366** series. Additionally, to verify the selectivity of the new analogs, the most active compounds were also tested in vitro against G9a and GLP (G9a like protein).

## 2. Results and Discussion

In order to explore the SAR of **A366** and to develop modified analogs, a congeneric series was synthesized and tested. This led to the generation of a dataset consisting of 21 compounds, 17 of which showed IC_50_ values in the range of 0.15–11.8 μM, two of which were found to be weakly active (Table 1, **1a** and **1b**), and three of which displayed a considerably decreased activity and were, thus, considered inactive (Table 1, **1c**, **1d**, **1e**). Apart from **A366** [31], **1f**, and **1g** [27], the other compounds are reported here for the first time as novel Spindlin1 inhibitors. The attained dataset was subsequently used for computational studies aimed to investigate the structural differences responsible for the activity of the compounds. Docking studies, molecular dynamics (MD) simulations, and molecular mechanics/generalized born surface area (MM-GBSA) rescoring calculations were performed using the Spindlin1 second domain as a binding site (PDB ID: 6I8Y).

### 2.1. Analysis of Spindlin1-A366 Complex

**Figure 2:**
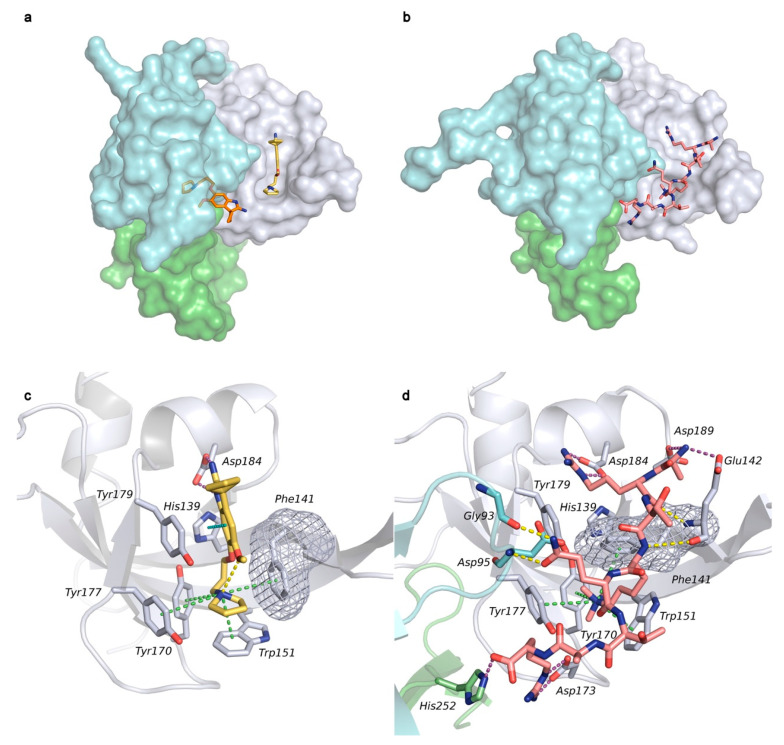
Crystal structures of Spindlin1 in complex with (**a**,**c**) **A366** (PDB ID: 6I8Y) and (**b**,**d**) H3K4me3 (PDB ID: 4H75). (**a**,**b**) show the protein surfaces of the three Tudor domains, which are colored in cyan (domain I), white (domain II), and green (domain III). **A366** bound to the first Spindlin1 domain is represented as orange sticks. (**c**,**d**) focus on domain II and the binding interactions of **A366** (yellow sticks) and H3K4me3 histone peptide (pink sticks). Only the side chains of the surrounding residues are shown for clarity, and they are displayed as sticks; for residues Gly93, Asp95, and Glu142, the main chains are shown. Phe141 is also depicted by the surface mesh. Hydrogen bonds are represented as dashed yellow lines, cation–pi interactions as dashed green lines, pi–pi stacking interactions as dashed cyan lines, and salt bridges as dashed magenta lines.

The binding mode of **A366** in the Spindlin1 second domain was first analyzed (Figure 2a,c) (PDB ID: 6I8Y [27]). This crystal structure shows a different shape of the binding pocket compared to the histones-bound crystal structures (e.g., PDB ID: 4H75 [2]). Indeed, **A366** induces a flip of Phe141 in the aromatic cage; this conformational change of the phenylalanine side chain consequently opens the pocket and changes its shape and size. The protonated pyrrolidine moiety is embedded in the aromatic cage and undergoes cation–pi interactions with the surrounding amino acids. Notably, also the side chain of Trp151 is slightly adapted to better interact with **A366**. The amidine group is involved in salt bridge interactions with Asp184. The ligand binding mode is further stabilized by the formation of an intramolecular hydrogen bond between the methoxy group and the positively charged pyrrolidine-NH group. For comprehension and comparison, the crystal structure of Spindlin1 bound to the H3K4me3 histone peptide with a focus on the second domain is shown in Figure 2b,d (PDB ID: 4H75).

### 2.2. Docking Studies

Initially, our attention was focused on understanding the role of the spacer length (R3 group), since biological assays revealed that changing the linker from a propyl chain to ethyl/butyl chains results in a drop in activity (Table 1, compounds **1c**, **1d**, **1e**). The compounds were hence docked into the crystal structure of Spindlin1 (PDB ID: 6I8Y) using Glide in the standard precision (SP) mode [32]. It is noteworthy that the used docking setup could successfully reproduce the experimentally determined binding mode and the interactions of **A366** with an RMSD of 0.54 Å (Supplementary Materials, Figure S1).

**Figure 3:**
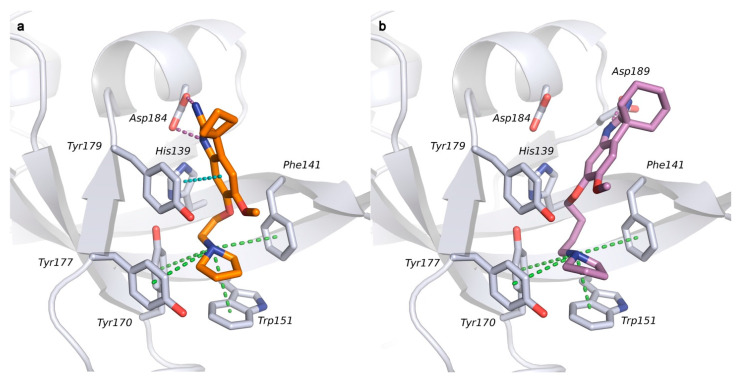
Docking poses of **1e** (**a**) and **1c** (**b**) in the Spindlin1 second domain (PDB ID: 6I8Y). Docking poses and side chains of the surrounding residues are shown as sticks. Binding interactions are represented with dashed lines colored in green (cation–pi), cyan (pi–pi stacking), and magenta (salt bridge).

The obtained docking poses and docking scores (DS) of the inactive compounds were analyzed and compared to **A366**. Interestingly, the docking scores showed no substantial differences between the active and inactive compounds (DS range of inactive ligands: −11.16/−11.28 kcal/mol; DS of **A366**: −11.37 kcal/mol—Table S1). Docking of the inactive derivative **1e** (Figure 3a), which bears a shorter ethoxy spacer as compared with **A366**, resulted in the same binding mode and showed the same interactions as observed for **A366** (Figure 2c). On the other hand, the inactive derivative bearing a butoxy spacer, compound **1c**, displayed a slightly different binding mode in which the amidine moiety is tilted towards Asp189 and establishes a salt bridge with it (Figure 3b). Nevertheless, the pyrrolidine ring of **1c** is still properly located in the aromatic cage where it undergoes cation–pi interactions.

The main difference detected in the binding modes of all three inactive compounds (**1c**, **1d**, and **1e**) lies in the absence of the intramolecular hydrogen bond observed in the binding mode of **A366**. There is reason to believe that the formation of an intramolecular hydrogen bond plays an important role in stabilizing the active conformation.

### 2.3. Molecular Dynamic Simulations

Given the poor understanding of the compounds’ inactivity by docking scores and predicted binding mode, the next step was the investigation of these ligands by means of MD simulations. The obtained docking poses of **A366** and two inactive compounds (**1e** and **1c**) were subjected to 200 ns MD simulations using Amber18 package [33] to gain insight into the stability of the predicted binding modes and protein–ligand interactions.

**Figure 4:**
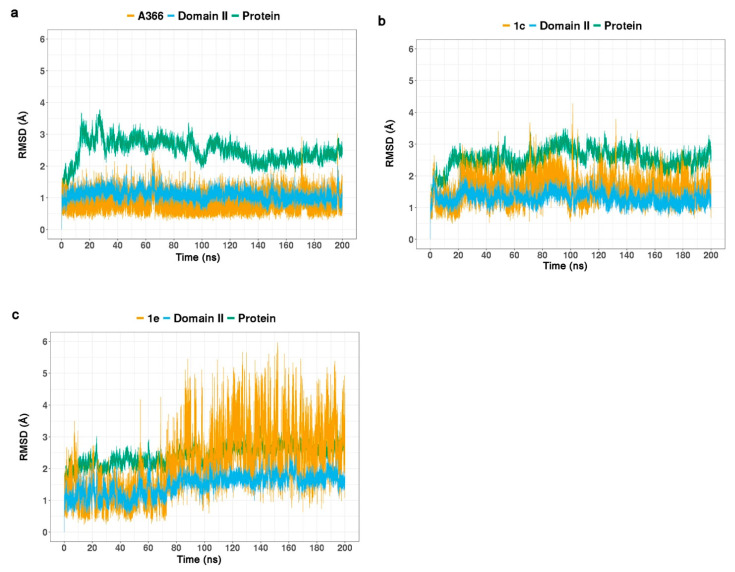
Analysis of 200 ns MD simulations of reference ligand **A366** and inactive compounds **1c** and **1e**. Root mean square deviation (RMSD) plots of the systems with (**a**) **A366**, (**b**) **1c**, and (**c**) **1e**.

**Figure 5:**
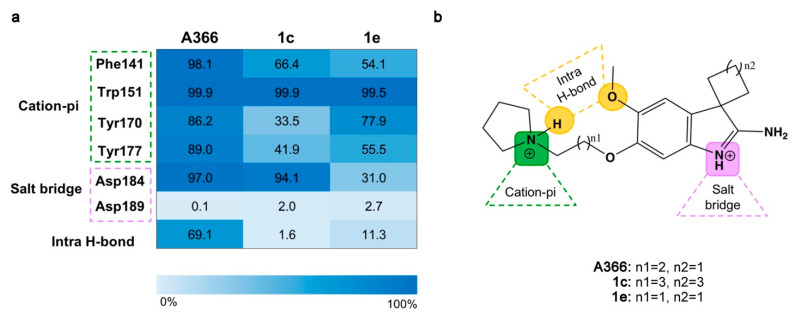
Summary of the binding interactions identified during 200 ns MD simulations of the reference ligand **A366** and the inactive compounds **1c** and **1e**. The binding poses were obtained from docking into Spindlin1 second domain binding site (PDB ID: 6I8Y). (**a**) Heat map showing the occupancy rates of the observed interactions. (**b**) Common structure of the compounds highlighting the atoms involved in the interactions.

The analysis of the MD simulation performed for **A366** indicated a stable binding mode, where the interactions observed in the crystal structure (Figure 2c) are preserved throughout the simulation. It is noteworthy that the intramolecular hydrogen bond between the methoxy group and the pyrrolidine-N^+^H was also maintained throughout the MD simulation. Meanwhile, for both inactive derivatives, **1c** and **1e**, this intramolecular hydrogen bond was barely observed during the MD simulation. Figure 4a shows the root-mean-square deviation (RMSD) plot, while the occupancy rates of the most important interactions are reported in the heat map (Figure 5).

Compound **1c**, despite its inactivity, was found to be relatively stable throughout the MD simulation in terms of RMSD values, albeit less stable than **A366** (Figure 4b). Although the obtained docking pose of **1c** shows an interaction between the amidine moiety of the ligand and Asp189 (Figure 3b), this interaction is lost during the MD simulation (occupancy 2%). Indeed, directly at the beginning of the MD simulation, the aminoindole moiety moves so that its amidine group establishes a salt-bridge with the side chain of Asp184, which is maintained for ca. 94% of the simulation time. Apart from the aminoindole group, small fluctuations of the ligand are observed, which mainly affect the pyrrolidine moiety and, consequently, the formation of cation–pi interactions. This was highlighted by the decreased occupancy of these interactions during the simulation (Figure 5). In fact, with the exception of the cation–pi interactions established with Trp151, the occupancies of the other aromatic interactions are dramatically decreased, pointing out the important role of these interactions for the activity.

On the other hand, the instability of the binding mode obtained for **1e** could give a plausible explanation for its inactivity (Figure 4c). Indeed, diverse binding conformations were detected during the MD simulation (Supplementary Materials, Figure S2), all of which seem to be unstable, as demonstrated also by the low occupancy rates of the observed interactions, as detailed in Figure 5. The salt bridge between the amidine group and Asp184 (occupancy rate 31%) is completely lost after 70 ns simulation time (Supplementary Materials, Figure S3). Moreover, the analysis of the cation–pi interactions underlined that even though the positively charged pyrrolidine moiety is located in the aromatic cage, its position is not optimal for establishing aromatic interactions with all amino acids that form the cage. This can be seen in Figure 5, specifically, in the low occupancy rates obtained for the interactions with Phe141 and Tyr177 (54.1% and 55.5%, respectively).

In conclusion, based on the MD simulations analysis of these three compounds, we could confirm the assumption that the spacer length is important for stabilizing the binding pose and optimizing the binding interactions. The finding shows that the linker chain can affect not only the presence of intramolecular hydrogen bond, but also the optimal location of the pyrrolidine ring in the pocket and generally the binding conformation, which in turn influences the formation of cation–pi interactions with the aromatic cage as well as the interactions of the amidine moiety with Asp184.

### 2.4. Re-Scoring Using Prime MM-GBSA

Although the analysis of the MD simulations was able to rationalize the difference in the compounds’ activity, this approach is time-consuming for a bigger dataset. Thus, the synthesis of the new derivatives was coupled with docking and MM-GBSA rescoring to find a fast approach that could help in discriminating active from inactive compounds of the same congeneric series. The structural differences between the active and inactive compounds lie in the linker chain, where the actives bear a propyl chain, whereas the inactive compounds bear either an ethyl or a butyl chain (Table 1, compounds **1c**, **1d**, and **1e**). Only ligands with determined IC_50_ values were used as actives.

**Figure 6:**
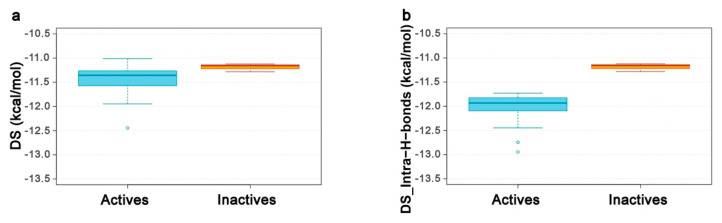
Box-plots showing docking scores distribution obtained for active and inactive compounds using glide standard precision (**a**) without intramolecular hydrogen bonds contribution and (**b**) rewarding intramolecular hydrogen bonds.

First, since from the above-described docking studies and MD simulations analysis the key role of the intramolecular hydrogen bond was evident, its contribution was incorporated into the docking scores. This was done by allowing the option “reward intramolecular hydrogen bonds” in Glide [32]; in this way, the compounds that exhibit these intramolecular interactions pay a smaller entropic penalty upon binding, which affects the final docking scores (DS_Intra-H-bonds). For the analysis of the results, box-plots were generated showing the distribution of docking scores without the “reward intramolecular hydrogen bonds” option (DS) and DS_Intra-H-bonds attained for our active and inactive ligands. As illustrated in Figure 6a, the DS values retrieved for active and inactive compounds are distributed in the same range, which clearly demonstrates that this scoring function is not able to discriminate between active and inactive compounds. On the other hand, a small improvement is observed when DS_Intra-H-bonds values are evaluated (Figure 6b). However, the difference between active and inactive ligands is still too small to be considered reliable, as highlighted by the mean values (−12.0 kcal/mol for the actives and −11.2 kcal/mol for the inactive compounds).

**Figure 7:**
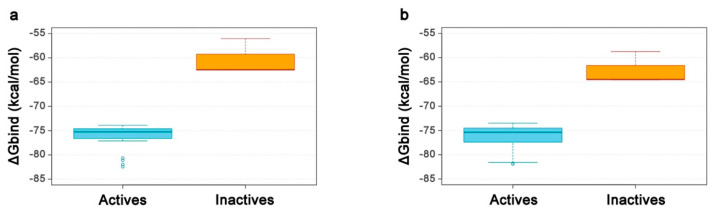
Box-plots showing ΔG_bind_ distribution obtained for active and inactive compounds using Prime MM-GBSA. (**a**) Default setting, only ligands are relaxed (**b**) also protein residues around 3 Å from the ligands are treated as flexible.

Owing to the low discriminatory power of the glide docking scores (DS and DS_Intra-H-bonds), the docking poses were post-processed using more accurate binding free energy (BFE) calculations with Prime MM-GBSA. Diverse settings were tested; here, we report the results obtained when only the ligands were relaxed, and when also the protein residues within 3 Å of the ligands were treated as flexible. The computed binding affinity values were visualized by box-plots, seen in Figure 7. Interestingly, the re-scoring methods were able to successfully discriminate between active and inactive compounds. The calculated BFE values retrieved for the active compounds were more favorable than those of the inactive compounds, and good discrimination ranges were found, as emphasized by the significant gaps between the mean values (MM-GBSA ΔG_bind_ mean values of −76.3 kcal/mol (actives) and −60.35 kcal/mol (inactives); MM-GBSA ΔG_bind_–3 Å mean values of −76.3 kcal/mol (actives) and −62.6 kcal/mol (inactives)).

The two re-scoring methods performed equally well from an efficiency perspective, but differently for the computation time. The CPU time required for the calculation of one inhibitor was 0.6 min when only the ligand was treated as flexible, but 1.7 min when the protein residues were included (MM-GBSA-3 Å). Given the promising findings, this developed protocol can be applied to guide further optimization steps in order to prioritize compounds for synthesis and biological characterization.

### 2.5. Structure–Activity Relationships

Once the role of the spacer was clear, we were interested in exploring the effect of other substitutions on the ligand’s inhibitory activity. The synthesized dataset of **A366** derivatives encompassed compounds bearing different spiro and dialkyl substituents at position-3 of the indole ring (R4), different substituents at the pyrrolidine ring, as well as varying groups at the R1 and R2 positions of the indole ring.

To investigate the influence of these modifications on the Spindlin1 inhibitory activity, the fluorescence polarization assay established earlier [31] was used to determine the potency of the compounds. Furthermore, the most active Spindlin1 inhibitors were tested in vitro against G9a and GLP to analyze their selectivity profile.

The biological assays revealed that the introduction of a substituent on R2 position led to a significant decrease in the Spindlin1 inhibitory activity as observed for **1a**, **1h**, **1i**, **1j**, and **1k** (IC_50_ values: 1.6–11.8 µM) when compared to the parent compound **A366** (IC_50_ 182.6 nM). Meanwhile, the replacement of the methoxy group at R1 with ethoxy (**1g**, **1l**) and isopropoxy (**1m**) groups showed different impacts on the activity of the compounds. The ethoxy derivatives showed only a two-fold decrease in the inhibitory activity, as observed when comparing **1g** and **1l** with **1f** and **1n**, respectively. On the other hand, the isopropoxy derivative **1m** displayed a significant decrease in activity with an IC_50_ value in the micromolar range.

Modifications of the spiro moiety (R4) had no major impact on the Spindlin1 inhibitory activity of the compounds; cyclobutyl, -pentyl, and -hexyl rings as well as the dimethyl analogues (**A366**, **1f**, **1n**, **1p**) were all well tolerated. Meanwhile, the 3-ethyl-3-methyl analogue **1r** (IC_50_ 408 ± 35 nM) displayed only a two-fold decrease in activity as compared to **A366**. However, no selectivity was detected for these compounds. Among the attempted R4-modifications, the major impact was observed with compound **1o** bearing the geminal diethyl group, which exhibited a significantly decreased activity for Spindlin1 (IC_50_: 1.21 ± 0.09 µM). Interestingly, this was coupled with an increase in selectivity for Spindlin1 over G9a and GLP (G9a IC_50_ >20 µM, GLP IC_50_ >20 µM). Noteworthy, the analogous derivative with dimethyl groups, **1p**, which showed an activity comparable to **A366** and was one of the most active Spindlin1 inhibitors of our series (Spindlin1 IC_50_: 157 ± 12 nM), showed no selectivity over G9a and GLP (G9a IC_50_: 759 nM, GLP IC_50_: 470 nM). The decrease in the Spindlin1 activity going from geminal dimethyl (**1p**) to 3-ethyl-3-methyl (**1r**) and ultimately to geminal diethyl (**1o**) is inversely related to the number of rotatable bonds and can, thus, be associated with the loss of conformational entropy of the compounds.

**Figure 8:**
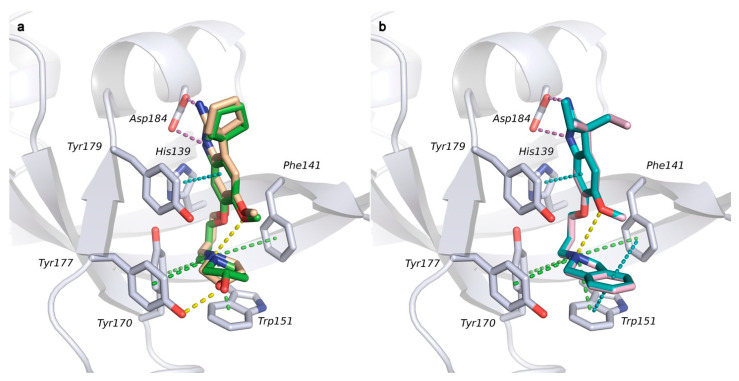
Predicted binding modes of some derivatives in the Spindlin1 second domain binding site (PDB ID: 6I8Y). (**a**) Compound **1q** (S, green; R, beige). (**b**) Compound **1s** (S, cyan; R, light pink). Docking poses and side chains of the surrounding residues are shown as sticks. Binding interactions are represented with dashed lines colored in yellow (hydrogen bond), green (cation–pi), cyan (pi–pi stacking), and magenta (salt bridge).

Given the important role of the pyrrolidine moiety in the binding pocket, it was not surprising that its modifications could impact the activity of the compounds. Notably, the replacement of the pyrrolidine ring in **1f** by a 3-hydroxypyrrolidine moiety (**1q**) only led to three-fold decrease in the Spindlin1 activity (IC_50_ values of 169 nM and 584 nM, respectively). The docking studies of **1q** predicted binding modes in which the hydroxy group is engaged in a hydrogen bond interaction with Tyr177 (Figure 8a). On the other hand, the replacement of the pyrrolidine ring by an isoindoline moiety led to compound **1s**, which showed strong inhibition of Spindlin1 (IC_50_: 360 ± 20 nM) combined with selectivity (G9a IC_50_ >20 µM, GLP IC_50_ >20 µM). Our docking studies in the Spindlin1 second domain binding site reveal that the aromatic ring of the isoindoline moiety can further stabilize the ligand through the formation of additional pi–pi stacking interactions with Phe141 and Trp151 in the aromatic cage (Figure 8b). To further explore the binding mode of compound **1s**, the obtained docking pose was subjected to 100 ns MD simulation. The analysis showed that the predicted binding mode is stable, and the key interactions (cation–pi, salt bridge, and intramolecular hydrogen bond) are conserved during the simulation time. However, the occupancy rates of the additional pi–pi stacking highlighted that such interactions are less preserved throughout the MD simulation (Figure S4).

A good selectivity profile was also observed for compound **1t**, which bears a 3-methylpyrrolidine moiety and displays a distinct inhibition of Spindlin1 (IC_50_: 467 ± 15 nM) coupled with selectivity over G9a (G9a IC_50_ >20 µM, GLP IC_50_ >20 µM). In conclusion, the obtained data underlined that specific substitutions on the pyrrolidine moiety by small substituents like 3-methyl and 3-hydroxymethyl groups as well as replacement of the pyrollidine ring by an isoindoline moiety led to a significant drop in the activity for G9a and GLP whilst maintaining the potent inhibitory activity against Spindlin1. These substitution patterns can hence be used to design selective compounds.

### 2.6. Synthesis

**Scheme 1:**
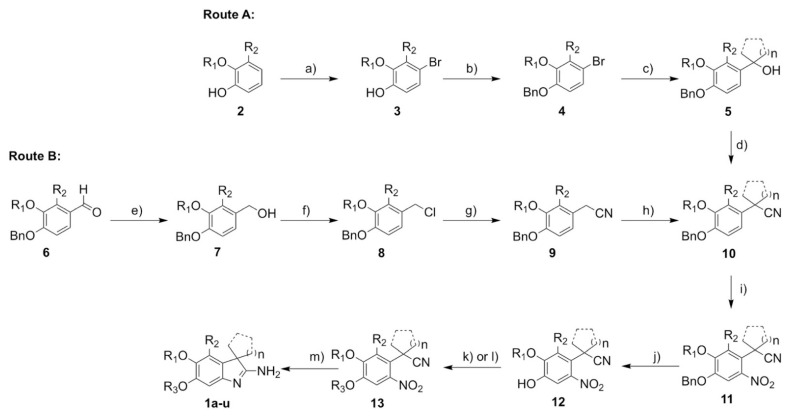
Reagents and conditions: (**a**) Br_2_ (1.1 eq), DCM, −20 to 0 °C, 1 h; (**b**) K_2_CO_3_ (1.2 eq), BnBr (1.2 eq), DMF, 90 °C, 2 h, 91–95% over two steps; (**c**) *n*-BuLi (1.1 eq); ketone (1.1 eq), THF, −78 °C to rt, 1 h, 44–70%; (**d**) TMSCN (3.0 eq); SnCl_4_ (1.0 eq), DCM, −78 °C to rt, 0.5 h, 39–90%; (**e**) LiAlH_4_ (1.5 eq), THF, 0 °C to rt, 4 h, 94–97%; (**f**) conc. HCl (15 eq), Et_2_O, 0 °C to rt, 30 min, 93–98%; (**g**) KCN (1.1 eq), NaI (0.5 eq), DMF, 80 °C, 17 h, 74–79%; (**h**) KOH (7 eq), 1,*n*-dibromo-*n*-alkane (1.0 eq), TBAB (1 mol%), PhMe/H_2_O, reflux, 1.0 h, 56–63%; or NaH (2.5 eq); iodomethane (4 eq), THF, reflux, 16 h, 90%; (**i**) HNO_3_ (1.5 eq), Ac_2_O/AcOH, 0 °C, 1.0 h, 56–92%; (**j**) AlCl_3_ (3.0 eq), DCM, 0 °C, 1 h, 80%-quant.; (**k**) K_2_CO_3_ (1.4 eq), NaI (0.2 eq), 1-(3-chloropropyl)pyrrolidine (1.2 eq), DMF, 80 °C, 1.5 h, 67–92%; (**l**) 1. NaH (1.2 eq); 1,*n*-dihalo-*n*-alkane (1.5 eq), DMF, 0 to 100 °C, 2 h, 2. *N*-heterocyclic compound (2 to 10 eq), DMF, 70 °C, 2–3 h, 30–86% over two steps; (**m**) Zn (10–15 eq), HOAc, 85 °C, 1 h, 61–99%.

All compounds were synthesized from easily accessible starting materials such as guaiacol derivatives **2** (Scheme 1, Route A) or vanillin derivatives **3** (Scheme 1, Route B). In the first route, bromination of **2** was achieved in a regioselective manner at low temperatures in order to obtain phenols **7**, which were directly benzylated and conveniently purified by recrystallization, resulting in excellent yields of intermediates **8**. The organolithium species resulting from subsequent bromine–lithium exchange were then added to a variety of ketone derivatives resulting in benzyl alcohols **5**. Mediated by a Lewis acid activation, benzyl cyanides **10** were obtained by a substitution reaction. After benzylation of their phenol moiety, the second route was initiated by the reduction of aldehydes **6** with lithium aluminum hydride. Benzyl alcohols **7** were generally obtained in excellent yields and could be transformed into benzyl cyanides **9** via two successive substitution steps using first concentrated hydrochloric acid and then potassium cyanide as nucleophile sources. Double alkylation in benzyl position under basic conditions led to either symmetrically dialkylated or cycloalkylated benzyl cyanides **10**, which were intermediates common to both synthetic routes. Nitration was then performed at low temperatures and under mild conditions; the lower yields of the desired regioisomers **11** resulted from poor regioselectivity for certain derivatives. Deprotection of the phenol moiety was selectively achieved using aluminum chloride. Typical hydrogenation conditions involving palladium on carbon as the catalyst partially resulted in a reduction of the nitro group for several derivatives, leading to a significant loss of desired materials **12**. Alkylation of the unprotected phenol moiety was performed either directly with 1-(3-chloropropyl)pyrrolidine under basic conditions or as a two-step sequence including first a terminal dihaloalkane with the desired spacer length and then the *N*-heterocyclic compound of choice. The final step consisted of reducing compounds 13 using zinc dust in acetic acid, delivering the 2-aminoindole derivatives **1a**-**u** resulting from direct ring closure under the chosen conditions.

## 3. Materials and Methods

### 3.1. Synthesis

#### 3.1.1. General Methods and Materials

Reagents and solvents were obtained from commercial sources and used without any further purification. Column chromatography was accomplished using MACHEREY-NAGEL silica gel 60^®^ (230–400 mesh). Thin layer chromatography was performed on aluminum plates pre-coated with silica gel (MERCK, 60 F254) unless specified. TLCs were visualized by UV fluorescence (λ_max_ = 254/320 nm) and/or by staining.

NMR spectra were acquired on a BRUKER Avance 400 spectrometer (400 MHz and 100.6 MHz for 1H and 13C respectively) or a Bruker 500 DRX NMR spectrometer with TBI probe head (499.6 and 125.6 MHz for 1H and 13C respectively) at a temperature of 303 K unless specified. Chemical shifts are reported in parts per million (ppm) relative to residual solvent.

Data for 1H NMR are described as follows: chemical shift (δ in ppm), multiplicity (s, singlet; d, doublet; t, triplet; q, quartet; quin, quintuplet; hept, heptuplet; m, multiplet; br, broad signal), coupling constant (Hz), and integration. Data for 13C NMR are described in terms of chemical shift (δ in ppm).

HR-MS were obtained on a THERMO SCIENTIFIC Advantage and a THERMO SCIENTIFIC Exactive instrument (APCI/MeOH: spray voltage 4–5 kV, ion transfer tube: 250–300 °C, vaporizer: 300–400 °C).

Optical rotation of chiral compounds was determined on a PERKIN-ELMER PE 241 apparatus and transformed for a given temperature according to the following formula (Equation (1)):

(1) $${\left[\alpha \right]}_{D}^{T}=\frac{\alpha \times 100}{c\times l}$$

α: measured value for optical rotation; c: concentration in g/100 mL; l: length of the cuvette in dm; T: temperature in °C.

#### 3.1.2. Synthesis and Analytical Data

Detailed synthetic route for each final derivative as well as syntheses and data for intermediary compounds are given in the Supplementary Materials.

### 3.2. Biological Assays

#### 3.2.1. Fluorescence Polarization Assay

Fluorescence polarization assays were carried out as described before [31]. The inhibitors were diluted in assay buffer (25 mM HEPES pH 7.5, NaCl 100 mM, 0.05% CHAPS, 1 mg/mL BSA) in a serial dilution of 12 inhibitor concentrations with equal DMSO concentrations, and added in a 384-well black non-binding microplate (Greiner Bio-One) to mixtures of H3(1–14)K4me3-Fluorescein (Peptide Specialty Laboratories) and His-Spindlin1(49–262), with final concentrations of 10 and 100 nM, respectively. After 30 min incubation at room temperature, the fluorescence polarization (Ex480, Em535) was measured using an Envision Plate reader (PerkinElmer). For the controls, a buffer solution with the same DMSO concentration as the inhibitor solutions was added to the plate instead of the inhibitor solution, the negative control contained the H3(1–14)K4me3-Fluorescein without Spindlin1. The blank control contained buffer solution with DMSO. Every inhibitor concentration and the controls were done in triplicates. Fluorescence polarization values (mP) were determined after blank correction as $\frac{I_{S}-G\times I_{P}}{I_{S}+G\times I_{P}}\times 1000$, where G is a device-specific factor and IS and IP are the fluorescence intensities of the S and P plane, respectively. Inhibition values were then calculated as $I=100\%\times \left(1-\left(\frac{P_{I}-P_{neg}}{P_{pos}-P_{neg}}\right)\right)$, with PI, *P_neg_*, and *P_pos_* being the fluorescence polarization results of the ligand solution, the negative control, and the positive control, respectively. Curve fitting was carried out using GraphPad Prism 7, applying a non-linear sigmoidal fit with a variable slope. Inhibition curves used for IC_50_ determination are reported in the Supplementary Materials (Figure S5).

#### 3.2.2. Reagents for Histone Methyltransferase Assays

H3 peptide (1–25) is purchased from Anaspec (Cat. NO. AS-61703), thiol fluorescent probe IV is purchased from Millipore-Sigma (Cat. NO. 595504), SAH (S-Adenosyl-L-homocysteine) is purchased from NEB (Cat. NO. B9003S), and adenosine deaminase (ADA) is purchased from Sigma (Cat. NO. 10102105001).

#### 3.2.3. Expression and Purification of G9a and GLP and SAHH

Catalytic domains of Human G9a (913–1193) and GLP (982–1266) were cloned, expressed, and purified as previously described [34]. Full length of S. solfataricus S-adenosylhomocysteine hydrolase (SAHH) was cloned, expressed, and purified using the published protocol [35].

#### 3.2.4. Coupled Enzyme Fluorescent Histone Methyltransferase Assay

The assay was performed using the previously published protocol [35]. Tested compounds were pre-dissolved in assay buffer (20 mM HEPES pH 7.5, 50 mM NaCl, 0.01% Triton X-100, 3 mM MgCl_2_, 0.1 mg/mL BSA) with 5 μM of purified SAHH, 1 unit of ADA, 10 μM of H3, and 5 nM of either G9a or GLP as 30 μL in black 96-well plate. After 5 min, 20 μL of SAM (25 μM) in assay buffer was added. After incubation for 10 min at room temperature, thiol fluorescent probe IV (20 μM, 50 μL) was added. After 10 min, the fluorescence of probe at Ex400/Em465 was measured by Infinite M Plex (Tecan, San Jose, CA, USA). Inhibition curves used for IC_50_ determination are reported in the Supplementary Materials (Figures S6 and S7).

### 3.3. Computational Studies

#### 3.3.1. Docking Studies

The crystal structure of the Spindlin1 in complex with **A366** (PDB ID: 6I8Y) was retrieved from the Protein Data Bank (PDB; www.rcsb.org (accessed on 17 March 2019)) [24] and prepared with Schrödinger’s Protein Preparation Wizard tool [36]. Solvent molecules and sodium ions were removed; meanwhile, hydrogen atoms and missing side chain residues were added to the protein structure. Protonation states were assigned by PROPKA at pH 7.0, and the hydrogen bonding network was consequently optimized. A restrained energy minimization step was then executed using the OPLS3 force field with default settings. 

A Maestro 2D sketcher [37] was used to draw the ligand structures, which were then converted into 3D structures and prepared with Schrödinger’s LigPrep tool [38]. All possible tautomeric forms and stereoisomers were generated at pH 7.0 ± 1.0 using Epik. Afterwards, a multi-conformational dataset was generated with ConfGen, allowing a maximum of 50 conformers per ligand and energy minimization of the output conformations using the default force field (OLPS_2005) [39,40].

Molecular docking studies were carried out by means of Glide using the standard precision (SP) mode [32]. Prior, a grid box was generated employing the Receptor Grid Generation tool, the co-crystallized **A366** inhibitor was selected as the center of the grid, and a cube of 15 Å was defined as the inner box. During docking, the option “sample ring conformation” was switched on, and a maximum of three docking poses were output for each ligand conformer; all other settings were kept as default. Moreover, the contribution of the intramolecular hydrogen bonds was evaluated and incorporated into the docking scores. This was done by allowing the option “reward intramolecular hydrogen bonds” in Glide [32]. In this way, the compounds that exhibit these intramolecular interactions pay a smaller entropic penalty upon the binding, which affects the final scores—referred to as DS_Intra-H-bonds in this article. Instead, DS refers to the default docking scores without the intramolecular hydrogen bonds’ contribution. The above-mentioned protocol was able to reproduce the binding mode of **A366** as observed in the co-crystal structure with Spindlin1 with a root-mean-square deviation (RMSD, heavy atoms) of 0.54 Å when using the top-scored pose (Supplementary Materials, Figure S1).

Box-plots were generated using the R package to evaluate the distribution of the docking scores (DS and DS_Intra-H-bonds) retrieved for the active and inactive ligands. Only the ligands with determined IC_50_ values were used as actives, whereas compounds **1c**, **1d**, and **1e** were treated as inactive.

#### 3.3.2. Molecular Dynamic Simulations

The previously generated docking poses of **A366**, **1c**, and **1e** in complex with Spindlin1 (PDB ID: 6I8Y) were used as initial coordinates for the generation of the MD systems. The simulations were performed using the Amber18 package, and the systems were prepared by means of AmberTools18 tools [33]. First, topologies and force field parameters were assigned to the ligands with the Antechamber package using General Amber Force Field (GAFF) and AM1-BCC as atomic charges method (AM1-BBC, semi-empirical (AM1) with bond charge correction (BCC)) [41,42]. The ligands were considered in the positively charged state; thus, a net molecular charge of 2 was assigned to them. Then, each system was prepared in TLEaP using Amber ff03 force field for the protein and GAFF for the ligands. The systems were solvated in a TIP3P octahedral water box of 10 Å and neutralized using Na^+^ and counterions. The prepared systems were then subjected to a protocol that encompassed diverse steps. Initially, two consecutive minimization steps using constant-volume periodic boundaries were carried out. In each step, 4000 iterations (first 2000 steepest descent and then 2000 conjugate gradient) were applied to the system. In the first minimization step, only solvent atoms were minimized, while the protein and ligand atoms were restrained to their initial coordinates with a force constant of 10 kcal mol^−1^ Å^−2^. In the second minimization step, the positional restraints were removed, and the whole system was minimized. Next, the system was heated, starting from a temperature of 0 K up to 300 K through 100 ps of MD, applying constant-volume periodic boundaries. To prevent large structural deviations, the complex atoms (protein and ligand) were restrained with a force constant of 10 kcal mol^−1^ Å^−2^. SHAKE was turned on and used to constrain bonds involving hydrogen [43]. The Langevin dynamics was employed to control the temperature using a collision frequency of 1 ps^−1^. Then, after a density step, the system was subjected to an equilibration run of 2 ns with a time step of 2 fs per step. The temperature and pressure were kept constant: 300 K and a constant-pressure periodic boundary maintained at an average pressure of 1 atm by using isotropic position scaling with a relaxation time of 2 ps. As for the heating step, SHAKE was turned, while the Langevin dynamics was employed to control the temperature using a collision frequency of 2 ps^−1^.

Finally, each system was subjected to a production run of 200 ns under the same conditions used in the equilibration step (time step of 2 fs per step, constant temperature of 300 K, Langevin dynamics with a collision frequency of 2 ps^−1^, constant-pressure periodic boundary with an average pressure of 1 atm using isotropic position scaling with a relaxation time of 2 ps). In all steps, a cutoff of 10 Å was used for non-bonded interactions, and long-range electrostatic interactions were treated by means of the Particle Mesh Ewald (PME) method [44,45].

The trajectories were analyzed using the CPPTRAJ module [46] from AmberTools18 [33] and visualized in VMD. Plots were generated by means of R package. For the analysis of the protein–ligand hydrogen bonds, the default settings were used, which encompass a distance cutoff of 3 Å between the donor and acceptor atoms and an angle cutoff of 135 degrees. Meanwhile, the distance cutoff was extended to 3.5 Å for the analysis of the ligand intramolecular hydrogen bonds. Two criteria were applied to detect the presence of cation–pi interactions between the ligand and the residues of the aromatic cage (Phe141, Trp151, Tyr170, and Tyr177): (i) a distance cutoff of 6 Å between the center of mass of the positively charged pyrrolidine nitrogen and the centroid of the aromatic ring and (ii) an angle cutoff of 45 degrees; the angle was defined as the angle between the normal vector of the aromatic ring plane and the vector connecting the positively charged pyrrolidine nitrogen and the centroid of the aromatic ring. Salt bridge interactions were considered to be formed if the distance between the negatively charged oxygen atom of Asp184 (or Asp189) and the positively charged amidine nitrogen of the ligand were within the cutoff distance of 4 Å.

All 100,000 frames retrieved from the 200 ns MDs were subjected to the analysis. The occupancy rates of the interactions were calculated as the percent of the frames in which a specific interaction was observed.

#### 3.3.3. Prime MM-GBSA

The first docking poses were rescored with Prime MM-GBSA, a tool implemented in Schrödinger suite that performs protein–ligand complex minimization and refinement [47]. The VSGB solvation model and OPLS3 force field were used [48]. Several settings were tested, and we report to two different protocols: (i) only the ligands were relaxed, and (ii) also the protein residues within 3 Å from the ligands were treated as flexible. For the analysis of the results, for the docking studies, box-plots were generated using the R package to show the distribution of the computed binding affinity values (MM-GBSA ΔG_bind_) obtained for the active and inactive ligands. Only the ligands with determined IC_50_ values were used as actives, whereas compounds **1c**, **1d**, and **1e** were treated as inactive compounds.

## 4. Conclusions

In previous work, we reported the discovery of **A366**, an originally developed G9a inhibitor [30], as a nanomolar Spindlin1 inhibitor (Figure 1) [31]. Later, the crystal structure of Spindlin1 in complex with **A366** was resolved (PDB ID: 6I8Y, [27]), and some modifications, as well as bivalent derivatives, were investigated [27]. In this study, we synthesized, tested, and computationally analyzed a series of 21 **A366** derivatives as Spindlin1 inhibitors to further explore the SAR of this series. The main modification that had a drastic impact on the activity was the length of the linker chain, where the actives bear a propyl chain, while the inactive compounds either an ethyl or a butyl chain. Since the pyrrolidine function mimics the trimethylated lysine moiety, it is crucial for the inhibitory activity. Minor modification of the pyrrolidine ring did not lead to significant changes in the inhibitory activity (e.g., **1q**), whereas its replacement with an isoindoline moiety yielded **1s**, a good Spindlin1 binder, and the most selective compound of our series (Spindlin1 IC_50_: 360 ± 20 nM, G9a IC_50_ >20 µM, and GLP IC_50_ >20 µM). Likewise, a good selectivity profile was observed for compound **1t** bearing a 3-methylpyrrolidine moiety and a 3-ethyl-3-methyl substitution on R4. Substituents on R2 position were not well tolerated, as highlighted by a significant decrease in the inhibitory activity of several compounds (**1a**, **1h**, **1i**, **1j**, and **1k**). Meanwhile, the replacement of the methoxy group at R1 displayed minor (**1g**, **1l**) to more significant (**1m**) decreases in the activity based on the substitutions. Finally, modifications of the spiro moiety showed no major impact on the inhibitory activity of the compounds; more relevant effects were observed with the geminal dimethyl and diethyl modifications. Notably, compound **1p** was the most active derivative of the series (Spindlin1 IC_50_: 157 ± 12 nM) but showed no selectivity over G9a; while compound **1o** exhibited a decreased activity for Spindlin1 coupled with an increase in selectivity over G9a and GLP (Spindlin1 IC_50_: 1.21 ± 0.09 µM, G9a IC_50_ >20 µM, GLP IC_50_ >20 µM). The synthesis and in vitro testing were coupled with various computational approaches. Docking studies and MD simulations were carried out to rationalize the lack of activity of some derivatives. Interestingly, while from the predicted binding modes and docking scores it was not possible to explain the differences in the activity, the analysis of the MD simulations shed light on the important interactions and their contributions. Moreover, rescoring the docking poses with MM-GBSA calculations was successful in discriminating active from inactive compounds within our congeneric series of **A366** inhibitors.

Considering the SAR of our series and the structural features of the binding site of Spindlin1 and G9a, future work will concentrate on attempting more substitutions on R2 and exploring other replacement for the pyrrolidine moiety. Specifically, longer and hydrophilic substitutions will be tested on R2, which are postulated, on the one hand, to be able to interact with the surrounding Spindlin1 residues (e.g., Asp95 and Glu142), and on the other clash with the smaller G9a pocket and ensure selectivity. Additionally, bisubstitutions on the pyrrolidine moiety as well as other bicyclic basic moieties will be probed to achieve further potent and selective compounds.

It was reported that high levels of Spindlin1 have been observed in liposarcoma and other types of tumors including ovarian cancer [13]. Thus, targeting the Spindlin1/H3K4me3 pocket with small molecule inhibitors as described in the present work might be an interesting therapeutic approach for such cancer treatment. The developed potent and selective Spindlin1 inhibitors are drug-like molecules that can be used for further cellular studies to achieve this goal.

## Figures and Tables

**Table 1:** Chemical structures and inhibitory activity of the **A366** derivatives.

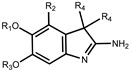							
Compounds	R1	R2	R3	R4	Spin1 IC_50_ ± SD (nM) ^[a]^	G9a IC_50_ (nM) ^[b]^	GLP IC_50_ (nM) ^[b]^
**A366**	Me	H	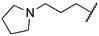	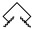	182.6 ± 9.1 [31]	38 ^[c]^	51
**1a**	Et	OEt	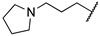	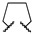	70% @ 25 μM ^[d]^		
**1b**	Me	H	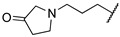	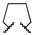	81% @ 100 μM ^[d]^		
**1c**	Me	H	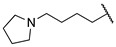	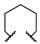	42% @ 50 μM ^[d]^	>20,000	>20,000
**1d**	Me	H	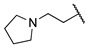	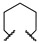	Inactive		
**1e**	Me	H	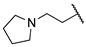	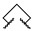	Inactive		
**1f**	Me	H	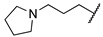	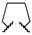	169 ± 13	431	334
**1g**	Et	H	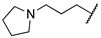	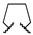	316 ± 26	243	2969
**1h**	Me	CN	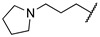	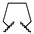	3900 ± 700		
**1i**	Me	Cl	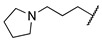	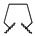	1600 ± 200	>20,000	>20,000
**1j**	Me	OMe	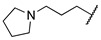	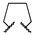	2500 ± 200		
**1k**	Me	Ac	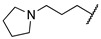	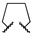	11,800 ± 210		
**1l**	Et	H	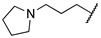	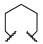	567 ± 35		
**1m**	2-Pr	H	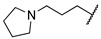	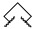	3300 ± 470		
**1n**	Me	H	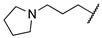	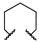	255 ± 35	779	527
**1o**	Me	H	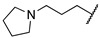	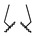	1210 ± 90	>20,000	>20,000
**1p**	Me	H	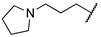	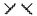	157 ± 12	759	470
**1q**	Me	H	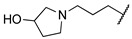	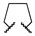	(R): 470 ± 27 (S): 478 ± 34 (rac): 584 ± 31	(rac): 2321	(rac): 4432
**1r**	Me	H	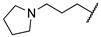	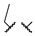	408 ± 35	3191	3185
**1s**	Me	H	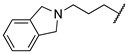	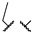	360 ± 20	>20,000	>20,000
**1t**	Me	H	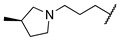	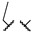	467 ± 15	>20,000	>20,000
**1u**	Me	H	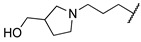	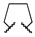	1250 ± 50	>20,000	>20,000

^[a]^ Fluorescence polarization assay, based on competition to a Fluorescein-H3K4me3 peptide. ^[b]^ Fluorescence based coupled enzyme assay, based on SAM-SAH conversion. For details, see experimental part. ^[c]^ Reported IC_50_ G9a for **A366**: 3.3 nM [30]. ^[d]^ If no IC_50_ was obtained, inhibition is reported in percentage at specified concentration.

## Data Availability

Not applicable.

## Supplementary Materials

The following are available online at https://www.mdpi.com/article/10.3390/ijms22115910/s1.
