# Supplementary material for: Structure-Based Design, Docking and Binding Free Energy Calculations of A366 Derivatives as Spindlin1 Inhibitors
Source: Int J Mol Sci. 2021 May 31;22(11):5910. doi: 10.3390/ijms22115910 (PMC8199216; doi:10.3390/ijms22115910)
Supplement: Supplementary file 1 [file ijms-22-05910-s001.zip › ijms-1230109-supplementary.pdf]

Supplementary Materials for "Structure-based design, docking and binding free energy calculations of A366 derivatives as Spindlin1 inhibitors"

Contents:

Figure S1. Superimposition of the re-docking pose and the co-crystallized pose of **A366** in the Spindlin1 second domain binding pocket.

Figure S2. Snapshots retrieved from 200 ns MD simulation of **1e** at 50 ns, 100 ns, 150 ns and 190 ns.

Figure S3. Distance variation between the side chain of Asp184 and the positively charged amidine of **A366**, **1c** and **1e**.

Figure S4. Analysis of 100 ns MD simulation of compound **1s**.

Table S1. ΔGbind, docking scores and salt bridge distances of the docked poses.

Figure S5. Spindlin1 inhibition curves used for IC_{50} determination.

Figure S6. G9a inhibition curves used for IC_{50} determination.

Figure S7. GLP inhibition curves used for IC_{50} determination.

Synthesis - General Methods and Materials

Synthesis - General procedure

Synthesis and analytical data of intermediate and final derivatives

NMR spectra

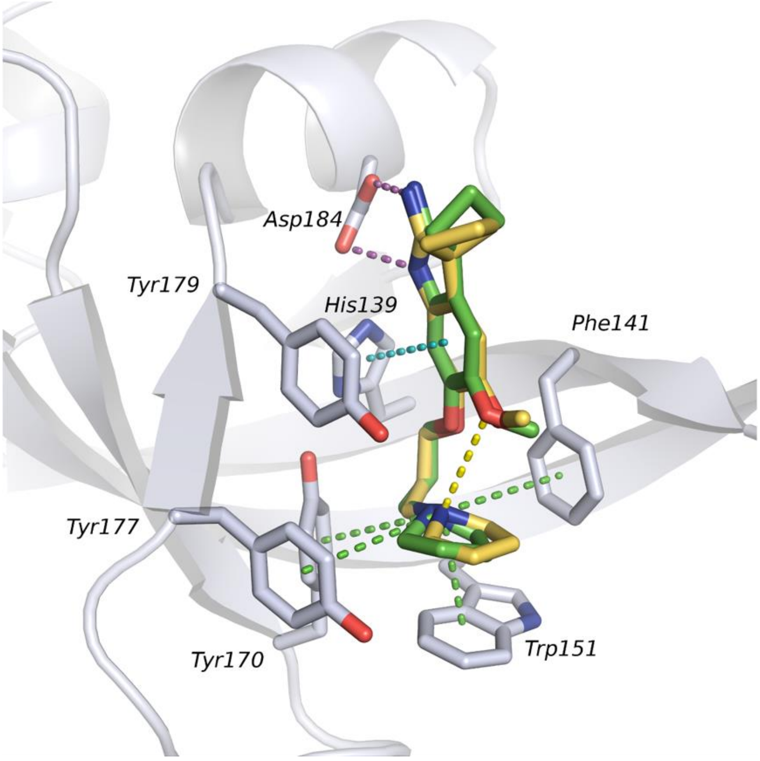

Figure S1. Superimposition of the re-docking pose (green) and the co-crystallized pose (yellow) of **A366** in the Spindlin1 second domain binding pocket (PDB ID: 6I8Y). Only surrounding amino acid residues are displayed for clarity. Docking poses and side chains of the surrounding residues are shown as sticks. Binding interactions are represented with dashed lines colored in yellow (hydrogen bond), green (cation-pi), cyan (pi-pi stacking) and magenta (salt bridge).

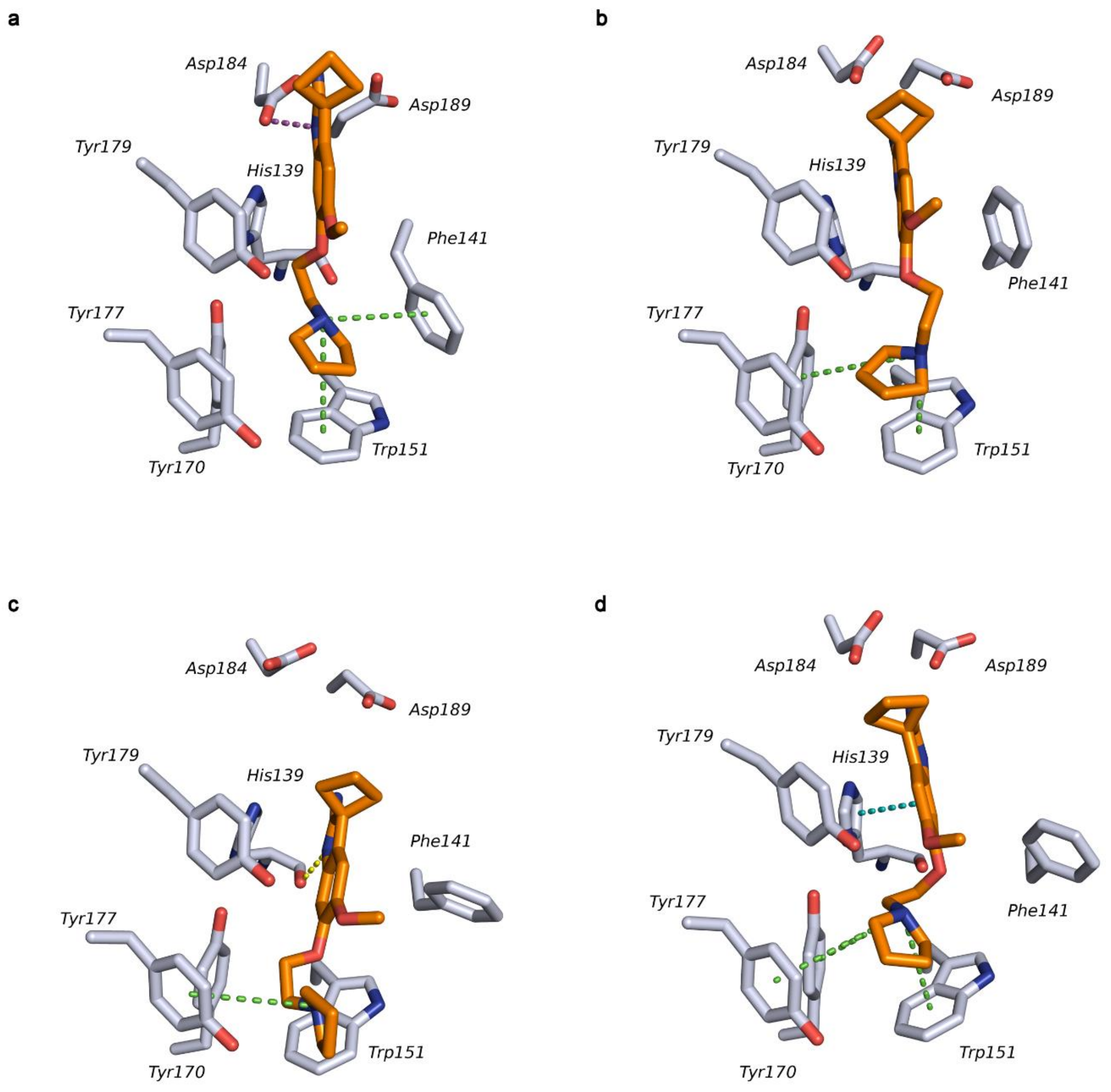

Figure S2. Snapshots retrieved from 200 ns MD simulation of **1e** at (**a**) 50 ns, (**b**) 100 ns, (**c**) 150 ns (**d**) 190 ns. The initial binding pose obtained from docking **1e** into Spindlin1 second domain binding site (PDB ID: 6I8Y). Only the side chains of the surrounding residues are shown for clarity and they are depicted as sticks; for residue His139 the main chain is shown. Binding interactions are represented with dashed lines colored in yellow (hydrogen bond), green (cation-pi), cyan (pi-pi stacking) and magenta (salt bridge).

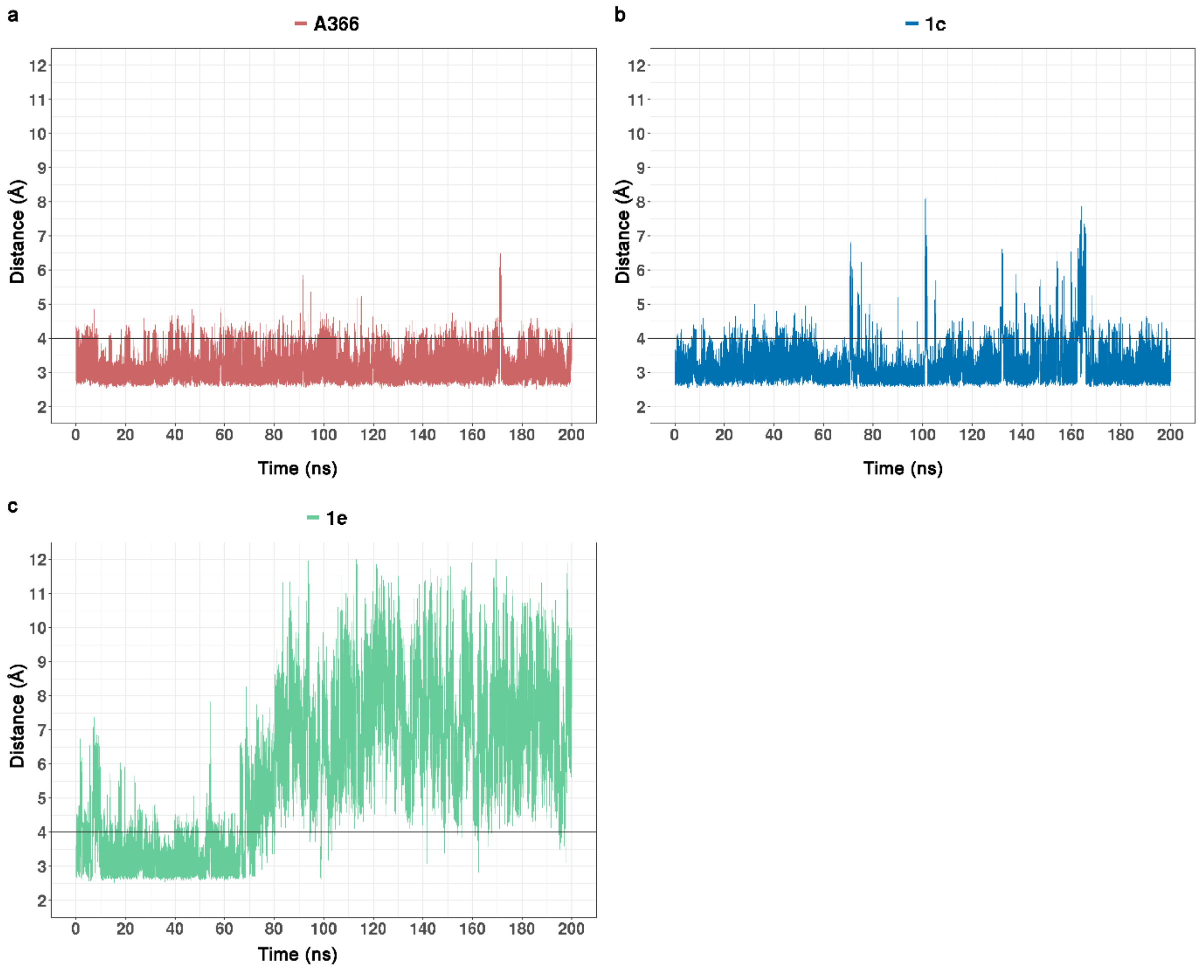

Figure S3. Distance variation between the side chain of Asp184 and the positively charged amidine of **A366** (**a**, red line), **1c** (**b**, blue line) and **1e** (**c**, green line).

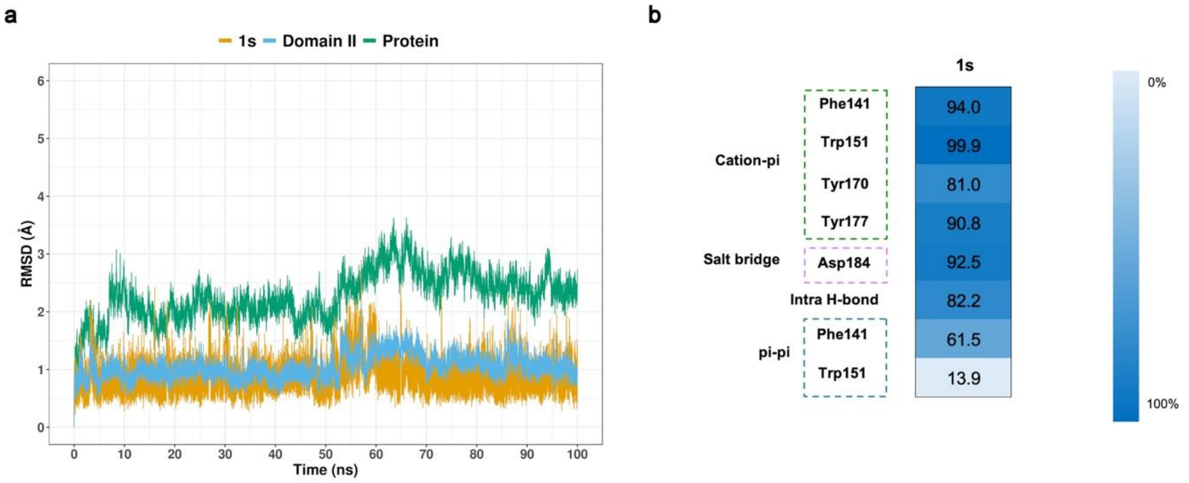

Figure S4. Analysis of 100 ns MD simulation of compound **1s**. **(a)** Root mean square deviation (RMSD) plot. (**b**) Heat map showing the occupancy rates of the interactions during the simulation. The initial binding pose was obtained from docking into Spindlin1 second domain binding site (PDB ID: 6I8Y).

Compounds	ΔGbind [a]	ΔGbind–3 Å [b]	DS [c]	DS_Intra-H-bonds [d]	*Salt bridge dist. (Asp184)* [e]
**A366**	-74.10	-75.06	-11.37	-11.87	3.01
**1a**	-75.36	-76.83	-11.27	-11.77	3.00
**1b**	-76.77	-76.30	-10.92	-11.92	3.03
**1c**	-56.05	-58.75	-11.12	-11.12	3.65 [f]
**1d**	-62.54	-64.48	-11.16	-11.16	3.03
**1e**	-62.46	-64.58	-11.28	-11.28	3.02
**1f**	-74.41	-73.95	-11.03	-12.03	3.04
**1g**	-76.09	-75.47	-11.26	-11.76	3.00
**1h**	-75.11	-75.75	-11.30	-11.80	3.03
**1i**	-74.69	-75.61	-11.23	-11.73	3.03
**1j**	-73.91	-75.65	-11.45	-11.95	2.93
**1k**	-76.20	-77.66	-11.41	-11.91	2.96
**1l**	-75.86	-75.18	-11.24	-11.74	3.01
**1m**	-75.45	-74.94	-11.32	-11.82	2.94
**1n**	-74.67	-74.51	-11.01	-12.01	3.05
**1o**	-74.61	-74.07	-11.39	-11.89	2.95
**1p**	-74.27	-73.73	-11.34	-11.84	2.95
**1q_R**	-80.65	-80.44	-11.85	-12.35	2.98
**1q_S**	-82.45	-81.91	-11.75	-12.75	2.99
**1r_R**	-74.26	-73.51	-11.13	-12.13	3.04
**1r_S**	-74.94	-75.27	-11.46	-11.96	3.03
**1s_R**	-76.62	-76.58	-11.57	-12.07	3.02
**1s_S**	-77.12	-77.41	-11.59	-12.09	3.03
**1t_R-R**	-74.96	-74.39	-11.33	-11.83	2.97
**1t_S-R**	-75.39	-75.27	-11.31	-11.81	2.93
**1u_R**	-81.96	-81.58	-11.95	-12.45	2.98
**1u_S**	-81.12	-81.89	-12.45	-12.95	3.03

Table S1. ΔGbind, docking scores and salt bridge distances of the docked poses. [a] MM-GBSA ΔGbind in kcal/mol computed using default setting, only ligands are relaxed. [b] MM-GBSA ΔGbind in kcal/mol computed treating ligands and protein residues around 3 Å from the ligands as flexible. [c] Docking Score. [d] Docking Score with reward intramolecular hydrogen bonds contributions. [e] Distance measured from negatively charged oxygen atom of Asp184 (for the exception of compound **1c**) and the positively charged amidine nitrogen of the ligand. [f] Distance measured from negatively charged oxygen atom of Asp189 and the positively charged amidine nitrogen of **1c**.

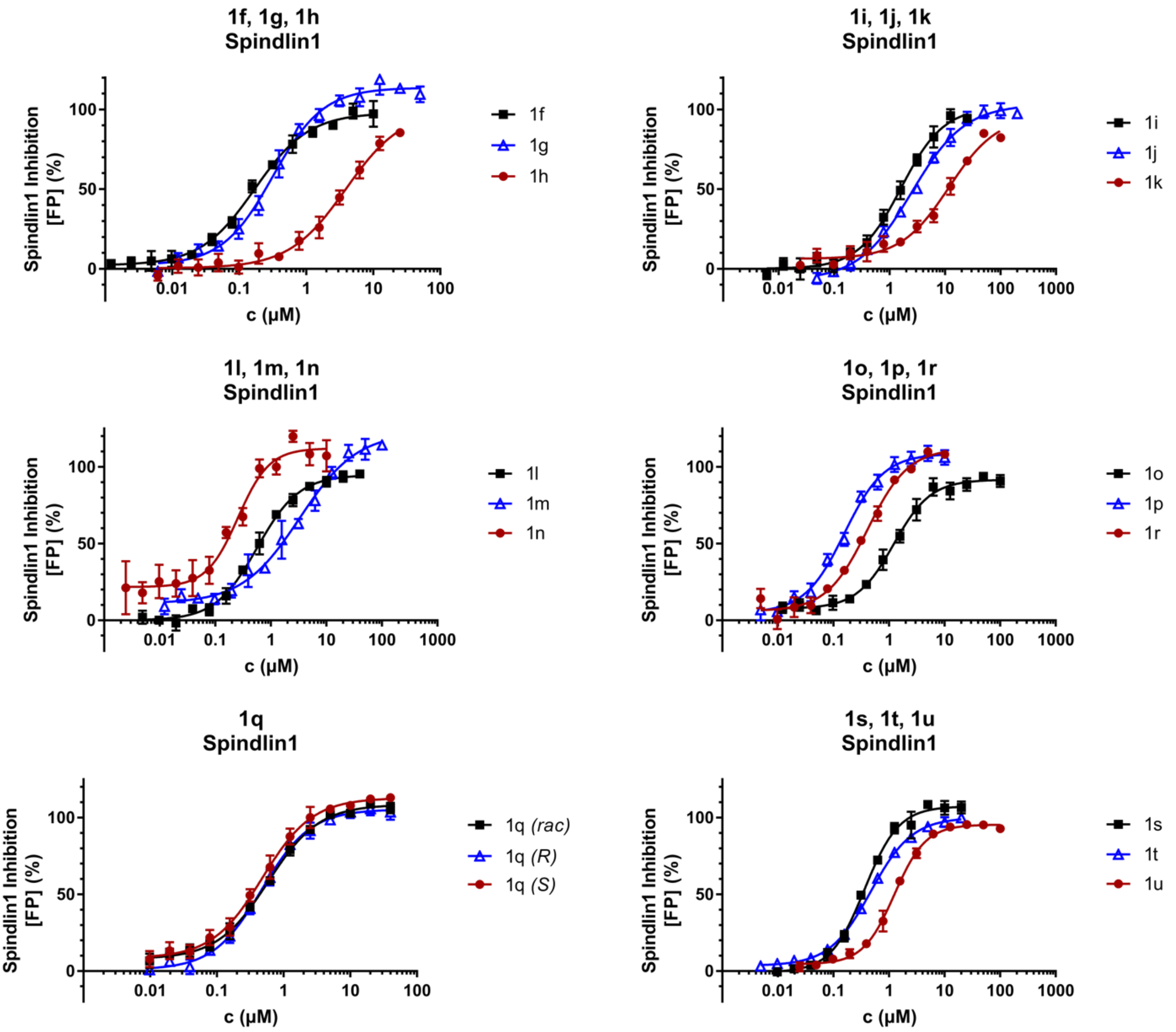

Figure S5. Spindlin1 inhibition curves used for IC$_{50}$ determination.

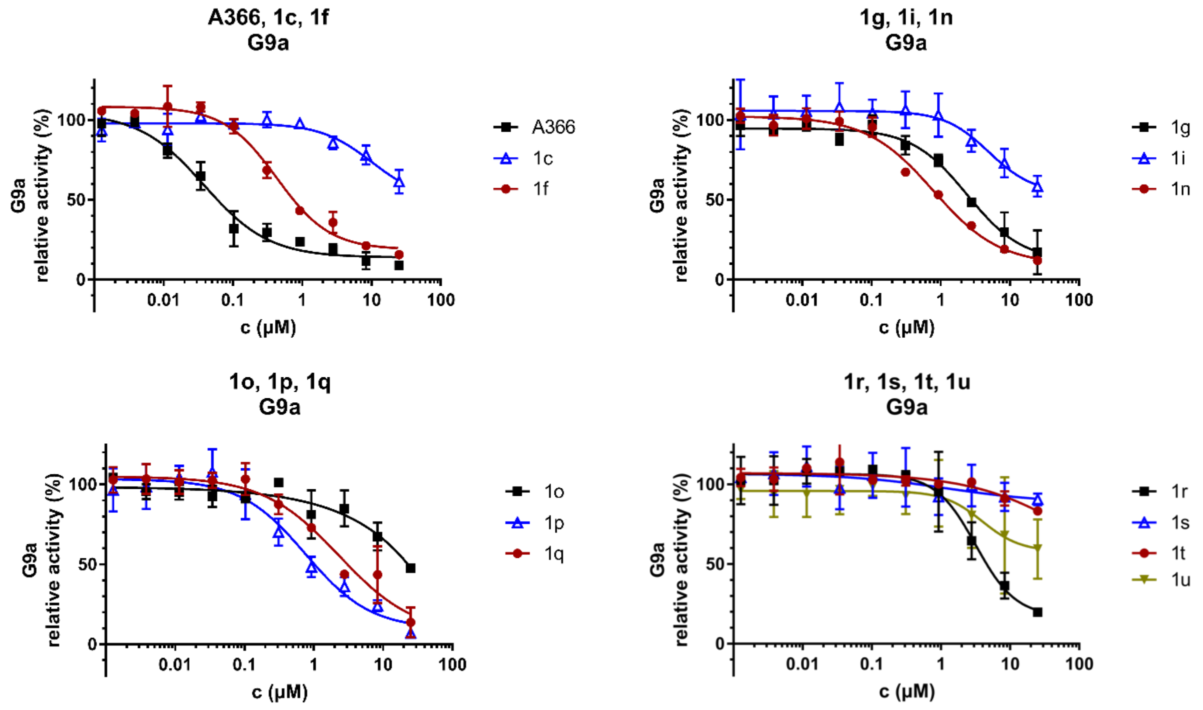

Figure S6. G9a inhibition curves used for IC_{50} determination.

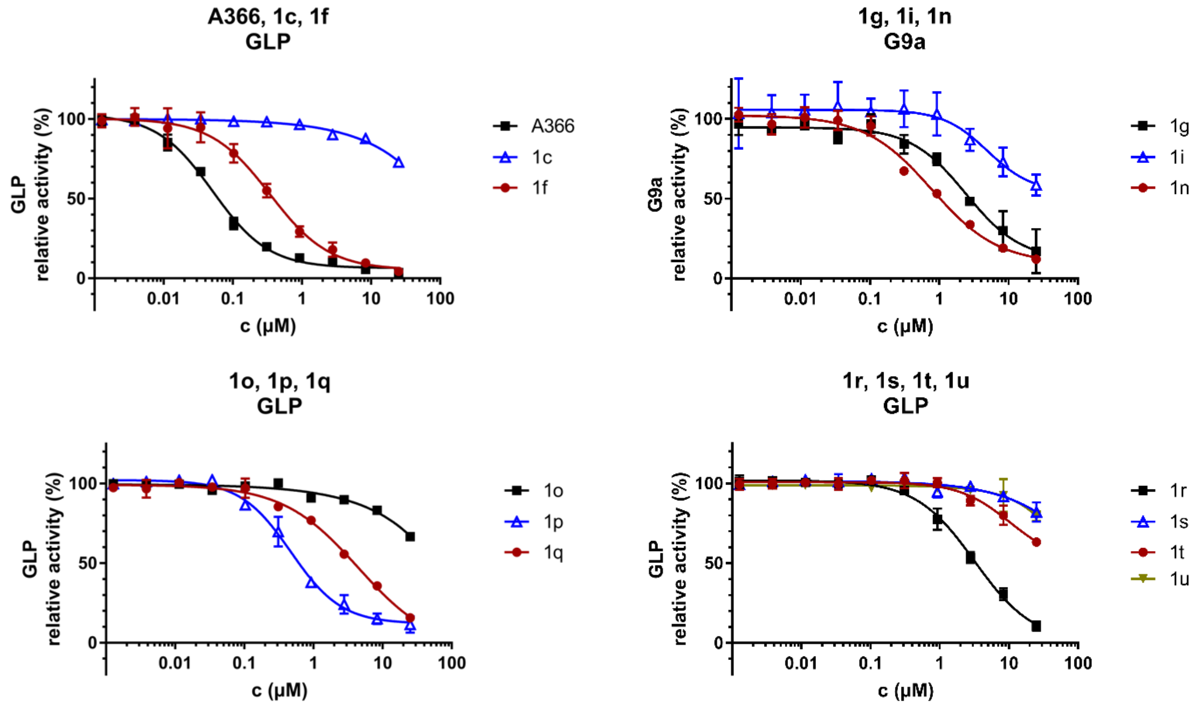

Figure S7. GLP inhibition curves used for IC_{50} determination.

Synthesis - General Methods and Materials

Reagents and solvents were obtained from commercial sources and used without any further purification. Column chromatography was accomplished using MACHEREY-NAGEL silica gel 60® (230-400 mesh). Thin layer chromatography was performed on aluminum plates pre-coated with silica gel (MERCK, 60 F_{254}) unless specified. TLCs were visualized by UV fluorescence (λmax = 254/320 nm) and/or by staining. Compound purity was determined on the basis of HPLC and NMR experiments as following:

NMR spectra were acquired on a BRUKER Avance 400 spectrometer (400 MHz and 100.6 MHz for ^{1}H and ^{13}C respectively) or a Bruker 500 DRX NMR spectrometer with TBI probe head (499.6 MHz and 125.6 MHz for 1H and 13C respectively) at a temperature of 303 K unless specified. Chemical shifts are reported in parts per million (ppm) relative to residual solvent.

Data for 1H NMR are described as following: chemical shift (δ in ppm), multiplicity (s, singlet; d, doublet; t, triplet; q, quartet; quin, quintuplet; hept, heptuplet; m, multiplet; br, broad signal), coupling constant (Hz), integration.

Data for 13C NMR are described in terms of chemical shift (δ in ppm).

HR-MS were obtained on a THERMO SCIENTIFIC Advantage and a THERMO SCIENTIFIC Exactive instrument (APCI/MeOH: spray voltage 4-5 kV, ion transfer tube: 250-300 °C, vaporizer: 300-400 °C).

Synthesis - General procedure

General procedure for benzyl deprotection

A solution of the benzyl protected compound (0.94 mmol, 1 equiv.) in DCM (12.7 mL) was cooled down to 0 °C. $AlCl_3$ (253 mg, 1.90 mmol, 3 equiv.) was added portionwise and the resulting suspension was stirred at 0 °C for 1 h. The reaction was quenched by addition of an aqueous solution of HCl (12.7 mL, 1M) and diluted with DCM. The reaction mixture was allowed to warm up to rt and was extracted three times with DCM. The combined organic layers were washed with brine, dried over Na_2SO_4, filtered and concentrated under reduced pressure. The residue was purified by column chromatography to afford the desired compound.

Synthesis and analytical data of intermediates and final derivatives

1. **<u>4-(Benzyloxy)-2-iodo-3-methoxybenzaldehyde (18)</u>**

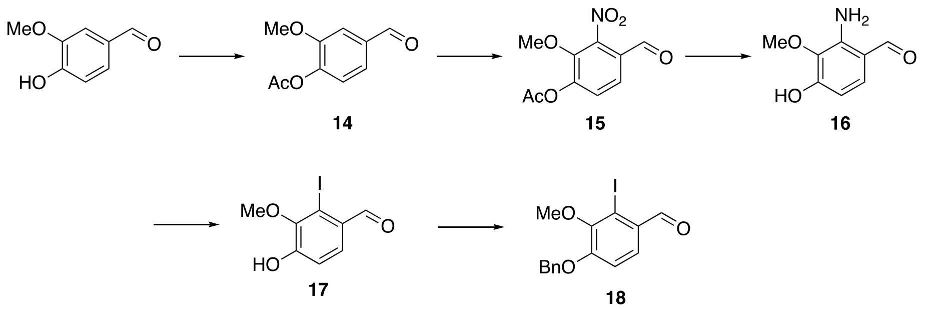

4-Formyl-2-methoxyphenyl acetate (14)

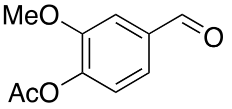

To a solution of vanillin (8.00 g, 52.6 mmol, 1 equiv.) in anhydrous pyridine (50 mL) at 0 °C was slowly added acetic anhydride (7.44 mL, 78.9 mmol, 1.5 equiv.). The solution was allowed to warm at rt and stirred overnight. The solution was then evaporated under reduced pressure. Water and EtOAc were added to the residue. The aqueous phase was extracted twice with EtOAc. The combined organic phase was washed with brine, dried over Na_2SO_4 and evaporated to afford the desired product (9.94 g, 51.2 mmol, **97%**) as a white solid. **R***f* = 0.53 (30% EtOAc in cyclohexane); **m.p.**: 69 – 71 °C; **^{1}H NMR** (400 MHz, $CDCl_3$) δ 9.90 (s, 1H), 7.45 (d, *J* = 1.8 Hz, 1H), 7.43 (dd, *J* = 8.0, 1.9 Hz, 1H), 7.17 (d, *J* = 8.0 Hz, 1H), 3.86 (s, 3H), 2.29 (s, 3H); **^{13}C NMR** (101 MHz, $CDCl_3$) δ 191.1, 168.4, 152.1, 145.1, 135.4, 124.8, 123.6, 111.1, 56.2, 20.7

4-Hydroxy-3-methoxy-2-nitrobenzaldehyde (15)

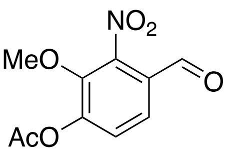

4-Formyl-2-methoxyphenyl acetate (**14**, 9.94 g, 1 mmol, 1 equiv.) was added to a cold solution of fuming HNO_3 (53.4 mL, 1.28 mol, 25 equiv.) using an ice bath. The reaction mixture was stirred for 3 h, keeping the temperature under 0 °C. The cooling bath was removed, the reaction was poured

into ice and the mixture was stirred at rt until the precipitation of a yellow solid was complete. The solution was poured in ice-water and the precipitate was filtered, washed with water and dried to afford the desired product (9.94 g, 41.6 mmol, **81%**) as a light yellow solid. **R***f* = 0.26 (30% EtOAc in cyclohexane); **^{1}H NMR** (400 MHz, $CDCl_3$) δ 9.87 (s, 1H), 7.78 (d, *J* = 8.5 Hz, 1H), 7.42 (d, *J* = 8.5 Hz, 1H), 3.92 (s, 3H), 2.38 (s, 3H); **^{13}C NMR** (101 MHz, $CDCl_3$) δ 185.9, 167.7, 149.3, 145.8, 144.6, 126.3, 126.2, 125.7, 63.1, 21.0; Spectral data matched previously reported values.

The obtained product, 4-formyl-2-methoxy-3-nitrophenyl acetate, (9.94 g, 41.6 mmol, 1 equiv.) was hydrolysed with a solution of KOH (150 mL, 2M) at 100 °C for 10 min. After cooling, the reaction mixture was acidified with concentrated HCl to afford the desired product as a yellow precipitate, which was filtered and washed with water (6.47 g, 32.8 mmol, **80%**). **R***f* = 0.15 (30% EtOAc in cyclohexane); **m.p.**: 113 – 115 °C; **^{1}H NMR** (400 MHz, Acetone-*d*6) δ 10.5 (br s, 1H), 9.82 (s, 1H), 7.75 (d, *J* = 8.5 Hz, 1H), 7.32 (d, *J* = 8.5 Hz, 1H), 3.94 (s, 3H); **^{13}C NMR** (101 MHz, Acetone-*d*6) δ 187.6, 157.8, 145.3, 140.8, 130.7, 120.8, 118.9, 62.6; **HRMS** (neg. ESI): calcd. for $C_8H_6NO_5$ $[M-H]^-$: 196.02460, found: 196.0251

2-Amino-4-hydroxy-3-methoxybenzaldehyde (16)

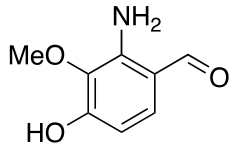

To a solution of 4-hydroxy-3-methoxy-2-nitrobenzaldehyde (**15**, 6.47 g, 32.8 mmol, 1 equiv.) in EtOH (60 mL) and water (20 mL) were added $Fe(SO_4).7\ H_2O$ (1.64 g, 5.91 mmol, 0.18 equiv.) and iron powder (16.5 g, 295.3 mmol, 9 equiv.). The solution was heated to reflux for 8 h, filtrate and evaporated. The residue was purified on silica gel column eluting with 20-40% EtOAc in cyclohexane to afford the desired product as an orange-brown solid (4.85 g, 29.0 mmol, **88%**). **R***f* = 0.40 (40% EtOAc in cyclohexane); **m.p.**: 138 – 140 °C; **^{1}H NMR** (400 MHz, Acetone-*d*6) δ 9.69 (s, 1H), 8.91 (br s, 1H), 7.19 (d, *J* = 8.6 Hz, 1H), 6.68 (br s, 2H), 6.32 (d, *J* = 8.6 Hz, 1H), 3.76 (s, 3H); **^{13}C NMR** (101 MHz, Acetone-*d*6) δ 192.6, 155.9, 146.5, 133.8, 133.4, 115.1, 106.5, 59.8

4-hydroxy-2-iodo-3-methoxybenzaldehyde (17)

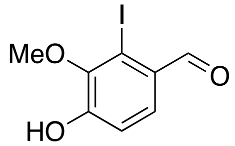

To a solution of 2-amino-4-hydroxy-3-methoxybenzaldehyde (**16,** 2.10 g, 12.6 mmol, 1 equiv.) in HCl 20% (25 mL) at 0 °C was slowly added a solution of NaNO2 (944 mg, 13.7 mmol, 1.09 equiv.) in water (8 mL). The stirring was continued for 15 min. A solution of KI (10.4 g, 62.8 mmol, 5 equiv.) was added slowly. The solution was allowed to warm at rt and stirring was continued for 16 h. $CHCl_3$ (60 mL) and $Na_2S_2O_3$ were added and the chloroform layer was separated, washed with brine, then dried over Na_2SO_4. Removal of the solvent under vacuum followed by purification on silica gel column afford the desired product (2.71 g, 9.75 mmol, 78%) as a yellow solid. **R***f* = 0.36 (25% EtOAc in cyclohexane); **m.p.**: 154 – 156 °C; **^{1}H NMR** (400 MHz, Acetone-*d*6) δ 9.94 (d, *J* = 0.6 Hz, 1H), 9.52 (br s, 1H), 7.57 (d, *J* = 8.5 Hz, 1H), 7.08 (dd, *J* = 8.5, 0.6 Hz, 1H), 3.86 (s, 3H); **^{13}C NMR** (101 MHz, Acetone-*d*6) δ 194.6, 156.8, 148.8, 129.6, 128.3, 117.9, 100.6, 60.8; **HRMS** (neg. APCI): calcd. for $C_8H_6O_3I$ $[M-H]^-$: 276.9364, found : 276.93617

4-(Benzyloxy)-2-iodo-3-methoxybenzaldehyde (18)

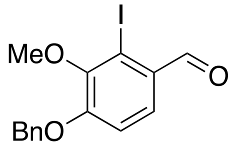

To a solution of 4-hydroxy-2-iodo-3-methoxybenzaldehyde (**17**, 3.43 g, 12.3 mmol, 1 equiv.) in DMF (20 mL) were added K_2CO_3 (2.05 g, 14.8 mmol, 1.2 equiv.) and benzyl bromide (1.76 mL, 14.8 mmol, 1.2 equiv.). The mixture was heated overnight at 90 °C. Then, the reaction mixture was allowed to cool to rt, evaporated and the residue was poured into water. The aqueous phase was extracted three times with EtOAc. The combined organic layers were dried over Na_2SO_4, filtered and concentrated under reduced pressure. Purification on silica gel column eluting with 10-20% EtOAc in cyclohexane afforded the desired compound (4.54 g, 12.3 mmol, **100%**) as a white solid. **R***f* = 0.41 (10% EtOAc in cyclohexane); **m.p.**: 126 – 128 °C; **^{1}H NMR** (400 MHz, $CDCl_3$) δ 10.0 (d, *J* = 0.7 Hz, 1H), 7.65 (d, *J* = 8.6 Hz, 1H), 7.44 – 7.31 (m, 5H), 7.00 (dd, *J* = 8.6, 0.7 Hz, 1H), 5.20 (s, 2H), 3.89 (s, 3H); **^{13}C NMR** (101 MHz, $CDCl_3$) δ 195.1, 156.9, 149.4, 135.8, 129.5, 129.0 (2C), 128.7, 127.5 (2C), 127.4, 113.6, 100.7, 71.2, 60.8; **HRMS** (pos. ESI): calcd. for $C_{15}H_{13}IO_3Na$ $[M+Na]^-$: 390.98072, found: 390.9802

1-(3-Chloropropyl)pyrrolidine (19)

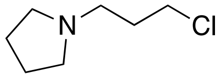

To a solution of pyrrolidine (11.8 mL, 127 mmol, 2 equiv.) in Et_2O (40 mL) at 0 °C was slowly added a solution of 1-bromo-3-chloropropane (6.28 mL, 63.6 mmol, 1 equiv.). The solution was allowed to warm at rt and was stirred for 16 h. An aqueous solution of NaOH (20%) and Et_2O were added. The aqueous phase was extracted twice with Et_2O. The combined organics were washed with water, brine, dried over Na_2SO_4 and evaporated under reduced pressure. Distillation of the residue (90 °C, 10 mbar) afforded the desired product as a colorless oil (5.42 g, 36.7 mmol, **58%**). **R***f* = 0.28 (100% EtOAc); **^{1}H NMR** (400 MHz, $CDCl_3$) δ 3.60 – 3.55 (m, 2H), 2.57 – 2.53 (m, 2H), 2.49 – 2.45 (m, 4H), 1.99 – 1.91 (m, 2H), 1.79 – 1.69 (m, 4H); **^{13}C NMR** (101 MHz, $CDCl_3$) δ 54.4, 53.8, 43.6, 32.2, 23.7; **HRMS** (pos. ESI): calcd. for $C_7H_{15}NCl$ $[M+H]^+$: 148.0893, found : 148.0887

2. 4',5'-Diethoxy-6'-(3-(pyrrolidin-1-yl)propoxy)spiro[cyclopentane-1,3'-indol]-2'-amine (1a)

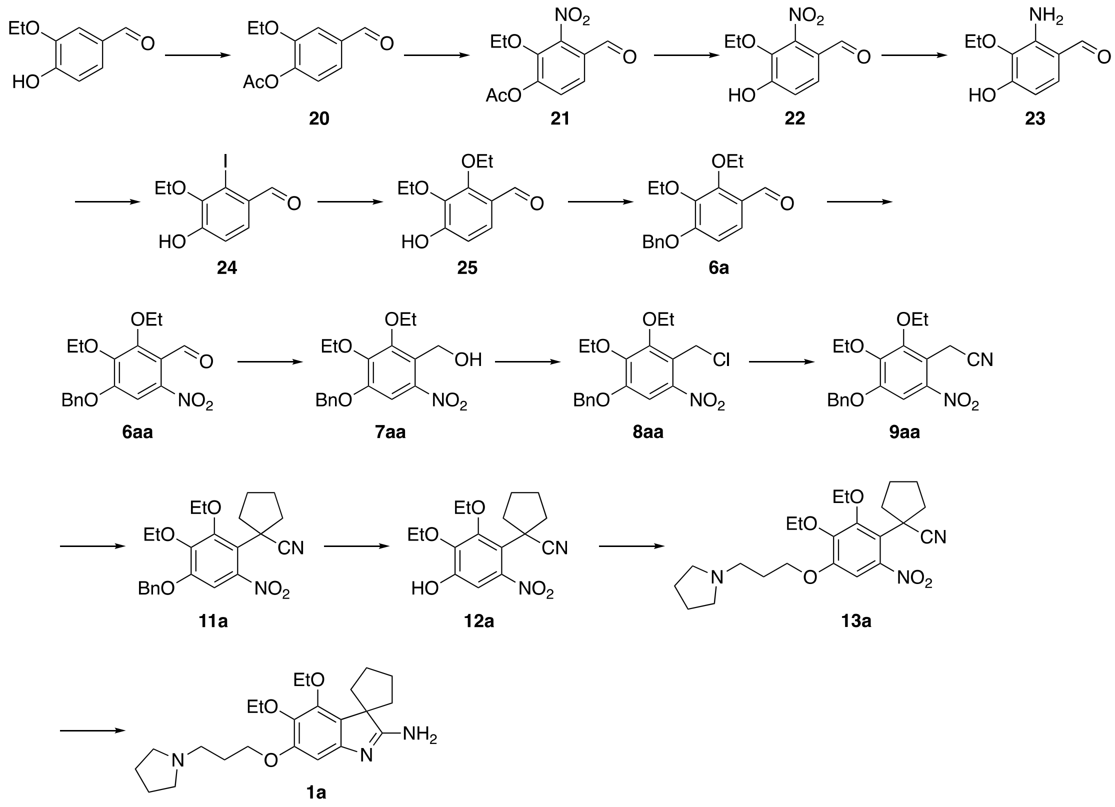

2-Ethoxy-4-formylphenyl acetate (20)

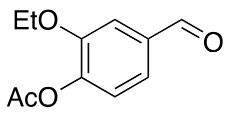

To a solution of 3-ethoxy-4-hydroxybenzaldehyde in pyridine (50 mL) at 0 °C was added slowly acetic anhydride (6.81 mL, 72.2 mmol, 1.5 equiv). The solution was allowed to warm at rt and stirred overnight. The solution was then evaporated under reduced pressure. Water and EtOAc were added to the residue. The aqueous phase was extracted twice with EtOAc. The combined organic phase was washed with brine, dried over Na_2SO_4 and evaporated to afford the desired product (9.91 g, 47.6 mmol, **99%**) as a white solid. **R**$_f$ = 0.61 (33% EtOAc in cyclohexane); **m.p.:** 53 – 55 °C; **^{1}H NMR** (400 MHz, $CDCl_3$) δ 9.88 (s, 1H), 7.43 (dd, *J* = 1.8, 0.3 Hz, 1H), 7.41 (dd, *J* = 8.0, 1.8 HZ, 1H), 7.16 (d, *J* = 8.0 Hz, 1H), 4.08 (q, *J* = 7.0 H, 2H), 2.28 (s, 3H), 1.37 (t, *J* = 7.0 Hz, 3H); **^{13}C NMR** (101 MHz, $CDCl_3$) δ 191.1, 168.4, 151.4, 145.4, 135.3, 124.6, 123.5, 112.0, 64.7, 20.6, 14.7

2-Ethoxy-4-formyl-3-nitrophenyl acetate (21)

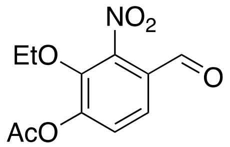

2-Ethoxy-4-formylphenyl acetate (**20**, 8.50 g, 40.8 mmol, 1 equiv.) was added to a cold solution of fuming HNO_3 (53.4 mL, 1.28 mol, 25 equiv.) using an ice bath. The reaction mixture was stirred for

3 h, keeping the temperature under 0 °C. The cooling bath was removed, the reaction was poured into ice and the mixture was stirred at rt until the precipitation of a yellow solid was complete. The solution was poured in ice-water and the precipitate was filtered, washed with water and dried to afford the desired product (9.21 g, 36.4 mmol, **89%**) as a light-yellow solid. **R**$_f$ = 0.36 (25% EtOAc in cyclohexane); **m.p.:** 66 – 68 °C; **^{1}H NMR** (400 MHz, Acetone-*d*$_6$) δ 9.97 (s, 1H), 7.91 (d, *J* = 8.5 Hz, 1H), 7.68 (d, *J* = 8.5 Hz, 1H), 4.20 (q, *J* = 7.0 Hz, 2H), 1.32 (t, *J* = 7.0 Hz, 3H); **^{13}C NMR** (101 MHz, Acetone-*d*$_6$) δ 188.2, 168.6, 150.2, 145.1, 144.5, 129.1, 127.4, 126.5, 72.8, 20.8, 15.8

3-Ethoxy-4-hydroxy-2-nitrobenzaldehyde (22)

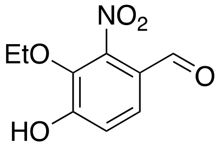

2-Ethoxy-4-formyl-3-nitrophenyl acetate (**21**, 8.00 g, 31.6 mmol, 1 equiv.) was hydrolysed by a solution of KOH (130 mL, 2M) at 100 °C for 10 min. After cooling, the reaction mixture was acidified with concentrated HCl. The precipitate was filtered and washed with water and recrystallized in toluene to afford the desired product as a light yellow solid (4.25 g, 20.1 mmol, **64%**). **R**$_f$ = 0.13 (25% EtOAc in cyclohexane); **m.p.:** 130 – 132 °C; **^{1}H NMR** (400 MHz, Acetone-*d*$_6$) δ 10.24 (br s, 1H), 9.79 (s, 1H), 7.69 (d, *J* = 8.5 Hz, 1H), 7.28 (d, *J* = 8.5 Hz, 1H), 4.22 (q, *J* = 7.0 Hz, 2H), 1.29 (t, *J* = 7.0 Hz, 3H); **^{13}C NMR** (101 MHz, Acetone-*d*$_6$) δ 187.4, 157.8, 145.6, 139.9, 130.4, 120.8, 118.7, 71.2, 15.5; **HRMS** (pos. APCI): calcd. for $C_9H_9NO_5Na$ $[M+Na]^+$: 234.03784, found: 234.0373

2-Amino-3-ethoxy-4-hydroxybenzaldehyde (23)

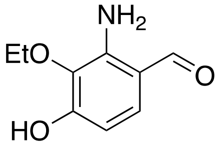

To a solution of 3-ethoxy-4-hydroxy-2-nitrobenzaldehyde (**22**, 4.14 g, 19.6 mmol, 1 equiv.) in a mixture of EtOH (60 mL) and water (20 mL) were added $FeSO_4$. 7 H_2O (981 mg, 3.53 mmol, 0.18 equiv.) and iron powder (9.85 g, 176 mmol, 9 equiv.). The mixture was stirred at reflux for 48 h. The solution was filtered, evaporated and the residue was purified on silica gel eluting with 25-50% EtOAc in cyclohexane to afford the desired product as a yellow powder (3.06 g, 16.9 mmol, **86%**). **R**$_f$ = 0.57 (50% EtOAc in cyclohexane); **m.p.**: 89 – 91 °C; **^{1}H NMR** (400 MHz, Acetone-*d*$_6$) δ 9.69 (s, 1H), 8.71 (br s, 1H), 7.19 (d, *J* = 8.6 Hz, 1H), 6.64 (br s, 2H), 6.32 (d, *J* = 8.6 Hz, 1H), 4.01 (q, *J* = 6.9 Hz, 2H), 1.35 (t, *J* = 6.9 Hz, 3H); **^{13}C NMR** (101 MHz, Acetone-*d*$_6$) δ 192.6, 155.9, 146.7, 133.2, 132.5, 115.0, 106.5, 68.2, 15.8; **HRMS** (pos. ESI): calcd. for $C_9H_{12}NO_3$ $[M+H]^-$: 182.081719, found: 182.0812

3-Ethoxy-4-hydroxy-2-iodobenzaldehyde (24)

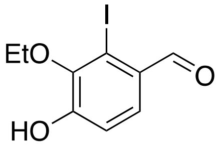

To a solution of 2-amino-3-ethoxy-4-hydroxybenzaldehyde (**23**, 3.05 g, 16.8 mmol, 1 equiv.) in a solution of HCl 20% (100 mL) at 0 °C was slowly added a solution of $NaNO_2$ (1.27 g, 18.4 mmol, 1.09 equiv.) in water (12 mL). The stirring was continued for 15 min. A solution of KI (13.9 g, 84.2 mmol, 5 equiv.) in water (45 mL) was added slowly. The solution was allowed to warm at rt and stirring was continued for 16 h. AcOEt and $Na_2S_2O_3$ were added and the organic layer was separated, washed with brine, then dried over Na_2SO_4. Removal of the solvent under vacuum followed by purification on silica gel column eluting with 10-50% EtOAc in cyclohexane afforded the desired product (3.40 g, 11.7 mmol, **69%**) as a yellow solid. **R*f*** = 0.51 (25% EtOAc in cyclohexane); **m.p.:** 115 – 117 °C; **^{1}H NMR** (400 MHz, Acetone-d_6) δ 9.95 (d, *J* = 0.8 Hz, 1H), 7.56 (d, *J* = 8.5 Hz, 1H), 7.05 (dd, *J* = 8.4, 0.8 Hz, 1H), 4.10 (q, *J* = 7.0 Hz, 2H), 1.45 (t, *J* = 7.0 Hz, 3H);); **^{13}C NMR** (101 MHz, Acetone-d_6) δ 194.7, 156.9, 147.9, 129.8, 128.1, 117.8, 101.2, 69.8, 15.8; **HRMS** (neg. APCI): calcd. for $C_9H_8IO_3$ [M-H]$^-$: 290.95182, found: 290.9524

2,3-Diethoxy-4-hydroxybenzaldehyde (25)

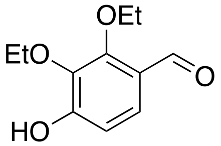

The synthesis was adapted from the following procedure (1). To a solution of 3-ethoxy-4-hydroxy-2-iodobenzaldehyde (**24**, 200 mg, 0.685 mmol, 1 equiv.) in DMF (5 mL) was added a solution of sodium ethoxide in EtOH (189 mg of sodium in 7 mL of EtOH). $CuCl_2$ (46 mg, 0.34 mmol, 0.5 equiv.) is added. The mixture is heated at 160 °C for 3 h. The pH was adjusted at 4 with a solution of concentrated aqueous HCl. The solvent was then removed under reduced pressure and the residue was poured into water. The resulting layers were separated, and the aqueous phase was extracted three times with EtOAc. The combined organic layers were dried over Na_2SO_4, filtered and concentrated under reduced pressure. Purification on silica gel column eluting with 10-40% EtOAc in cyclohexane afforded the desired product (122 mg, 0.58 mmol, **85%**) as a yellow solid. **R*f*** = 0.42 (25% EtOAc in cyclohexane); **m.p.:** 53 – 55 °C; **^{1}H NMR** (400 MHz, $CDCl_3$) δ 10.18 (d, *J* = 0.8 Hz, 1H), 7.52 (d, *J* = 8.5 Hz, 1H), 6.76 (dd, *J* = 8.7, 0.8 Hz, 1H), 6.58 (br s, 1H), 4.19 (q, *J* = 7.0 Hz, 2H), 4.17 (q, *J* = 7.0 Hz, 2H), 1.38 (t, *J* = 7.0 Hz, 3H), 1.36 (t, *J* = 7.0 Hz, 3H); **^{13}C NMR** (101 MHz, $CDCl_3$) δ 189.3, 156.9, 155.9, 138.6, 125.1, 124.1, 111.8, 71.0, 69.9; 16.1, 15.9; **HRMS** (neg. APCI): calcd. for $C_{11}H_{13}O_4$ [M-H]$^-$: 209.08138, found: 209.0819

4-(Benzyloxy)-2,3-diethoxybenzaldehyde (6a)

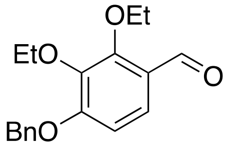

To a solution of 2,3-diethoxy-4-hydroxybenzaldehyde (**25**, 2.47 g, 11.7 mmol, 1 equiv.) in DMF (10 mL) were added K_2CO_3 (1.95 g, 14.1 mmol, 1.2 equiv.) and benzyl bromide (1.68 mL, 14.1 mmol, 1.2 equiv.). The mixture was heated at 90 °C overnight. The reaction mixture was allowed to cool to rt and was poured into water. The resulting layers were separated, and the aqueous phase was extracted three times with EtOAc. The combined organic layers were dried over Na_2SO_4, filtered and concentrated under reduced pressure. Purification on silica gel column eluting with 10-20% EtOAc in cyclohexane afforded the desired compound (2.94 g, 9.79 mmol, **83%**) as a light-yellow

oil. **R**$_f$ = 0.62 (20% EtOAc in cyclohexane); **^{1}H NMR** (400 MHz, $CDCl_3$) δ 10.26 (d, *J* = 0.6 Hz, 1H), 7.55 (d, *J* = 8.8 Hz, 1H), 7.44 – 7.29 (m, 5H), 6.78 (dd, *J* = 8.8, 0.6 Hz, 1H), 5.16 (s, 2H), 4.26 (q, *J* = 7.0 Hz, 2H), 4.09 (q, *J* = 7.0 Hz, 2H), 1.39 (t, *J* = 7.0 Hz, 3H), 1.36 (t, *J* = 7.0 Hz, 3H); **^{13}C NMR** (101 MHz, $CDCl_3$) δ 189.3, 158.9, 156.8, 141.5, 136.4, 128.9, 128.4, 127.4, 124.3, 123.9, 109.2, 71.1, 70.9, 69.5, 15.9, 15.8; **HRMS** (pos. ESI): calcd. for $C_{18}H_{21}O_4$ $[M+H]^+$: 301.1440, found: 301.1435

4-(Benzyloxy)-2,3-diethoxy-6-nitrobenzaldehyde (6aa)

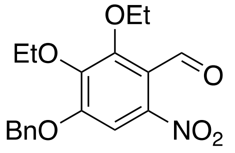

To a solution of 4-(benzyloxy)-2,3-diethoxybenzaldehyde (**6a**, 2.90 g, 9.66 mmol, 1 equiv.) in AcOH (23 mL) was added dropwise a solution of AcOH/HNO_3 (1:2 v/v, 23 mL) at 0 °C. The solution was stirred at 0 °C for 90 min and then poured into ice water (150 mL) and extracted three times with DCM. The combined organic layers were washed with saturated aqueous $NaHCO_3$ and brine, dried over Na_2SO_4, and concentrated *in vacuo*. Silica gel column eluting with 15% EtOAc in cyclohexane provided the desired compound (2.36 g, 6.83 mmol, **71%**) as a yellow solid. **R**$_f$ = 0.34 (10% EtOAc in cyclohexane); **m.p.:** 94 – 96 °C; **^{1}H NMR** (400 MHz, $CDCl_3$) δ 10.23 (s, 1H), 7.54 – 7.33 (m, 5H), 7.33 (s, 1H), 5.17 (s, 2H), 4.19 (q, *J* = 7.3 Hz, 1H), 4.17 (q, *J* = 7.3 Hz, 2H), 1.36 (t, *J* = 7.3 Hz, 3H), 1.36 (t, *J* = 7.3 Hz, 3H); **^{13}C NMR** (101 MHz, $CDCl_3$) δ 187.3, 155.1, 153.2, 146.7, 143.4, 135.3, 129.1, 128.9, 127.7, 121.7, 105.3, 71.8, 71.7, 70.1, 15.9, 15.6; **HRMS** (pos. ESI): calcd. for $C_{18}H_{19}O_6NNa$ $[M+Na]^+$: 368.1110, found: 368.1105

(4-(Benzyloxy)-2,3-diethoxy-6-nitrophenyl)methanol (7aa)

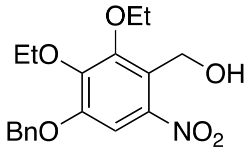

To a solution of 4-(benzyloxy)-2,3-diethoxy-6-nitrobenzaldehyde (**6aa**, 2.34 g, 6.78 mmol, 1 equiv.) was dissolved in a solution of MeOH (65 mL). $NaBH_4$ (385 mg, 10.2 mmol, 1.5 equiv.) was added portionwise at 0 °C and the mixture was stirred at 0 °C for 30 min. The solution was then allowed to return at rt, evaporated. Water was added to the residue and the aqueous phase was extracted three times with EtOAc. The combined organic layers were dried over Na_2SO_4, filtered and concentrated under reduced pressure to afford the desired compound (2.35 g, 6.78 mmol, **100%**) as a colorless oil. **R**$_f$ = 0.48 (20% EtOAc in cyclohexane); **m.p.:** 95 – 97 °C; **^{1}H NMR** (400 MHz, $CDCl_3$) δ 7.44 – 7.30 (m, 6H), 5.13 (s, 2H), 4.78 (br s, 2H), 4.18 (q, *J* = 7.0 Hz, 2H), 4.16 (q, *J* = 7.0 Hz, 2H), 2.89 (br s, 1H), 1.41 (t, *J* = 7.0 Hz, 3H), 1.36 (t, *J* = 7.0 Hz, 3H); **^{13}C NMR** (101 MHz, $CDCl_3$) δ 152.6, 152.2, 147.5, 144.7, 135.8, 128.9, 128.6, 127.6, 124.8, 106.4, 71.5, 71.1, 69.9, 56.0, 15.9, 15.8; **HRMS** (pos. APCI): calcd. for $C_{18}H_{21}NO_6Na$ $[M+Na]^+$: 370.1267, found: 370.1261

1-(Benzyloxy)-4-(chloromethyl)-2,3-diethoxy-5-nitrobenzene (8aa)

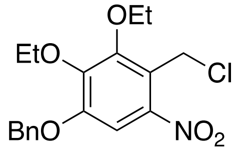

To a solution of (4-(benzyloxy)-2,3-diethoxy-6-nitrophenyl)methanol (**7aa**, 2.25 g, 6.48 mmol, 1 equiv.) in anhydrous DCM (20 mL) at 0 °C was added dropwise $SOCl_2$ (1.08 mL, 13.0 mmol, 2 equiv.). The solution was stirred for 1 h at 0 °C. The solution was then evaporated, DCM and water were added. The aqueous phase extracted three times with DCM. The combined organic phase was washed with brine, dried over Na_2SO_4 and evaporated to afford the desired compound (2.37 g, 6.48 mmol, **100%**) as a yellow solid. **R*f*** = 0.50 (10% EtOAc in cyclohexane); **m.p.:** 100 – 102 °C; **^{1}H NMR** (400 MHz, $CDCl_3$) δ 7.48 (s, 1H), 7.44 – 7.31 (m, 5H), 5.14 (s, 2H), 4.96 (s, 2H), 4.22 (q, *J* = 7.0 Hz, 2H), 4.17 (q, *J* = 7.0 Hz, 1H), 1.44 (t, *J* = 7.0 Hz, 3H), 1.36 (t, *J* = 7.0 Hz, 3H); **^{13}C NMR** (101 MHz, $CDCl_3$) δ 152.7, 152.5, 146.8, 143.8, 135.7, 129.0, 128.7, 127.7, 121.3, 106.7, 71.5, 70.8, 69.8, 36.3, 15.9, 15.9; **HRMS** (pos. ESI): calcd. for $C_{18}H_{20}ClNO_5Na$ $[M+Na]^+$: 388.0928, found: 388.0922

2-(4-(Benzyloxy)-2,3-diethoxy-6-nitrophenyl)acetonitrile (9aa)

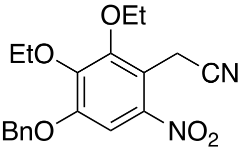

The compound was synthetized following the same procedure used for 2-(4-(benzyloxy)-2-iodo-3-methoxyphenyl)acetonitrile. The residue was purified on silica gel column eluting with 10-40% EtOAc in cyclohexane to afford the desired product as a light-yellow oil (1.11 g, 3.56 mmol, **50%**). **R*f*** = 0.49 (20% EtOAc in cyclohexane); **m.p.:** 76 – 78 °C; **^{1}H NMR** (400 MHz, $CDCl_3$) δ 7.58 (s, 1H), 7.45 – 7.32 (m, 5H), 5.15 (s, 2H), 4.24 (q, *J* = 7.1 Hz, 2H), 4.18 (q, *J* = 7.0 Hz, 2H), 4.03 (s, 2H), 1.44 (t, *J* = 7.1 Hz, 3H), 1.36 (t, *J* = 7.0 Hz, 3H); **^{13}C NMR** (101 MHz, $CDCl_3$) δ 152.5, 152.0, 147.2, 143.3, 135.6, 129.0, 128.7, 127.7, 117.2, 114.6, 106.7, 71.6, 70.6, 70.0, 15.9, 15.5; **HRMS** (pos. ESI): calcd. for $C_{19}H_{20}N_2O_5Na$ $[M+Na]^+$: 379.1270, found: 379.1266

1-(4-(Benzyloxy)-2,3-diethoxy-6-nitrophenyl)cyclopentane-1-carbonitrile (11a)

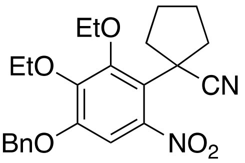

To a solution of 2-(4-(benzyloxy)-2,3-diethoxy-6-nitrophenyl)acetonitrile (**9aa**, 220 mg, 0.62 mmol, 1 equiv.) in dry DMF (2 mL) were added $CsCO_3$ (1.01 g, 1.85 mmol, 3 equiv.) and 1,4-dibromobutane (81 µL, 0.68 mmol, 1.1 equiv.) at rt. The mixture was stirred for 2 days at rt and evaporated. Water was added to the residue and the aqueous phase was extracted three times with EtOAc. The combined organic phase was washed with brine, dried over Na_2SO_4 and evaporated. Purification on silica gel eluting with 10-30% EtOAc in cyclohexane afford the desired product (151 mg, 0.37 mmol, **60%**) as a light yellow solid. **R*f*** = 0.46 (20% EtOAc in cyclohexane); **m.p.:** 90 – 92 °C; **^{1}H NMR** (400 MHz, $CDCl_3$) δ 7.42 – 7.30 (m, 5H), 6.81 (s, 1H), 5.07 (s, 2H), 4.31 (q, *J* = 7.1 Hz, 2H), 4.11 (q, *J* = 7.1 Hz, 2H), 2.87-2.77 (m, 2H), 1.97 – 1.72 (m, 6H), 1.48 (t, *J* = 7.1 Hz, 3H), 1.35 (t, *J* = 7.1 Hz, 1H); **^{13}C NMR** (101 MHz, $CDCl_3$) δ 153.8, 152.5, 146.3, 144.9, 135.7, 128.9, 128.6, 127.5, 123.4, 119.9,

104.9, 71.5, 70.1, 69.7, 43.6, 38.6, 23.6, 15.9, 15.8; **HRMS** (pos. ESI): calcd. for $C_{23}H_{26}N_2O_5Na$ [M+Na]$^+$: 433.1739, found: 433.1734

1-(2,3-Diethoxy-4-hydroxy-6-nitrophenyl)cyclopentane-1-carbonitrile (12a)

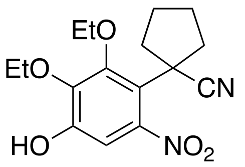

The compound was synthetized following general procedure for benzyl deprotection starting from 1-(4-(benzyloxy)-2,3-diethoxy-6-nitrophenyl)cyclopentane-1-carbonitrile (**11a**) in DCM (12.7 mL). The residue was purified on silica gel eluting with 10-30% EtOAc in cyclohexane to afford a brown oil (169 mg, 0.53 mmol, **83%**). $\mathbf{R}_f$ = 0.49 (30% EtOAc in cyclohexane); **^{1}H NMR** (400 MHz, $CDCl_3$) δ 6.81 (s, 1H), 6.16 (br s, 1H), 4.23 (q, *J* = 7.0 Hz, 2H), 4.14 (q, *J* = 7.0 Hz, 2H), 2.86 – 2.75 (m, 2H), 1.94 – 1.73 (m, 6H), 1.47 (t, *J* = 7.0 Hz, 3H), 1.37 (t, *J* = 7.0 Hz, 3H); **^{13}C NMR** (101 MHz, $CDCl_3$) δ 152.5, 150.1, 146.9, 141.7, 123.3, 118.8, 106.5, 69.5, 43.4, 38.6, 23.5, 15.8, 15.7; **HRMS** (pos. ESI): calcd. for $C_{16}H_{20}N_2O_5Na$ [M+Na]$^+$: 343.1270, found: 343.1264

1-(2,3-Diethoxy-6-nitro-4-(3-(pyrrolidin-1-yl)propoxy)phenyl)cyclopentane-1-carbonitrile (13a)

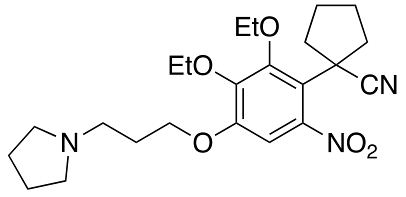

To a solution of 1-(2,3-diethoxy-4-hydroxy-6-nitrophenyl)cyclopentane-1-carbonitrile (**12a**, 169 mg, 0.53 mmol, 1 equiv) in DMF (3.4 mL) were added 1-(3-chloropropyl)pyrrolidine (**19**, 93 mg, 0.63 mmol, 1.2 equiv), K_2CO_3 (102 mg, 0.74 mmol, 1.4 equiv) and NaI (16 mg, 0.11 mmol, 0.2 equiv). The mixture was heated at 80 °C for 1 h. Water was added, and the aqueous phase was extracted three times with EtOAc. The combined organic phase was washed with brine, dried over Na_2SO_4 and concentrated under reduced pressure. The residue was purified on silica gel column eluting with 0-10% MeOH in DCM to afford the desired product (150 mg, 0.35 mmol, **66%**) as a light yellow oil. $\mathbf{R}_f$ = 0.38 (6% MeOH in DCM); **^{1}H NMR** (400 MHz, $CDCl_3$) δ 6.76 (s, 1H), 4.27 (q, *J* = 7.1 Hz, 2H), 4.06 (q, *J* = 7.1 Hz, 2H), 4.03 (t, *J* = 6.1 Hz, 2H), 2.84-2.76 (m, 2H), 2.69 – 2.64 (m, 2H), 2.61 – 2.53 (m, 4H), 2.09 – 2.00 (m, 2H), 1.93 – 1.71 (m, 10H), 1.45 (t, *J* = 7.1 Hz, 3H), 1.35 (t, *J* = 7.1 Hz, 3H); **^{13}C NMR** (101 MHz, $CDCl_3$) δ 153.6, 152.8, 146.4, 144.5, 123.4, 119.4, 104.3, 70.1, 69.6, 67.8, 54.2, 52.8, 43.6, 38.6, 28.4, 23.6, 23.6, 15.9, 15.8; **HRMS** (pos. ESI): calcd. for $C_{16}H_{20}N_2O_5Na$ [M+Na]$^+$: 432.2498, found: 432.2493

4',5'-diethoxy-6'-(3-(pyrrolidin-1-yl)propoxy)spiro[cyclopentane-1,3'-indol]-2'-amine (1a)

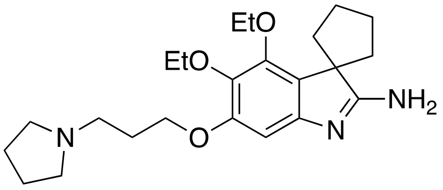

To a solution of 1-(2,3-diethoxy-6-nitro-4-(3-(pyrrolidin-1-yl)propoxy)phenyl)cyclopentane-1-carbonitrile (**13a,** 120 mg, 0.28 mmol, 1 equiv.) in AcOH (1.91 mL) was added zinc dust (273 mg, 4.17 mmol, 15 equiv.). The reaction mixture was heated up to 80 °C and was stirred at that temperature for 1.5 h. The solids were filtered and washed with AcOH. The filtrate was concentrated under reduced pressure. The residue was dissolved using EtOAc and an aqueous

solution of NaOH (10 mL, 2M). The layers were separated, and the aqueous layer was extracted three times with EtOAc. The combined organic layers were dried over Na_2SO_4, filtered and concentrated under reduced pressure. The product was obtained as a light brown solid (78 mg, 0.27 mmol, **78%**). **^{1}H NMR** (400 MHz, $CDCl_3$) δ 6.55 (s, 1H), 6.10 (br s, 2H), 4.20 (q, *J* = 7.1 Hz, 2H), 3.96 (t, *J* = 6.3 Hz, 2H), 3.93 (q, *J* = 7.0 Hz, 2H), 2.63 – 2.58 (m, 2H), 2.53 – 2.45 (m, 4H), 2.23 – 1.68 (m, 14H), 1.34 (t, *J* = 7.0 Hz, 3H), 1.32 (t, *J* = 7.0 Hz, 3H); **^{13}C NMR** (101 MHz, $CDCl_3$, 60 °C) δ 182.4, 153.6, 148.7, 148.4, 137.0, 124.8, 98.1, 69.2, 68.8, 67.9, 58.6, 54.4, 53.4, 37.2, 29.3, 28.1, 23.8, 16.0, 15.8; **HRMS** (pos. ESI): calcd. for $C_{23}H_{36}N_3O_3$ $[M+H]^+$: 402.2751, found: 402.2751

3. **1-(3-((2'-Amino-5'-methoxyspiro[cyclopentane-1,3'-indol]-6'-yl)oxy)propyl)pyrrolidin-3-one (1b)**

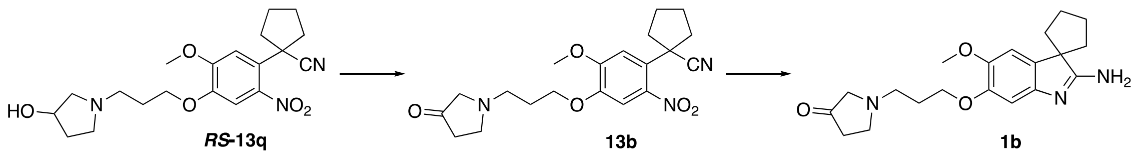

1-(4-(3-(3-Hydroxypyrrolidin-1-yl)propoxy)-5-methoxy-2-nitrophenyl)cyclopentane-1-carbonitrile (*RS*-13q)

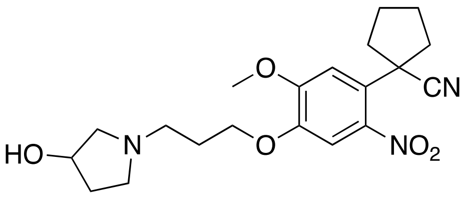

Synthesis was done according to the procedure used for (*R*)-1-(4-(3-(3-hydroxypyrrolidin-1-yl)propoxy)-5-methoxy-2-nitrophenyl)cyclopentane-1-carbonitrile (**13q**) to afford the desired product (215 mg, 0.55 mmol, **75**%) as a colorless oil. The product was directly used for the next step.

1-(5-Methoxy-2-nitro-4-(3-(3-oxopyrrolidin-1-yl)propoxy)phenyl)cyclopentane-1-carbonitrile (13b)

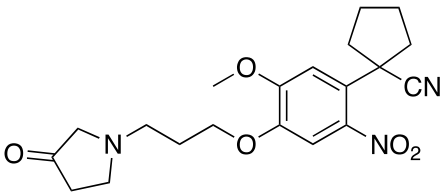

To a solution of DMSO (392 µL, 3.31 mmol, 1.1 equiv.) in dry DCM (11 mL) was added $(COCl)_2$ (142 µL, 1.66 mmol, 3 equiv.) at -78 °C dropwise. The mixture was stirred at – 78 °C for 15 min, then 1-(4-(3-(3-hydroxypyrrolidin-1-yl)propoxy)-5-methoxy-2-nitrophenyl)cyclopentane-1-carbonitrile (***RS*-13q**) was added. After 30 min, NEt_3 (0.77 mL, 5.52 mmol, 10 equiv.) was added. The mixture was stirred 30 min at -78 °C, then, was allowed to return to rt. The solution was evaporated and water was added. The aqueous phase was extracted three times with EtOAc and evaporated to afford after purification on silica gel column (20-100% EtOAc in cyclohexane), the desired product (143 mg, 0.37 mmol, **67%**) as a colorless oil. $\mathbf{R}_f$ = 0.60 (100% DCM + 2% NEt_3); **^{1}H NMR** (400 MHz, $CDCl_3$) δ 7.47 (s, 1H), 6.96 (s, 1H), 4.15 (t, *J* = 6.4 Hz, 2H), 3.94 (s, 3H), 2.97 (s, 2H), 2.93 (t, *J* = 6.9 Hz, 2H), 2.76 – 2.60 (m, 4H), 2.40 (t, *J* = 6.9 Hz, 2H), 2.12 – 1.94 (m, 6H), 1.94 – 1.81 (m, 2H); **^{13}C NMR** (101 MHz, $CDCl_3$) δ 213.8, 152.4, 148.0, 142.7, 127.2, 122.8, 110.9, 110.5, 67.5, 61.6, 56.5, 53.0, 51.7, 45.5, 39.0, 38.0, 27.5, 23.9; **HRMS** (pos. ESI): calcd. for $C_{20}H_{25}N_3O_5Na$ $[M+Na]^+$: 410.1686, found: 410.1686

1-(3-((2'-Amino-5'-methoxyspiro[cyclopentane-1,3'-indol]-6'-yl)oxy)propyl)pyrrolidin-3-one (1b)

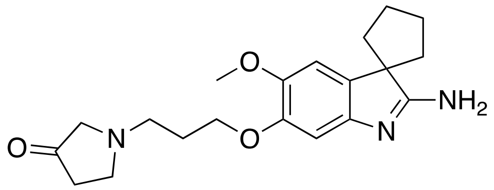

To a solution of 1-(5-methoxy-2-nitro-4-(3-(3-oxopyrrolidin-1-yl)propoxy)phenyl)cyclopentane-1-carbonitrile (**13b**, 80 mg, 0.21 mmol, 1 equiv.) in AcOH (1.4 mL) was added zinc dust (203 mg, 3.10 mmol, 15 equiv.). The reaction mixture was heated up to 80 °C and was stirred at that temperature for 1.5 h. The solids were filtered and washed with AcOH. The filtrate was concentrated under reduced pressure. The residue was dissolved in EtOAc and an aqueous solution of NaOH (10 mL, 2M). The layers were separated, and the aqueous layer was extracted three times with EtOAc. The combined organic layers were dried over Na_2SO_4, filtered and concentrated under reduced pressure. The product was obtained as a light yellow solid (64 mg, 0.18 mmol, **87%**). **^{1}H NMR** (400 MHz, MeOD-*d4*) δ 6.85 (s, 1H), 6.71 (s, 1H), 4.06 (t, *J* = 6.2 Hz, 2H), 3.79 (s, 3H), 2.96 (s, 2H), 2.81–2.73 (m, 2H), 2.11 – 1.95 (m, 10H), 1.88 – 1.78 (m, 3H); **^{13}C NMR** (101 MHz, MeOD-*d4*) δ 215.9, 182.0, 150.0, 148.4, 146.4, 136.3, 109.6, 103.5, 68.6, 60.1, 58.2, 54.6, 52.5, 39.4, 28.9, 27.2; **HRMS** (pos. ESI): calcd. for $C_{20}H_{28}N_3O_3$ $[M+H]^+$: 358.21251, found: 358.2125

4. 5'-Methoxy-6'-(4-(pyrrolidin-1-yl)butoxy)spiro[cyclohexane-1,3'-indol]-2'-amine (1c)

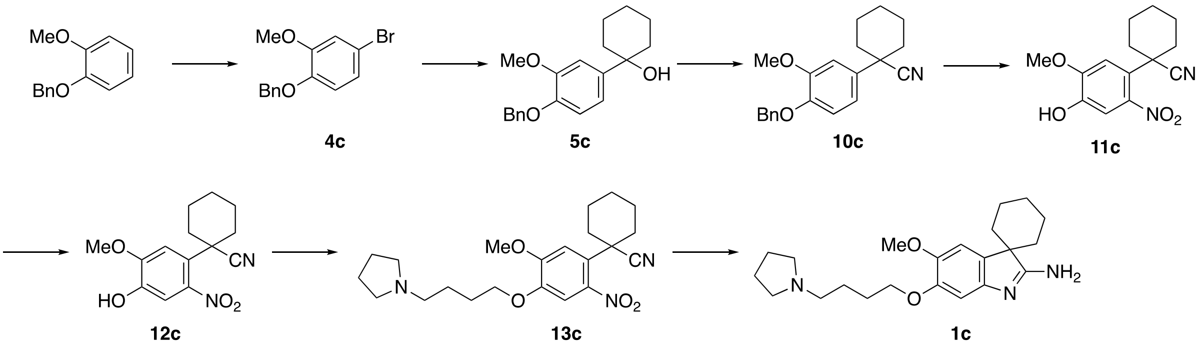

1-(Benzyloxy)-4-bromo-2-methoxybenzene (4c)

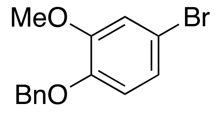

A solution of 2-methoxyphenol (5.0 g, 40 mmol, 1 equiv.) in DCM (50 mL) was cooled down to −20 °C and a solution of Br_2 (2.3 mL, 44 mmol, 1.1 equiv.) in DCM (20 mL) was added dropwise. The reaction mixture was allowed to warm up to 0 °C and was stirred at that temperature for 1 h. The reaction was quenched by addition of a saturated aqueous solution of $Na_2S_2O_3$ (10 mL). The reaction mixture was extracted three times with DCM. The combined organic layers were dried over Na_2SO_4, filtered and concentrated under reduced pressure. The residual yellow oil was dissolved in DMF (40 mL). K_2CO_3 (6.4 g, 46 mmol, 1.2 equiv.) and benzyl bromide (5.5 mL, 46 mmol, 1.2 equiv.) were added. The reaction mixture was heated up to 90 °C and was stirred at this temperature for 3 h. The reaction mixture was allowed to cool to rt and was poured into water (200 mL). The precipitate was filtered and purified by recrystallization from toluene. The product was obtained as a pink solid (11.2 g, 38 mmol, 95% over two steps). **R**$_f$ = 0.68 (33% EtOAc in cyclohexane); **^{1}H NMR** (300 MHz, $CDCl_3$) δ 7.41 – 7.29 (m, 5H), 7.00 (d, *J* = 2.3 Hz, 1H), 6.96 (dd, *J* = 8.5, 2.3 Hz, 1H), 6.74 (d, *J* = 8.5 Hz, 1H), 5.12 (s, 2H), 3.87 (s, 3H). Spectral data matched reported literature values (2).

1-(4-(Benzyloxy)-3-methoxyphenyl)cyclohexan-1-ol (5c)

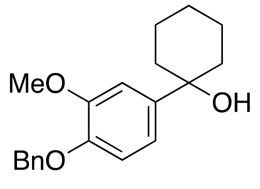

A solution of 1-(benzyloxy)-4-bromo-2-methoxybenzene (**4c**, 3.00 g, 10.2 mmol, 1 equiv.) in THF (30 mL) was cooled down to −78 °C and a solution of *n*-BuLi in hexanes (2.5 M, 4.50 mL, 11.3 mmol, 1.1 equiv.) was added dropwise. The reaction mixture was stirred at −78 °C for 20 min and a solution of cyclohexanone (1.16 mL, 11.3 mmol, 1.1 equiv.) in THF (9 mL) was added dropwise. The resulting solution was stirred at −78 °C for 5 min and was afterwards allowed to warm up to rt. The reaction was quenched by addition of a saturated aqueous solution of NH_4Cl (10 mL). The reaction mixture was extracted with DCM three times. The combined organic layers were dried over Na_2SO_4, filtered and concentrated under reduced pressure. The residue was

purified by column chromatography eluting with 20-33% EtOAc in cyclohexane. The product was obtained as a colorless solid (2.24 mg, 7.2 mmol, 70%). **R**$_f$ = 0.24 (17% EtOAc in cyclohexane); **m.p.:** 91 – 93 °C; **^{1}H NMR** (400 MHz, $CDCl_3$) δ 7.46 – 7.27 (m, 5H), 7.13 (d, *J* = 2.2 Hz, 1H), 6.94 (dd, *J* = 8.4, 2.2 Hz, 1H), 6.85 (d, *J* = 8.4 Hz, 1H), 5.14 (s, 2H), 3.91 (d, *J* = 2.5 Hz, 3H), 1.87 – 1.58 (m, 9H), 1.38 – 1.21 (m, 1H); **^{13}C NMR** (101 MHz, $CDCl_3$) δ 149.6, 147.2, 143.1, 137.5, 128.7, 127.9, 127.4, 116.8, 113.9, 109.4, 73.1, 71.3, 56.3, 39.1, 25.7, 22.4; **MS** (pos. ESI), *m*/*z* (%): 317 (100), 226 (66), 91 (19); **HRMS** (pos. ESI): calcd. for $C_{20}H_{22}O_2Na$ [M + Na^+ - H_2O]: 317.15120; found: 317.15143

1-(4-(Benzyloxy)-3-methoxyphenyl)cyclohexane-1-carbonitrile (10c)

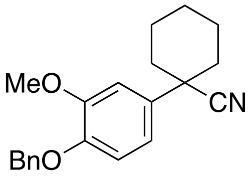

A solution of 1-(4-(benzyloxy)-3-methoxyphenyl)cyclohexan-1-ol (**5c**, 2.20 g, 7.0 mmol, 1 equiv.) in DCM (30 mL) was cooled down to −78 °C and TMSCN (2.64 mL, 21.1 mmol, 3.0 equiv.) was added dropwise. The reaction mixture was stirred at −78 °C for 5 min. Then, a solution of $SnCl_4$ in DCM (1.0 M, 7.0 mL, 7.0 mmol, 1.0 equiv.) was added dropwise and the reaction mixture was stirred at −78 °C for 5 min. The reaction mixture was poured into a saturated aqueous solution of $NaHCO_3$. The resulting emulsion was stirred at rt for 30 min and was afterwards extracted with DCM three times. The combined organic layers were dried over Na_2SO_4, filtered and concentrated under reduced pressure. The residue was purified on silica gel column eluting with 17-25% EtOAc in cyclohexane. The product was obtained as a colorless solid (1.63 g, 5.1 mmol, 72%). **R**$_f$ = 0.41 (17% EtOAc in cyclohexane); **m.p.:** 107 – 109 °C; **^{1}H NMR** (400 MHz, $CDCl_3$) δ 7.47 – 7.27 (m, 5H), 7.03 (d, *J* = 2.2 Hz, 1H), 6.94 (dd, *J* = 8.4, 2.3 Hz, 1H), 6.87 (d, *J* = 8.5 Hz, 1H), 5.15 (s, 2H), 3.92 (s, 3H), 2.20 – 2.10 (m, 2H), 1.91 – 1.66 (m, 7H), 1.34 – 1.20 (m, 1H); **^{13}C NMR** (101 MHz, $CDCl_3$) δ 150.0, 148.0, 137.2, 134.8, 128.7, 128.0, 127.4, 123.0, 117.7, 114.2, 110.2, 71.3, 56.4, 44.0, 37.7, 25.2, 23.8; **MS** (pos. ESI), *m*/*z* (%): 345 (22), 344 (100), 91 (23); **HRMS** (pos. ESI): calcd. for $C_{21}H_{23}NO_2Na$ [M + Na^+]: 344.16210; found: 344.16251

1-(4-(Benzyloxy)-5-methoxy-2-nitrophenyl)cyclohexane-1-carbonitrile (11c)

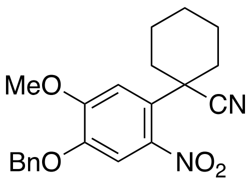

A solution of 1-(4-(benzyloxy)-3-methoxyphenyl)cyclohexane-1-carbonitrile (**10c**, 750 mg, 2.33 mmol 1 equiv.) in Ac_2O (6 mL) and AcOH (4 mL) was cooled down to 0 °C and a solution of HNO_3 (0.15 mL, 3.5 mmol, 1.5 equiv.) in AcOH (2.0 mL) was added dropwise. The reaction mixture was stirred at 0 °C for 1 h and was poured into iced water (100 mL). The resulting emulsion was allowed to warm to rt and was stirred at that temperature for 3 h. The layers were separated, and the aqueous layer was extracted three times with DCM. The combined organic layers were dried over Na_2SO_4, filtered and concentrated under reduced pressure. The residue was purified on silica gel column eluting with 17-25% EtOAc in cyclohexane. The product was

obtained as a yellow solid (769 mg, 2.10 mmol, 90%). **R$_f$** = 0.42 (25% EtOAc in cyclohexane); **m.p.:** 122 – 124 °C; **^{1}H NMR** (400 MHz, $CDCl_3$) δ 7.46 – 7.31 (m, 6H), 7.08 (s, 1H), 5.16 (s, 2H), 3.97 (s, 3H), 2.49 – 2.27 (m, 2H), 2.02 – 1.67 (m, 7H), 1.43 – 1.14 (m, 1H); **^{13}C NMR** (101 MHz, $CDCl_3$) δ 152.6, 147.5, 143.0, 135.6, 128.9, 128.6, 127.8, 127.7, 120.6, 111.5, 110.4, 71.5, 56.6, 43.3, 36.3, 24.9, 23.6; **MS** (pos. ESI), *m/z* (%): 390 (22), 389 (100); **HRMS** (pos. ESI): calcd. for $C_{21}H_{22}N_2O_4Na$ $[M + Na]^+$: 389.14718; found: 389.14713

1-(4-Hydroxy-5-methoxy-2-nitrophenyl)cyclohexane-1-carbonitrile (12c)

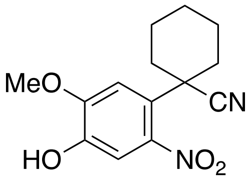

A solution of 1-(4-(benzyloxy)-5-methoxy-2-nitrophenyl)cyclohexane-1-carbonitrile (**11c**, 400 mg, 1.09 mmol, 1 equiv.) in DCM (50 mL) was cooled down to 0 °C. $AlCl_3$ (437 mg, 3.27 mmol, 3.00 equiv.) was added portionwise and the resulting suspension was stirred at 0 °C for 1 h. The reaction was quenched by addition of an aqueous solution of HCl (10 mL, 1M). The reaction mixture was allowed to warm up to rt and was extracted three times with DCM. The combined organic layers were dried over Na_2SO_4, filtered and concentrated under reduced pressure. The residue was purified on silica gel column eluting with 5% MeOH in $CHCl_3$. The product was obtained as a yellow solid (301 mg, 1.09 mmol, quant.). **R$_f$** = 0.27 (50% EtOAc in cyclohexane); **m.p.:** 173 – 175 °C; **^{1}H NMR** (400 MHz, $CDCl_3$) δ 7.34 (s, 1H), 7.07 (s, 1H), 5.84 (s, 1H), 4.00 (s, 3H), 2.44 – 2.28 (m, 2H), 1.93 – 1.80 (m, 7H), 1.36 – 1.22 (m, 1H); **^{13}C NMR** (101 MHz, $CDCl_3$) δ 149.3, 145.2, 143.8, 126.4, 120.8, 112.7, 109.5, 56.6, 43.5, 36.3, 24.9, 23.6; **MS** (pos. APCI), *m/z* (%): 309 (33), 294 (100), 277 (39), 250 (44) ; **HRMS** (pos. APCI): calcd. for $C_{14}H_{20}N_3O_4$ $[M + NH_4^+]$: 294.14483; found: 294.14474

(5-Methoxy-2-nitro-4-(4-(pyrrolidin-1-yl)butoxy)phenyl)cyclohexane-1-carbonitrile (13c)

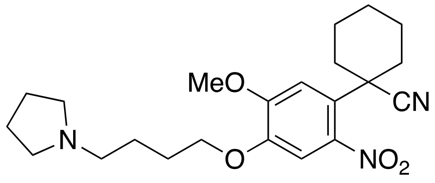

A solution of 1-(4-hydroxy-5-methoxy-2-nitrophenyl)cyclohexane-1-carbonitrile (**12c**, 36 mg, 0.13 mmol, 1 equiv.) in DMF (1.5 mL) was cooled down to 0 °C and NaH (60% in mineral oil, 7 mg, 0.17 mmol, 1.30 equiv.) was added portionwise. The reaction mixture was allowed to warm up to rt and 1,4-dichlorobutane (28 mg, 0.22 mmol, 1.69 equiv.) was added dropwise. After addition of NaI (7 mg, 0.04 mmol, 0.31 equiv.), the reaction mixture was heated up to 100 °C and was stirred at this temperature for 2 h. The reaction mixture was cooled down to 80 °C and pyrrolidine (24 µL, 0.29 mmol, 2.23 equiv.) and K_2CO_3 (24 mg, 0.17 mmol, 1.31 equiv.) were added. The reaction mixture was stirred at 80 °C for 3 h and was then allowed to cool down to rt. The reaction was quenched by addition of a saturated aqueous solution of NH_4Cl (0.5 mL) and the resulting suspension was extracted three times with DCM. The combined organic layers were dried over

Na_2SO_4, filtered and concentrated under reduced pressure. The residue was purified on silica gel column eluting with 5-10% MeOH in $CHCl_3$. The product was obtained as a yellow gum (37 mg, 0.09 mmol, 71%). **R***f* = 0.15 (5% MeOH in $CHCl_3$); **^{1}H NMR** (400 MHz, $CDCl_3$) δ 7.29 (s, 1H), 7.04 (s, 1H), 4.08 (t, *J* = 6.0 Hz, 2H), 3.94 (s, 3H), 3.27 – 3.15 (m, 4H), 3.16 – 3.06 (m, 2H), 2.44 – 2.29 (m, 2H), 2.17 – 2.02 (m, 4H), 1.99 – 1.79 (m, 9H), 1.38 – 1.18 (m, 3H); **^{13}C NMR** (101 MHz, $CDCl_3$) δ 152.5, 147.5, 143.1, 127.9, 120.6, 111.0, 110.3, 68.9, 56.6, 55.4, 53.8, 43.3, 36.3, 26.5, 24.9, 23.6, 23.5, 23.1; **MS** (pos. APCI), *m/z* (%): 403 (22), 402 (100), 376 (15); **HRMS** (pos. APCI): calcd. for $C_{22}H_{32}N_3O_4$ [M + H$^+$]: 402.23873; found: 402.23856

5'-Methoxy-6'-(4-(pyrrolidin-1-yl)butoxy)spiro[cyclohexane-1,3'-indol]-2'-amine (1c)

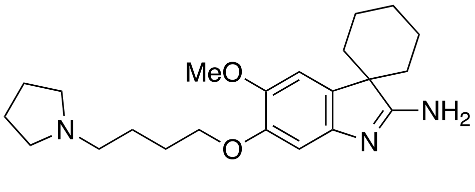

To a solution of 1-(5-methoxy-2-nitro-4-(4-(pyrrolidin-1-yl)butoxy)phenyl)cyclohexane-1-carbonitrile (**13c**, 35 mg, 0.09 mmol, 1 equiv.) in AcOH (0.30 mL, 5.2 mmol, 60.3 equiv.) was added zinc dust (86 mg, 1.31 mmol, 15.0 equiv.). The reaction mixture was heated up to 85 °C and was stirred at that temperature for 1 h. The solids were filtered and washed with AcOH. The filtrate was concentrated under reduced pressure. The residue was dissolved in EtOAc and the resulting solution was washed with a saturated aqueous solution of $NaHCO_3$ (5 mL). The layers were separated, and the aqueous layer was extracted three times with EtOAc. The combined organic layers were dried over Na_2SO_4, filtered and concentrated under reduced pressure. The product was obtained as a light yellow gum (24 mg, 0.08 mmol, 90%). **^{1}H NMR** (400 MHz, $CDCl_3$) δ 7.14 (s, 1H), 6.84 (s, 1H), 4.04 (t, *J* = 6.6 Hz, 2H), 3.86 (s, 3H), 2.52 – 2.44 (m, 6H), 1.96 – 1.82 (m, 5H), 1.81 – 1.74 (m, 6H), 1.73 – 1.65 (m, 2H), 1.63 – 1.55 (m, 4H), 1.49 – 1.38 (m, 1H). **^{13}C NMR** (101 MHz, $CDCl_3$) δ 179.0, 149.4, 149.0, 144.3, 132.4, 111.6, 103.5, 69.2, 58.3, 56.5, 54.4, 53.0, 34.1, 27.7, 25.9, 25.4, 23.6, 21.8; **MS** (pos. APCI), *m/z* (%): 373 (21), 372 (100) ; **HRMS** (pos. APCI): calcd. for $C_{22}H_{34}N_3O_2$ [M + H$^+$]: 372.26455; found: 372.26437

5. <u>5'-Methoxy-6'-(2-(pyrrolidin-1-yl)ethoxy)spiro[cyclohexane-1,3'-indol]-2'-amine (1d)</u>

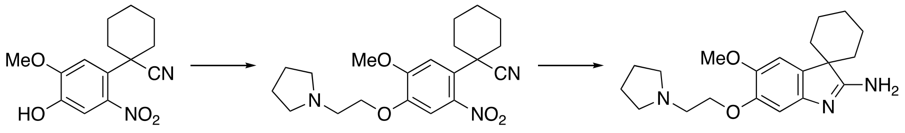

1-(5-Methoxy-2-nitro-4-(2-(pyrrolidin-1-yl)ethoxy)phenyl)cyclohexane-1-carbonitrile (13d)

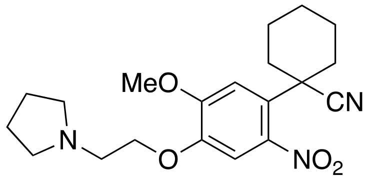

A solution of 1-(4-hydroxy-5-methoxy-2-nitrophenyl)cyclohexane-1-carbonitrile (**12c**, 70 mg, 0.25 mmol, 1 equiv.) in DMF (1.0 mL) was cooled down to 0 °C and NaH (60% in mineral oil, 12 mg, 0.30 mmol, 1.20 equiv.) was added portionwise. The reaction mixture was allowed to warm up to rt and 1-bromo-2-chloroethane (32 µL, 0.38 mmol, 1.54 equiv.) was added dropwise. The reaction mixture was heated up to 100 °C and was stirred at this temperature for 2 h. Then, pyrrolidine (42 µL, 0.50 mmol, 2.0 equiv.) and K_2CO_3 (42 mg, 0.30 mmol, 1.20 equiv.) were added. The reaction mixture was stirred at 100 °C for 2 h and was then allowed to cool down to rt. The reaction was quenched by addition of a saturated aqueous solution of NaCl (0.5 mL) and the resulting suspension was extracted five times with DCM. The combined organic layers were dried over Na_2SO_4, filtered and concentrated under reduced pressure. The residue was purified on silica gel column eluting with 5-10% MeOH in $CHCl_3$. The product was obtained as a yellow gum (38 mg, 0.10 mmol, 40%, 94% brsm over two steps). **R***f* = 0.35 (5% MeOH in $CHCl_3$); **^{1}H NMR** (400 MHz, $CDCl_3$) δ 7.36 (s, 1H), 7.06 (s, 1H), 4.23 (t, *J* = 6.0 Hz, 2H), 3.95 (s, 3H), 3.03 (t, *J* = 5.9 Hz, 2H), 2.82 – 2.65 (m, 4H), 2.44 – 2.30 (m, 2H), 1.87 (m, 13H), 1.37 – 1.22 (m, 1H); **^{13}C NMR** (101 MHz, $CDCl_3$) δ 152.5, 147.6, 143.1, 127.8, 120.7, 111.3, 110.4, 68.5, 56.6, 54.9, 54.6, 43.4, 36.3, 24.9, 23.7, 23.6; **MS** (pos. APCI), *m*/*z* (%): 375 (22), 374 (100); **HRMS** (pos. APCI): calcd. for $C_{20}H_{28}N_3O_4$: 374.20743; found: 374.20709

5'-Methoxy-6'-(2-(pyrrolidin-1-yl)ethoxy)spiro[cyclohexane-1,3'-indol]-2'-amine (1d)

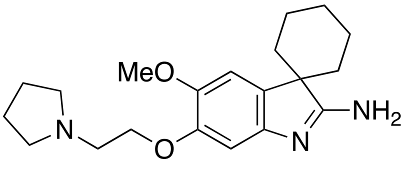

To a solution of 1-(5-methoxy-2-nitro-4-(2-(pyrrolidin-1-yl)ethoxy)phenyl)cyclohexane-1-carbonitrile (**13d**, 21 mg, 0.06 mmol, 1 equiv.) in AcOH (0.20 mL, 3.5 mmol, 62.5 equiv.) was added zinc dust (55 mg, 0.84 mmol, 15.05 equiv.). The reaction mixture was heated up to 85 °C and was stirred at that temperature for 1 h. The solids were filtered and washed with AcOH. The filtrate was concentrated under reduced pressure. The residue was dissolved in EtOAc and the resulting solution was washed with a saturated aqueous solution of $NaHCO_3$ (5 mL). The layers were separated, and the aqueous layer was extracted three times with EtOAc. The combined organic layers were dried over Na_2SO_4, filtered and concentrated under reduced pressure. The product

was obtained as a light yellow gum (19 mg, 0.06 mmol, 98%). **^{1}H NMR** (500 MHz, $CDCl_3$) δ 7.12 (s, 1H), 6.86 (s, 1H), 4.14 (t, *J* = 6.5 Hz, 2H), 3.85 (s, 3H), 2.93 (t, *J* = 6.5 Hz, 2H), 2.67 – 2.57 (m, 4H), 1.94 – 1.83 (m, 3H), 1.83 – 1.73 (m, 6H), 1.63 – 1.54 (m, 4H), 1.52 – 1.39 (m, 1H); **^{13}C NMR** (126 MHz, $CDCl_3$) δ 179.5, 149.0, 144.2, 132.0, 128.5, 111.1, 103.1, 68.1, 57.9, 55.0, 54.7, 52.8, 34.0, 25.3, 23.6, 21.7; **MS** (pos. APCI), *m*/*z* (%): 345 (20), 344 (100); **HRMS** (pos. APCI): calcd. for $C_{20}H_{30}N_3O_2$ $[M + H]^+$: 344.23325; found: 344.23322

6. 5'-Methoxy-6'-(2-(pyrrolidin-1-yl)ethoxy)spiro[cyclobutane-1,3'-indol]-2'-amine (1e)

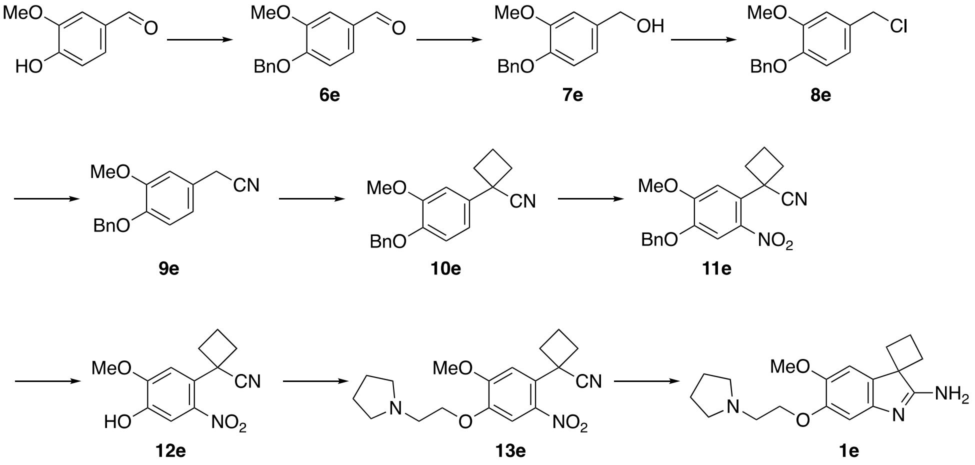

4-(Benzyloxy)-3-methoxybenzaldehyde (6e)

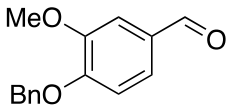

To a solution of vanillin (6.1 g, 40 mmol) in DMF (40 mL) were added K_2CO_3 (6.6 g, 48 mmol, 1.2 equiv.), and benzyl bromide (5.7 mL, 48 mmol, 1.2 equiv.). The reaction mixture was heated up to 90 °C and was stirred at this temperature for 2 h. The reaction mixture was allowed to cool to rt and was poured into water (300 mL). The resulting layers were separated, and the aqueous phase was extracted with three times EtOAc. The combined organic layers were dried over Na_2SO_4, filtered and concentrated under reduced pressure. The residue was purified by recrystallization from cyclohexane. The product was obtained as a yellow solid (8.8 g, 36 mmol, 91%). $\mathbf{R}_f$ = 0.35 (25% EtOAc in cyclohexane); **^{1}H NMR** (400 MHz, $CDCl_3$) δ 9.84 (s, 1H), 7.46 – 7.41 (m, 2H), 7.41 – 7.29 (m, 5H), 6.99 (d, *J* = 8.2 Hz, 1H), 5.24 (s, 2H), 3.95 (s, 3H). Spectral data matched reported values.

(4-(Benzyloxy)-3-methoxyphenyl)methanol (7e)

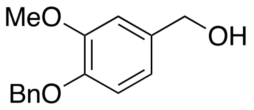

A solution of 4-(benzyloxy)-3-methoxybenzaldehyde (**6e**, 8.7 g, 36 mmol, 1 equiv.) in THF (50 mL) was cooled down to 0 °C. $LiAlH_4$ (2.1 g, 54 mmol, 1.5 equiv.) was added portionwise. The reaction mixture was allowed to warm up to rt and was stirred at this temperature for 4 h. The reaction mixture was cooled down to 0 °C and the reaction was quenched by successive addition of H_2O (2 mL), an aqueous solution of NaOH (20%, 4 mL), and H_2O (6 mL). The resulting suspension was stirred at 0 °C for 30 min and was diluted with Et_2O (70 mL). The precipitate was filtered through a plug of kieselguhr and the filter cake was washed three times with Et_2O. The filtrate was dried over Na_2SO_4, filtered and concentrated under reduced pressure to yield the product as a colorless solid (8.5 g, 35 mmol, 97%). $\mathbf{R}_f$ = 0.21 (33% EtOAc in cyclohexane); **^{1}H NMR** (300 MHz, $CDCl_3$) δ 7.47 – 7.28 (m, 5H), 6.99 – 6.78 (m, 3H), 5.16 (s, 2H), 4.61 (s, 2H), 3.91 (s, 3H), 1.56 (s, 1H). Spectral data matched reported values (3).

1-(Benzyloxy)-4-(chloromethyl)-2-methoxybenzene (8e)

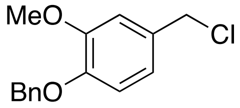

A solution of (4-(benzyloxy)-3-methoxyphenyl)methanol (**7e**, 8.4 g, 34 mmol), 1 equiv. in Et_2O (90 mL) was cooled down to 0 °C and concentrated HCl (32 mL, 380 mmol, 11 equiv.) was added dropwise. The reaction mixture was allowed to warm up to rt and was stirred at this temperature for 30 min. The reaction was quenched by addition of H_2O (50 mL) and Et_2O (50 mL). The resulting layers were separated, and the aqueous phase was extracted three times with Et_2O. The combined organic layers were dried over Na_2SO_4, filtered and concentrated under reduced pressure to yield the product as a white gum (8.4 g, 32 mmol, 93%). **R**$_f$ = 0.51 (33% EtOAc in cyclohexane); **^{1}H NMR** (400 MHz, $CDCl_3$) δ 7.48 – 7.27 (m, 5H), 6.95 – 6.83 (m, 3H,), 5.16 (s, 2H), 4.55 (s, 2H), 3.91 (s, 3H). Spectral data matched reported values (4).

2-(4-(Benzyloxy)-3-methoxyphenyl)acetonitrile (9e)

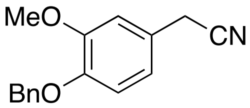

To a solution of 1-(benzyloxy)-4-(chloromethyl)-2-methoxybenzene (**8e**, 8.4 g, 32 mmol, 1 equiv.) in DMF (60 mL) were added NaI (2.4 g, 16 mmol, 0.5 equiv.) and KCN (2.3 g, 35 mmol, 1.1 equiv.). The resulting suspension was heated up to 80 °C and stirred at this temperature for 17 h. The reaction mixture was cooled down to rt and poured into H_2O (100 mL). The resulting suspension was extracted five times with EtOAc. The combined organic layers were dried over Na_2SO_4, filtered and concentrated under reduced pressure. The resulting yellow oil was purified by silica gel column eluting with 14-33% EtOAc in cyclohexane. The product was obtained as a yellow solid (6.5 g, 26 mmol, 79%). **R**$_f$ = 0.40 (25% EtOAc in cyclohexane); **^{1}H NMR** (300 MHz, $CDCl_3$) δ 7.47 – 7.27 (m, 5H), 6.92 – 6.74 (m, 3H), 5.15 (s, 2H), 3.90 (s, 3H), 3.68 (s, 2H). Spectral data matched reported values (5).

1-(4-(Benzyloxy)-3-methoxyphenyl)cyclobutane-1-carbonitrile (10e)

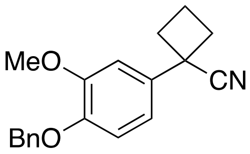

To a solution of 2-(4-(benzyloxy)-3-methoxyphenyl)acetonitrile (**9e,** 1.01 g, 4.0 mmol, 1 equiv.), 1,3-dibromopropane (0.41 mL, 4.0 mmol, 1.0 equiv.), and TBAB (13 mg, 0.04 mmol, 0.01 equiv.) in toluene (11 mL) were added KOH (85%, 1.8 g, 27 mmol, 6.8 equiv.) and H_2O (0.6 mL). The reaction mixture was heated up to reflux (approx. 120 °C) and stirred at this temperature for 1 h. The reaction mixture was cooled down to rt and was extracted with DCM three times. The combined organic layers were dried over Na_2SO_4, filtered and concentrated under reduced pressure. The residue was purified on silica gel column eluting with 11-25% EtOAc in cyclohexane. The product was obtained as a light yellow solid (0.66 g, 2.2 mmol, 56%). **R**$_f$ = 0.66 (33% EtOAc in cyclohexane); **m.p.:** 76 – 78 °C; **^{1}H NMR** (400 MHz, $CDCl_3$) δ 7.46 – 7.28 (m, 5H,), 6.94 – 6.86 (m, 3H), 5.16 (s, 2H), 3.92 (s, 3H), 2.89 – 2.72 (m, 2H), 2.64 – 2.52 (m, 2H), 2.47 – 2.31 (m, 1H), 2.12 – 1.94 (m, 1H); **^{13}C**

NMR (101 MHz, $CDCl_3$) δ 150.2, 148.0, 137.1, 133.0, 128.7, 128.1, 127.4, 124.7, 117.9, 114.3, 109.9, 71.3, 56.3, 40.1, 34.9, 17.1; **MS** (pos. APCI, $CHCl_3$), *m/z* (%): 311 (97), 294 (49), 177 (100), 149 (33), 91 (33); **HRMS** (pos. APCI): calcd. for $C_{19}H_{23}N_2O_2$ $[M + NH_4]^+$: 311.17540; found: 311.17535

1-(4-(Benzyloxy)-5-methoxy-2-nitrophenyl)cyclobutane-1-carbonitrile (11e)

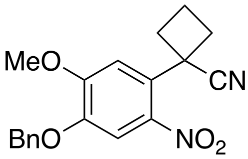

A solution of 1-(4-(benzyloxy)-3-methoxyphenyl)cyclobutane-1-carbonitrile (**10e,** 500 mg, 1.70 mmol, 1 equiv.) in Ac_2O (4 mL) and AcOH (3 mL) was cooled down to 0 °C and a solution of HNO_3 (0.11 mL, 2.6 mmol, 1.5 equiv.) in AcOH (1.4 mL) was added dropwise. The reaction mixture was stirred at 0 °C for 1 h and was poured into ice-water (100 mL). The resulting emulsion was allowed to warm to rt overnight and was extracted three times with DCM. The combined organic layers were dried over Na_2SO_4, filtered and concentrated under reduced pressure. The residue was purified on silica gel column eluting with 17-40% EtOAc in cyclohexane. The product was obtained as a yellow solid (448 mg, 1.32 mmol, 78%). $\mathbf{R}_f$ = 0.32 (33% EtOAc in cyclohexane); **m.p.:** 147 – 149 °C; **^{1}H NMR** (400 MHz, $CDCl_3$) δ 7.64 (s, 1H), 7.48 – 7.29 (m, 5H), 6.76 (s, 1H), 5.19 (s, 2H), 3.97 (s, 3H), 3.04 – 2.83 (m, 2H), 2.57 – 2.40 (m, 3H), 2.00 – 1.85 (m, 1H); **^{13}C NMR** (101 MHz, $CDCl_3$) δ 153.8, 147.7, 140.3, 135.5, 129.7, 128.9, 128.6, 127.7, 123.0, 110.8, 110.7, 71.5, 56.7, 39.3, 34.2, 16.8; **MS** (pos. APCI, $CHCl_3$), *m/z* (%): 356 (23), 339 (100), 312 (15); **HRMS** (pos. APCI): calcd. for $C_{19}H_{19}N_2O_4$ $[M + H]^+$: 339.13393; found: 339.13364

1-(4-Hydroxy-5-methoxy-2-nitrophenyl)cyclobutane-1-carbonitrile (12e)

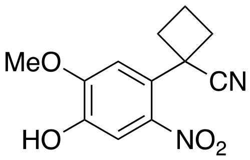

A solution of 1-(4-(benzyloxy)-5-methoxy-2-nitrophenyl)cyclobutane-1-carbonitrile (**11e,** 320 mg, 0.95 mmol, 1 equiv.) in DCM (50 mL) was cooled down to 0 °C. $AlCl_3$ (378 mg, 2.84 mmol, 3.00 equiv.) was added portionwise and the resulting suspension was stirred at 0 °C for 1 h. The reaction was quenched by addition of an aqueous solution of HCl (1M, 10 mL). The reaction mixture was allowed to warm up to room temperature and was extracted three times with DCM. The combined organic layers were dried over Na_2SO_4, filtered and concentrated under reduced pressure. The residue was purified on silica gel column eluting with 2-5% MeOH in $CHCl_3$. The product was obtained as a yellow solid (223 mg, 0.90 mmol, 95%). $\mathbf{R}_f$ = 0.34 (5% MeOH in $CHCl_3$); **m.p.:** 177 – 179 °C; **^{1}H NMR** (400 MHz, $CDCl_3$) δ 7.62 (s, 1H), 6.73 (s, 1H,), 5.84 (s, 1H), 4.01 (s, 3H), 3.02 – 2.86 (m, 2H), 2.57 – 2.39 (m, 3H), 2.00 – 1.87 (m, 1H); **^{13}C NMR** (101 MHz, $CDCl_3$) δ 150.4, 145.5, 141.3, 128.6, 123.0, 112.3, 109.9, 56.7, 39.2, 34.2, 16.8; **MS** (pos. APCI), *m/z* (%): 281 (31), 266 (100), 249 (53), 222 (32); **HRMS** (pos. APCI): calcd. for $C_{12}H_{16}N_3O_4$ $[M + NH_4]^+$: 266.11353; found: 226.11371

1-(5-Methoxy-2-nitro-4-(2-(pyrrolidin-1-yl)ethoxy)phenyl)cyclobutane-1-carbonitrile (13e)

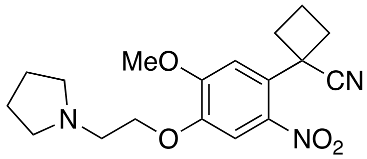

A solution of 1-(4-hydroxy-5-methoxy-2-nitrophenyl)cyclobutane-1-carbonitrile (**12e,** 100 mg, 0.40 mmol, 1 equiv.) in DMF (4.0 mL) was cooled down to 0 °C and NaH (60% in mineral oil, 19 mg, 0.48 mmol, 1.20 equiv.) was added portionwise. The reaction mixture was allowed to warm up to rt and 1-bromo-2-chloroethane (51 µL, 0.60 mmol, 1.50 equiv.) was added dropwise. The reaction mixture was heated up to 80 °C and was stirred at this temperature for 1.5 h. The reaction was quenched by addition of a saturated aqueous solution of NaCl (5 mL) and the reaction mixture was extracted with five times EtOAc. The combined organic layers were dried over Na_2SO_4, filtered and concentrated under reduced pressure. The residue was dissolved in DMF (0.5 mL) and pyrrolidine (0.19 mL, 2.3 mmol, 5.7 equiv.) was added dropwise. The reaction mixture was heated up to 70 °C and stirred at that temperature for 2 h. The reaction was quenched by addition of a saturated aqueous solution of NaCl (5 mL) and the reaction mixture was extracted with five times DCM. The combined organic layers were dried over Na_2SO_4, filtered and concentrated under reduced pressure. The residue was purified on silica gel column eluting with 2-5% MeOH in $CHCl_3$. The product was obtained as a yellow gum (42 mg, 0.12 mmol, 30%, 82% brsm over two steps). **R***f* = 0.27 (5% MeOH in $CHCl_3$); **^{1}H NMR** (400 MHz, $CDCl_3$) δ 7.62 (s, 1H), 6.73 (s, 1H), 4.26 (t, *J* = 5.9 Hz, 2H), 3.95 (s, 3H), 3.04 (t, *J* = 5.9 Hz, 2H), 3.00 – 2.89 (m, 2H), 2.81 – 2.71 (m, 4H), 2.55 – 2.41 (m, 3H), 1.99 – 1.81 (m, 5H); **^{13}C NMR** (101 MHz, $CDCl_3$) δ 153.7, 147.9, 140.4, 129.7, 123.0, 110.7, 110.6, 68.5, 56.6, 54.9, 54.6, 39.3, 34.2, 23.7, 16.8; **MS** (pos. ESI), *m*/*z* (%): 347 (20), 346 (100), 318 (16); **HRMS** (pos. ESI): calcd. for $C_{18}H_{27}N_4O_4$ $[M + H]^+$: 346.17613; found: 346.17648

5'-Methoxy-6'-(2-(pyrrolidin-1-yl)ethoxy)spiro[cyclobutane-1,3'-indol]-2'-amine (1e)

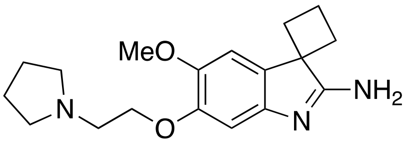

To a solution of 1-(5-methoxy-2-nitro-4-(2-(pyrrolidin-1-yl)ethoxy)phenyl)cyclobutane-1-carbonitrile (**13e,** 25 mg, 0.07 mmol, 1 equiv.) in AcOH (0.25 mL, 4.4 mmol, 62.9 equiv.) was added zinc dust (70 mg, 1.09 mmol, 15.57 equiv.). The reaction mixture was heated up to 85 °C and was stirred at that temperature for 1 h. The solids were filtered and washed with AcOH. The filtrate was concentrated under reduced pressure. The residue was dissolved in EtOAc and the resulting solution was washed with a saturated aqueous solution of $NaHCO_3$ (5 mL). The layers were separated, and the aqueous layer was extracted three times with EtOAc. The combined organic layers were dried over Na_2SO_4, filtered and concentrated under reduced pressure. The product was obtained as a light yellow gum (22 mg, 0.07 mmol, 96%). **^{1}H NMR** (400 MHz, $CDCl_3$) δ 7.04 (s, 1H), 6.81 (s, 1H), 4.08 (t, *J* = 6.4 Hz, 2H), 3.86 (s, 3H), 2.90 (t, *J* = 6.3 Hz, 2H), 2.66 – 2.55 (m, 4H), 2.53 – 2.40 (m, 4H), 2.40 – 2.26 (m, 1H), 2.23 – 2.12 (m, 1H), 1.87 – 1.70 (m, 4H); **^{13}C NMR** (101 MHz, $CDCl_3$) δ 149.2, 145.7, 107.7, 68.5, 57.6, 55.1, 54.7, 52.6, 32.2, 23.7, 16.9; **MS** (pos. APCI), *m*/*z* (%): 317

(19), 316 (100), 299 (12), 98 (16); **HRMS** (pos. APCI): calcd. for $C_{18}H_{27}N_4O_4$ [M + H]$^+$: 316.20195; found: 316.20209

7. 5'-Methoxy-6'-(3-(pyrrolidin-1-yl)propoxy)spiro[cyclopentane-1,3'-indol]-2'-amine (1f)

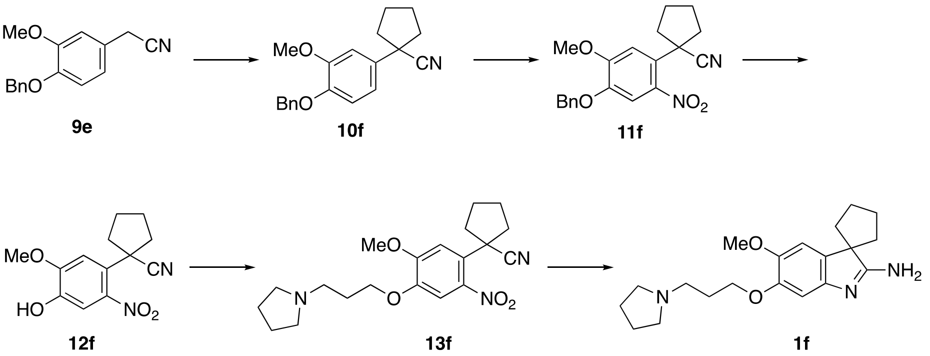

1-(4-(benzyloxy)-3-methoxyphenyl)cyclopentane-1-carbonitrile (10f)

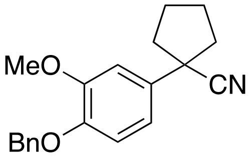

To a solution of 2-(4-(benzyloxy)-3-methoxyphenyl)acetonitrile (3.66 g, 14.4 mmol, 1 equiv., **9e**) in dry THF (50 mL) was added NaH (60% in oil, 1.50 g, 36.1 mmol, 2.5 equiv). The mixture was heated to 70 °C and a solution of 1,4-dibromobutane (2.07 mL, 17.3 mmol, 1.2 equiv.) in THF (50 mL) was added dropwise. The mixture was allowed to stir at 70 °C for 16 h. The solution was then evaporated under reduced pressure. Water was added to the residue and the aqueous phase was extracted three times with EtOAc. The combined organic phase was washed with water, brine, dried over Na_2SO_4 and evaporated. The residue was purified on silica gel column eluting with 10-30% EtOAc in cyclohexane to afford the desired product as a light yellow oil (2.34 g, 7.61 mmol, **53%**). **^{1}H NMR** (400 MHz, $CDCl_3$) δ 7.44-7.24 (m, 5H), 6.97 (d, *J* = 2.2 Hz, 1H), 6.89 (dd, *J* = 8.4, 2.2 Hz, 1H), 6.84 (d, *J* = 8.4 Hz, 1H), 5.13 (s, 2H), 3.90 (s, 3H), 2.49 – 2.37 (m, 2H), 2.08 – 1.84 (m, 6H); **^{13}C NMR** (101 MHz, $CDCl_3$) δ 150.0, 148.1, 137.2, 133.1, 128.8, 128.1, 127.5, 124.8, 118.3, 114.3, 110.6, 71.4, 56.4, 47.6, 40.6, 24.4

1-(4-(benzyloxy)-5-methoxy-2-nitrophenyl)cyclopentane-1-carbonitrile (11f)

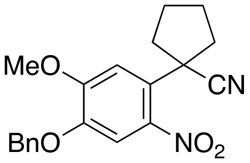

A solution of 1-(4-(benzyloxy)-3-methoxyphenyl)cyclopentane-1-carbonitrile (150 mg, 0.49 mmol, 1 equiv., **10f**) in Ac_2O (1.2 mL) and AcOH (0.9 mL) was cooled down to 0 °C and a solution of HNO_3 (31 µL, 0.73 mmol, 1.50 equiv.) in AcOH (0.4 mL) was added dropwise. The reaction mixture was stirred at 0 °C for 1 h and was poured into ice-water (10 mL). The resulting emulsion was allowed to warm up to rt. The layers were separated, and the aqueous layer was extracted three times with DCM. The combined organic layers were dried over Na_2SO_4, filtered and concentrated under reduced pressure. The residue was purified on silica gel column eluting with 10-50% EtOAc in cyclohexane. The product was obtained as a yellow oil (110 mg, 0.31 mmol, 64%). **^{1}H NMR** (400 MHz, $CDCl_3$) δ 7.53 (s, 1H), 7.48-7.18 (m, 5H), 7.02 (s, 1H), 5.20 (s, 2H), 3.99 (s, 3H), 2.79-2.65 (m, 2H), 2.14-1.85 (m, 6H); **^{13}C NMR** (100 MHz, $CDCl_3$) δ 152.9, 147.7, 135.6, 129.0, 128.7, 127.8 127.7,

123.0, 111.5, 111.3, 71.7, 56.7, 45.7, 39.2, 24.1; **HRMS** (ESI) : calcd. for $C_{20}H_{20}N_2O_4Na$ [M + Na]$^+$: 375.13208, found : 375.1315

1-(4-Hydroxy-5-methoxy-2-nitrophenyl)cyclopentane-1-carbonitrile (12f)

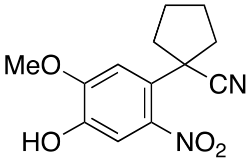

A solution of 1-(4-(benzyloxy)-5-methoxy-2-nitrophenyl)cyclopentane-1-carbonitrile (30 mg, 0.09 mmol,1 equiv.) in DCM (4 mL) was cooled down to 0 °C. $AlCl_3$ (34 mg, 0.26 mmol, 2.89 equiv.) was added portionwise and the resulting suspension was stirred at 0 °C for 1 h. The reaction was quenched by addition of an aqueous solution of HCl (2 mL, 1M). The reaction mixture was allowed to warm up to rt and was extracted three times with DCM. The combined organic layers were dried over Na_2SO_4, filtered and concentrated under reduced pressure. The residue was purified on silica gel column eluting with 2-10% MeOH in $CHCl_3$. The product was obtained as a yellow solid (21 mg, 0.08 mmol, 94%). **R*f*** = 0.24 (50% EtOAc in cyclohexane); **m.p.:** 175 – 177 °C; **^{1}H NMR** (400 MHz, $CDCl_3$) δ 7.46 (s, 1H), 6.97 (s, 1H), 5.89 (s, 1H), 4.00 (s, 3H), 2.74 – 2.61 (m, 2H), 2.12 – 1.97 (m, 4H), 1.95 – 1.82 (m, 2H); **^{13}C NMR** (101 MHz, $CDCl_3$) δ 149.5, 145.4, 143.5, 126.3, 123.0, 112.7, 110.2, 56.6, 45.6, 39.1, 24.0; **MS** (pos. ESI), *m/z* (%): 286 (15), 285 (100); **HRMS** (pos. ESI): calcd. for $C_{13}H_{14}N_2O_4Na$ [M + Na]$^+$: 285.08458; found: 285.08472.

1-(5-Methoxy-2-nitro-4-(3-(pyrrolidin-1-yl)propoxy)phenyl)cyclopentane-1-carbonitrile (13f)

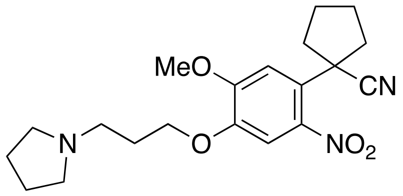

To a solution of 1-(4-hydroxy-5-methoxy-2-nitrophenyl)cyclopentane-1-carbonitrile (**12f**, 51 mg, 0.19 mmol, 1 equiv.) in DMF (3.7 mL) were added NaI (6 mg, 0.04 mmol, 0.21 equiv.), K_2CO_3 (38 mg, 0.27 mmol, 1.42 equiv.), and 1-(3-chloropropyl)pyrrolidine (**19**, 35 mg, 0.23 mmol, 1.21 equiv.). The resulting suspension was heated up to 80 °C and stirred at this temperature for 1.5 h. The reaction was quenched by addition of a saturated aqueous solution of NaCl (1.5 mL). The resulting suspension was extracted three times with DCM. The combined organic layers were dried over Na_2SO_4, filtered and concentrated under reduced pressure. The resulting orange oil was purified on silica gel column eluting with 5-10% MeOH in $CHCl_3$. The product was obtained as a yellow gum (62 mg, 0.17 mmol, 85%). **R*f*** = 0.21 (5% MeOH in $CHCl_3$); **^{1}H NMR** (400 MHz, $CDCl_3$) δ 7.46 (s, 1H), 6.96 (s, 1H), 4.16 (t, *J* = 6.2 Hz, 2H), 3.94 (s, 3H), 3.06 – 2.78 (m, 6H), 2.76 – 2.60 (m, 2H), 2.24 (dt, *J* = 13.0, 6.4 Hz, 2H), 2.09 – 1.81 (m, 10H); **^{13}C NMR** (101 MHz, $CDCl_3$) δ 152.5, 147.7, 142.7, 127.5, 122.9, 111.1, 111.0, 67.6, 56.5, 54.1, 52.8, 45.5, 39.1, 27.3, 23.9, 23.5, **MS** (pos. APCI), *m/z* (%): 375 (22), 374 (100); **HRMS** (pos. APCI): calcd. for $C_{20}H_{28}N_3O_4$ [M + H]$^+$: 374.20743; found: 374.20728

5'-Methoxy-6'-(3-(pyrrolidin-1-yl)propoxy)spiro[cyclopentane-1,3'-indol]-2'-amine (1f)

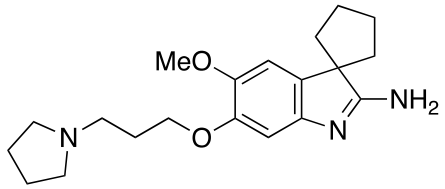

To a solution of 1-(5-methoxy-2-nitro-4-(3-(pyrrolidin-1-yl)propoxy)phenyl)cyclopentane-1-carbonitrile (**13f**, 38 mg, 0.10 mmol, 1 equiv.) in AcOH (0.40 mL, 7.0 mmol, 70.0 equiv.) was added zinc dust (66 mg, 1.02 mmol, 10.20 equiv.). The reaction mixture was heated up to 80 °C and was stirred at that temperature for 1.5 h. The solids were filtered and washed with AcOH. The filtrate was concentrated under reduced pressure. The residue was dissolved in EtOAc and the resulting solution was washed with a saturated aqueous solution of $NaHCO_3$ (5 mL). The layers were separated, and the aqueous layer was extracted three times with EtOAc. The combined organic layers were dried over Na_2SO_4, filtered and concentrated under reduced pressure. The product was obtained as a light-yellow gum (30 mg, 0.09 mmol, 86%). **^{1}H NMR** (500 MHz, $CDCl_3$) δ 6.85 (s, 1H), 6.75 (s, 1H), 4.07 (t, *J* = 6.7 Hz, 2H), 3.82 (s, 3H), 2.66 – 2.57 (m, 2H), 2.54 – 2.49 (m, 4H), 2.09 – 1.90 (m, 10H), 1.78 – 1.76 (m, 4H); **^{13}C NMR** (126 MHz, $CDCl_3$) δ 179.3, 148.9, 147.5, 145.3, 134.5, 107.9, 103.1, 68.0, 58.8, 57.8, 54.4, 53.3, 38.4, 29.0, 26.8, 23.6; **MS** (pos. ESI), *m/z* (%): 344 (62), 331 (22), 172 (100), 84 (34) ; **HRMS** (pos. ESI): calcd. for $C_{20}H_{30}N_3O_2$ $[M + H]^+$: 344.23325; found: 344.23340

8. <u>5'-Ethoxy-6'-(3-(pyrrolidin-1-yl)propoxy)spiro[cyclopentane-1,3'-indol]-2'-amine (1g)</u>

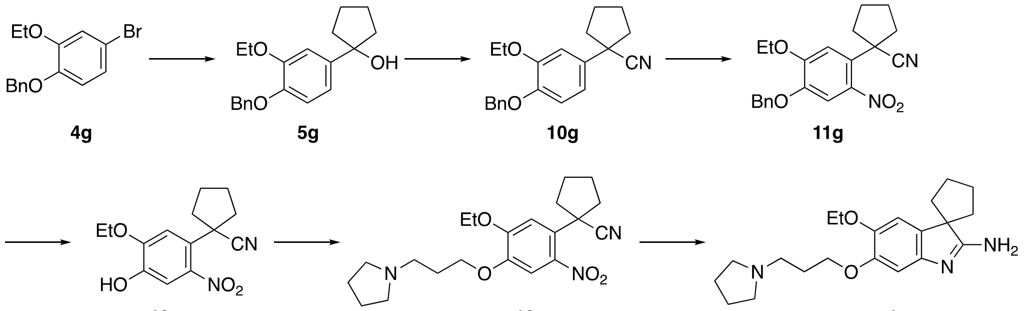

1-(4-(Benzyloxy)-3-ethoxyphenyl)cyclopentan-1-ol (5g)

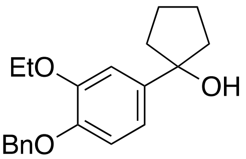

A solution of 1-(benzyloxy)-4-bromo-2-ethoxybenzene (**4g**, 1.00 g, 3.2 mmol, 1 equiv.) in THF (9 mL) was cooled down to −78 °C and a solution of *n*-BuLi in hexanes (2.5 M, 1.43 mL, 3.6 mmol, 1.1 equiv.) was added dropwise. The reaction mixture was stirred at −78 °C for 20 min and a solution of cyclopentanone (0.32 mL, 3.6 mmol, 1.1 equiv.) in THF (3 mL) was added dropwise. The resulting solution was stirred at −78 °C for 5 min and was afterwards allowed to warm up to rt. The reaction was quenched by addition of a saturated aqueous solution of NH_4Cl (10 mL). The reaction mixture was extracted three times with DCM. The combined organic layers were dried over Na_2SO_4, filtered and concentrated under reduced pressure. The residue was purified on silica gel column eluting with 17-33% EtOAc in cyclohexane. After recrystallization from cyclohexane, the product was obtained as a colorless solid (0.44 g, 1.4 mmol, 44%). **R**$_f$ = 0.22 (17% EtOAc in cyclohexane); **m.p.:** 72 – 75 °C; **^{1}H NMR** (400 MHz, Acetone-d_6) δ 7.53 – 7.27 (m, 5H), 7.18 (d, *J* = 2.1 Hz, 1H), 7.00 (dd, *J* = 8.3, 2.1 Hz, 1H), 6.94 (d, *J* = 8.3 Hz, 1H), 5.11 (s, 2H), 4.09 (q, *J* = 7.0 Hz, 2H), 3.62 (s, 1H), 1.99 – 1.87 (m, 6H), 1.82 – 1.72 (m, 2H), 1.37 (t, *J* = 7.0 Hz, 3H); **^{13}C NMR** (101 MHz, Acetone-d_6) δ 149.8, 148.2, 143.0, 139.1, 129.2, 128.4, 128.3, 118.4, 115.6, 113.2, 83.0, 71.9, 65.3, 42.8, 24.6, 15.4; **MS** (pos. ESI), *m/z* (%): 336 (21), 335 (100), 295 (32); **HRMS** (pos. ESI): calcd. for $C_{20}H_{24}O_3Na$ [M + Na]$^+$: 335.16177; found: 335.16208

1-(4-(Benzyloxy)-3-ethoxyphenyl)cyclopentane-1-carbonitrile (10g)

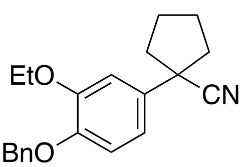

A solution of 1-(4-(benzyloxy)-3-ethoxyphenyl)cyclopentan-1-ol (**5g**, 400 mg, 1.28 mmol, 1 equiv.) in DCM (5 mL) was cooled down to −78 °C and TMSCN (480 µL, 3.84 mmol, 3.00 equiv.) was added dropwise. The reaction mixture was stirred at −78 °C for 5 min. Then, a solution of $SnCl_4$ in DCM (1.0 M, 1.28 mL, 1.28 mmol, 1.00 equiv.) was added dropwise and the reaction mixture was stirred at −78 °C for 30 min. The reaction mixture was poured into a saturated aqueous solution of $NaHCO_3$. The resulting emulsion was stirred at rtfor 30 min and was afterwards extracted three

times with DCM. The combined organic layers were dried over Na_2SO_4, filtered and concentrated under reduced pressure. The residue was purified on silica gel column eluting with 17% EtOAc in cyclohexane. The product was obtained as a yellow oil (181 mg, 0.56 mmol, 44%). **R*f*** = 0.42 (17% EtOAc in cyclohexane); **^{1}H NMR** (400 MHz, $CDCl_3$) δ 7.48 – 7.28 (m, 5H), 7.00 (d, *J* = 2.2 Hz, 1H), 6.91 (dd, *J* = 8.4, 2.2 Hz, 1H), 6.87 (d, *J* = 8.4 Hz, 1H), 5.14 (s, 2H), 4.14 (q, *J* = 7.0 Hz, 2H), 2.51 – 2.39 (m, 2H), 2.12 – 1.84 (m, 6H), 1.46 (t, *J* = 7.0 Hz, 3H); **^{13}C NMR** (101 MHz, $CDCl_3$) δ 149.5, 148.4, 137.4, 133.2, 128.6, 128.0, 127.3, 124.7, 118.4, 115.1, 112.6, 71.5, 65.2, 47.5, 40.5, 24.3, 15.1; **MS** (pos. ESI), *m*/*z* (%): 345 (21), 344 (100), 205 (15), 91 (26); **HRMS** (pos. ESI): calcd. for $C_{21}H_{23}NO_2Na$ [M + Na]$^+$: 344.16210; found: 344.16229

1-(4-(benzyloxy)-5-ethoxy-2-nitrophenyl)cyclopentane-1-carbonitrile (11g)

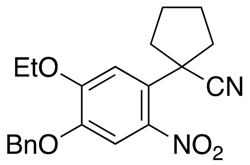

A solution of 1-(4-(benzyloxy)-3-ethoxyphenyl)cyclopentane-1-carbonitrile (**10g**, 128 mg, 0.40 mmol, 1 equiv.) in Ac_2O (1.0 mL) and AcOH (0.6 mL) was cooled down to 0 °C and a solution of HNO_3 (30 µL, 0.72 mmol, 1.80 equiv.) in AcOH (0.3 mL) was added dropwise. The reaction mixture was stirred at 0 °C for 1 h and was poured into ice-water (10 mL). The resulting emulsion was allowed to warm up to rt. The layers were separated, and the aqueous layer was extracted three times with DCM. The combined organic layers were dried over Na_2SO_4, filtered and concentrated under reduced pressure. The residue was purified on silica gel column eluting with 17% EtOAc in cyclohexane. The product was obtained as a yellow oil (121 mg, 0.33 mmol, 83%). **R*f*** = 0.45 (Silica, CH/EtOAc = 3:1); **^{1}H NMR** (500 MHz, $CDCl_3$) δ 7.51 (s, 1H), 7.45 – 7.32 (m, 5H), 6.99 (s, 1H), 5.18 (s, 2H), 4.19 (q, *J* = 6.9 Hz, 2H), 2.72 – 2.62 (m, 2H), 2.08 – 1.97 (m, 4H), 1.93 – 1.82 (m, 2H), 1.50 (t, ^3J = 7.0 Hz, 3H); **^{13}C NMR** (126 MHz, $CDCl_3$) δ 152.4, 147.8, 142.5, 135.8, 128.9, 128.5, 127.7, 127.5, 123.0, 112.5, 111.9, 71.5, 65.5, 45.5, 39.1, 23.9, 14.8; **MS** (pos. APCI), *m*/*z* (%): 385 (19), 384 (87), 368 (23), 367 (100), 340 (47), 339 (17); **HRMS** (pos. APCI): calcd. for $C_{21}H_{23}N_2O_4$ [M + H]$^+$: 367.16523; found: 367.16501

1-(5-Ethoxy-4-hydroxy-2-nitrophenyl)cyclopentane-1-carbonitrile (12g)

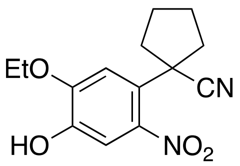

A solution of 1-(4-(benzyloxy)-5-ethoxy-2-nitrophenyl)cyclopentane-1-carbonitrile (**11g**, 100 mg, 0.27 mmol, 1 equiv.) in DCM (14 mL) was cooled down to 0 °C. $AlCl_3$ (109 mg, 0.82 mmol, 3.00 equiv.) was added portionwise and the resulting suspension was stirred at 0 °C for 1 h. The reaction was quenched by addition of an aqueous solution of HCl (5 mL, 1M). The reaction mixture was allowed to warm up to rt and was extracted three times with DCM. The combined organic layers were dried over Na_2SO_4, filtered and concentrated under reduced pressure. The

residue was purified on silica gel column eluting with 5% MeOH in $CHCl_3$. The product was obtained as a yellow oil (75 mg, 0.27 mmol, quant.). **R*f*** = 0.53 (5% MeOH in $CHCl_3$); **^{1}H NMR** (500 MHz, $CDCl_3$) δ 7.47 (s, 1H), 6.95 (s, 1H), 5.86 (s, 1H), 4.22 (q, *J* = 7.0 Hz, 2H), 2.77 – 2.61 (m, 2H), 2.08 – 1.95 (m, 4H), 1.93 – 1.80 (m, 2H), 1.51 (t, *J* = 7.0 Hz, 3H); **^{13}C NMR** (126 MHz, $CDCl_3$) δ 148.9, 145.4, 143.3, 126.3, 123.0, 112.7, 110.8, 65.5, 45.6, 39.1, 24.0, 14.8; **MS** (pos. APCI), *m/z* (%): 294 (100), 277 (77), 250 (61); **HRMS** (pos. APCI): calcd. for $C_{14}H_{17}N_2O_4$ [M + H]$^+$: 277.11828; found: 277.11807

1-(5-Ethoxy-2-nitro-4-(3-(pyrrolidin-1-yl)propoxy)phenyl)cyclopentane-1-carbonitrile (13g)

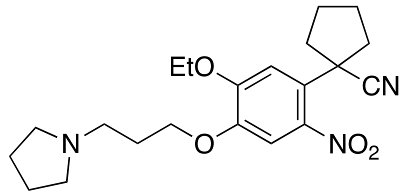

To a solution of 1-(5-ethoxy-4-hydroxy-2-nitrophenyl)cyclopentane-1-carbonitrile (**12g**, 60 mg, 0.22 mmol, 1 equiv.) in DMF (1.5 mL) were added NaI (7 mg, 0.04 mmol, 0.18 equiv.), K_2CO_3 (42 mg, 0.30 mmol, 1.36 equiv.), and 1-(3-chloropropyl)pyrrolidine (**19**, 39 mg, 0.26 mmol, 1.18 equiv.). The resulting suspension was heated up to 80 °C and stirred at this temperature for 1.5 h. The reaction was quenched by addition of a saturated aqueous solution of NaCl (1 mL). The resulting suspension was extracted three times with DCM. The combined organic layers were dried over Na_2SO_4, filtered and concentrated under reduced pressure. The resulting orange oil was purified on silica gel column eluting with 5-10% MeOH in $CHCl_3$. The product was obtained as a yellow oil (56 mg, 0.15 mmol, 67%). **R*f*** = 0.24 (10% MeOH in $CHCl_3$); **^{1}H NMR** (400 MHz, $CDCl_3$) δ 7.46 (s, 1H), 6.95 (s, 1H), 4.19 – 4.11 (m, 4H), 2.96 – 2.77 (m, 6H), 2.73 – 2.59 (m, 2H), 2.26 – 2.12 (m, 2H), 2.09 – 1.81 (m, 10H), 1.47 (t, *J* = 7.0 Hz, 3H); **^{13}C NMR** (101 MHz, $CDCl_3$) δ 152.2, 148.0, 142.6, 127.5, 122.9, 112.4, 111.5, 67.8, 65.4, 54.2, 52.9, 45.5 , 39.1, 27.5, 24.0, 23.6, 14.8; **MS** (pos. ESI), *m/z* (%): 389 (22), 388 (100); **HRMS** (pos. ESI): calcd. for $C_{21}H_{30}N_3O_4$ [M + H]$^+$: 388.22308; found: 388.22327

5'-Ethoxy-6'-(3-(pyrrolidin-1-yl)propoxy)spiro[cyclopentane-1,3'-indol]-2'-amine (1g)

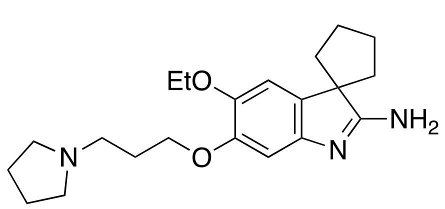

To a solution of 1-(5-ethoxy-2-nitro-4-(3-(pyrrolidin-1-yl)propoxy)phenyl)cyclopentane-1-carbonitrile (**13g**, 27 mg, 0.07 mmol, 1 equiv.) in AcOH (0.25 mL, 4.4 mmol, 62.9 equiv.) was added zinc dust (68 mg, 1.05 mmol, 15.0 equiv.). The reaction mixture was heated up to 85 °C and was stirred at that temperature for 1 h. The solids were filtered and washed with AcOH. The filtrate was concentrated under reduced pressure. The residue was dissolved in EtOAc and the resulting solution was washed with a saturated aqueous solution of $NaHCO_3$ (2 mL). The layers were separated, and the aqueous layer was extracted three times with EtOAc. The combined organic layers were dried over Na_2SO_4, filtered and concentrated under reduced pressure. After

recrystallization from benzene/pentane (1:10), the product was obtained as a light-yellow gum (15 mg, 0.04 mmol, 61%). **^{1}H NMR** (400 MHz, $CDCl_3$) δ 6.81 (s, 1H), 6.78 (s, 1H), 4.09 – 3.99 (m, 4H), 2.69 – 2.59 (m, 2H), 2.57 – 2.46 (m, 4H), 2.11 – 1.88 (m, 10H), 1.83 – 1.73 (m, 4H), 1.38 (t, *J* = 7.0 Hz, 3H); **^{13}C NMR** (101 MHz, $CDCl_3$) δ 179.5, 149.9, 148.0, 144.4, 130.1, 111.1, 103.6, 68.1, 67.1, 58.8, 54.4, 53.4, 38.3, 29.2, 26.8, 23.7, 15.5; **MS** (pos. ESI), *m/z* (%): 359 (20), 358 (100), 231 (30), 179 (96), 84 (53); **HRMS** (pos. ESI): calcd. for $C_{21}H_{32}N_3O_2$ $[M + H]^+$: 358.24890; found: 358.24915

9. **<u>2'-Amino-5'-methoxy-6'-(3-(pyrrolidin-1-yl)propoxy)spiro[cyclopentane-1,3'-indole]-4'-carbonitrile (1h)</u>**

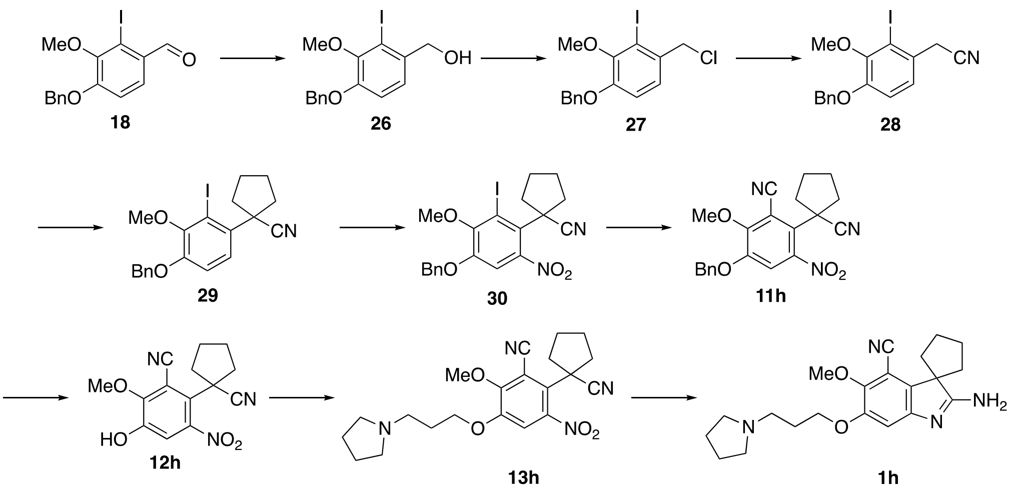

(4-(Benzyloxy)-2-iodo-3-methoxyphenyl)methanol (26)

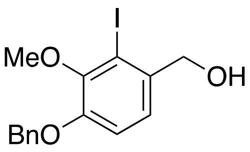

To a solution of 4-(benzyloxy)-2-iodo-3-methoxybenzaldehyde (**18,** 4.55 g, 12.3 mmol, 1 equiv.) was dissolved in a solution of MeOH (120 mL). $NaBH_4$ (700 mg, 18.5 mmol, 1.5 equiv.) was added portionwise at 0 °C and the mixture was stirred at 0 °C for 30 min. The solution was then allowed to return at rt, evaporated. Water was added to the residue and the aqueous phase was extracted three times with EtOAc. The combined organic layers were dried over Na_2SO_4, filtered and concentrated under reduced pressure to afford the desired compound (4.55 g, 12.3 mmol, 100%) as a light yellow solid. **R***f* = 0.38 (20% EtOAc in cyclohexane); **m.p.:** 79 – 80 °C; **^{1}H NMR** (400 MHz, $CDCl_3$) δ 7.44 – 7.35 (m, 5H), 7.10 (br d, *J* = 8.3 Hz, 1H), 6.92 (d, *J* = 8.3 Hz, 1H), 5.11 (s, 2H), 4.63 (br s, 2H), 3.87 (s, 3H), 1.96 (br s, 1H); **^{13}C NMR** (101 MHz, $CDCl_3$) δ 151.2, 149.6, 136.8, 136.6, 128.8, 128.3, 127.5, 124.5, 114.7, 96.9, 71.3, 69.4, 60.6; **HRMS** (pos. ESI): calcd. for $C_{15}H_{15}IO_3Na$ $[M+Na]^+$: 392.9964, found: 392.9958

1-(Benzyloxy)-4-(chloromethyl)-3-iodo-2-methoxybenzene (27)

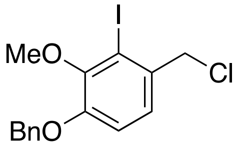

To a solution of (4-(benzyloxy)-2-iodo-3-methoxyphenyl)methanol (**26**, 4.53 g, 12.2 mmol, 1 equiv.) in Et_2O (70 mL) was cooled down to 0 °C and concentrated HCl (45.9 mL, 551 mmol, 45 equiv.) was added dropwise. The reaction mixture was allowed to warm up to rt and was stirred at this temperature for 30 min. The reaction was then allowed to warm at rt for 2 h and ether was evaporated. The aqueous phase was extracted three times with DCM. The combined organic phase

was washed with water, brine, dried over Na_2SO_4, filtered and concentrated under reduced pressure to yield the product as a yellow solid (4.67 g, 12.0 mmol, 98%). **R$_f$** = 0.63 (10% EtOAc in cyclohexane); **^{1}H NMR** (400 MHz, $CDCl_3$) δ 7.43 – 7.27 (m, 5H), 7.16 (d, *J* = 8.5 Hz, 1H), 6.90 (d, *J* = 8.5 Hz, 1H), 5.12 (s, 2H), 4.69 (s, 2H), 3.87 (s, 3H); **^{13}C NMR** (101 MHz, $CDCl_3$) δ 151.7, 150.0, 136.6, 133.5, 128.9, 128.4, 127.5, 126.0, 114.5, 99.1, 71.3, 60.6, 51.6; **HRMS** (pos. ESI): calcd. for $C_{15}H_{18}NClIO_2$ $[M+NH_4]^+$: 406.0065, found: 406.0071

2-(4-(Benzyloxy)-2-iodo-3-methoxyphenyl)acetonitrile (28)

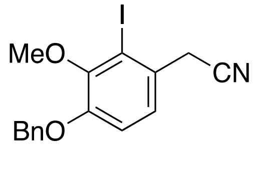

To a solution of 1-(benzyloxy)-4-(chloromethyl)-3-iodo-2-methoxybenzene (**27,** 5.61 g, 14.4 mmol, 1 equiv.) in DMF (20 mL) was added NaI (216 mg, 1.44 mmol, 0.1 equiv.) and NaCN (1.13 g, 17.3 mmol, 1.2 equiv.). The solution was heated at 80 °C for 16 h. The solution was then evaporated, water was added and the aqueous phase was extracted three times with EtOAc. The combined organic phase was washed with water, brine, dried over Na_2SO_4 and evaporated. The residue was purified on silica gel eluting with 10% EtOAc in cyclohexane to afford the desired compound (3.50 g, 9.22 mmol, 64%) as a light yellow solid. **R$_f$** = 0.29 (10% EtOAc in cyclohexane); **m.p.**: 109 – 111 °C; **^{1}H NMR** (400 MHz, $CDCl_3$) δ 7.44 – 7.31 (m, 5H), 7.19 (dt, *J* = 8.5, 0.7 Hz, 1H), 6.95 (d, *J* = 8.5 Hz, 1H), 5.13 (s, 2H), 3.88 (s, 3H), 3.79 (d, *J* = 0.7 Hz, 2H); **^{13}C NMR** (101 MHz, $CDCl_3$) δ 151.3, 151.1, 136.5, 128.9, 128.4, 127.5, 126.6, 124.5, 117.7, 114.8, 98.4, 71.3, 60.6; **HRMS** (pos. ESI): calcd. for $C_{16}H_{18}N_2IO_2$ $[M+NH_4]^+$: 397.0413, found: 397.0407

1-(4-(Benzyloxy)-2-iodo-3-methoxyphenyl)cyclopentane-1-carbonitrile (29)

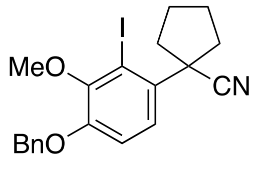

To a mixture of NaH (2.38 g, 57.2 mmol, 2.5 equiv., 60% w/w) in dry THF (90 mL), was added 2-(4-(benzyloxy)-2-iodo-3-methoxyphenyl)acetonitrile (**28**, 8.67 g, 22.9 mmol, 1 equiv.) in dry THF (90 mL). Then, 1,4-dibromopropane (3.28 mL, 27.4 mmol, 1.2 equiv.) was added. The mixture was stirred at rt for 18 h, and was evaporated. Water was added and the aqueous phase was extracted three times with EtOAc. The combined organic phase was washed with brine, dried over Na_2SO_4 and evaporated. The residue was purified on silica gel eluting with 10-30% EtOAc in cyclohexane to afford a light yellow solid (7.71 g, 17.8 mmol, **78%**). **m.p.**: 109 – 111 °C; **R$_f$** = 0.29 (10% EtOAc in cyclohexane); **^{1}H NMR** (400 MHz, $CDCl_3$) δ 7.44 – 7.29 (m, 5H), 7.02 (d, *J* = 8.6 Hz, 1H), 6.87 (d, *J* = 8.6 Hz, 1H), 5.11 (s, 2H), 3.87 (s, 3H), 2.86 – 2.74 (m, 2H), 2.19 – 2.07 (m, 2H), 2.07 – 1.92 (m, 2H), 1.92 – 1.79 (m, 2H); **^{13}C NMR** (101 MHz, $CDCl_3$) δ 151.2, 150.4, 136.6, 134.1, 128.9, 128.3, 127.4, 123.5, 123.3, 113, 9, 98.2, 71.1, 60.5, 49.5, 39.2, 24.0; **HRMS** (pos. ESI): calcd. for $C_{20}H_{20}INO_2Na$ $[M+Na]^+$: 456.0437, found: 456.0431

1-(4-(Benzyloxy)-2-iodo-3-methoxy-6-nitrophenyl)cyclopentane-1-carbonitrile (30)

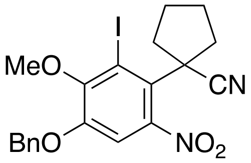

To a solution of 1-(4-(benzyloxy)-2-iodo-3-methoxyphenyl)cyclopentane-1-carbonitrile (**29**, 400 mg, 0.92 mmol, 1 equiv.) in Ac_2O (2.4 mL) and AcOH (1.8 mL) at 0 °C was added a solution of HNO_3 (0.58 mL, 13.8 mmol, 15 equiv.) in AcOH (0.77 mL). The solution was stirred for 2 h at rt. Then, the solution was poured into ice-water and the resulting solution was neutralized with Na_2CO_3, extracted with DCM. The organic phase was washed with brine, dried over Na_2SO_4 and evaporated. Purification on silica gel column eluting with 20% EtOAc in cyclohexane afforded the desired compound (384 mg, 0.80 mmol, 87%) as a light yellow solid. **R***f* = 0.70 (80% DCM in cyclohexane); **m.p.**: 180 – 182 °C**;** **^{1}H NMR** (400 MHz, $CDCl_3$) δ 7.41 – 7.31 (m, 5H), 7.07 (s, 1H), 5.10 (s, 2H), 3.88 (s, 3H), 3.19 – 3.05 (m, 2H), 2.14 – 1.75 (m, 6H); **^{13}C NMR** (101 MHz, $CDCl_3$) δ 152.1, 149.9, 148.0, 135.1, 129.1, 128.9, 127.6, 127.3, 121.8, 110.3, 100.2, 71.7, 60.7, 48.5, 39.4, 24.2; **HRMS** (pos. ESI): calcd. for $C_{20}H_{19}IN_2O_4Na$ $[M+Na]^+$: 501.0282, found: 501.0287

3-(Benzyloxy)-6-(1-cyanocyclopentyl)-2-methoxy-5-nitrobenzonitrile (11h)

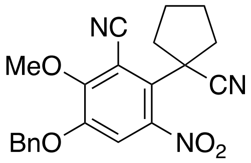

To a solution of 1-(4-(benzyloxy)-2-iodo-3-methoxy-6-nitrophenyl)cyclopentane-1-carbonitrile (**30**, 43 mg, 0.09 mmol, 1 equiv.) in dry DMF (1 mL) was added CuCN (8 mg, 0.09 mmol, 1 equiv.). The mixture was stirred at 100 °C for 6 h, then water was added and the solution was extracted with EtOAC. The organic phase was washed with brine, dried over Na_2SO_4 and evaporated. The residue was purified on silica gel column eluting with 0-40% EtOAc in cyclohexane to afford the desired product (32 mg, 0.08 mmol, 94%) as a light yellow solid. **R***f* = 0.19 (20% EtOAc in cyclohexane); **m.p.:** 156 – 158 °C; **^{1}H NMR** (400 MHz, $CDCl_3$) δ 7.44 – 7.35 (m, 5H), 7.28 (s, 1H), 5.15 (s, 2H), 4.09 (s, 3H), 2.96 – 2.87 (m, 2H), 2.20 – 1.89 (m, 6H); **^{13}C NMR** (101 MHz, $CDCl_3$) δ 156.1, 150.9, 146.8, 134.4, 129.3 (3C), 127.8 (2C), 127.7, 121.1, 113.9, 113.7, 109.0, 72.2, 62.4, 45.3, 39.7 (2C), 24.4 (2C); **HRMS** (ESI): calcd. for $C_{21}H_{19}N_3O_4Na$ $[M+Na]^+$: 400.1273, found: 400.1268.

2-(1-Cyanocyclopentyl)-5-hydroxy-6-methoxy-3-nitrobenzonitrile (12h)

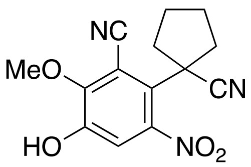

The compound was synthetized following general procedure for benzyl deprotection starting from 3-(benzyloxy)-6-(1-cyanocyclopentyl)-2-methoxy-5-nitrobenzonitrile (**11h**). The residue was purified on silica gel eluting with 20-50% EtOAc in cyclohexane to afford the desired product (270 mg, 0.94 mmol, **73%**) as a slightly brown powder. **R***f* = 0.32 (40% EtOAc in cyclohexane); **m.p.** 175 –

177 °C; **^{1}H NMR** (400 MHz, $CDCl_3$) δ 10.05 (br s, 1H), 7.46 (s, 1H), 4.11 (s, 3H), 2.92-2.82, (m, 2H), 2.24-2.13 (m, 2H), 2.01-1.88 (m, 4H); **^{13}C NMR** (100 MHz, $CDCl_3$) δ 155.3, 151.1, 147.9, 126.8, 121.9, 117.5, 115.0, 109.8, 62.4, 45.6, 39.8, 24.5; **HRMS** (ESI): calcd. for $C_{14}H_{13}N_3O_4Na$ $[M+Na]^+$: 310.0804, found: 310.0798

2-(1-Cyanocyclopentyl)-6-methoxy-3-nitro-5-(3-(pyrrolidin-1-yl)propoxy)benzonitrile (13h)

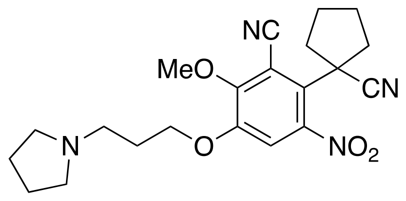

To a solution of 2-(1-cyanocyclopentyl)-5-hydroxy-6-methoxy-3-nitrobenzonitrile (**12h**, 270 mg, 0.94 mmol, 1 equiv.) in DMF (6.1 mL) were added 1-(3-chloropropyl)pyrrolidine (**19**, 167 mg, 1.13 mmol, 1.2 equiv.), K_2CO_3 (182 mg, 1.32 mmol, 1.4 equiv.) and NaI (28 mg, 0.19 mmol, 0.2 equiv.). The mixture was heated at 80 °C for 1 h. Water wad added, and the aqueous phase was extracted three times with EtOAc. The combined organic phase was washed with brine, dried over Na_2SO_4 and concentrated under reduced pressure. The residue was purified on silica gel column eluting with 0-10% MeOH in DCM to afford the desired product (243 mg, 0.61 mmol, **65%**) as a light-yellow oil. $\mathbf{R}_f$= 0.50 (10% MeOH in DCM); **m.p.:** 106 – 108 °C; **^{1}H NMR** (400 MHz, $CDCl_3$) δ 7.29 (s, 1H), 4.15 (t, *J* = 6.4 Hz, 2H), 4.08 (s, 3H), 2.94 – 2.87 (m, 2H), 2.66 (t, *J* = 7.2 Hz, 2H), 2.62 – 2.49 (m, 4H), 2.17 – 2.04 (m, 4H), 2.03 – 1.89 (m, 4H), 1.84 - 1.77 (m, 4H); **^{13}C NMR** (101 MHz, $CDCl_3$) δ 155.7, 151.4, 146.9, 127.1, 121.2, 114.0, 113.3, 108.7, 68.5, 62.3, 54.4, 52.6, 45.2, 39.6, 28.3, 24.4, 23.7; **HRMS** (ESI): calcd. for $C_{21}H_{27}N_4O_4$ $[M+H]^+$: 399.2032, found: 399.2027

2'-Amino-5'-methoxy-6'-(3-(pyrrolidin-1-yl)propoxy)spiro[cyclopentane-1,3'-indole]-4'-carbonitrile (1h)

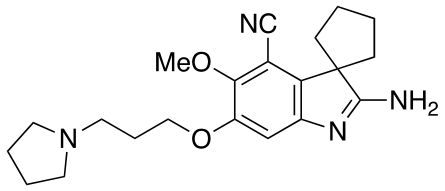

To a solution of 2-(1-cyanocyclopentyl)-6-methoxy-3-nitro-5-(3-(pyrrolidin-1-yl)propoxy)benzonitrile (**13h**, 137 mg, 0.34 mmol, 1 equiv.) in AcOH (2.36 mL) was added zinc dust (337 mg, 5.16 mmol, 15 equiv.). The reaction mixture was heated up to 80 °C and was stirred at that temperature for 1.5 h. The solids were filtered and washed with AcOH. The filtrate was concentrated under reduced pressure. The residue was dissolved in EtOAc and an aqueous solution of NaOH (10 mL, 2M). The layers were separated, and the aqueous layer was extracted three times with EtOAc. The combined organic layers were dried over Na_2SO_4, filtered and concentrated under reduced pressure. The product was obtained as a light brown solid (78 mg, 0.27 mmol, **78%**). **m.p.:** 85 – 87 °C; **^{1}H NMR** (400 MHz, $CDCl_3$) δ 7.04 (s, 1H), 6.17 (br s, 2H), 4.03 (t, *J* = 6.4 Hz, 2H), 3.91 (s, 3H), 2.63 – 2.58 (m, 2H), 2.54 – 2.47 (m, 4H), 2.33 – 2.16 (m, 4H), 2.04 – 1.84 (m, 6H), 1.79 – 1.71 (m, 4H); **^{13}C NMR** (101 MHz, $CDCl_3$) δ 183.4, 152.0, 150.1, 147.0, 135.1, 114.7,

107.0, 101.0, 68.0, 62.1, 59.0, 54.4, 53.2, 37.3, 28.8, 28.1, 23.6; **HRMS** (ESI): calcd. for $C_{21}H_{27}N_4O_4$ $[M+H]^+$: 369.2291, found: 369.2285

10. 4'-Chloro-5'-methoxy-6'-(3-(pyrrolidin-1-yl)propoxy)spiro[cyclopentane-1,3'-indol]-2'-amine (1i)

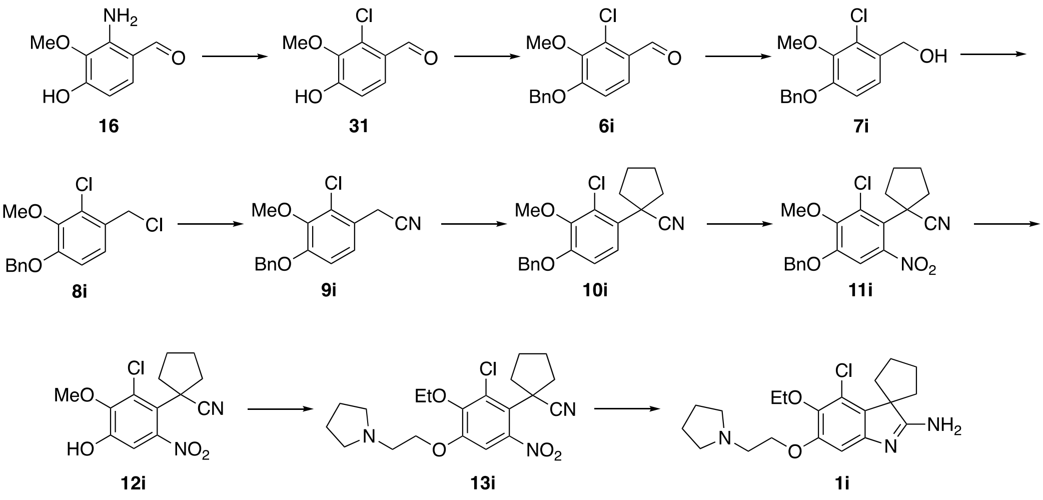

2-Chloro-4-hydroxy-3-methoxybenzaldehyde (31)

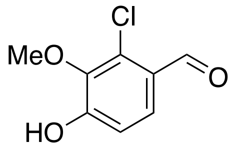

The synthesis was adapted from the literature (6). To a solution of 2-amino-4-hydroxy-3-methoxybenzaldehyde (**16**, 1.50 g, 8.97 mmol, 1 equiv.) in HCl concentrated (5 mL) and water (5 mL), was added $NaNO_2$ (674 mg, 9.78 mmo, 1.09 equiv.) portionwise at 0 °C. After 30 min, a solution of CuCl (1.04 g, 10.5 mmol, 1.17 equiv.) in HCl conc. (7.5 mL) was added slowly. The mixture was then heated to 50 °C for 1 h. The solution was then filtrated, and the residue was washed with water to afford the desired product (1.49 g, 7.96 mmol, **89%**) as a brown solid. **R_f** = 0.52 (40% EtOAc in cyclohexane); **m.p.:** 124 – 126 °C; **^{1}H NMR** (400 MHz, Acetone-d_6) δ 10.26 (d, *J* = 0.6 Hz, 1H), 9.50 (br s, 1H), 7.60 (s, *J* = 8.6 Hz, 1H), 7.02 (dd, *J* = 8.6, 0.6 Hz, 1H), 3.89 (s, 3H); **^{13}C NMR** (101 MHz, Acetone-d_6) δ 188.5, 157.9, 145.2, 132.9, 126.9, 126.8, 116.4; **HRMS** (pos. APCI): calcd. for $C_8H_6ClO_3$ [M-H]: 185.00055, found: 185.0011

4-(Benzyloxy)-2-chloro-3-methoxybenzaldehyde (6i)

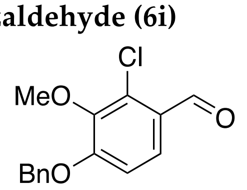

To a solution of 2-chloro-4-hydroxy-3-methoxybenzaldehyde (**31**, 1.40 g, 7.50 mmol, 1 equiv.) in DMF (10 mL) were added K_2CO_3 (1.24 g, 9.00 mmol, 1.2 equiv.) and benzyl bromide (1.07 mL, 9.00 mmol, 1.2 equiv.). The mixture was heated at 90 °C for 16 h. The reaction mixture was allowed to cool to rt and was poured into water. The resulting layers were separated and the aqueous phase was extracted three times with EtOAc. The combined organic layers were dried over Na_2SO_4, filtered and concentrated under reduced pressure. Purification on silica gel column eluting with 10-30% EtOAc in cyclohexane afforded the desired compound (1.95 g, 7.05 mmol, **94%**) as a white solid. **R_f** = 0.48 (25% EtOAc in cyclohexane); **m.p.**: 96 – 98 °C; **^{1}H NMR** (400 MHz, $CDCl_3$) δ 10.32 (d,

J = 0.8 Hz, 1H), 7.66 (d, J = 8.7 Hz, 1H), 7.43-7.30 (m, 5H), 6.95 (dd, J = 8.9, 0.8 Hz, 1H), 5.20 (s, 2H), 3.91 (s, 3H); **^{13}C NMR** (101 MHz, $CDCl_3$) δ 188.9, 157.9, 146.0, 135.8, 132.9, 129.0, 128.6, 127.5, 126.9, 125.8, 112.1, 71.2, 61.0

(4-(Benzyloxy)-2-chloro-3-methoxyphenyl)methanol (7i)

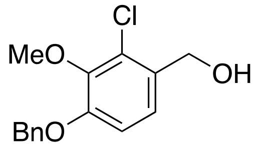

To a solution of 4-(benzyloxy)-2-chloro-3-methoxybenzaldehyde (**6i**, 1.70 g, 6.14 mmol, 1 equiv.) was dissolved in a solution of MeOH (60 mL). $NaBH_4$ (349 mg, 9.22 mmol, 1.5 equiv.) was added portionwise at 0 °C and the mixture was stirred at 0 °C for 30 min. The solution was then allowed to return at rt, evaporated. Water was added to the residue and the aqueous phase was extracted three times with EtOAc. The combined organic layers were dried over Na_2SO_4, filtered and concentrated under reduced pressure to afford the desired compound (1.62 g, 5.82 mmol, **95%**) as a colorless oil. **R_f** = 0.46 (25% EtOAc in cyclohexane); **^{1}H NMR** (300 MHz, $CDCl_3$) δ 7.45 – 7.27 (m, 5H), 7.08 (d, J = 8.6 Hz, 1H), 6.84 (d, J = 8.4 Hz, 1H), 5.11 (s, 2H), 4.67 (s, 2H), 3.89 (s, 3H); **^{13}C NMR** (101 MHz, $CDCl_3$) δ 152.4, 146.3, 136.8, 132.0, 128.8, 128.2, 127.7, 127.5, 123.9, 112.9, 71.3, 63.0, 60.9; **HRMS** (pos. APCI): calcd. for $C_{15}H_{15}ClNaO_3$ $[M+Na]^+$: 301.06074, found: 301.0602

1-(Benzyloxy)-3-chloro-4-(chloromethyl)-2-methoxybenzene (8i)

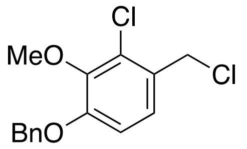

A solution of (4-(benzyloxy)-2-chloro-3-methoxyphenyl)methanol (**7i**, 1.60 g, 5.88 mmol, 1 equiv.) in Et_2O (12 mL) was cooled down to 0 °C and concentrated HCl (22 mL, 264 mmol, 45 equiv.) was added dropwise. The reaction mixture was allowed to warm up to rt and was stirred at this temperature for 30 min. The reaction was quenched by addition of H_2O and Et_2O. The resulting layers were separated, and the aqueous phase was extracted with three times Et_2O. The combined organic layers were dried over Na_2SO_4, filtered and concentrated under reduced pressure to yield the product as a colorless oil (1.67 g, 5.61 mmol, **95%**). **R_f** = 0.57 (10% EtOAc in cyclohexane); **^{1}H NMR** (400 MHz, $CDCl_3$) δ 7.46 – 7.31 (m, 5H), 7.12 (d, J = 8.7 Hz, 1H), 6.86 (d, J = 8.7 Hz, 1H), 5.13 (s, 2H), 4.66 (s, 2H), 3.93 (s, 3H); **^{13}C NMR** (100 MHz, $CDCl_3$) δ 153.2, 146.5, 136.5, 129.0, 128.8, 128.6, 128.3, 127.4, 125.8, 112.6, 71.1, 60.8, 44.1; **HRMS** (pos. ESI): calcd. for $C_{15}H_{14}Cl_2O_2Na$ $[M+Na]^+$: 319.0269, found: 319.0263

2-(4-(Benzyloxy)-2-chloro-3-methoxyphenyl)acetonitrile (9i)

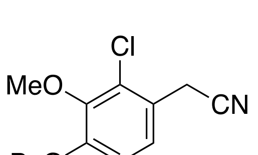

To a solution of 1-(benzyloxy)-3-chloro-4-(chloromethyl)-2-methoxybenzene (**8i**, 1.60 g, 5.38 mmol, 1 equiv.) in DMF (10 mL) was added NaI (81 mg, 0.54 mmol, 0.1 equiv.) and KCN (421 mg, 6.46 mmol, 1.2 equiv.). The solution was heated at 80 °C for 16 h. The solution was then evaporated, water was added, and the aqueous phase was extracted three times with EtOAc. The combined organic phase was washed with water, brine, dried over Na_2SO_4 and evaporated. The residue was purified on silica gel eluting with 10% EtOAc in cyclohexane to afford the desired compound (1.19 g, 4.14 mmol, **77%**) as a light yellow solid. **R***f*= 0.23 (10% EtOAc in cyclohexane); **m.p.:** 103 – 105 °C; **^{1}H NMR** (400 MHz, $CDCl_3$) δ 7.44 – 7.29 (m, 5H), 7.13 (dt, *J* = 8.5, 0.7 Hz, 1H), 6.88 (d, *J* = 8.5 Hz, 1H), 5.13 (s, 2H), 3.90 (s, 3H), 3.74 (d, *J* = 0.7 Hz, 1H); **^{13}C NMR** (101 MHz, $CDCl_3$) δ 152.9, 146.8, 136.5, 128.9, 128.4, 127.5, 124.2, 121.6, 117.3, 113.0, 71.3, 61.0.

1-(4-(Benzyloxy)-2-chloro-3-methoxyphenyl)cyclopentane-1-carbonitrile (10i)

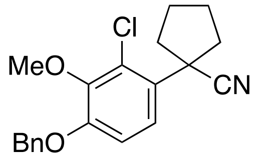

To a mixture of NaH (430 mg, 10.3 mmol, 2.5 equiv., 60% w/w) in dry THF (16.2 mL), was added 2-(4-(benzyloxy)-2-chloro-3-methoxyphenyl)acetonitrile (**9i**, 1.18 g, 4.12 mmol, 1 equiv.) in dry THF (16.2 mL). Then, 1,4-dibromopropane (0.59 mL, 4.95 mmol, 1.2 equiv.) was added. The mixture was allowed to stir at rt for 18 h, and was evaporated. Water was added and the aqueous phase was extracted three times with EtOAc. The combined organic phase was washed with brine, dried over Na_2SO_4 and evaporated. The residue was purified on silica gel eluting with 10% EtOAc in cyclohexane to afford a light yellow oil (1.05 g, 3.06 mmol, **74%**). **R***f* = 0.28 (10% EtOAc in cyclohexane); **^{1}H NMR** (400 MHz, $CDCl_3$) δ 7.43 – 7.26 (m, 5H), 7.02 (d, *J* = 8.9 Hz, 1H), 6.83 (d, *J* = 8.9 Hz, 1H), 5.08 (s, 2H), 3.88 (s, 3H), 2.66 – 2.56 (m, 2H), 2.13 – 1.75 (m, 6H); **^{13}C NMR** (101 MHz, $CDCl_3$) δ 152.7, 147.1, 136.5, 129.7, 129.1, 128.8, 128.3, 127.4, 123.5, 122.2, 112.1, 71.0, 60.7, 46.3, 38.4, 23.7; **HRMS** (pos. ESI) : calcd. for $C_{20}H_{24}ClN_2O_2$ $[M+NH_4]^+$: 359.1521, found: 359.1526

1-(4-(Benzyloxy)-2-chloro-3-methoxy-6-nitrophenyl)cyclopentane-1-carbonitrile (11i)

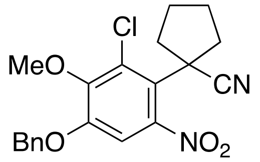

To a solution of 1-(4-(benzyloxy)-2-chloro-3-methoxyphenyl)cyclopentane-1-carbonitrile (**10i**, 1.00 g, 2.93 mmol, 1 equiv.) in Ac_2O (7.5 mL) and AcOH (5.6 mL) was added a solution of HNO_3 (1.83 mL, 43.9 mmol, 15 equiv.) in AcOH (2.4 mL). The mixture was stirred for 5 h at rt and poured into a solution of ice-water. The solution was basified with Na_2CO_3 and the aqueous solution was extracted three times with DCM. The combined organic layer was washed with water, brine and dried over Na_2SO_4. The residue was purified on silica gel column eluting with 10-20% EtOAc in cyclohexane to afford the desired product (690 mg, 1.78 mmol, **61%**) as a yellow solid. **R***f* = 0.46 (20% EtOAc in cyclohexane); **m.p.:** 166 – 168 °C; **^{1}H NMR** (400 MHz, $CDCl_3$) δ 7.43 – 7.32 (m, 5H),

6.96 (s, 1H), 5.11 (s, 2H), 3.92 (s, 3H), 2.99 – 2.81 (m, 2H), 2.03 – 1.75 (m, 6H); ^{13}**C NMR** (101 MHz, $CDCl_3$) δ 152.0, 149.2, 147.3, 135.2, 132.2, 129.2, 129.0, 127.7, 123.2, 121.8, 108.6, 71.8, 61.3, 45.9, 38.7, 24.1; **HRMS** (pos. ESI): calcd. for $C_{20}H_{19}ClN_2O_4Na$ $[M+Na]^+$: 409.0931, found: 409.0926

1-(2-Chloro-4-hydroxy-3-methoxy-6-nitrophenyl)cyclopentane-1-carbonitrile (<u>12i</u>)

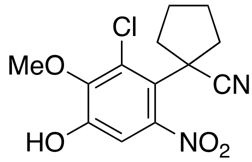

The compound was synthetized following general procedure for benzyl deprotection starting from 1-(4-(benzyloxy)-2-chloro-3-methoxy-6-nitrophenyl)cyclopentane-1-carbonitrile (**11i**). The residue was purified on silica gel eluting with 10-40% EtOAc in cyclohexane to afford a yellow solid (436 mg, 1.47 mmol, **86%**). $\mathbf{R}_f$ = 0.31 (33% EtOAc in cyclohexane); **m.p.:** 122 – 124 °C; ^{1}**H NMR** (400 MHz, $CDCl_3$) δ 6.96 (s, 1H), 6.87 (br s, 1H), 3.89 (s, 3H), 2.93 – 2.83 (m, 2H), 2.00 – 1.74 (m, 6H); ^{13}**C NMR** (101 MHz, $CDCl_3$) δ 150.0, 147.7, 147.0, 131.2, 121.7, 110.9, 61.3, 45.5, 38.5, 23.8; **HRMS** (pos. ESI): calcd. for $C_{13}H_{13}ClN_2O_4Na$ $[M+Na]^+$: 319.0456, found: 319.0462

1-(2-Chloro-3-methoxy-6-nitro-4-(3-(pyrrolidin-1-yl)propoxy)phenyl)cyclopentane-1-carbonitrile (13i)

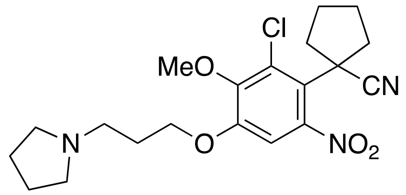

To a solution of 1-(2-chloro-4-hydroxy-3-methoxy-6-nitrophenyl)cyclopentane-1-carbonitrile (**12i**, 250 mg, 0.84 mmol, 1 equiv.) in DMF (5.5 mL) were added 1-(3-chloropropyl)pyrrolidine (**19**, 149 mg, 1.01 mmol, 1.2 equiv.), K_2CO_3 (163 mg, 1.18 mmol, 1.4 equiv.) and NaI (25 mg, 0.17 mmol, 0.2 equiv.). The mixture was heated at 80 °C for 1 h. Water wad added, and the aqueous phase was extracted three times with EtOAc. The combined organic phase was washed with brine, dried over Na_2SO_4 and concentrated under reduced pressure. The residue was purified on silica gel column eluting with 0-10% MeOH in DCM to afford the desired product (246 mg, 0.60 mmol, **72%**) as a light orange oil. $\mathbf{R}_f$= 0.31 (8% MeOH in DCM); ^{1}**H NMR** (400 MHz, $CDCl_3$) δ 6.94 (s, 1H), 4.08 (t, J = 6.3 Hz, 2H), 3.87 (s, 3H), 2.93 – 2.81 (m, 2H), 2.61 (t, J = 7.1 Hz, 2H), 2.53 – 2.47 (m, 4H), 2.02 (tt, J = 7.1, 6.3 Hz, 2H), 1.97 – 1.85 (m, 4H), 1.85 – 1.70 (m, 6H); ^{13}**C NMR** (101 MHz, $CDCl_3$) δ 152.2, 148.6, 147.2, 131.7, 122.4, 121.7, 107.9, 68.1, 61.0, 54.3, 52.6, 45.7, 38.5, 28.5, 23.9, 23.6; **HRMS** (pos. ESI): calcd. for $C_{20}H_{27}ClN_3O_4$ $[M+H]^+$: 408.1685, found: 408.1690

4'-Chloro-5'-methoxy-6'-(3-(pyrrolidin-1-yl)propoxy)spiro[cyclopentane-1,3'-indol]-2'-amine (1i)

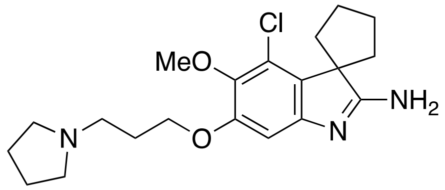

To a solution of 1-(2-chloro-3-methoxy-6-nitro-4-(3-(pyrrolidin-1-yl)propoxy)phenyl)cyclopentane-1-carbonitrile (**13i**, 140 mg, 0.34 mmol, 1 equiv.) in AcOH (2.36 mL) was added zinc dust (337 mg, 5.16 mmol, 15 equiv.). The reaction mixture was heated up to 80 °C and was stirred at that temperature for 1.5 h. The solids were filtered and washed with AcOH. The filtrate was concentrated under reduced pressure. The residue was dissolved in EtOAc and an aqueous solution of NaOH (10 mL, 2M). The layers were separated, and the aqueous layer was extracted three times with EtOAc. The combined organic layers were dried over Na_2SO_4, filtered and concentrated under reduced pressure. The product was obtained as a light brown solid (103 mg, 0.27 mmol, **79%**). **m.p.:** 149 – 151 °C; **^{1}H NMR** (400 MHz, $CDCl_3$) δ 6.71 (s, 1H), 5.50 (br s, 2H), 4.03 (t, *J* = 6.5 Hz, 2H), 3.79 (s, 3H), 2.64 – 2.58 (m, 2H), 2.52 – 2.42 (m, 4H), 2.46 – 2.36 (m, 2H), 2.22 – 2.12 (m, 2H), 2.04 – 1.96 (m, 2H), 1.91 – 1.80 (m, 4H), 1.78 – 1.73 (m, 4H); **^{13}C NMR** (101 MHz, $CDCl_3$) δ 182.6, 152.9, 151.3, 140.8, 129.9, 123.1, 101.6, 67.9, 61.1, 59.8, 54.5, 53.4, 35.0, 29.1, 28.2, 23.7; **HRMS** (pos. ESI): calcd. for $C_{20}H_{29}ClN_3O_2$ $[M+H]^+$: 378.1948, found: 378.1943

11. <u>4',5'-Dimethoxy-6'-(3-(pyrrolidin-1-yl)propoxy)spiro[cyclopentane-1,3'-indol]-2'-amine (1j)</u>

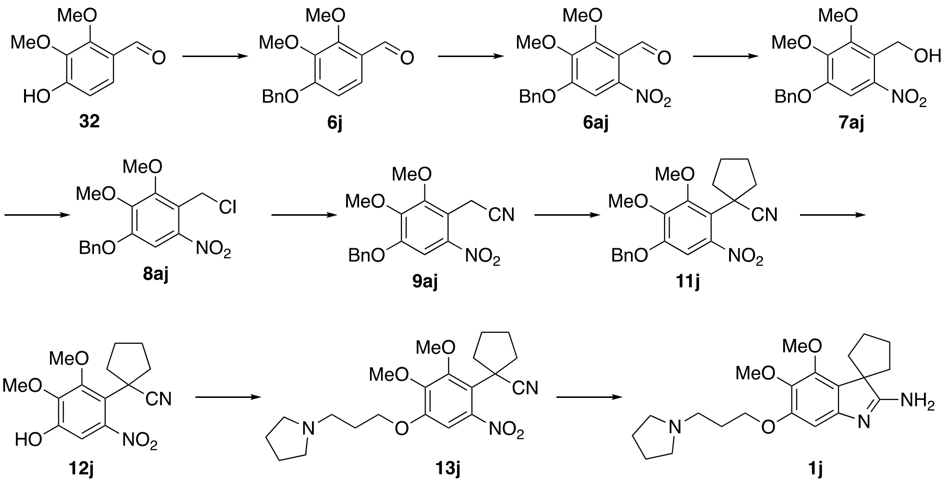

4-Hydroxy-2,3-dimethoxybenzaldehyde (32)

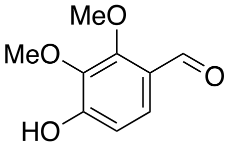

The synthesis was adapted from the following procedure (1). To a solution of 4-hydroxy-2-iodo-3-methoxybenzaldehyde (**17**, 2.49 g, 8.96 mmol, 1 equiv.) in DMF was added a solution of sodium methoxide in MeOH (2.47 g of sodium in 84 mL of MeOH). $CuCl_2$ (601 mg, 4.48 mmol, 0.5 equiv.) was added. The mixture is heated at 160 °C for 2 h. Solvents are then removed under reduced pressure and the residue was poured into water. The resulting layers were separated, and the aqueous phase was extracted three times with EtOAc. The combined organic layers were dried over Na_2SO_4, filtered and concentrated under reduced pressure. Purification on silica gel column eluting with 10-50% EtOAc in cyclohexane afforded the desired product (1.27 g, 6.97 mmol, **78%**) as a light yellow solid. $\mathbf{R}_f$ = 0.40 (25% EtOAc in cyclohexane); **m.p.:** 78 – 80 °C; **^{1}H NMR** (400 MHz, $CDCl_3$) δ 10.16 (d, *J* = 0.8 Hz, 1H), 7.52 (d, *J* = 8.6 Hz, 1H), 6.80 (br s, 1H), 6.76 (dd, *J* = 8.7, 0.8 Hz, 1H), 3.97 (s, 3H), 3.92 (s, 3H); **^{13}C NMR** (101 MHz, $CDCl_3$) δ 188.8, 156.6, 156.3, 139.5, 125.2, 123.1, 111.8, 62.2, 61.2; **HRMS** (pos. APCI) : calcd. for $C_9H_9O_4$ [M-H]$^-$: 181.05008, found: 181.0506

4-(Benzyloxy)-2,3-dimethoxybenzaldehyde (6j)

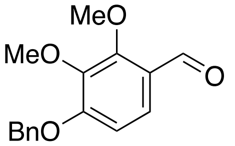

To a solution of 4-hydroxy-2,3-dimethoxybenzaldehyde (1.43 g, 7.85 mmol, 1 equiv.) in DMF (10 mL) were added K_2CO_3 (1.32 g, 9.42 mmol, 1.2 equiv.) and benzyl bromide (1.12 mL, 9.42 mmol, 1.2 equiv.). The mixture was heated at 90 °C for overnight. The reaction mixture was allowed to cool to rt and was poured into water. The resulting layers were separated, and the aqueous phase was extracted three times with EtOAc. The combined organic layers were dried over Na_2SO_4, filtered and concentrated under reduced pressure. Purification on silica gel column eluting with 10-20% EtOAc in cyclohexane afforded the desired compound (1.99 g, 7.30 mmol, **95%**) as a white solid. $\mathbf{R}_f$

= 0.54 (20% EtOAc in cyclohexane); **m.p.:** 44 – 46 °C; **^{1}H NMR** (400 MHz, $CDCl_3$) δ 10.23 (d, *J* = 0.7 Hz, 1H), 7.54 (d, *J* = 8.8 Hz, 1H), 7.44-7.29 (m, 5H), 6.78 (dd, *J* = 8.8, 0.9 Hz, 1H), 5.17 (s, 2H), 4.02 (s, 3H), 3.89 (s, 3H); **^{13}C NMR** (101 MHz, $CDCl_3$) δ 188.9, 158.6, 157.2, 142.2, 136.2, 128.8, 128.4, 127.4, 124.1, 123.7, 109.1, 71.0, 62.5, 61.1; **HRMS** (pos. ESI): calcd. for $C_{16}H_{17}O_4$ [M+H]$^+$: 273.11269, found: 273.1121

4-(Benzyloxy)-2,3-dimethoxy-6-nitrobenzaldehyde (6aj)

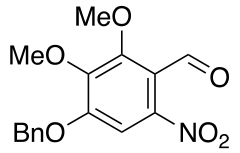

To a solution of 4-(benzyloxy)-2,3-dimethoxybenzaldehyde (**6j**, 1.96 g, 7.20 mmol, 1 equiv.) in AcOH (17 mL) was added dropwise a solution of HNO_3 in AcOH (2:1 v/v, 17 mL) at 0 °C. The solution was stirred at 0 °C for 90 min and then poured into ice-water (150 mL) and extracted three times with DCM. The combined organic layers were washed with saturated aqueous $NaHCO_3$ and brine, dried over Na_2SO_4, and concentrated *in vacuo*. Silica gel column eluting with 15% EtOAc in cyclohexane provided the desired compound (1.80 g, 5.68 mmol, **79%**) as a yellow solid. **R*f*** = 0.27 (10% EtOAc in cyclohexane); **m.p.:** 112 – 114 °C; **^{1}H NMR** (400 MHz, $CDCl_3$) δ 10.21 (d, *J* = 0.4 Hz, 1H), 7.44 – 7.35 (m, 6H), 5.18 (s, 2H), 3.96 (s, 3H), 3.95 (s, 3H); **^{13}C NMR** (101 MHz, $CDCl_3$) δ 187.1, 154.8, 153.5, 147.5, 143.3, 135.1, 129.1, 128.9, 127.8, 121.5, 105.4, 71.8, 63.1, 61.5; **HRMS** (pos. ESI): calcd. for $C_{16}H_{15}O_6NNa$ [M+Na]$^+$: 340.0797, found: 368.1105

(4-(Benzyloxy)-2,3-dimethoxy-6-nitrophenyl)methanol (7aj)

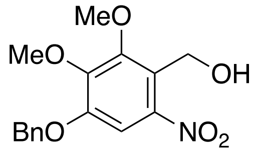

To a solution of 4-(benzyloxy)-2,3-dimethoxy-6-nitrobenzaldehyde (**6aj**, 1.74 g, 5.48 mmol, 1 equiv.) was dissolved in a solution of MeOH (60 mL). $NaBH_4$ (311 mg, 8.23 mmol, 1.5 equiv.) was added portionwise at 0 °C and the mixture was stirred at 0 °C for 30 min. The solution was then allowed to heat at rt, evaporated. Water was added to the residue and the aqueous phase was extracted three times with EtOAc. The combined organic layers were dried over Na_2SO_4, filtered and concentrated under reduced pressure to afford the desired compound (1.63 g, 5.12 mmol, **93%**) as a light-yellow oil. **R*f*** = 0.31 (20% EtOAc in cyclohexane); **^{1}H NMR** (400 MHz, $CDCl_3$) δ 7.45-7.31 (m, 6H), 5.15 (s, 2H), 4.76 (br s, 2H), 3.96 (s, 3H), 3.95 (s, 3H), 2.85 (br s, 1H); **^{13}C NMR** (101 MHz, $CDCl_3$) δ 153.1, 152.0, 148.2, 144.7, 135.7, 129.0, 128.7, 127.7, 124.5, 106.5, 71.6, 62.6, 61.4, 55.8; **HRMS** (pos. ESI): calcd. for $C_{16}H_{17}O_6NNa$ [M+Na]$^+$: 342.0954, found: 342.0948

1-(Benzyloxy)-4-(chloromethyl)-2,3-dimethoxy-5-nitrobenzene (8aj)

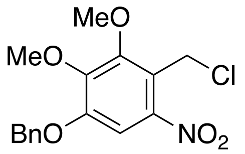

To a solution of (4-(benzyloxy)-2,3-dimethoxy-6-nitrophenyl)methanol (**7aj**, 1.63 g, 5.11 mmol, 1 equiv.) in anhydrous DCM (15.8 mL) at 0 °C was added dropwise $SOCl_2$ (0.85 mL, 10.2 mmol, 2 equiv.). The solution was stirred for 1 h at 0 °C. The solution was then evaporated, DCM and water were added. The aqueous phase was extracted three times with DCM. The combined organic phase was washed with brine, dried over Na_2SO_4 and evaporated to afford the desired compound (1.69 g, 5.00 mmol, **98%**) as a yellow solid. **R**$_f$ = 0.40 (10% EtOAc in cyclohexane); **m.p.:** 82 – 84 °C; **^{1}H NMR** (400 MHz, $CDCl_3$) δ 7.49 (s, 1H), 7.45 – 7.32 (m, 5H), 5.15 (s, 2H), 4.94 (s, 2H), 4.00 (s, 3H), 3.96 (s, 3H); **^{13}C NMR** (101 MHz, $CDCl_3$) δ 153.0, 152.5, 147.5, 143.8, 135.6, 129.0, 128.8, 127.8, 121.1, 106.7, 71.6, 62.4, 61.3, 36.2; **HRMS** (pos. ESI): calcd. for $C_{16}H_{16}ClNO_5Na$ [M+Na]$^+$: 360.0615, found: 360.0609

2-(4-(Benzyloxy)-2,3-dimethoxy-6-nitrophenyl)acetonitrile (9aj)

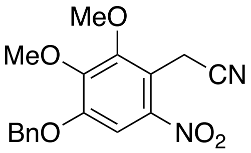

To a solution of 1-(benzyloxy)-4-(chloromethyl)-2,3-dimethoxy-5-nitrobenzene (**8aj**, 3.81 g, 11.3 mmol, 1 equiv.) in MeCN (100 mL), KCN (8.08 g, 12.4 mmol, 1.1 equiv.) and 18-crown-6 (298 mg, 1.13 mmol, 0.1 equiv.) were added at rt. The mixture was allowed to stir at rt for 16 h and evaporated. Water and K_2CO_3 (500 mg) were added and the aqueous phase was extracted three times with EtOAc. The combined organics were washed with brine, dried over Na_2SO_4 and evaporated. The residue was purified on silica gel eluting with 20% EtOAc in cyclohexane to afford the desired product (1.64 g, 5.15 mmol, **46%**) as a light-yellow solid. **R**$_f$ = 0.38 (20% EtOAc in cyclohexane); **m.p.:** 89 – 91 °C; **^{1}H NMR** (400 MHz, $CDCl_3$) δ 7.57 (s, 1H), 7.44 – 7.33 (m, 5H), 5.15 (s, 2H), 4.00 (s, 3H), 3.99 (s, 2H), 3.96 (s, 3H); **^{13}C NMR** (101 MHz, $CDCl_3$) δ 152.5, 152.4, 147.9, 143.4, 135.5, 129.2, 128.8, 127.9, 117.3, 114.4, 106.8, 71.7, 62.1, 61.5, 15.4; **HRMS** (pos. ESI): calcd. for $C_{17}H_{16}N_2O_5Na$ [M+Na]$^+$: 351.0957, found: 351.0951

1-(4-(Benzyloxy)-2,3-dimethoxy-6-nitrophenyl)cyclopentane-1-carbonitrile (11j)

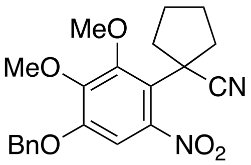

To a solution of 2-(4-(benzyloxy)-2,3-dimethoxy-6-nitrophenyl)acetonitrile (**9aj**, 1.45 g, 4.42 mmol, 1 equiv.) in dry DMF (14 mL) were added $CsCO_3$ (7.20 g, 13.2 mmol, 3 equiv.) and 1,4-dibromobutane (580 µL, 4.86 mmol, 1.1 equiv.) at rt. The mixture was stirred for 1 day at rt and evaporated. Water was added to the residue and the aqueous phase was extracted three times with EtOAc. The combined organic phase was washed with brine, dried over Na_2SO_4 and evaporated. Purification on silica gel eluting with 10-30% EtOAc in cyclohexane afford the desired product (1.07 g, 2.80 mmol, **63%**) as a yellow solid. **R**$_f$ = 0.56 (20% EtOAc in cyclohexane); **m.p.:** 113 – 115 °C; **^{1}H NMR** (500 MHz, $CDCl_3$) δ 7.44 – 7.33 (m, 5H), 6.82 (s, 1H), 5.10 (s, 2H), 4.06 (s, 3H), 3.92 (s, 3H), 2.83 – 2.71 (m, 2H), 2.00 – 1.75 (m, 6H); **^{13}C NMR** (126 MHz, $CDCl_3$) δ 154.2, 152.4, 146.0, 145.4, 135.5, 129.0, 128.8, 127.6, 123.4, 119.3, 104.7, 71.5, 61.7, 61.2, 43.8, 38.5, 23.6; **HRMS** (pos. ESI): calcd. for $C_{21}H_{22}N_2O_5Na$ [M + Na]$^+$: 405.1426, found: 405.1421

1-(4-Hydroxy-2,3-dimethoxy-6-nitrophenyl)cyclopentane-1-carbonitrile (12j)

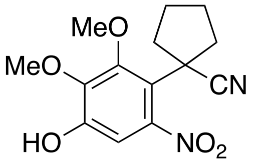

The compound was synthetized following general procedure for benzyl deprotection starting from 1-(4-(benzyloxy)-2,3-dimethoxy-6-nitrophenyl)cyclopentane-1-carbonitrile (**11j**). The residue was purified on silica gel eluting with 10-40% EtOAc in cyclohexane to afford a light yellow solid (451 mg, 1.54 mmol, **62%**). **R*f*** = 0.42 (30% EtOAc in cyclohexane); **m.p.:** 144 – 146 °C; **¹H NMR** (400 MHz, $CDCl_3$) δ 6.81 (s, 1H), 5.97 (s, 1H), 4.01 (s, 3H), 3.92 (s, 3H), 2.81-2.71 (m, 2H), 1.99 – 1.73 (m, 6H); **¹³C NMR** (101 MHz, $CDCl_3$) δ 153.3, 149.8, 146.7, 142.6, 123.4, 118.5, 106.6, 61.0, 60.9, 43.7, 38.5, 23.5

1-(2,3-Dimethoxy-6-nitro-4-(3-(pyrrolidin-1-yl)propoxy)phenyl)cyclopentane-1-carbonitrile (13j)

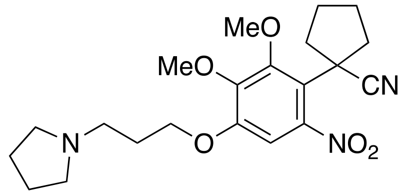

To a solution of 1-(4-hydroxy-2,3-dimethoxy-6-nitrophenyl)cyclopentane-1-carbonitrile (**12j**, 470 mg, 1.61 mmol, 1 equiv.) in DMF (10.5 mL) were added 1-(3-chloropropyl)pyrrolidine (**19**, 311 mg, 1.93 mmol, 1.2 equiv.), K_2CO_3 (311 mg, 2.25 mmol, 1.4 equiv.) and NaI (48 mg, 0.32 mmol, 0.2 equiv.). The mixture was heated at 80 °C for 1 h. Water wad added, and the aqueous phase was extracted three times with EtOAc. The combined organic phase was washed with brine, dried over Na_2SO_4 and concentrated under reduced pressure. The residue was purified on silica gel column eluting with 0-10% MeOH in DCM afforded the desired product (504 mg, 1.17 mmol, **73%**) as a yellow oil. **R*f*** = 0.58 (4% MeOH in DCM + 2% NEt_3); **¹H NMR** (400 MHz, $CDCl_3$) δ 6.76 (s, 1H), 4.04 (t, *J* = 6.4 Hz, 2H), 3.99 (s, 3H), 3.85 (s, 3H), 2.76 – 2.66 (m, 2H), 2.60 (t, *J* = 7.2 Hz, 2H), 2.53 – 2.46 (m, 4H), 2.06 – 1.97 (m, 2H), 1.92 – 1.71 (m, 10H); **¹³C NMR** (101 MHz, $CDCl_3$) δ 154.0, 152.7, 146.1, 145.0, 123.4, 118.7, 104.2, 67.9, 61.6, 61.0, 54.3, 52.8, 43.7, 38.4, 28.6, 23.6, 23.6; **HRMS** (pos. ESI): calcd. for $C_{21}H_{30}N_3O_5$ $[M+H]^+$: 404.2185, found: 404.2180

4',5'-Dimethoxy-6'-(3-(pyrrolidin-1-yl)propoxy)spiro[cyclopentane-1,3'-indol]-2'-amine (1j)

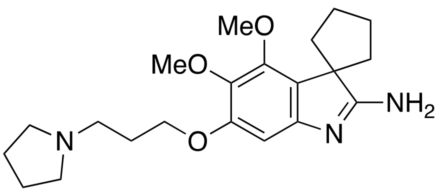

To a solution of 1-(2,3-dimethoxy-6-nitro-4-(3-(pyrrolidin-1-yl)propoxy)phenyl)cyclopentane-1-carbonitrile (**13j**, 440 mg, 1.09 mmol, 1 equiv.) in AcOH (7.9 mL) was added zinc dust (1.07 g, 16.4 mmol, 15 equiv.). The reaction mixture was heated up to 80 °C and was stirred at that temperature for 1.5 h. The solids were filtered and washed with AcOH. The filtrate was concentrated under reduced pressure. The residue was dissolved in EtOAc and the resulting solution was washed with

an aqueous solution of NaOH (5 mL, 1M). The layers were separated, and the aqueous layer was extracted three times with EtOAc. The combined organic layers were dried over Na_2SO_4, filtered and concentrated under reduced pressure. The product was obtained as a white solid (384 mg, 1.03 mmol, **94%**). **m.p.:** 144 – 146 °C; **^{1}H NMR** (400 MHz, $CDCl_3$) δ 6.56 (s, 1H), 5.45 (br s, 2H), 4.03 (t, *J* = 6.4 Hz, 2H), 3.95 (s, 3H), 3.80 (s, 3H), 2.64 – 2.58 (m, 2H), 2.52 – 2.47 (m, 4H), 2.22 – 2.13 (m, 2H), 2.11 – 1.96 (m, 4H), 1.92 – 1.78 (m, 4H), 1.78 – 1.72 (m, 4H); **^{13}C NMR** (101 MHz, $CDCl_3$) δ 182.0, 153.3, 150.4, 148.8, 137.6, 125.6, 98.2, 67.7, 61.1, 60.9, 58.5, 54.5, 53.5, 37.1, 29.3, 28.2, 23.7; **HRMS** (pos. ESI): calcd. for $C_{21}H_{32}N_3O_3$ $[M+H]^+$ 374.2438, found: 374.2438

12. <u>1-(2'-Amino-5'-methoxy-6'-(3-(pyrrolidin-1-yl)propoxy)spiro[cyclopentane-1,3'-indol]-4'-yl)ethan-1-one (1k)</u>

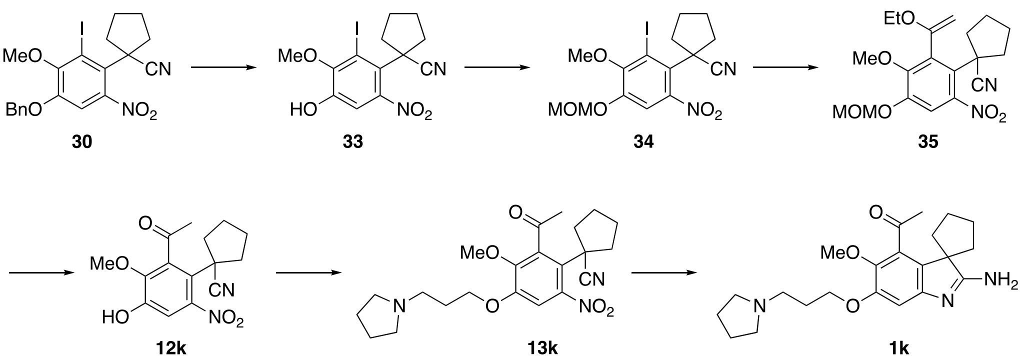

1-(4-Hydroxy-2-iodo-3-methoxy-6-nitrophenyl)cyclopentane-1-carbonitrile (33)

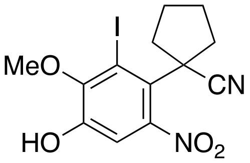

The compound was synthetized following general procedure for benzyl deprotection starting from 1-(4-(benzyloxy)-2-iodo-3-methoxy-6-nitrophenyl)cyclopentane-1-carbonitrile. The residue was purified on silica gel eluting with 10-30% EtOAc in cyclohexane to afford the desired product (49 mg, 0.13 mmol, 67%) as a slightly brown powder. $\mathbf{R}_f$ = 0.40 (30% EtOAc in cyclohexane); **^{1}H NMR** (400 MHz, $CDCl_3$) δ 7.08 (s, 1H), 6.25 (br s, 1H), 3.87 (s, 3H), 3.18 – 3.07 (m, 2H), 2.00 – 1.75 (m, 6H); **^{13}C NMR** (101 MHz, $CDCl_3$) δ 150.5, 148.8, 148.1, 126.5, 121.8, 112.8, 98.5, 61.3, 48.5, 39.4, 24.2; **HRMS** (pos. ESI): calcd. for $C_{13}H_{13}IN_2O_4Na$ [M+Na]$^+$: 410.9812, found: 410.9812

1-(2-Iodo-3-methoxy-4-(methoxymethoxy)-6-nitrophenyl)cyclopentane-1-carbonitrile (34)

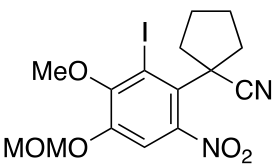

To a solution of 1-(4-hydroxy-2-iodo-3-methoxy-6-nitrophenyl)cyclopentane-1-carbonitrile (**33**, 650 mg, 1.68 mmol, 1 equiv.) in DCM (10 mL) were added DIEA (0.57 mL, 3.35 mmol, 2 equiv.) and MOMCl 2.1M in toluene (0.80 mL, 3.35 mmol, 2 equiv.) at 0 °C. The mixture was allowed to stir at rt for 16 h and evaporated. The residue was purified on silica gel column eluting with 50-80% DCM in cyclohexane to afford the desired product (520 mg, 1.20 mmol, 72%) as a yellow oil. $\boldsymbol{R_f}$ = 0.38 (20% cyclohexane in DCM); **^{1}H NMR** (400 MHz, $CDCl_3$) δ 7.26 (s, 1H), 5.21 (s, 1H), 3.85 (s, 3H), 3.46 (s, 3H), 3.15 – 3.03 (m, 2H), 1.98 – 1.74 (m, 6H); **^{13}C NMR** (101 MHz, $CDCl_3$) δ 152.2, 148.4, 147.9, 128.0, 121.7, 112.4, 100.2, 95.4, 60.7, 56.8, 48.4, 39.3, 24.1; **HRMS** (pos. ESI): calcd. for $C_{15}H_{17}IN_2O_5Na$ [M+Na]$^+$: 455.0080, found : 445.0074

1-(2-(1-Ethoxyvinyl)-3-methoxy-4-(methoxymethoxy)-6-nitrophenyl)cyclopentane-1-carbonitrile (35)

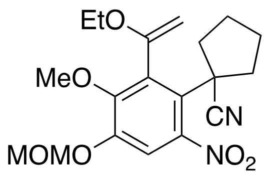

To a solution of 1-(2-iodo-3-methoxy-4-(methoxymethoxy)-6-nitrophenyl)cyclopentane-1-carbonitrile (**34**, 400 mg, 0.93 mmol, 1 equiv.) in THF at rt under argon, tributyl(1-ethoxyvinyl)tin (0.63 mmol, 1.85 mmol, 2 equiv.) and bis(triphenylphosphine)palladium(II) dichloride (65 mg, 0.09 mmol, 0.1 equiv.) were added. The mixture was allowed to stir at 100 °C under microwave irradiation for 16 h. The solution was then evaporated and purified on silica gel column eluting with 10-40% EtOAc in cyclohexane to afford the desired product (178 mg 0.47 mmol, **51%**) as a yellow oil. **R**$_f$ = 0.36 (20% EtOAc in cyclohexane); **^{1}H NMR** (400 MHz, $CDCl_3$) δ 7.38 (s, 1H), 5.21 (s, 2H) 4.49 (d, *J* = 2.9 Hz, 1H), 4.39 (d, *J* = 2.9 Hs, 1H), 3.96 – 3.88 (m, 2H), 3.84 (s, 3H), 3.47 (s, 3H), 3.10 – 2.83 (m, 1H), 2.74 – 2.53 (m, 1H), 2.05 – 1.67 (m, 6H), 1.36 (t, *J* = 7.0 Hz, 3H); **^{13}C NMR** (101 MHz, $CDCl_3$) δ 155.6, 151.3, 149.6, 148.1, 136.2, 126.3, 123.3, 112.9, 95.2, 90.1, 63.6, 61.8, 56.7, 45.1, 39.4, 38.4, 23.7, 14.6; **HRMS** (pos. ESI): calcd. for $C_{18}H_{18}NO_3Na$ [M + Na]$^+$: 399.1527, found: 399.1527

1-(2-Acetyl-4-hydroxy-3-methoxy-6-nitrophenyl)cyclopentane-1-carbonitrile (12k)

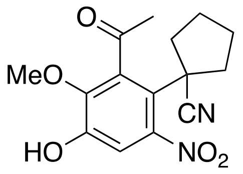

To a solution of 1-(2-(1-ethoxyvinyl)-3-methoxy-4-(methoxymethoxy)-6-nitrophenyl)cyclopentane-1-carbonitrile (**35**, 190 mg, 0.51 mmol, 1 equiv.) in MeOH (8 mL), HCl 4M in dioxane (4 mL) was added at 0 °C. The solution was stirred for 1 h at rt, evaporated and purified on silica gel eluting with 20-30% EtOAc in cyclohexane to afford the desired product (150 mg, 0.49 mmol, **98%**) as a yellow solid. **R**$_f$ = 0.20 (20% EtOAc in cyclohexane); **m.p.**: 136 – 138 °C; **^{1}H NMR** (400 MHz, $CDCl_3$) δ 7.21 (s, 1H), 6.90 (br s, 1H), 3.78 (s, 3H), 2.65 (s, 3H), 2.59 – 2.50 (m, 2H), 1.97 – 1.74 (m, 6H); **^{13}C NMR** (101 MHz, $CDCl_3$) δ 202.8, 149.6, 148.3, 146.4, 139.3, 122.6, 120.8, 113.9, 62.7, 44.7, 39.9, 33.7, 23.9; **HRMS** (pos. ESI): calcd. for $C_{15}H_{16}N_2O_5Na$ [M + Na]$^+$: 327.0951, found: 327.0951

1-(2-Acetyl-3-methoxy-6-nitro-4-(3-(pyrrolidin-1-yl)propoxy)phenyl)cyclopentane-1-carbonitrile (13k)

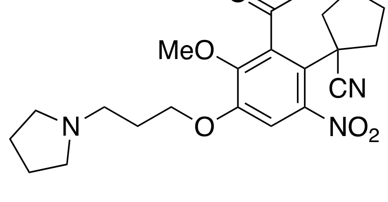

To a solution of 1-(2-acetyl-4-hydroxy-3-methoxy-6-nitrophenyl)cyclopentane-1-carbonitrile (**12k**, 250 mg, 0.84 mmol, 1 equiv.) in DMF (3.2 mL) was added 1-(3-chloropropyl)pyrrolidine (**19**, 87 mg, 0.59 mmol, 1.2 equiv.), K_2CO_3 (95 mg, 0.69 mmol, 1.4 equiv.) and NaI (15 mg, 0.10 mmol, 0.2 equiv.). The mixture was heated at 80 °C for 1 h. Water wad added, and the aqueous phase was extracted three times with EtOAc. The combined organic phase was washed with brine, dried over Na_2SO_4 and concentrated under reduced pressure. The residue was purified on silica gel column eluting with 0-10% MeOH in DCM afforded the desired product (149 mg, 0.36 mmol, **73%**) as a light orange oil. **R***f* = 0.60 (8% MeOH in DCM + 2% NEt_3); **^{1}H NMR** (400 MHz, $CDCl_3$) δ 7.17 (s, 1H), 4.14 (t, *J* = 6.2 Hz, 2H), 3.79 (s, 3H), 2.91 (t, *J* = 7.4 Hz, 2H), 2.89 – 2.82 (m, 4H), 2.58 (s, 3H), 2.53 – 2.46 (m, 2H), 2.24 – 2.15 (m, 2H), 1.93 – 1.73 (m, 10H); **^{13}C NMR** (101 MHz, $CDCl_3$) δ 202.2, 151.3, 147.9, 147.8, 139.1, 122.5, 121.5, 110.5, 67.5, 62.0, 54.1, 52.5, 44.7, 39.8, 33.6, 27.2, 23.8, 23.5; **HRMS** (pos. ESI): calcd. for $C_{22}H_{30}N_3O_5$ [M + H]$^+$: 416.2180, found: 416.2180

1-(2'-Amino-5'-methoxy-6'-(3-(pyrrolidin-1-yl)propoxy)spiro[cyclopentane-1,3'-indol]-4'-yl)ethan-1-one (1k)

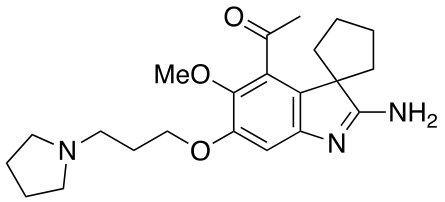

To a solution of 1-(2-Acetyl-3-methoxy-6-nitro-4-(3-(pyrrolidin-1-yl)propoxy)phenyl)-cyclopentane-1-carbonitrile (**13k**, 150 mg, 0.49 mmol, 1 equiv.) in AcOH (2.14 mL) was added zinc dust (307 mg, 4.69 mmol, 15 equiv.). The reaction mixture was heated up to 80 °C and was stirred at that temperature for 1.5 h. The solids were filtered and washed with AcOH. The filtrate was concentrated under reduced pressure. The residue was dissolved in EtOAc and an aqueous solution of NaOH (10 mL, 2M). The layers were separated, and the aqueous layer was extracted three times with EtOAc. The combined organic layers were dried over Na_2SO_4, filtered and concentrated under reduced pressure. The product was obtained as a light-yellow solid (78 mg, 0.27 mmol, **78%**). **^{1}H NMR** (400 MHz, $CDCl_3$) δ 6.77 (s, 1H), 6.39 (br s, 2H), 3.96 (t, *J* = 6.3 Hz, 2H), 3.70 (s, 3H), 2.59 – 2.54 (m, 2H), 2.48 – 2.43 (m, 7H), 2.19 – 2.09 (m, 2H), 1.99 – 1.64 (m, 14H); **^{13}C NMR** (101 MHz, $CDCl_3$) δ 204.5, 183.2, 151.7, 150.6, 141.3, 131.8, 128.8, 103.0, 67.5, 61.9, 59.0, 54.4, 53.3, 37.8, 33.2, 29.0, 27.8, 23.6; **HRMS** (pos. ESI): calcd. for $C_{22}H_{32}N_3O_3$ [M + H]$^+$: 386.2438, found: 386.2438

13. 5'-Ethoxy-6'-(3-(pyrrolidin-1-yl)propoxy)spiro[cyclohexane-1,3'-indol]-2'-amine (1l)

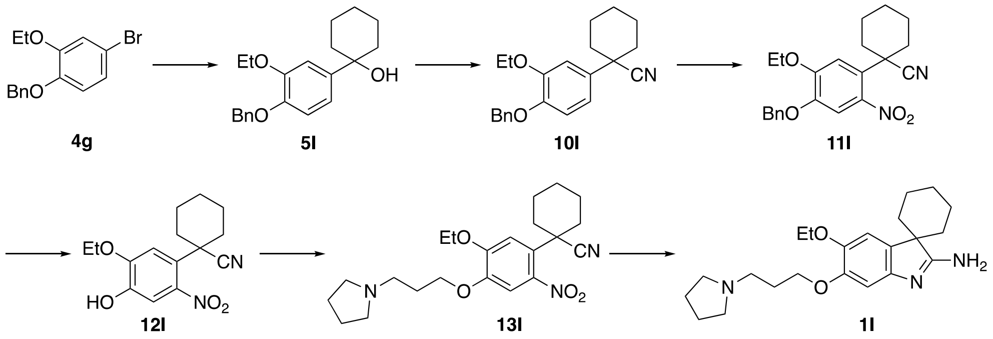

1-(4-(Benzyloxy)-3-ethoxyphenyl)cyclohexan-1-ol (5l)

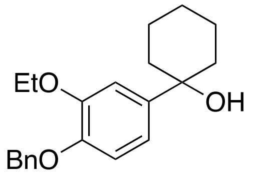

Synthesis was done according to the procedure used for 3-(4-(benzyloxy)-3-methoxyphenyl)pentan-3-ol (**5o**). **^{1}H NMR** (300 MHz, Acetone-d$_6$): δ 7.52 (m, 2H), 7.37 (m, 3H), 7.22 (d, *J* = 3.0 Hz, 1H), 7.02 (dd, *J* = 8.7, 3.0 Hz, 1H), 6.96 (d, *J* = 8.7 Hz, 1H), 5.12 (s, 2H), 4.11 (q, *J* = 9.0 Hz, 2H), 3.57 (s, 1H), 1.79 (m, 8H), 1.56 (m, 2H), 1.39 (t, *J* = 9.0 Hz, 3H); **^{13}C NMR** (101 MHz, Acetone-d$_6$) δ 149.8, 148.2, 145.7, 139.1, 129.1, 128.3, 117.9, 115.5, 112.7, 72.7, 71.8, 65.2, 39.8, 26.4, 22.9, 15.4; **MS** (pos. ESI): *m/z* (%): 349 (100), 309 (23); **HRMS** (pos. ESI): *m/z* calcd. for $C_{21}H_{26}O_3Na$ $[M+Na]^+$: 349.1774; found: 349.1776

1-(4-(Benzyloxy)-3-ethoxyphenyl)cyclohexane-1-carbonitrile (10l)

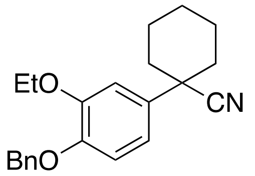

Synthesis was done according to the procedure used for 2-(4-(benzyloxy)-3-methoxyphenyl)-2-ethylbutanenitrile (**10o**). **^{1}H NMR** (300 MHz, CDCl$_3$) δ 7.16 (m, 3H), 6.84 (d, *J* = 3.0 Hz, 1H), 6.73 (dd, *J* = 8.4, 3.0 Hz, 1H), 6.68 (d, *J* = 8.4 Hz, 1H), 4.93 (s, 2H), 3.93 (q, *J* = 9.0 Hz, 2H), 1.93 (m, 2H), 1.58 (m, 7H), 1.25 (t, *J* = 9.0 Hz, 3H), 1.05 (m, 1H); **^{13}C NMR** (101 MHz, CDCl$_3$) δ 149.4, 148.4, 137.6, 134.9, 128.6, 127.9, 127.3, 123.0, 117.9, 115.0, 112.2, 71.4, 65.1, 43.9, 37.6, 27.0, 25.1, 23.7, 15.1

1-(4-(benzyloxy)-5-ethoxy-2-nitrophenyl)cyclohexane-1-carbonitrile (11l)

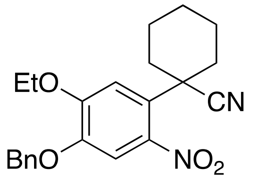

Synthesis was done according to the procedure used for 2-(4-(benzyloxy)-5-methoxy-2-nitrophenyl)-2-ethylbutanenitrile (**11o**). **^{1}H NMR** (300 MHz, Acetone-d$_6$) δ 7.57 (s, 1H), 7.54 (m, 2H), 7.41 (m, 3H), 7.29 (s, 1H), 5.29 (s, 2H), 4.28 (q, *J* = 9.0 Hz, 2H), 2.42 (m, 2H), 1.86 (m, 5H), 1.44 (t, *J* = 9.0 Hz, 3H), 1.37 (m, 3H); **^{13}C NMR** (101 MHz, Acetone-d$_6$) δ 14.9, 24.2, 25.4, 27.5, 37.6, 43.1, 65.9, 71.8, 112.3, 112.5, 120.8, 128.3 (C-1′), 128.5, 128.9, 129.4, 137.5, 143.9, 148.5, 153.0; **MS** (pos. ESI):

m/*z* (%): 313 (100); **HRMS** (pos. ESI): *m*/*z* calcd. for $C_{15}H_{18}O_4N_2Na$ [M+Na]$^+$: 313.1159; found: 313.1159

1-(5-Ethoxy-4-hydroxy-2-nitrophenyl)cyclohexane-1-carbonitrile (12l)

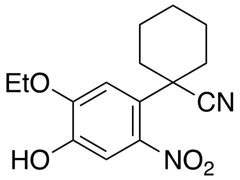

Synthesis was done according to the procedure used for 2-ethyl-2-(4-hydroxy-5-methoxy-2-nitrophenyl)butanenitrile (**12o**). **^{1}H NMR** (300 MHz, Acetone-d$_6$) δ 8.62 (s, 1H), 7.26 (s, 1H), 7.18 (s, 1H), 4.20 (q, *J* = 9.0 Hz, 2H), 2.32 (m, 2H), 1.93 (m, 2H), 1.74 (m, 5H), 1.34 (t, *J* = 9.0 Hz, 3H), 1.26 (m, 1H); **^{13}C NMR** (101 MHz, Acetone-d$_6$) δ 150.9, 147.2, 144.3, 126.8, 120.9, 113.5, 111.8, 65.9, 43.0, 37.2, 25.4, 24.2, 14.8

1-(5-Ethoxy-2-nitro-4-(3-(pyrrolidin-1-yl)propoxy)phenyl)cyclohexane-1-carbonitrile (13l)

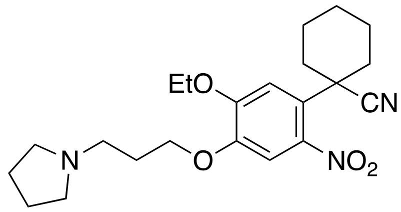

Synthesis was done according to the procedure used for 2-ethyl-2-(5-methoxy-2-nitro-4-(3-(pyrrolidin-1-yl)propoxy)phenyl)butanenitrile (**13o**). **^{1}H NMR** (300 MHz, Acetone-d$_6$) δ 7.50 (s, 1H), 7.26 (s, 1H), 4.26 (m, 4H), 2.79 (m, 6H), 2.41 (m, 2H), 1.83 (m, 9H), 1.43 (t, *J* = 7.8 Hz, 3H), 1.36 (m, 1H); **^{13}C NMR** (101 MHz, Acetone-d$_6$) δ 152.8, 149.1, 144.1, 127.9, 120.8, 112.5, 111.8, 68.4, 65.9, 54.4, 52.9, 43.1, 37.1, 25.4, 24.2, 24.1, 15.0

5'-Ethoxy-6'-(3-(pyrrolidin-1-yl)propoxy)spiro[cyclohexane-1,3'-indol]-2'-amine (1l)

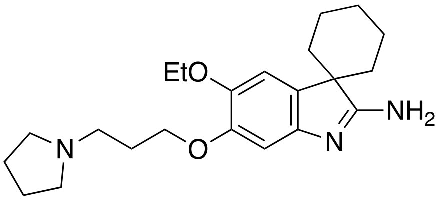

Synthesis was done according to the procedure used for 3,3-diethyl-5-methoxy-6-(3-(pyrrolidin-1-yl)propoxy)-3*H*-indol-2-amine (**1o**). **^{1}H NMR** (400 MHz, CDCl$_3$) δ 7.14 (s, 1H), 6.82 (s, 1H), 5.30 (s, 2H), 4.04 (q, *J* = 6.9 Hz, 2H), 2.66 – 2.59 (m, 2H), 2.55 – 2.48 (m, 4H), 2.05 – 1.96 (m, 3H), 1.94 – 1.82 (m, 3H), 1.80 – 1.70 (m, 6H), 1.63 – 1.56 (m, 4H), 1.52 – 1.41 (m, 2H), 1.38 (t, *J* = 7.0 Hz, 3H).**^{13}C NMR** (101 MHz, CDCl$_3$) δ 179.4, 150.3, 143.4, 131.6, 130.1, 114.6, 103.1, 67.9, 67.4, 54.4, 53.4, 52.6, 34.1, 29.1, 25.3, 23.7, 21.6, 15.6; **HRMS** (pos. APCI):, calcd.for $C_{22}H_{34}N_3O_2$ [M + H]$^+$: 372.26510; found: 372.26492

14. 5'-Isopropoxy-6'-(3-(pyrrolidin-1-yl)propoxy)spiro[cyclobutane-1,3'-indol]-2'-amine (1m)

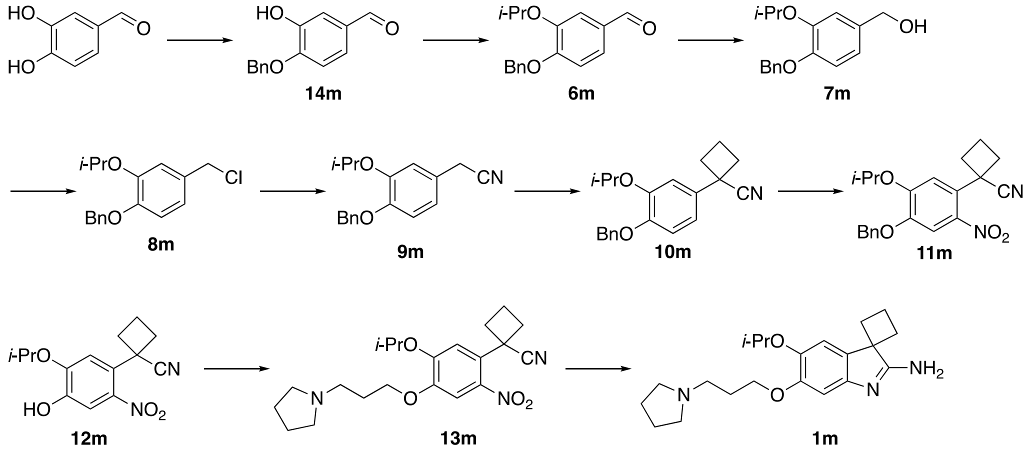

4-(Benzyloxy)-3-hydroxybenzaldehyde (14m)

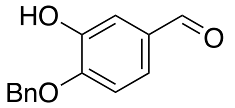

To a solution of 3,4-dihydroxybenzaldehyde (7.0 g, 51 mmol, 1 equiv.) in DMF (60 mL) was added K_2CO_3 (6.1 g, 44 mmol, 0.9 equiv.). The resulting suspension was heated up to 60 °C and was stirred at that temperature for 4 h. The reaction mixture was allowed to cool down to rt and benzyl bromide (6.5 mL, 55 mmol, 1.1 eq) was added. The resulting suspension was stirred at rt for 21 h and was then poured into water (200 mL). The precipitate was filtered and purified by recrystallization from toluene. The product was obtained as a colorless solid (6.9 g, 30 mmol, 60%). **R***f* = 0.28 (33% EtOAc in cyclohexane); **¹H NMR** (400 MHz, $CDCl_3$) δ 9.82 (s, 1H), 7.46 (d, *J* = 2.0 Hz, 1H), 7.44 – 7.36 (m, 6H), 7.04 (d, *J* = 8.2 Hz, 1H), 5.90 (s, 1H), 5.20 (s, 2H). Spectral data matched reported literature values (7).

4-(Benzyloxy)-3-isopropoxybenzaldehyde (6m)

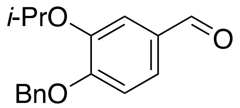

To a solution of 4-(benzyloxy)-3-hydroxybenzaldehyde (**14m,** 3.00 g, 13.1 mmol, 1 equiv.) in DMF (20 mL) were added K_2CO_3 (3.63 g, 26.3 mmol, 2.00 equiv.) and isopropyl *p*-toluenesulfonate (8.45 g, 39.4 mmol, 3.00 equiv.). The resulting suspension was heated up to 80 °C and was stirred at that temperature for 16 h. After cooling down to rt, the reaction was quenched by addition of a saturated aqueous solution of NH_4Cl (10 mL). The resulting layers were separated, and the aqueous phase was extracted three times with DCM. The combined organic layers were dried over Na_2SO_4, filtered and concentrated under reduced pressure. The residue was purified by silica gel column eluting with 9-17% EtOAc in cyclohexane. The product was obtained as a light-yellow oil (3.00 g, 11.1 mmol, 85%). **R***f* = 0.35 (25% EtOAc in cyclohexane); **¹H NMR** (400 MHz, $CDCl_3$) δ 9.83 (s, 1H), 7.50 – 7.29 (m, 7H), 7.01 (d, *J* = 8.2 Hz, 1H), 5.22 (s, 2H), 4.63 (hept, *J* = 6.2 Hz, 1H), 1.39 (d, *J* = 6.1 Hz, 6H); **¹³C NMR** (101 MHz, $CDCl_3$) δ 191.0, 155.2, 148.7, 136.6, 130.6, 128.7, 128.1, 127.1, 126.4, 114.8, 113.7, 72.1, 71.0, 22.2; **MS** (pos. APCI), *m*/*z* (%): 288 (17), 272 (19), 271 (100), 91 (15); **HRMS** (pos. APCI): calcd. for $C_{17}H_{19}O_3$ $[M + H]^+$: 271.13287; found: 271.13272

(4-(Benzyloxy)-3-isopropoxyphenyl)methanol (7m)

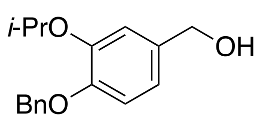

A solution of 4-(benzyloxy)-3-isopropoxybenzaldehyde (**6m,** 3.00 g, 11.1 mmol, 1 equiv.) in THF (20 mL) was cooled down to 0 °C. $LiAlH_4$ (1.00 g, 26.3 mmol, 2.37 equiv.) was added portionwise. The reaction mixture was allowed to warm up to rt and was stirred at this temperature for 4 h. The reaction mixture was cooled down to 0 °C and the reaction was quenched by successive addition of H_2O (1 mL), an aqueous solution of NaOH (20%, 2 mL), and H_2O (3 mL). The resulting suspension was stirred at 0 °C for 30 min and was diluted with Et_2O (30 mL). The precipitate was filtered through a plug of kieselguhr and the filter cake was washed three times with Et_2O. The filtrate was dried over Na_2SO_4, filtered and concentrated under reduced pressure. The residue was purified on silica gel column eluting with 50% EtOAc in cyclohexane to yield the product as a colorless solid (2.90 g, 10.6 mmol, 94%). $\mathbf{R}_f$ = 0.27 (33% EtOAc in cyclohexane); **m.p.**: 43 – 45 °C; **^{1}H NMR** (400 MHz, $CDCl_3$) δ 7.47 – 7.27 (m, 5H), 6.98 (d, *J* = 2.0 Hz, 1H), 6.90 (d, *J* = 8.2 Hz, 1H), 6.85 (dd, *J* = 8.2, 2.0 Hz, 1H), 5.12 (s, 2H), 4.62 – 4.47 (m, 3H), 1.74 (s, 1H), 1.37 (d, *J* = 6.1 Hz, 6H); **^{13}C NMR** (101 MHz, $CDCl_3$) δ 149.5, 148.5, 137.6, 134.7, 128.5, 127.8, 127.4, 120.4, 116.7, 115.8, 72.3, 71.6, 65.3, 22.4; **MS** (pos. APCI), *m*/*z* (%): 290 (100), 255 (42), 213 (12), 91 (17); **HRMS** (pos. APCI): calcd. for $C_{17}H_{24}NO_3$ $[M + NH_4]^+$: 290.17507; found: 290.17508

1-(Benzyloxy)-4-(chloromethyl)-2-isopropoxybenzene (8m)

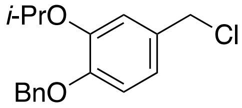

A solution of (4-(benzyloxy)-3-isopropoxyphenyl)methanol (**7m**, 2.90 g, 10.6 mmol, 1 equiv.) in Et_2O (25 mL) was cooled down to 0 °C and conc. HCl (16 mL, 160 mmol, 15 equiv.) was added dropwise. The reaction mixture was allowed to warm up to rt and was stirred at this temperature for 30 min. The reaction was quenched by addition of H_2O (50 mL) and Et_2O (50 mL). The resulting layers were separated, and the aqueous phase was extracted three times with Et_2O. The combined organic layers were dried over Na_2SO_4, filtered and concentrated under reduced pressure to yield the product as a yellow oil (3.03 g, 10.4 mmol, 98%). $\mathbf{R}_f$ = 0.56 (33% EtOAc in cyclohexane); **^{1}H NMR** (400 MHz, $CDCl_3$) δ 7.46 – 7.27 (m, 5H), 6.98 (d, *J* = 1.7 Hz, 1H), 6.93 – 6.83 (m, 2H), 5.12 (s, 2H), 4.62 – 4.47 (m, 3H), 1.37 (d, *J* = 6.1 Hz, 6H); **^{13}C NMR** (101 MHz, $CDCl_3$) δ 150.2, 148.5, 137.4, 130.9, 128.6, 127.9, 127.3, 122.0, 118.0, 115.4, 72.4, 71.5, 46.6, 22.4; **MS** (pos. APCI), *m*/*z* (%): 310 (33), 308 (100), 255 (58), 91 (15); **HRMS** (pos. APCI): calcd. for $C_{17}H_{23}ClNO_2$ $[M + NH_4]^+$: 308.14118; found: 308.14114

2-(4-(Benzyloxy)-3-isopropoxyphenyl)acetonitrile (9m)

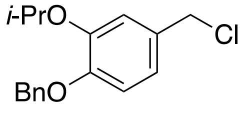

To a solution of 1-(benzyloxy)-4-(chloromethyl)-2-isopropoxybenzene (**8m**, 3.03 g, 10.4 mmol, 1 equiv.) in DMF (20 mL) were added NaI (0.80 g, 5.3 mmol, 0.51 equiv.) and KCN (0.76 g,

11.7 mmol, 1.13 equiv.). The resulting suspension was heated up to 80 °C and stirred at this temperature for 17 h. The reaction mixture was cooled down to room temperature and poured into H_2O (100 mL). The resulting suspension was extracted five times with EtOAc. The combined organic layers were dried over Na_2SO_4, filtered and concentrated under reduced pressure. The resulting red oil was purified on silica gel column eluting with 20-33% EtOAc in cyclohexane. The product was obtained as a yellow oil (2.18 g, 7.7 mmol, 74%). $\mathbf{R}_f$ = 0.42 (33% EtOAc in cyclohexane). **^{1}H NMR** (500 MHz, $CDCl_3$) δ 7.45 – 7.28 (m, 5H), 6.93 – 6.86 (m, 2H), 6.81 (dd, *J* = 8.3, 2.2 Hz, 1H), 5.12 (s, 2H), 4.55 (hept, *J* = 6.1 Hz, 1H), 3.66 (s, 2H), 1.37 (d, *J* = 6.1 Hz, 6H); **^{13}C NMR** (126 MHz, $CDCl_3$) δ 149.7, 148.8, 137.3, 128.6, 128.0, 127.4, 123.0, 121.1, 118.2, 117.2, 116.0, 72.5, 71.5, 23.3, 22.3; **MS** (pos. APCI), *m/z* (%): 300 (19), 299 (100), 65 (33); **HRMS** (pos. APCI): calcd. for $C_{18}H_{23}N_2O_2$ [M + $NH_4]^+$: 299.17540; found: 299.17545

1-(4-(Benzyloxy)-3-isopropoxyphenyl)cyclobutane-1-carbonitrile (10m)

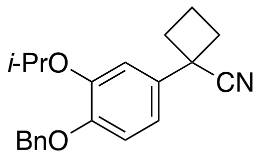

To a solution of 2-(4-(benzyloxy)-3-isopropoxyphenyl)acetonitrile (**9m**, 1.00 g, 3.6 mmol, 1 equiv.), 1,3-dibromopropane (0.36 mL, 3.6 mmol, 1.0 equiv.), and TBAB (12 mg, 0.04 mmol, 0.01 equiv.) in toluene (10 mL) were added KOH (85%, 1.6 g, 24 mmol, 6.8 equiv.) and H_2O (0.5 mL). The reaction mixture was heated up to reflux (approx. 120 °C) and stirred at this temperature for 1 h. The reaction mixture was cooled down to rt and was extracted three times with DCM. The combined organic layers were dried over Na_2SO_4, filtered and concentrated under reduced pressure. The residue was purified on silica gel column eluting with 11-20% EtOAc in cyclohexane. The product was obtained as a light-yellow solid (0.72 g, 2.2 mmol, 63%). $\mathbf{R}_f$ = 0.55 (33% EtOAc in cyclohexane); **m.p.**: 77 – 79 °C; **^{1}H NMR** (400 MHz, $CDCl_3$) δ 7.46 – 7.28 (m, 5H), 7.00 – 6.94 (m, 1H), 6.94 – 6.89 (m, 2H), 5.12 (s, 2H), 4.56 (hept, *J* = 6.1 Hz, 1H), 2.83 – 2.73 (m, 2H,), 2.64 – 2.51 (m, 2H), 2.47 – 2.31 (m, 1H), 2.15 – 1.95 (m, 1H), 1.36 (d, *J* = 6.1 Hz, 6H); **^{13}C NMR** (101 MHz, $CDCl_3$) δ 149.8, 148.5, 137.4, 133.2, 128.6, 128.0, 127.3, 124.7, 118.9, 115.7, 115.6, 72.7, 71.5, 40.0, 34.9, 22.4, 17.1; **MS** (pos. ESI, $CHCl_3$), *m/z* (%): 344 (46), 339 (100), 322 (25), 205 (80), 91 (17); **HRMS** (pos. ESI): calcd. for $C_{21}H_{27}N_2O_2$ [M + $NH_4]^+$: 339.20670; found: 339.20679

1-(4-(Benzyloxy)-5-isopropoxy-2-nitrophenyl)cyclobutane-1-carbonitrile (11m)

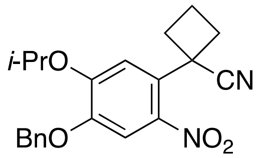

A solution of 1-(4-(benzyloxy)-3-isopropoxyphenyl)cyclobutane-1-carbonitrile (**10m**, 650 mg, 2.02 mmol, 1 equiv.) in Ac_2O (5 mL) and AcOH (4 mL) was cooled down to 0 °C and a solution of HNO_3 (0.13 mL, 3.0 mmol, 1.5 equiv.) in AcOH (1.7 mL) was added dropwise. The reaction mixture was stirred at 0 °C for 1 h and was poured into ice-water (100 mL). The resulting emulsion was allowed to warm up to rt. The layers were separated, and the aqueous layer was extracted three times with DCM. The combined organic layers were dried over Na_2SO_4, filtered and

concentrated under reduced pressure. The residue was purified by silica gel column eluting with 17-25% EtOAc in cyclohexane. The product was obtained as a yellow solid (678 mg, 1.85 mmol, 92%). **R*f*** = 0.41 (33% EtOAc in cyclohexane); **m.p.**: 114 – 116 °C; **^{1}H NMR** (400 MHz, $CDCl_3$) δ 7.65 (s, 1H), 7.49 – 7.30 (m, 5H), 6.78 (s, 1H), 5.18 (s, 2H), 4.68 (hept, *J* = 6.1 Hz, 1H), 3.05 – 2.83 (m, 2H), 2.54 – 2.35 (m, 3H), 1.99 – 1.82 (m, 1H), 1.41 (d, *J* = 6.1 Hz, 6H); **^{13}C NMR** (101 MHz, $CDCl_3$) δ 152.6, 148.8, 140.4, 135.9, 129.8, 128.9, 128.5, 127.5, 123.0, 114.7, 111.9, 73.0, 71.6, 39.2, 34.2, 22.2, 16.8; **MS** (pos. ESI), *m*/*z* (%): 390 (23), 389 (100), 91 (14); **HRMS** (pos. ESI): calcd. for $C_{21}H_{22}N_2O_4Na$ $[M + Na]^+$: 389.14718; found: 389.14713

1-(4-Hydroxy-5-isopropoxy-2-nitrophenyl)cyclobutane-1-carbonitrile (12m)

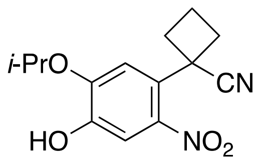

A solution of 1-(4-(benzyloxy)-5-isopropoxy-2-nitrophenyl)cyclobutane-1-carbonitrile (**11m**, 200 mg, 0.55 mmol, 1 equiv.) in DCM (25 mL) was cooled down to 0 °C. $AlCl_3$ (218 mg, 1.64 mmol, 3.00 equiv.) was added portionwise and the resulting suspension was stirred at 0 °C for 1 h. The reaction was quenched by addition of an aqueous solution of HCl (10 mL, 1M). The reaction mixture was allowed to warm up to rt and was extracted three times with DCM. The combined organic layers were dried over Na_2SO_4, filtered and concentrated under reduced pressure. The residue was purified by silica gel column eluting with 17-25% EtOAc in cyclohexane. The product was obtained as a yellow solid (121 mg, 0.44 mmol, 80%). **R*f*** = 0.41 (50% EtOAc in cyclohexane); **m.p.**: 128 – 130 °C; **^{1}H NMR** (400 MHz, $CDCl_3$) δ 7.63 (s, 1H), 6.72 (s, 1H), 5.85 (s, 1H), 4.72 (hept, *J* = 6.1 Hz, 1H), 2.95 – 2.86 (m, 2H), 2.51 – 2.44 (m, 3H), 1.97 – 1.91 (m, 1H), 1.44 (d, *J* = 6.1 Hz, 6H); **^{13}C NMR** (101 MHz, $CDCl_3$) δ 148.8, 146.2, 141.0, 128.5, 123.1, 112.3, 111.7, 73.1, 39.2, 34.3, 22.1, 16.8; **MS** (pos. ESI), *m*/*z* (%): 300 (14), 299 (100); **HRMS** (pos. ESI): calcd. for $C_{14}H_{16}N_2O_4Na$ $[M + Na]^+$: 299.10023; found: 299.10049

1-(5-Isopropoxy-2-nitro-4-(3-(pyrrolidin-1-yl)propoxy)phenyl)cyclobutane-1-carbonitrile (13m)

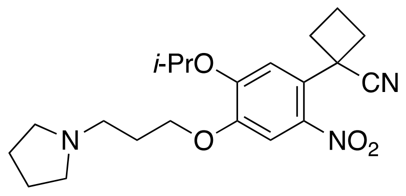

To a solution of 1-(4-hydroxy-5-isopropoxy-2-nitrophenyl)cyclobutane-1-carbonitrile (**12m**, 88 mg, 0.32 mmol, 1 equiv.) in DMF (2.1 mL) were added NaI (10 mg, 0.07 mmol, 0.20 equiv.), K_2CO_3 (62 mg, 0.45 mmol, 1.40 equiv.), and 1-(3-chloropropyl)pyrrolidine (**19**, 56 mg, 0.38 mmol, 1.19 equiv.). The resulting suspension was heated up to 80 °C and stirred at this temperature for 1 h. The reaction was quenched by addition of a saturated aqueous solution of NaCl (1 mL). The resulting suspension was extracted three times with DCM. The combined organic layers were dried over Na_2SO_4, filtered and concentrated under reduced pressure. The resulting orange oil was purified by silica gel column eluting with 5-10% MeOH in $CHCl_3$. The product was obtained as a yellow oil (113 mg, 0.29 mmol, 92%). **R*f*** = 0.20 (5% MeOH in $CHCl_3$); **^{1}H NMR** (500 MHz, $CDCl_3$) δ

7.61 (s, 1H), 6.75 (s, 1H), 4.64 (hept, *J* = 6.1 Hz, 1H), 4.13 (t, *J* = 6.3 Hz, 2H), 2.97 – 2.90 (m, 2H), 2.80 – 2.57 (m, 6H), 2.52 – 2.39 (m, 3H), 2.19 – 2.06 (m, 2H), 1.99 – 1.79 (m, 5H), 1.39 (d, *J* = 6.1 Hz, 6H); **^{13}C NMR** (126 MHz, $CDCl_3$) δ 152.2, 149.2, 140.5, 129.4, 123.1, 114.8, 111.0, 72.9, 68.0, 54.3, 53.0, 39.2, 34.2, 28.2, 23.6, 22.2, 16.8; **MS** (pos. ESI), *m/z* (%): 389 (23), 388 (100); **HRMS** (pos. ESI): calcd. for $C_{21}H_{30}N_3O_4$ $[M + H]^+$, calcd: 388.22308; found: 388.22302

5'-Isopropoxy-6'-(3-(pyrrolidin-1-yl)propoxy)spiro[cyclobutane-1,3'-indol]-2'-amine (1m)

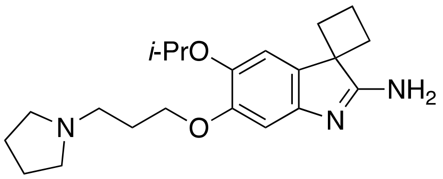

To a solution of 1-(5-isopropoxy-2-nitro-4-(3-(pyrrolidin-1-yl)propoxy)phenyl)cyclobutane-1-carbonitrile (**13m**, 60 mg, 0.16 mmol, 1 equiv.) in AcOH (0.55 mL, 9.6 mmol, 60.0 equiv.) was added zinc dust (152 mg, 2.32 mmol, 14.5 equiv.). The reaction mixture was heated up to 85 °C and was stirred at that temperature for 1 h. The solids were filtered and washed with AcOH. The filtrate was concentrated under reduced pressure. The residue was dissolved in EtOAc and the resulting solution was washed with a saturated aqueous solution of $NaHCO_3$ (5 mL). The layers were separated, and the aqueous layer was extracted three times with EtOAc. The combined organic layers were dried over Na_2SO_4, filtered and concentrated under reduced pressure. The product was obtained as a light yellow gum (55 mg, 0.15 mmol, 99%). **^{1}H NMR** (400 MHz, $CDCl_3$) δ 7.07 (s, 1H), 6.75 (s, 1H), 4.33 (hept, *J* = 6.1 Hz, 1H), 4.02 (t, *J* = 6.4 Hz, 2H), 2.68 – 2.58 (m, 2H), 2.57 – 2.41 (m, 8H), 2.40 – 2.26 (m, 1H), 2.22 – 2.06 (m, 1H), 2.08 – 1.96 (m, 2H), 1.82 – 1.74 (m, 4H), 1.31 (d, *J* = 6.1 Hz, 6H); **^{13}C NMR** (101 MHz, $CDCl_3$) δ 178.0, 151.6, 143.0, 131.5, 130.2, 114.8, 102.7, 74.3, 67.8, 54.4, 53.5, 52.6, 31.9, 29.2, 23.6, 22.6, 16.9; **MS** (pos. ESI), *m/z* (%): 358 (92), 176 (96), 144 (100), 112 (50), 84 (67); **HRMS** (pos. ESI): calcd. for $C_{21}H_{32}N_3O_2$ $[M + H]^+$: 358.24890; found: 358.24896

15. 5'-Methoxy-6'-(3-(pyrrolidin-1-yl)propoxy)spiro[cyclohexane-1,3'-indol]-2'-amine (1n)

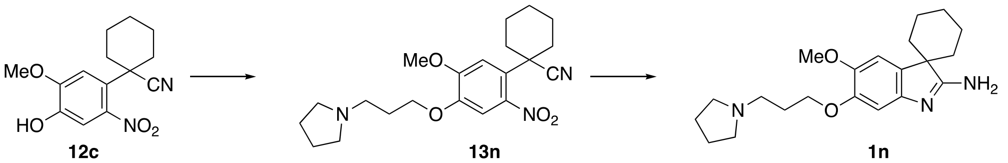

1-(5-Methoxy-2-nitro-4-(3-(pyrrolidin-1-yl)propoxy)phenyl)cyclohexane-1-carbonitrile (13n)

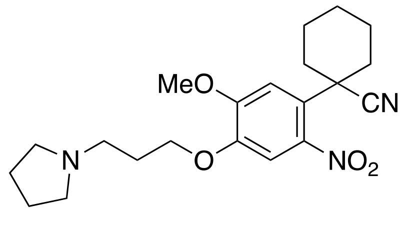

To a solution of 1-(4-hydroxy-5-methoxy-2-nitrophenyl)cyclohexane-1-carbonitrile (**12c**, 160 mg, 0.55 mmol, 1 equiv.) in DMF (3.7 mL) were added NaI (16 mg, 0.11 mmol, 0.20 equiv.), K_2CO_3 (106 mg, 0.76 mmol, 1.38 equiv.), and 1-(3-chloropropyl)pyrrolidine (**19**, 97 mg, 0.65 mmol, 1.18 equiv.). The resulting suspension was heated up to 80 °C and stirred at this temperature for 1.5 h. The reaction was quenched by addition of a saturated aqueous solution of NaCl (1.5 mL). The resulting suspension was extracted three times with DCM. The combined organic layers were dried over Na_2SO_4, filtered and concentrated under reduced pressure. The resulting orange oil was purified by silica gel column eluting with 5-10% MeOH in $CHCl_3$. The product was obtained as a yellow solid (193 mg, 0.50 mmol, 91%). **R*f*** = 0.11 (5% MeOH in $CHCl_3$); **m.p.**: 111 – 113 °C; **^{1}H NMR** (400 MHz, $CDCl_3$) δ 7.36 (s, 1H), 7.05 (s, 1H), 4.14 (t, *J* = 6.5 Hz, 2H), 3.95 (s, 3H), 2.72 (t, *J* = 7.3 Hz, 2H), 2.68 – 2.59 (m, 4H), 2.42 – 2.32 (m, 2H), 2.12 (dt, *J* = 6.8 Hz, 2H), 1.93 – 1.78 (m, 11H), 1.38 – 1.20 (m, 1H); **^{13}C NMR** (101 MHz, $CDCl_3$) δ 152.3, 147.9, 143.2, 127.3, 120.7, 110.8, 110.4, 68.0, 56.6, 54.3, 52.8, 43.3, 36.3, 28.2, 24.9, 23.6, 23.6; **MS** (pos. ESI), *m/z* (%): 389 (21), 388 (100), 151 (12); **HRMS** (pos. ESI): calcd. for $C_{21}H_{30}N_3O_4$ $[M + H]^+$: 388.22308; found: 388.22314

5'-Methoxy-6'-(3-(pyrrolidin-1-yl)propoxy)spiro[cyclohexane-1,3'-indol]-2'-amine (1n)

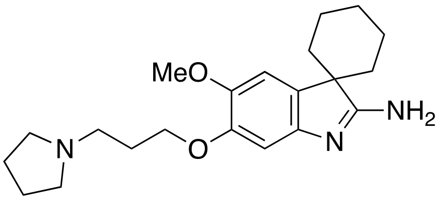

To a solution of 1-(5-methoxy-2-nitro-4-(3-(pyrrolidin-1-yl)propoxy)phenyl)cyclohexane-1-carbonitrile (**13n**, 60 mg, 0.16 mmol, 1 equiv.) in AcOH (0.45 mL, 7.9 mmol, 50.0 equiv.) was added zinc dust (101 mg, 1.55 mmol, 9.69 equiv.). The reaction mixture was heated up to 80 °C and was stirred at that temperature for 1.5 h. The solids were filtered and washed with AcOH. The filtrate was concentrated under reduced pressure. The residue was dissolved in EtOAc and the resulting solution was washed with a saturated aqueous solution of $NaHCO_3$ (5 mL). The layers were separated, and the aqueous layer was extracted three times with EtOAc. The combined organic layers were dried over Na_2SO_4, filtered and concentrated under reduced pressure. The product was obtained as a light yellow gum (50 mg, 0.14 mmol, 90%). **^{1}H NMR** (400 MHz, $CDCl_3$) δ 7.12 (s, 1H), 6.86 (s, 1H), 4.07 (t, *J* = 6.6 Hz, 2H), 3.85 (s, 3H), 2.62 (t, *J* = 7.1 Hz, 2H), 2.55 – 2.46 (m, 4H), 2.12 – 1.96 (m, 2H), 1.95 – 1.81 (m, 3H), 1.80 – 1.71 (m, 6H), 1.60 – 1.56 (m, 4H), 1.49 – 1.40 (m, 1H); **^{13}C NMR** (101 MHz, $CDCl_3$) δ 179.5, 149.3, 144.2, 131.9, 122.9, 111.5, 103.1, 67.8), 58.2, 54.3, 53.3, 52.8, 34.0, 29.0, 25.3, 23.6, 21.7; **MS** (pos. APCI), *m*/*z* (%): 359 (23), 358 (100), 222 (11); **HRMS** (pos. APCI): calcd. for $C_{21}H_{32}N_3O_2$ $[M + H]^+$: 358.24890; found: 358.24915

16. 3,3-Diethyl-5-methoxy-6-(3-(pyrrolidin-1-yl)propoxy)-3*H*-indol-2-amine (1o)

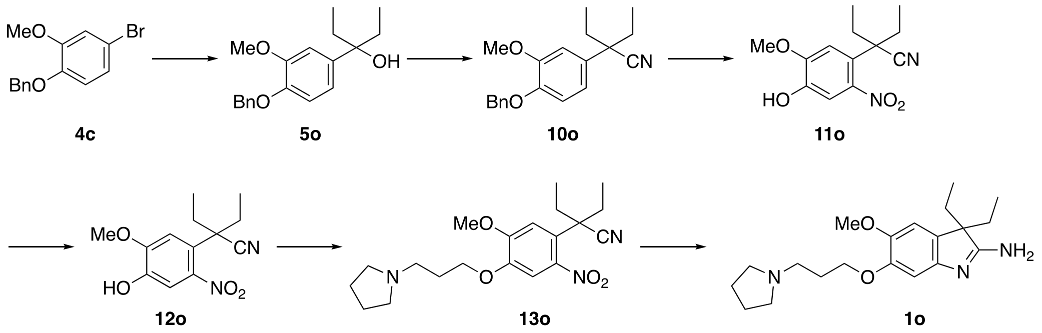

3-(4-(Benzyloxy)-3-methoxyphenyl)pentan-3-ol (5o)

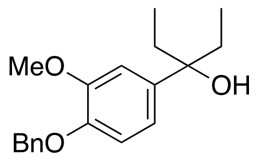

A solution of 1-(benzyloxy)-4-bromo-2-methoxybenzene (**4c**, 2.00 g, 6.8 mmol, 1 equiv.) in THF (20 mL) was cooled down to −78 °C and a solution of *n*-BuLi in hexanes (1.6 M, 4.70 mL, 7.5 mmol, 1.1 equiv.) was added dropwise. The reaction mixture was stirred at −78 °C for 20 min and a solution of 3-pentanone (0.80 mL, 7.5 mmol, 1.1 equiv.) in THF (6 mL) was added dropwise. The resulting solution was stirred at −78 °C for 5 min and was afterwards allowed to warm up to rt. The reaction was quenched by addition of a saturated aqueous solution of NH_4Cl (10 mL). The reaction mixture was extracted three times with DCM. The combined organic layers were dried over Na_2SO_4, filtered and concentrated under reduced pressure. The residue was purified on silica gel column eluting with 17-33% EtOAc in cyclohexane. The product was obtained as a light yellow oil (1.27 mg, 4.2 mmol, 62%); $\mathbf{R}_f$ = 0.25 (20% EtOAc in cyclohexane); **^{1}H NMR** (400 MHz, $CDCl_3$) δ 7.48 – 7.28 (m, 5H), 7.00 (d, *J* = 2.1 Hz, 1H), 6.86 (d, *J* = 8.4 Hz, 1H), 6.80 (dd, *J* = 8.4, 2.1 Hz, 1H), 5.14 (s, 2H), 3.90 (s, 3H), 1.88 – 1.72 (m, 4H), 1.60 (s, 1H), 0.77 (t, *J* = 7.4 Hz, 6H); **^{13}C NMR** (101 MHz, $CDCl_3$) δ 149.5, 146.9, 139.4, 137.6, 128.6, 127.9, 127.5, 117.8, 113.8, 110.2, 77.4, 71.3, 56.3, 35.0, 8.0; **MS** (pos. APCI), *m*/*z* (%): 318 (60), 284 (20), 283 (100); **HRMS** (pos. APCI): calcd. for $C_{19}H_{28}NO_3$ [M + NH_4]$^+$: 318.20637; found: 318.20645

2-(4-(Benzyloxy)-3-methoxyphenyl)-2-ethylbutanenitrile (10o)

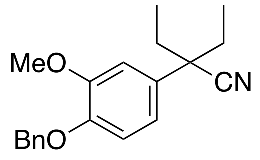

A solution of 3-(4-(benzyloxy)-3-methoxyphenyl)pentan-3-ol (**5o**, 1.20 g, 4.0 mmol, 1 equiv.) in DCM (30 mL) was cooled down to −78 °C and TMSCN (1.50 mL, 12.0 mmol, 3.0 equiv.) was added dropwise. The reaction mixture was stirred at −78 °C for 5 min. Then, a solution of $SnCl_4$ in DCM (1.0 M, 4.0 mL, 4.0 mmol, 1.0 equiv.) was added dropwise and the reaction mixture was stirred at −78 °C for 5 min. The reaction mixture was poured into a saturated aqueous solution of $NaHCO_3$. The resulting emulsion was stirred at rt for 30 min and was afterwards extracted three times with DCM. The combined organic layers were dried over Na_2SO_4, filtered and concentrated under

reduced pressure. The residue was purified on silica gel column eluting with 11-33% EtOAc in cyclohexane. The product was obtained as a light yellow oil (1.06 g, 3.4 mmol, 86%). **R***f* = 0.37 (`20% EtOAc in cyclohexane); **¹H NMR** (400 MHz, $CDCl_3$) δ 7.47 – 7.28 (m, 5H), 6.91 – 6.87 (m, 2H), 6.85 (dd, *J* = 8.4, 2.1 Hz, 1H), 5.15 (s, 2H), 3.91 (s, 3H), 2.08 – 1.78 (m, 4H), 0.92 (t, *J* = 7.3 Hz, 6H); **¹³C NMR** (101 MHz, $CDCl_3$) δ 149.9, 147.8, 137.2, 131.3, 128.7, 128.0, 127.5, 122.6, 118.6, 114.2, 110.4, 71.3, 56.4, 49.6, 34.0, 9.9; **MS** (pos. APCI), *m*/*z* (%): 328 (21), 327 (100), 310 (17); **HRMS** (pos. APCI): calcd. for $C_{20}H_{27}N_2O_2$ $[M + NH_4]^+$: 327.20670; found: 327.20660

2-(4-(Benzyloxy)-5-methoxy-2-nitrophenyl)-2-ethylbutanenitrile (11o)

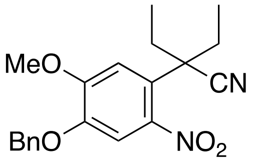

A solution of 2-(4-(benzyloxy)-3-methoxyphenyl)-2-ethylbutanenitrile (**10o**, 1.00 mg, 3.2 mmol,1 equiv.) in Ac_2O (8 mL) and AcOH (6 mL) was cooled down to 0 °C and a solution of HNO_3 (0.20 mL, 4.9 mmol, 1.5 equiv.) in AcOH (2.7 mL) was added dropwise. The reaction mixture was stirred at 0 °C for 1 h and was poured into ice-water (50 mL). The resulting emulsion was allowed to warm up to rt. The layers were separated, and the aqueous layer was extracted three times with DCM. The combined organic layers were dried over Na_2SO_4, filtered and concentrated under reduced pressure. The residue was purified by column chromatography eluting with 17-33% EtOAc in cyclohexane. After recrystallization from EtOAc/pentane (4:1), the product was obtained as a yellow solid (720 mg, 2.0 mmol, 63%). **R***f* = 0.45 (33% EtOAc in cyclohexane); **m.p.**: 139 – 141 °C; **¹H NMR** (400 MHz, $CDCl_3$) δ 7.47 – 7.32 (m, 5H), 7.21 (s, 1H), 7.16 (s, 1H), 5.14 (s, 2H), 3.98 (s, 3H), 2.29 – 1.97 (m, 4H), 1.01 (t, *J* = 7.4 Hz, 6H); **¹³C NMR** (101 MHz, $CDCl_3$) δ 151.8, 147.6, 143.0, 135.6, 128.9, 128.6, 127.7, 123.2, 122.0, 113.6, 111.1, 71.6, 56.6, 50.8, 31.8, 10.4; **MS** (pos. ESI), *m*/*z* (%): 378 (20), 377 (100), 91 (14); **HRMS** (pos. ESI): calcd. for $C_{20}H_{22}N_2O_4Na$ $[M + Na]^+$: 377.14718; found: 377.14737

2-Ethyl-2-(4-hydroxy-5-methoxy-2-nitrophenyl)butanenitrile (12o)

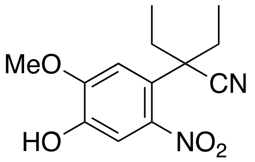

A solution of 2-(4-(benzyloxy)-5-methoxy-2-nitrophenyl)-2-ethylbutanenitrile (**11o**, 200 mg, 0.56 mmol, 1 equiv.) in DCM (25 mL) was cooled down to 0 °C. $AlCl_3$ (226 mg, 1.69 mmol, 3.01 equiv.) was added portionwise and the resulting suspension was stirred at 0 °C for 1 h. The reaction was quenched by addition of an aqueous solution of HCl (10 mL, 1M). The reaction mixture was allowed to warm up to rt and was extracted three times with DCM. The combined organic layers were dried over Na_2SO_4, filtered and concentrated under reduced pressure. The residue was purified on silica gel eluting with 17-25% EtOAc in cyclohexane. The product was obtained as a yellow solid (140 mg, 0.53 mmol, 94%). **R***f* = 0.43 (50% EtOAc In cyclohexane); **m.p.**:

122 – 124 °C; **^{1}H NMR** (400 MHz, $CDCl_3$) δ 7.19 (s, 1H), 7.15 (s, 1H), 5.85 (s, 1H), 4.01 (s, 3H), 2.25 – 1.98 (m, 4H), 1.00 (t, *J* = 7.4 Hz, 6H); **^{13}C NMR** (101 MHz, $CDCl_3$) δ 148.5, 145.3, 143.7, 122.2, 121.7, 112.7, 112.3, 56.6, 51.0, 31.9, 10.4; **MS** (pos. ESI), *m*/*z* (%): 300 (23), 287 (100); **HRMS** (pos. ESI): calcd. for $C_{13}H_{16}N_2O_4Na$ $[M + Na]^+$: 287.10023; found: 287.10034.

2-Ethyl-2-(5-methoxy-2-nitro-4-(3-(pyrrolidin-1-yl)propoxy)phenyl)butanenitrile (13o)

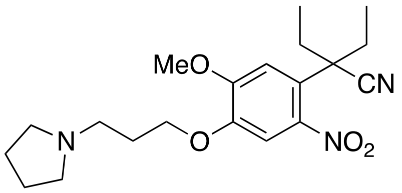

To a solution of 2-ethyl-2-(4-hydroxy-5-methoxy-2-nitrophenyl)butanenitrile (**12o**, 90 mg, 0.34 mmol, 1 equiv.) in DMF (2.3 mL) were added NaI (10 mg, 0.07 mmol, 0.21 equiv.), K_2CO_3 (66 mg, 0.48 mmol, 1.41 equiv.), and 1-(3-chloropropyl)pyrrolidine (**19**, 60 mg, 0.41 mmol, 1.21 equiv.). The resulting suspension was heated up to 80 °C and stirred at this temperature for 1.5 h. The reaction was quenched by addition of a saturated aqueous solution of NaCl (1.0 mL). The resulting suspension was extracted three times with DCM. The combined organic layers were dried over Na_2SO_4, filtered and concentrated under reduced pressure. The resulting orange oil was purified by silica gel column eluting with 5-10% MeOH in $CHCl_3$. The product was obtained as a yellow gum (125 mg, 0.33 mmol, 98%). **R_f** = 0.13 (5% MeOH in $CHCl_3$); **^{1}H NMR** (500 MHz, $CDCl_3$) δ 7.19 (s, 1H), 7.12 (s, 1H), 4.13 (t, *J* = 6.4 Hz, 2H), 3.95 (s, 3H), 2.80 – 2.62 (m, 6H), 2.25 – 2.02 (m, 6H), 1.91 – 1.82 (m, 4H), 0.99 (t, *J* = 7.4 Hz, 6H); **^{13}C NMR** (126 MHz, $CDCl_3$) δ 151.4, 147.8, 143.1, 122.7, 122.1, 113.4, 110.4, 67.9, 56.5, 54.3, 52.9, 50.8, 31.8, 28.0, 23.6, 10.4; **MS** (pos. APCI), *m*/*z* (%): 377 (21), 376 (100); **HRMS** (pos. APCI): calcd. for $C_{20}H_{30}N_3O_4$ $[M + H]^+$: 376.22308; found: 376.22275

3,3-Diethyl-5-methoxy-6-(3-(pyrrolidin-1-yl)propoxy)-3*H*-indol-2-amine (1o)

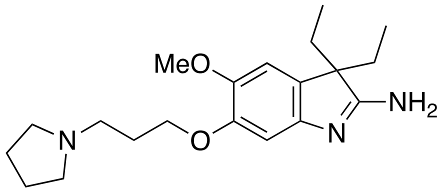

To a solution of 2-ethyl-2-(5-methoxy-2-nitro-4-(3-(pyrrolidin-1-yl)propoxy)phenyl)butanenitrile (**13o**, 77 mg, 0.21 mmol, 1 equiv.) in AcOH (0.70 mL, 12.2 mmol, 58.1 equiv.) was added zinc dust (201 mg, 3.08 mmol, 15.02 equiv.). The reaction mixture was heated up to 80 °C and was stirred at that temperature for 1.5 h. The solids were filtered and washed with AcOH. The filtrate was concentrated under reduced pressure. The residue was dissolved in EtOAc and the resulting solution was washed with a saturated aqueous solution of $NaHCO_3$ (5 mL). The layers were separated, and the aqueous layer was extracted three time with EtOAc. The combined organic layers were dried over Na_2SO_4, filtered and concentrated under reduced pressure. The product was obtained as a light yellow gum (46 mg, 0.13 mmol, 65%). **^{1}H NMR** (500 MHz, $CDCl_3$) δ 6.83 (s, 1H), 6.62 (s, 1H), 4.08 (t, *J* = 6.7 Hz, 2H), 3.83 (s, 3H), 2.66 – 2.59 (m, 2H), 2.54 – 2.47 (m, 4H), 2.08 – 2.00 (m, 2H), 1.88 – 1.73 (m, 6H), 1.63 – 1.55 (m, 2H), 0.52 (t, *J* = 7.4 Hz, 6H); **^{13}C NMR** (126 MHz, $CDCl_3$) δ 176.5, 148.9, 145.2, 142.3, 128.6, 107.7, 103.0, 67.8, 59.2, 57.6, 54.4, 53.4, 31.3, 29.1, 23.6, 8.5;

MS (pos. APCI), *m*/*z* (%): 347 (21), 346 (100); **HRMS** (pos. APCI): calcd for $C_{20}H_{32}N_3O_2$ [M + H]$^+$: 346.24890; found: 346.24896

17. 5-Methoxy-3,3-dimethyl-6-(3-(pyrrolidin-1-yl)propoxy)-3*H*-indol-2-amine (1p)

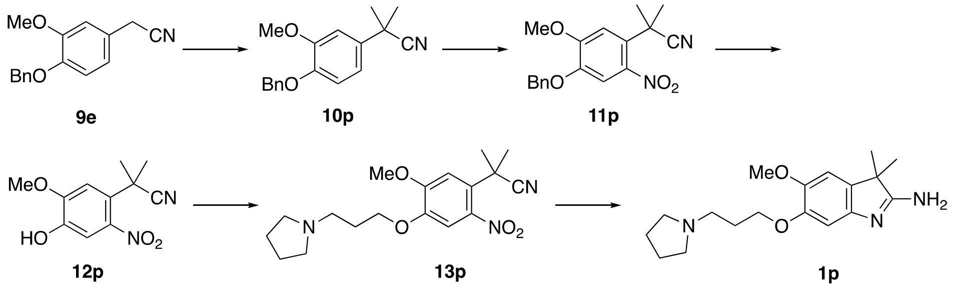

2-(4-(Benzyloxy)-3-methoxyphenyl)-2-methylpropanenitrile (10p)

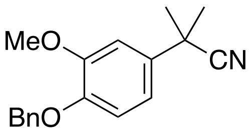

To a solution of 2-(4-(benzyloxy)-3-methoxyphenyl)acetonitrile (**9e**, 480 mg, 1.90 mmol, 1 equiv.) and NaH (60% in oil) (189 mg, 4.74 mmol, 2.5 equiv.) in THF was added CH_3I (474 µL, 7.58 mmol, 4 equiv.). The mixture was heated to reflux for 16 h. The reaction was then evaporated, water was added and the residue was extracted three times with EtOAc. The organic phase was washed with brine, dried over Na_2SO_4 and evaporated under reduced pressure. Purification on silica gel eluting with 10-30% EtOAc in cyclohexane (482 mg, 1.90 mmol, 90%). **R***f* = 0.28 (25% EtOAc in cyclohexane); **^{1}H NMR** (400 MHz, MeOD-*d*$_4$) δ 7.45-7.24 (m, 5H), 7.0 (d, *J* = 2.3 Hz, 1H), 6.91 (dd, *J* = 8.4, 2.3 Hz, 1H), 6.86 (d, *J* = 8.4L, Hz, 1H), 5.13 (s, 2H), 3.90 (s, 3H), 1.68 (s, 6H); **^{13}C NMR** (101 MHz, MeOD-*d*$_4$) δ 150.9, 148.3, 142.8, 139.0, 129.6, 129.0, 128.8, 118.8, 115.7, 111.7, 84.1, 72.6, 56.8, 42.6, 24.9; **HRMS** (pos. ESI): calcd. for $C_{18}H_{19}O_2NNa$ [M+Na]$^+$: 304.13135, found: 304.13080

2-(4-(Benzyloxy)-5-methoxy-2-nitrophenyl)-2-methylpropanenitrile (11p)

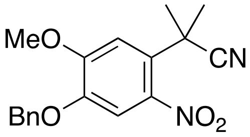

The compound was synthetized following the same procedure used for the synthesis of 2-(4-(benzyloxy)-5-methoxy-2-nitrophenyl)-2-methylbutanenitrile (**11r**) starting from 2-(4-(benzyloxy)-3-methoxyphenyl)-2-methylpropanenitrile (**10p**). The residue was purified on silica gel eluting with 10-30% EtOAc in cyclohexane to afford a yellow oil (387 mg, 1.19 mmol, **73%**). **R***f* = 0.40 (30% EtOAc in cyclohexane); **m.p.**: 146 – 148 °C; **^{1}H NMR** (400 MHz, $CDCl_3$) δ 7.44-7.28 (m, 6H), 7.04 (s, 1H), 5.14 (s, 2H), 3.95 (s, 3H), 1.86 (s, 6H); **^{13}C NMR** (101 MHz, $CDCl_3$) δ 152.8, 147.6, 142.4, 135.6, 128.9, 128.6, 128.0, 127.7, 123.1, 111.3, 110.4, 71.6, 56.7, 36.5, 28.6; **HRMS** (pos. ESI): calcd. for $C_{18}H_{18}N_2O_4Na$ [M+Na]$^+$: 349.11643, found: 349.1159

2-(4-Hydroxy-5-methoxy-2-nitrophenyl)-2-methylpropanenitrile (12p)

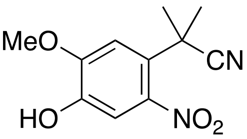

The compound was synthetized following general procedure for benzyl deprotection starting from 2-(4-(benzyloxy)-5-methoxy-2-nitrophenyl)-2-methylpropanenitrile (**11p**) in DCM (50 mL). The

residue was purified on silica gel eluting with 20-50% EtOAc in cyclohexane to afford a yellow oil (302 mg, 1.21 mmol, **82%**). **^{1}H NMR** (400 MHz, $CDCl_3$) δ 7.33 (s, 1H), 7.05 (s, 1H), 5.83 (br s, 1H), 3.99 (s, 3H), 1.86 (s, 6H); **^{13}C NMR** (101 MHz, $CDCl_3$) δ 149.4, 145.4, 143.3, 126.6, 123.4, 112.7, 109.6, 56.7, 36.9, 28.5; **HRMS** (pos. APCI): calcd. for $C_{11}H_{13}N_2O_4$ $[M+H]^+$: 237.08753, found: 237.080

2-(5-Methoxy-2-nitro-4-(3-(pyrrolidin-1-yl)propoxy)phenyl)-2-methylpropanenitrile (13p)

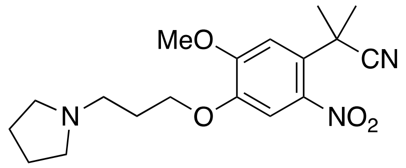

To a solution of 2-(4-hydroxy-5-methoxy-2-nitrophenyl)-2-methylpropanenitrile (**12p**, 100 mg, 0.42 mmol, 1 equiv.) in DMF (2.8 mL) were added 1-(3-chloropropyl)pyrrolidine (**19**, 75 mg, 0.51 mmol, 1.2 equiv.), K_2CO_3 (82 mg, 0.59 mmol, 1.4 equiv.) and NaI (13 mg, 0.09 mmol, 0.2 equiv.). The mixture was heated at 80 °C for 1 h. Water wad added, and the aqueous phase was extracted three times with EtOAc. The combined organic phase was washed with brine, dried over Na_2SO_4 and concentrated under reduced pressure. The residue was purified on silica gel column eluting with 0-10% MeOH in DCM afforded the desired product (112 mg, 0.32 mmol, **76%**) as a light-yellow oil. **R_f** = 0.37 (10% MeOH in DCM); **^{1}H NMR** (400 MHz, $CDCl_3$) δ 7.35 (s, 1H), 7.02 (s, 1H), 4.13 (t, *J* = 6.5 Hz, 2H), 3.93 (s, 3H), 2.73 (t, *J* = 7.1 Hz, 2H), 2.71 – 2.59 (m, 4H), 2.17 – 2.07 (m, 2H), 1.86 (s, 3H), 1.87 – 1.82 (m, 4H), 1.86 (s, 6H); **^{13}C NMR** (101 MHz, $CDCl_3$) δ 152.5, 148.0, 142.6, 127.6, 123.3, 110.8, 110.5, 68.0, 56.7, 54.3, 52.9, 36.7, 28.6, 28.1, 23.7; **HRMS** (pos. ESI): calcd. for $C_{18}H_{26}N_3O_4$ $[M+H]^+$: 348.19233, found: 348.1918

5-Methoxy-3,3-dimethyl-6-(3-(pyrrolidin-1-yl)propoxy)-3*H*-indol-2-amine (1p)

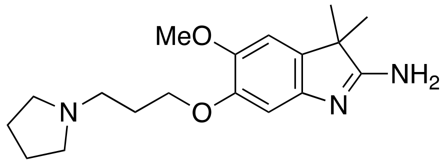

To a solution of 2-(5-methoxy-2-nitro-4-(3-(pyrrolidin-1-yl)propoxy)phenyl)-2-methylpropanenitrile (**13p**, 150 mg, 0.43 mmol, 1 equiv.) in AcOH (1.48 mL) was added zinc dust (423 mg, 6.48 mmol, 15 equiv.). The reaction mixture was heated up to 80 °C and was stirred at that temperature for 1.5 h. The solids were filtered and washed with AcOH. The filtrate was concentrated under reduced pressure. The residue was dissolved using EtOAc and the resulting solution was washed with an aqueous solution of NaOH (5 mL, 1M). The layers were separated, and the aqueous layer was extracted three times with EtOAc. The combined organic layers were dried over Na_2SO_4, filtered and concentrated under reduced pressure. The product was obtained as a light-yellow gum (95 mg, 0.30 mmol, **69%**). **^{1}H NMR** (400 MHz, $CDCl_3$) δ 6.93 (br s, 1H), 6.69 (s, 1H), 4.62 (br s, 2H), 4.05 (t, *J* = 6.6 Hz, 2H), 3.81 (s, 3H), 2.70 – 2.62 (m, 2H), 2.58 – 2.51 (m, 4H), 2.07 – 1.98 (m, 2H), 1.81 – 1.74 (m, 4H), 1.33 (s, 6H); **^{13}C NMR** (101 MHz, $CDCl_3$) δ 180.6, 149.0, 145.6, 131.7, 128.5, 107.3, 102.7, 68.0, 57.5, 54.3, 53.2, 48.8, 28.7, 25.3, 23.6; **HRMS** (pos. ESI): calcd. for $C_{18}H_{28}N_3O_2$ $[M+H]^+$: 318.21815, found: 318.2176

i

18. (*R*)-1-(3-((2'-amino-5'-methoxyspiro[cyclopentane-1,3'-indol]-6'-yl)oxy)propyl)pyrrolidin-3-ol (1q)

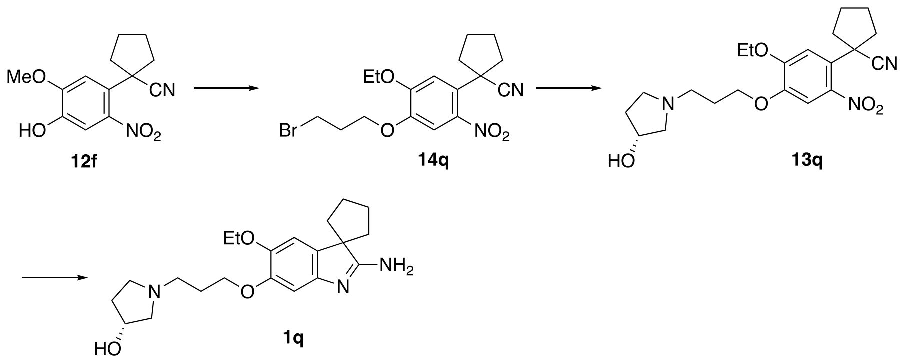

1-(4-(3-Bromopropoxy)-5-methoxy-2-nitrophenyl)cyclopentane-1-carbonitrile (14q)

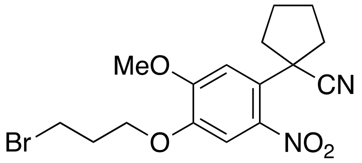

To a solution of 1-(4-hydroxy-5-methoxy-2-nitrophenyl)cyclopentane-1-carbonitrile (**12f**, 700 mg, 2.67 mmol, 1 equiv.) in DMF (10 mL) at rt were added K_2CO_3 (553 mg, 4.00 mmol, 1.5 equiv.) and 1,3-dibromopropane (544 µL, 5.34 mmol, 2 equiv.). The mixture was allowed to stir at rt for 16 h. Water was added, and the aqueous phase was extracted three times with EtOAc. The combined organic phase was washed with brine, dried over Na_2SO_4 and evaporated. Purification on silica gel column eluting with 10-30% EtOAc in cyclohexane afforded the desired product (775 mg, 2.02 mmol, **76%**) as a yellow solid. **R_f** = 0.40 (30% EtOAc in cyclohexane); **m.p.**: 88 – 90 °C; **^{1}H NMR** (400 MHz, $CDCl_3$) δ 7.45 (s, 1H), 6.95 (s, 1H), 4.18 (t, *J* = 5.9 Hz, 2H), 3.92 (s, 3H), 3.59 (t, *J* = 6.3 Hz, 2H), 2.71-2.62 (m, 2H), 2.36 (quin, *J* = 6.1 Hz, 6.1 Hz), 2.07-1.96 (m, 4H), 1.93-1.81 (m, 2H); **^{13}C NMR** (101 MHz, $CDCl_3$) δ 152.6, 147.7, 142.7, 127.7, 122.9, 111.1, 110.8, 67.2, 56.6, 45.6, 39.1, 32.1, 29.7, 24.0; **HRMS** (pos. ESI): calcd. for $C_{16}H_{19}BrN_2O_4Na$ $[M+Na]^+$: 405.04259, found: 405.0420

(*R*)-1-(4-(3-(3-hydroxypyrrolidin-1-yl)propoxy)-5-methoxy-2-nitrophenyl)cyclopentane-1-carbonitrile (13q)

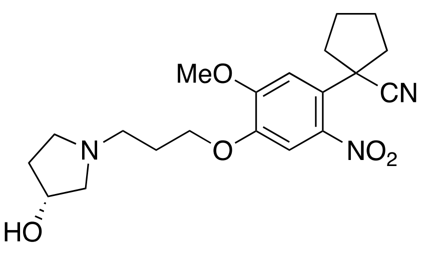

To a 1-(4-(3-bromopropoxy)-5-methoxy-2-nitrophenyl)cyclopentane-1-carbonitrile (**14q**, 100 mg, 0.26 mmol, 1 equiv.) in DMF (5 mL), were added (*R*)-pyrrolidin-3-ol (57 mg, 0.65 mmol, 2.5 equiv.) and K_2CO_3 (72 mg, 0.52 mmol, 2 equiv.). The mixture as stirred at rt for 16 h, then 1 h at 70 °C. The solution was evaporated ad the residue was dissolved in EtOAc and water. The organic phase was washed with brine and dried over Na_2SO_4, evaporated and purified on silica gel column (0-10% MeOH in DCM + 0.2% NEt_3) to afford the desired product (62 mg, 0.16 mmol, 61%) as a colorless

oil. **R**$_f$ = 0.34 (4% MeOH in DCM + 0.2% NEt_3); $[\alpha]_D^{20}$ = +19.6 °(*c* 0.25, MeOH); **^{1}H NMR** (400 MHz, $CDCl_3$) δ 7.45 (s, 1H), 6.92 (s, 1H), 4.45 – 4.40 (m, 1H), 4.14 (t, *J* = 6.3 Hz, 2H), 3.91 (s, 3H), 3.17 – 3.10 (m, 1H), 2.99 – 2.94 (m, 1H), 2.94 – 2.83 (m, 3H); **^{13}C NMR** (101 MHz, $CDCl_3$) δ 152.6, 147.8, 142.7, 127.5, 123.0, 110.9, 110.8, 70.7, 67.6, 62.7, 56.6, 53.0, 52.9, 45.5, 39.1, 34.6, 27.3, 24.0; **HRMS** (pos. ESI): calcd. for $C_{20}H_{28}N_3O_5$ [M+H]$^+$: 390.2024, found: 390.2023

(*R*)-1-(3-((2'-amino-5'-methoxyspiro[cyclopentane-1,3'-indol]-6'-yl)oxy)propyl)pyrrolidin-3-ol (1q)

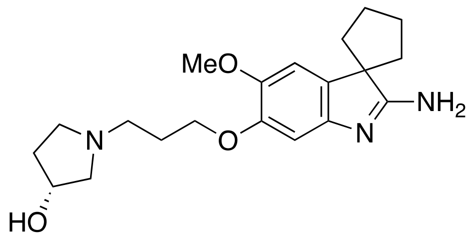

To a solution of (*R*)-1-(4-(3-(3-hydroxypyrrolidin-1-yl)propoxy)-5-methoxy-2-nitrophenyl)cyclopentane-1-carbonitrile (**13q**, 40 mg, 0.10 mmol, 1 equiv.) in AcOH (0.7 mL) was added zinc dust (101 mg, 1.54 mmol, 15 equiv.). The reaction mixture was heated up to 80 °C and was stirred at that temperature for 1.5 h. The solids were filtered and washed with AcOH. The filtrate was concentrated under reduced pressure. The residue was dissolved using EtOAc and an aqueous solution of NaOH (10 mL, 2M). The layers were separated, and the aqueous layer was extracted three times with EtOAc. The combined organic layers were dried over Na_2SO_4, filtered and concentrated under reduced pressure. The product was obtained as a light yellow solid (21 mg, 0.06 mmol, **57%**). **HRMS** (pos. ESI): calcd. for $C_{20}H_{28}N_3O_3$ [M+H]$^+$: 360.2282, found: 360.2282

19. <u>3-Ethyl-5-methoxy-3-methyl-6-(3-(pyrrolidin-1-yl)propoxy)-3*H*-indol-2-amine (1r)</u>

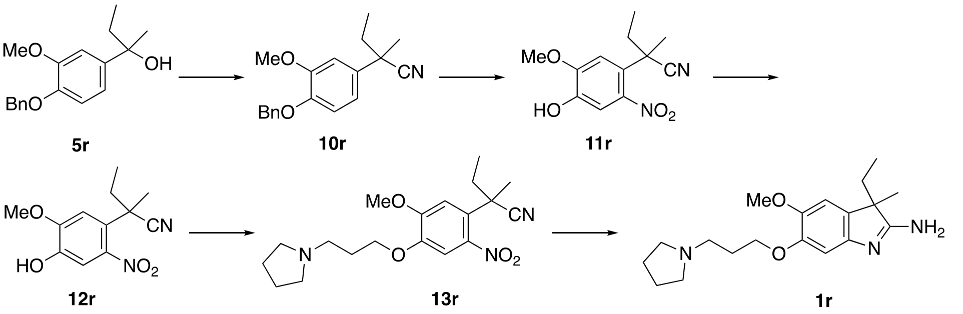

2-(4-(Benzyloxy)-3-methoxyphenyl)butan-2-ol (5r)

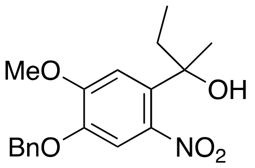

The compound was synthetized following the procedure used for 3-(4-(benzyloxy)-3-methoxyphenyl)pentan-3-ol (**5o**). Purification on silica gel column eluting with 10-30% EtOAc in cyclohexane afforded the desired product (970 mg, 3.39 mmol, 66%). The compound was directly used for the next step without analysis.

2-(4-(Benzyloxy)-3-methoxyphenyl)-2-methylbutanenitrile (10r)

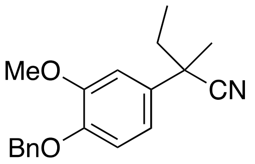

The compound was synthetized following the procedure used for 2-(4-(benzyloxy)-3-methoxyphenyl)-2-ethylbutanenitrile (**10o**). The residue was purified on silica gel column eluting with 10-20% EtOAc in cyclohexane to afford the desired product as a yellow oil (680 mg, 2.30 mmol, **62%**). **R***f* = 0.47 (20% EtOAc in cyclohexane); **^{1}H NMR** (400 MHz, $CDCl_3$) δ 7.45 – 7.25 (m, 5H), 6.95 – 6.94 (m, 1H), 6.89 – 6.84 (m, 2H), 5.13 (s, 2H), 3.90 (s, 3H), 1.96 – 1.85 (m, 2H), 1.66 (s, 3H), 0.95 (t, *J* = 7.3 Hz, 3H); **^{13}C NMR** (101 MHz, $CDCl_3$) δ 150.0, 147.9, 137.2, 133.4, 128.7, 128.1, 127.5, 123.7, 117.9, 114.3, 110.1, 71.3, 56.4, 43.0, 35.5, 27.5, 10.1; **HRMS** (pos. ESI): calcd. for $C_{19}H_{21}NO_2Na$ $[M+Na]^+$: 318.14670, found: 318.14645

2-(4-(benzyloxy)-5-methoxy-2-nitrophenyl)-2-methylbutanenitrile (11r)

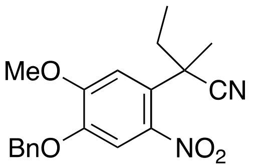

To a solution of 2-(4-(benzyloxy)-3-methoxyphenyl)-2-methylbutanenitrile (680 mg, 2.30 mmol, 1 equiv., **10r**) in Ac_2O (5.9 mL) and AcOH (4.4 mL) was added fuming HNO_3 (0.16 mL, 3.45 mmol, 1.5 equiv., 90%). The solution was stirred for 2 h at rt. Then, the solution was poured into ice-water and the resulting solution was neutralized with Na_2CO_3, extracted with DCM. The organic phase was washed with brine, dried over Na_2SO_4 and evaporated. The residue was purified on silica gel eluting with 10-30% EtOAc in cyclohexane to afford a yellow oil (684 mg, 2.30 mmol, **87%**). **R***f* =

0.31 (20% EtOAc in cyclohexane); **^{1}H NMR** (400 MHz, $CDCl_3$) δ 7.43 – 7.27 (m, 6H), 7.07 (s, 1H), 5.12 (s, 2H), 3.95 (s, 3H), 2.26 (dq, *J* = 14.1, 7.4 Hz, 1H), 2.04 (dq, *J* = 14.1, 7.4 Hz, 1H), 1.82 (s, 3H), 1.05 (t, *J* = 7.4 Hz, 3H); **^{13}C NMR** (101 MHz, $CDCl_3$) δ 152.3, 147.5, 142.7, 135.6, 128.9, 128.6, 127.7, 126.2, 122.4, 111.6, 111.2, 71.6, 56.6, 42.9, 33.5, 26.1, 10.3; **HRMS** (pos. ESI): calcd. for $C_{19}H_{20}N_2O_4Na$ $[M+Na]^+$: 363.13208, found: 363.13162

2-(4-Hydroxy-5-methoxy-2-nitrophenyl)-2-methylbutanenitrile (12r)

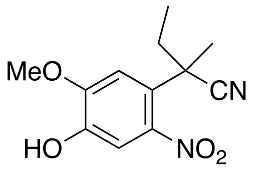

The compound was synthetized following general procedure for debenzylation starting from 2-(4-(benzyloxy)-5-methoxy-2-nitrophenyl)-2-methylbutanenitrile (**11r**). The residue was purified on silica gel eluting with 10-40% EtOAc in cyclohexane to afford a light brown solid (402 mg, 1.61 mmol, **80%**). **R_f** = 0.40 (40% EtOAc in cyclohexane); **m.p.**: 120 – 122 °C; **^{1}H NMR** (400 MHz, $CDCl_3$) δ 7.24 (s, 1H), 7.07 (s, 1H), 3.98 (s, 3H), 5.84 (br s, 1H), 2.23 (dq, *J* = 14.2, 7.4 Hz, 1H), 2.03 (dq, *J* = 14.2, 7.4 Hz, 1H), 1.81 (s, 3H), 1.04 (t, *J* = 7.4 Hz, 3H); **^{13}C NMR** (101 MHz, $CDCl_3$) δ 149.1, 145.4, 143.6, 124.7, 122.7, 112.5, 110.9, 56.7, 43.4, 33.5, 26.2, 10.4; **HRMS** (pos. ESI): calcd. for $C_{12}H_{14}N_2O_4Na$ $[M+Na]^+$: 273.08513, found: 273.0846

2-(5-Methoxy-2-nitro-4-(3-(pyrrolidin-1-yl)propoxy)phenyl)-2-methylbutanenitrile (13r)

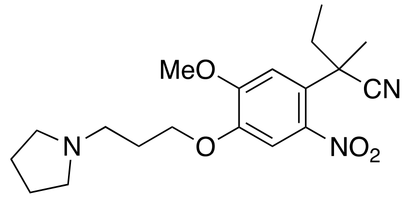

To a solution of 2-(4-hydroxy-5-methoxy-2-nitrophenyl)-2-methylbutanenitrile (**12r** 150 mg, 0.60 mmol, 1 equiv.) in DMF (3.9 mL) was added 1-(3-chloropropyl)pyrrolidine (**19**, 106 mg, 0.72 mmol, 1.2 equiv.), K_2CO_3 (116 mg, 0.84 mmol, 1.4 equiv.) and NaI (18 mg, 0.12 mmol, 0.2 equiv.). The mixture was heated at 80 °C for 1 h. Water wad added and the aqueous phase was extracted three times with EtOAc. The combined organic phase was washed with brine, dried over Na_2SO_4 and concentrated under reduced pressure. The residue was purified on silica gel column eluting with 0-10% MeOH in DCM afforded the desired product (175 mg, 0.48 mmol, **81%**) as a light yellow oil. **R_f** = 0.45 (10% MeOH in DCM); **^{1}H NMR** (400 MHz, $CDCl_3$) δ 7.26 (s, 1H), 7.06 (s, 1H), 4.11 (t, *J* = 6.6 Hz, 2H), 3.93 (s, 3H), 2.65 (t, *J* = 7.3 Hz, 2H), 2.60 – 2.50 (m, 4H), 2.25 (dq, *J* = 14.1, 7.4 Hz, 1H), 2.11 – 1.97 (m, 3H), 1.83 – 1.74 (m, 4H), 1.82 (s, 3H), 1.04 (t, *J* = 7.5 Hz, 3H); **^{13}C NMR** (101 MHz, $CDCl_3$) δ 152.1, 148.1, 143.0, 125.6, 122.6, 111.8, 110.6, 68.2, 56.7, 54.4, 52.9, 43.3, 33.5, 28.5, 10.4; **HRMS** (pos. ESI): calcd. for $C_{19}H_{28}N_3O_4$ $[M+H]^+$: 362.20798, found: 362.2074

3-Ethyl-5-methoxy-3-methyl-6-(3-(pyrrolidin-1-yl)propoxy)-3*H*-indol-2-amine (1r)

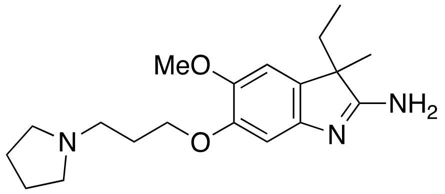

To a solution of 2-(5-methoxy-2-nitro-4-(3-(pyrrolidin-1-yl)propoxy)phenyl)-2-methylbutanenitrile (**13r**, 163 mg, 0.45 mmol, 1 equiv.) in AcOH (1.54 mL) was added zinc dust (442 mg, 6.77 mmol, 15 equiv.). The reaction mixture was heated up to 80 °C and was stirred at that temperature for 1.5 h. The solids were filtered and washed with AcOH. The filtrate was concentrated under reduced pressure. The residue was dissolved using EtOAc and the resulting solution was washed with an aqueous solution of NaOH (5 mL, 1M). The layers were separated and the aqueous layer was extracted three times with EtOAc. The combined organic layers were dried over Na_2SO_4, filtered and concentrated under reduced pressure. The product was obtained as a light yellow gum (99 mg, 0.30 mmol, **66%**). **^{1}H NMR** (400 MHz, $CDCl_3$) δ 6.84 (br s, 1H), 6.65 (s, 1H), 6.26 (br s, 2H), 4.04 (t, *J* = 6.7 Hz, 2H), 3.81 (s, 3H), 2.65 – 2.59 (m, 2H), 2.57 – 2.49 (m, 4H), 2.06 – 1.97 (m, 2H), 1.84 – 1.65 (m, 6H), 1.33 (s, 3H), 0.51 (t, *J* = 7.2 Hz, 3H); **^{13}C NMR** (101 MHz, $CDCl_3$) δ 178.8, 149.0, 145.6, 145.1, 128.5, 107.7, 102.2, 67.9, 57.5, 54.3, 53.5, 53.2, 32.0, 28.8, 24.5, 23.6, 8.7; **HRMS** (pos. ESI): calcd. for $C_{19}H_{30}N_3O_2$ $[M+H]^+$: 332.23380, found: 332.2333

20. <u>3-Ethyl-6-(3-(isoindolin-2-yl)propoxy)-5-methoxy-3-methyl-3*H*-indol-2-amine (1s)</u>

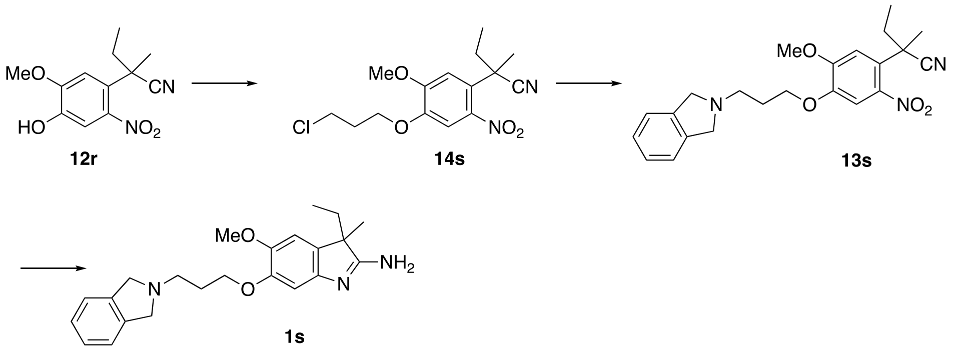

2-(4-(3-Chloropropoxy)-5-methoxy-2-nitrophenyl)-2-methylbutanenitrile (14s)

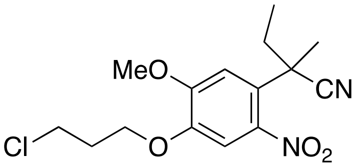

To a solution of 2-(4-hydroxy-5-methoxy-2-nitrophenyl)-2-methylbutanenitrile (**12r**, 640 mg, 2.56 mmol, 1 equiv.) in MeCN (25.6 mL) were added 1-bromo-3-chloropropane (0.28 mL, 2.81 mmol, 1.1 equiv.), K_2CO_3 (530 mg, 3.84 mmol, 1.5 equiv.) and NaI (38 mg, 0.26 mmol, 0.1 equiv.). The mixture was stirred at rt for 16 h. Water wad added, and the aqueous phase was extracted three times with EtOAc. The combined organic phase was washed with brine, dried over Na_2SO_4 and concentrated under reduced pressure. The residue was purified on silica gel column eluting with 0-100% AcOEt in cyclohexane to afford the desired product (735 mg, 2.25 mmol, **88%**) as a light yellow oil. **R*f*** = 0.35 (30% EtOAc in cyclohexane); **^{1}H NMR** (400 MHz, $CDCl_3$) δ 7.26 (s, 1H), 7.09 (s, 1H), 4.19 (t, *J* = 5.9 Hz, 2H), 3.95 (s, 3H), 3.76 (t, *J* = 6.3 Hz, 2H), 2.36 – 2.22 (m, 3H), 2.12 – 1.97 (m, 1H), 1.84 (s, 3H), 1.07 (t, *J* = 7.4 Hz, 3H); **^{13}C NMR** (101 MHz, $CDCl_3$) δ 152.0, 147.6, 142.8, 126.0, 122.3, 111.6, 110.6, 66.1, 56.5, 43.1, 41.2, 33.4, 32.0, 26.0, 10.3; **HRMS** (pos. ESI): calcd. for $C_{15}H_{19}ClN_2O_4Na$ $[M+Na]^+$: 349.09255, found: 349.0926.

2-(4-(3-(Isoindolin-2-yl)propoxy)-5-methoxy-2-nitrophenyl)-2-methylbutanenitrile (13s)

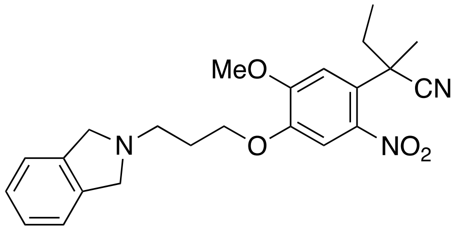

To a solution of 2-(4-(3-chloropropoxy)-5-methoxy-2-nitrophenyl)-2-methylbutanenitrile (**14s**, 300 mg, 1.20 mmol, 1 equiv.) in DMF (7.8 mL) were added K_2CO_3 (199 mg, 1.44 mmol, 1.2 equiv.), NaI (18 mg, 0.12 mmol, 0.1 equiv.) and isoindoline (429 mg, 3.60 mmol, 3 equiv.). The mixture was heated at 80 °C for 1 h. Water was added, and the aqueous phase was extracted three times with EtOAc. The combined organic phase was washed with brine, dried over Na_2SO_4 and concentrated under reduced pressure. The residue was purified on silica gel column eluting with 0-10% MeOH in DCM to afford the desired product (421 mg, 1.03 mmol, **86%**) as a light-yellow oil. **R*f***= 0.37 (8% MeOH in DCM); **^{1}H NMR** (400 MHz, MeOD-*d*$_4$) δ 7.48 – 7.40 (m, 5H), 7.05 (s, 1H), 4.82 – 4.77 (m, 4H), 4.29 (t, *J* = 5.6 Hz, 2H), 3.75 (s, 3H), 3.73 (t, *J* = 7.0 Hz, 2H), 2.44 – 2.35 (m, 2H), 2.35 – 2.25 (m,

1H), 2.11 – 1.97 (m, 1H), 1.83 (s, 3H), 1.03 (t, *J* = 7.4 Hz, 3H); ^{13}**C NMR** (101 MHz, MeOD-d_4) δ 153.4, 148.4, 144.3, 135.1, 130.2, 127.6, 124.0, 122.8, 111.5, 111.3, 68.6, 60.1, 57.0, 54.6, 42.6, 34.7, 27.1, 26.5, 10.2; **HRMS** (pos. ESI): calcd. for $C_{23}H_{28}N_3O_4H$ [M+H]$^+$: 410.20743, found: 410.2074

3-Ethyl-6-(3-(isoindolin-2-yl)propoxy)-5-methoxy-3-methyl-3*H*-indol-2-amine (1s)

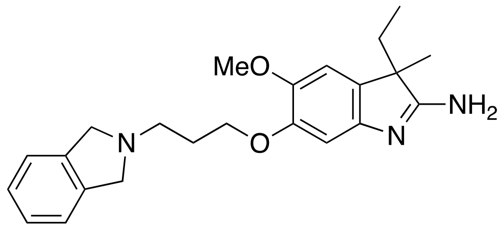

To a solution of 2-(4-(3-(isoindolin-2-yl)propoxy)-5-methoxy-2-nitrophenyl)-2-methylbutanenitrile (**13s**, 220 mg, 0.54 mmol, 1 equiv.) in AcOH (3.7 mL) was added zinc dust (527 mg, 8.06 mmol, 15 equiv.). The reaction mixture was heated up to 80 °C and was stirred at that temperature for 1.5 h. The solids were filtered and washed with AcOH. The filtrate was concentrated under reduced pressure. The residue was dissolved using EtOAc and an aqueous solution of NaOH (10 mL, 2M). The layers were separated, and the aqueous layer was extracted three times with EtOAc. The combined organic layers were dried over Na_2SO_4, filtered and concentrated under reduced pressure. The product was obtained as a light yellow solid (146 mg, 0.38 mmol, **72%**). ^{1}**H NMR** (400 MHz, MeOD-d_4) δ 7.26 – 7.15 (m, 5H), 6.82 (s, 1H), 6.75 (s, 1H), 4.10 (t, *J* = 6.2 Hz, 2H), 3.97 (s, 4H), 3.81 (s, 3H), 3.02 – 2.91 (m, 2H), 2.16 – 1.98 (m, 2H), 1.92 – 1.72 (m, 2H), 1.33 (s, 3H), 0.49 (t, *J* = 7.3 Hz, 3H); ^{13}**C NMR** (101 MHz, MeOD-d_4) δ 181.0, 150.1, 149.8, 146.5, 140.7, 132.5, 128.1, 123.3, 109.4, 103.8, 68.9, 60.0, 58.1, 54.9, 54.2, 32.1, 29.6, 24.7, 9.0; **HRMS** (pos. ESI): calcd. for $C_{23}H_{29}N_3O_4$ [M+H]$^+$: 380.23325, found: 380.2333

21. 3-Ethyl-5-methoxy-3-methyl-6-(3-((R)-3-methylpyrrolidin-1-yl)propoxy)-3H-indol-2-amine (1t)

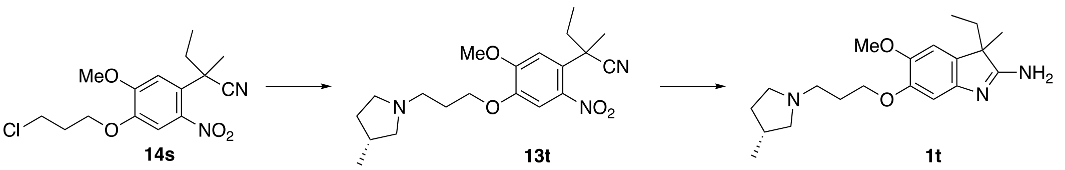

2-(5-Methoxy-4-(3-((*R*)-3-methylpyrrolidin-1-yl)propoxy)-2-nitrophenyl)-2-methylbutanenitrile (13t)

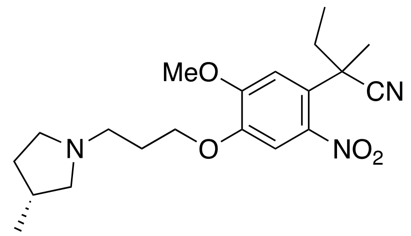

To a solution of 2-(4-(3-chloropropoxy)-5-methoxy-2-nitrophenyl)-2-methylbutanenitrile (**14s**, 220 mg, 0.67 mmol, 1 equiv.) in DMF (4.4 mL) were added K_2CO_3 (205 mg, 1.48 mmol, 2.2 equiv.), NaI (10 mg, 0.07 mmol, 0.1 equiv.) and (*R*)-3-methylpyrrolidine hydrochloride (246 mg, 2.02 mmol, 3 equiv.). The mixture was heated at 80 °C for 1 h. Water was added, and the aqueous phase was extracted three times with EtOAc. The combined organic phase was washed with brine, dried over Na_2SO_4 and concentrated under reduced pressure. The residue was purified on silica gel column eluting with 0-10% MeOH in DCM afforded the desired product (124 mg, 0.33 mmol, **49%**) as a light-yellow oil. **R*f*** = 0.33 (8% MeOH in DCM); **^{1}H NMR** (400 MHz, $CDCl_3$) δ 7.27 (s, 1H), 7.04 (s, 1H), 4.10 (t, *J* = 6.6 Hz, 2H), 3.93 (s, 3H), 2.83 – 2.75 (m, 1H), 2.68 – 2.47 (m, 3H), 2.45 – 2.39 (m, 1H), 2.32 – 2.15 (m, 2H), 2.09 – 1.93 (m, 5H), 1.81 (d, *J* = 0.8 Hz, 3H), 1.39 – 1.25 (m, 1H), 1.04 (t, *J* = 7.4 Hz, 3H), 0.99 (d, *J* = 6.8 Hz, 3H); **^{13}C NMR** (101 MHz, $CDCl_3$) δ 151.9, 147.9, 142.8, 125.3, 122.3, 111.4, 110.3, 68.0, 62.3, 56.5, 54.2, 52.8, 42.9, 33.3, 32.6, 31.9, 28.4, 26.0, 20.4, 10.2; **HRMS** (pos. ESI): calcd. for $C_{20}H_{30}N_3O_4$ $[M+H]^+$: 376.2230, found: 376.2234

3-Ethyl-5-methoxy-3-methyl-6-(3-((*R*)-3-methylpyrrolidin-1-yl)propoxy)-3H-indol-2-amine (1t)

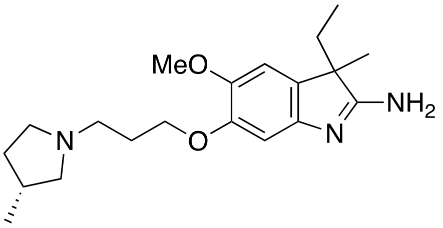

To a solution of 2-(5-methoxy-4-(3-((*R*)-3-methylpyrrolidin-1-yl)propoxy)-2-nitrophenyl)-2-methylbutanenitrile (**13t**, 70 mg, 0.19 mmol, 1 equiv.) in AcOH (1.3 mL) was added zinc dust (183 mg, 3.09 mmol, 15 equiv.). The reaction mixture was heated up to 80 °C and was stirred at that temperature for 1.5 h. The solids were filtered and washed with AcOH. The filtrate was concentrated under reduced pressure. The residue was dissolved using EtOAc and an aqueous solution of NaOH (10 mL, 2M). The layers were separated, and the aqueous layer was extracted three times with EtOAc. The combined organic layers were dried over Na_2SO_4, filtered and concentrated under reduced pressure. The product was obtained as a light yellow solid (146 mg, 0.38 mmol, **72%**). **^{1}H NMR** (400 MHz, MeOD-*d*$_4$) δ 6.80 (s, 1H), 6.70 (s, 1H), 4.13 – 3.88 (m, 2H), 3.80

(s, 3H), 2.99 – 2.86 (m, 1H), 2.82 – 2.74 (m, 1H), 2.72 – 2.59 (m, 3H), 2.59 – 2.49 (m, 1H), 2.33 – 2.18 (m, 1H), 2.12 – 1.88 (m, 5H), 1.89 – 1.75 (m, 2H), 1.44 – 1.32 (m, 1H), 1.32 (s, 3H), 1.04 (d, *J* = 6.8 Hz, 3H), 0.48 (t, *J* = 7.4 Hz, 3H); **^{13}C NMR** (101 MHz, MeOD-*d*$_4$) δ 180.4, 149.8, 146.3, 132.1, 109.0, 103.4, 68.7, 62.9, 57.9, 54.8, 54.6, 33.2, 32.1, 29.3, 24.7, 20.2, 9.1; **HRMS** (pos. ESI): calcd. for $C_{20}H_{31}N_3O_2Na$ [M+Na]$^+$: 368.23084, found; 368.2307

22. **(1-(3-((2'-Amino-5'-methoxyspiro[cyclopentane-1,3'-indol]-6'-yl)oxy)propyl)pyrrolidin-3-yl)methanol (1u)**

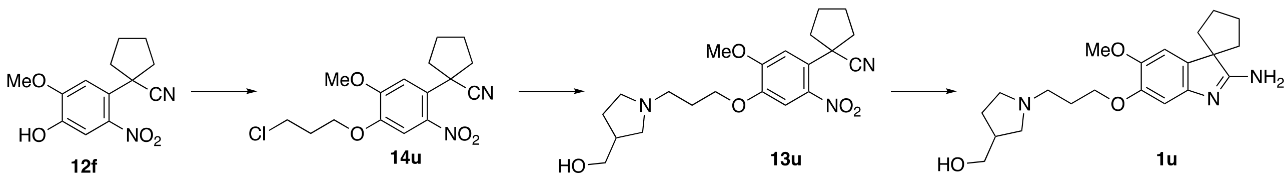

1-(4-(3-Chloropropoxy)-5-methoxy-2-nitrophenyl)cyclopentane-1-carbonitrile (14u)

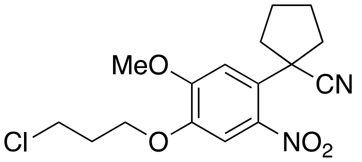

To a solution of 1-(4-hydroxy-5-methoxy-2-nitrophenyl)cyclopentane-1-carbonitrile (**12f**, 2.20 g, 8.39 mmol, 1 equiv.) in MeCN (83.9 mL) at rt were added K_2CO_3 (1.74 g, 12.6 mmol, 1.5 equiv) and 1-bromo-3-chloropropane (913 µL, 9.23 mmol, 1.1 equiv). The mixture was allowed to stir at rt for 16 h. Water was added and the aqueous phase was extracted three times with EtOAc. The combined organic phase was washed with brine, dried over Na_2SO_4 and evaporated. Purification on silica gel column eluting with 10-60% EtOAc in cyclohexane afforded the desired product (2.14 g, 6.32 mmol, 75%) as a yellow solid. **^{1}H NMR** (400 MHz, $CDCl_3$) δ 7.46 (s, 1H), 6.97 (s, 1H), 4.25 – 4.17 (m, 2H), 3.94 (s, 3H), 3.75 (t, *J* = 6.2 Hz, 2H), 2.72 – 2.60 (m, 2H), 2.30 (p, *J* = 6.1 Hz, 2H), 2.10 – 1.98 (m, 4H), 1.93 – 1.84 (m, 2H); **13C NMR** (101 MHz, $CDCl_3$) δ 152.5, 147.7, 142.6, 127.5, 122.8, 111.0, 110.8, 66.1, 56.5, 45.4, 41.1, 39.0, 31.9, 23.9

1-(4-(3-(3-(Hydroxymethyl)pyrrolidin-1-yl)propoxy)-5-methoxy-2-nitrophenyl)cyclopentane-1-carbonitrile (13u)

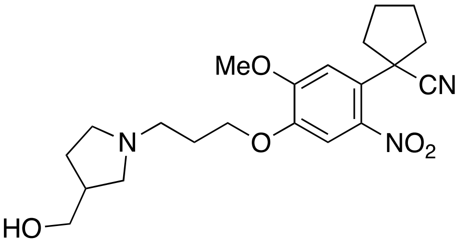

A solution of 1-(4-(3-chloropropoxy)-5-methoxy-2-nitrophenyl)cyclopentane-1-carbonitrile (250 mg, 0.74 mmol, 1 equiv.), pyrrolidin-3-ylmethanol hydrochloride (254 mg, 1.85 mmol, 2.5 equiv.), K_2CO_3 (153 mg, 1.11 mmol, 1.5 equiv.) and NaI (11 mg, 0.07 mmol, 0.1 equiv.) in DMF (5.9 mL) was heated at 80 °C for 2 h. The solvent was evaporated, and the residue was purified using silica gel column eluting with 0-20% MeOH in DCM (+ 2% NEt_3) to afford the desired product (124 mg, 0.31 mmol, **42%**) as a yellow oil. **R_f** = 0.12 (12% MeOH in DCM + 2% NEt_3); **^{1}H NMR** (500 MHz, $CDCl_3$) δ 7.45 (s, 1H), 6.95 (s, 1H), 4.12 (t, *J* = 6.4 Hz, 2H), 3.94 (s, 3H), 3.65 (dd, *J* = 10.2, 4.7 Hz, 1H), 3.53 (dd, *J* = 10.2, 5.5 Hz, 1H), 2.97 (s, 1H), 2.87 (td, *J* = 8.8, 4.7 Hz, 1H), 2.78 – 2.56 (m, 6H), 2.52 – 2.33 (m, 2H), 2.09 – 1.99 (m, 7H), 1.91 – 1.85 (m, 2H), 1.74 – 1.57 (m, 1H); **^{13}C NMR** (126 MHz, $CDCl_3$) δ 152.4, 147.8, 142.6, 127.2, 122.9, 110.9, 110.6, 67.7, 66.7, 57.9, 56.5, 54.0, 52.4, 45.4, 39.0, 27.9, 26.9, 23.9; **HRMS** (pos. ESI): calcd. for $C_{21}H_{30}N_3O_5$ $[M+H]^+$: 404.21799, found: 404.2180

(1-(3-((2'-Amino-5'-methoxyspiro[cyclopentane-1,3'-indol]-6'-yl)oxy)propyl)pyrrolidin-3-yl)methanol (1u)

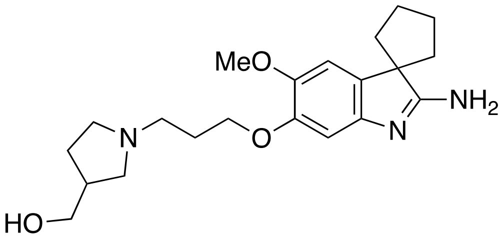

To a solution of 1-(4-(3-(3-(hydroxymethyl)pyrrolidin-1-yl)propoxy)-5-methoxy-2-nitrophenyl)cyclopentane-1-carbonitrile (**13u**, 105 mg, 0.26 mmol, 1 equiv.) in AcOH (1.9 mL) was added zinc dust (255 mg, 3.90 mmol, 15 equiv.). The reaction mixture was heated up to 80 °C and was stirred at that temperature for 1.5 h. The solids were filtered and washed with AcOH. The filtrate was concentrated under reduced pressure. The residue was dissolved in EtOAc and an aqueous solution of NaOH (10 mL, 2M). The layers were separated, and the aqueous layer was extracted three times with EtOAc. The combined organic layers were dried over Na_2SO_4, filtered and concentrated under reduced pressure. The product was obtained as a light yellow solid (43 mg, 0.12 mmol, **44%**). **^{1}H NMR** (500 MHz, MeOD-*d*$_4$) δ 6.85 (s, 1H), 6.71 (s, 1H), 4.05 (t, *J* = 6.1 Hz, 2H), 3.79 (s, 3H), 3.53 – 3.45 (m, 2H), 2.87 – 2.80 (m, 1H), 2.82 – 2.66 (m, 4H), 2.46 – 2.35 (m, 1H), 2.45 – 2.36 (m, 1H), 2.12 – 1.93 (m, 9H), 1.87 – 1.80 (m, 2H), 1.59 – 1.54 (m, 1H); **^{13}C NMR** (126 MHz, MeOD-*d*$_4$) δ 182.1, 150.0, 149.0, 146.4, 136.7, 109.8, 103.9, 69.0, 66.2, 60.1, 58.4, 58.3, 55.0, 54.5, 40.9, 39.3, 29.5, 28.1, 27.2; **HRMS** (pos. ESI): calcd. for $C_{21}H_{32}N_3O_3$ [M+H]$^+$: 374.2438, found: 374.2438

NMR spectra

4',5'-diethoxy-6'-(3-(pyrrolidin-1-yl)propoxy)spiro[cyclopentane-1,3'-indol]-2'-amine (1a)

^{1}H NMR (400 MHz, $CDCl_3$):

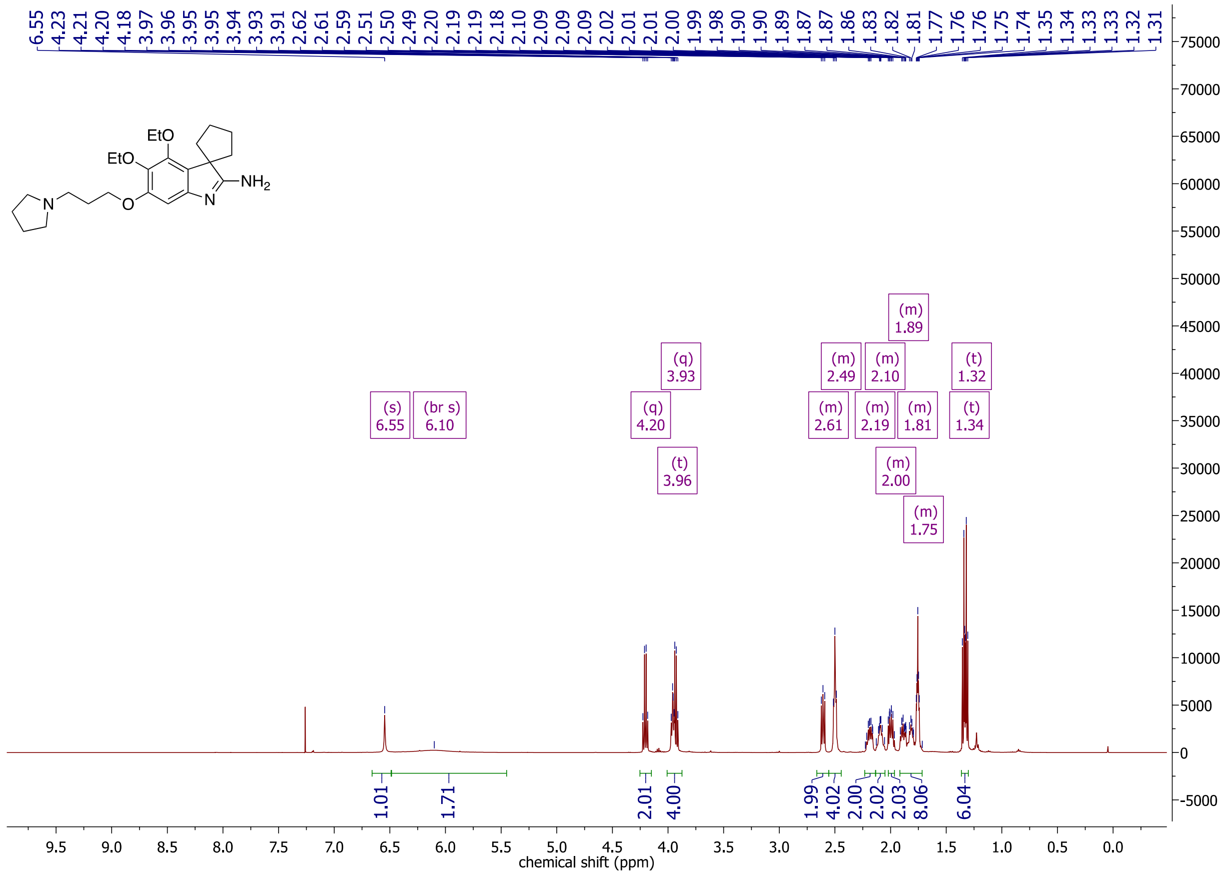

^{13}C NMR (101 MHz, $CDCl_3$, 60 °C):

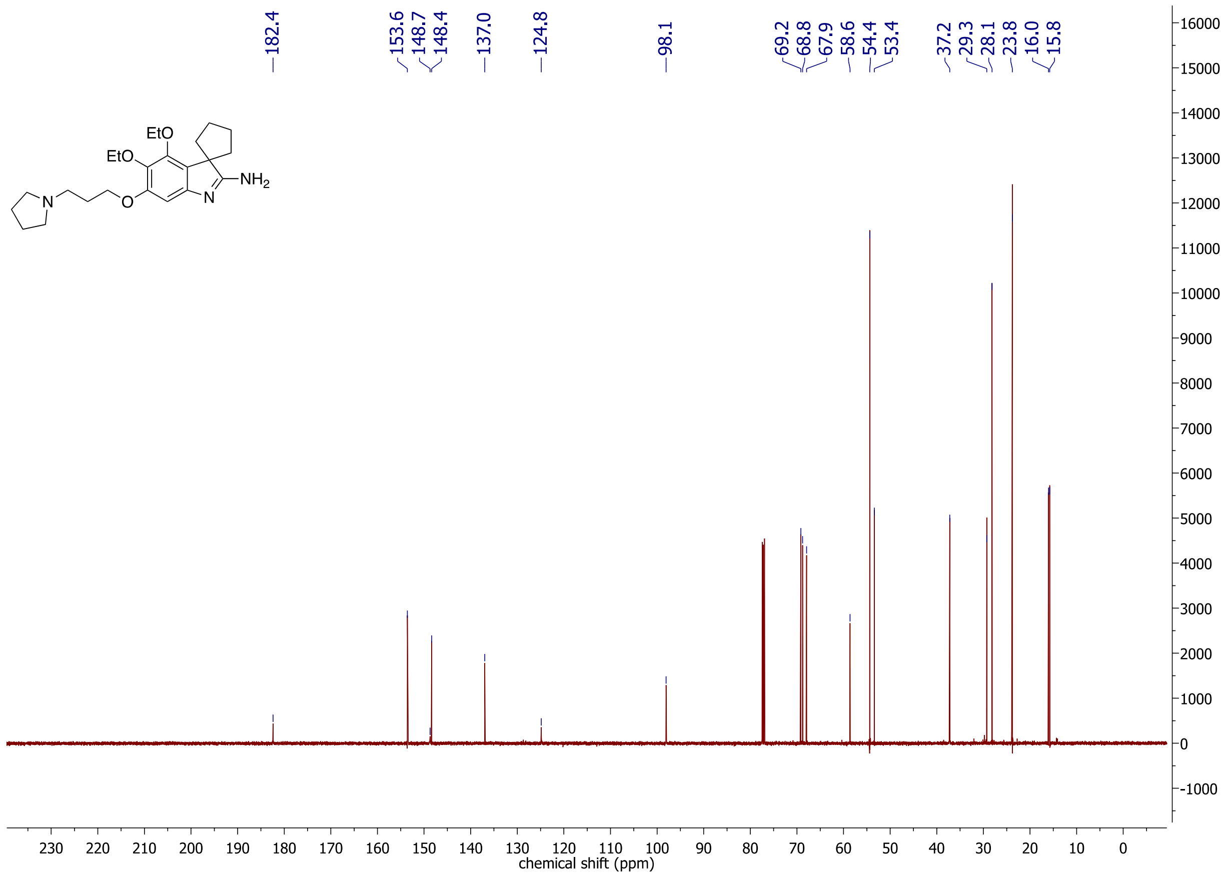
182.4
153.6
148.7
148.4
137.0
124.8
98.1
69.2
68.8
67.9
58.6
54.4
53.4
37.2
29.3
28.1
23.8
16.0
15.8
EtO
EtO
O
N
N
NH2
16000
15000
14000
13000
12000
11000
10000
9000
8000
7000
6000
5000
4000
3000
2000
1000
0
-1000
230 220 210 200 190 180 170 160 150 140 130 120 110 100 90 80 70 60 50 40 30 20 10 0
chemical shift (ppm)

1-(3-((2'-Amino-5'-methoxyspiro[cyclopentane-1,3'-indol]-6'-yl)oxy)propyl)pyrrolidin-3-one (1b)

^{1}H NMR (400 MHz, MeOD-*d*$_4$):

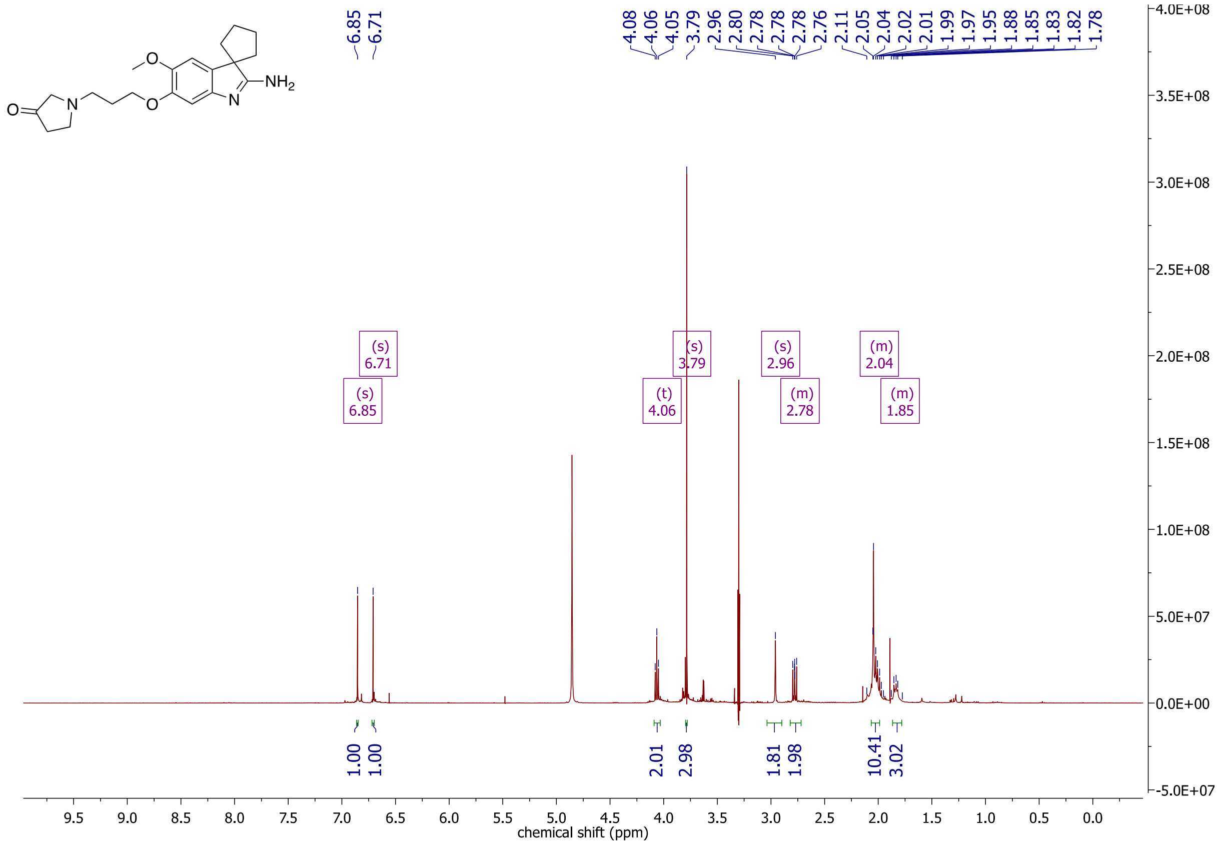

^{13}C NMR (101 MHz, MeOD-*d*$_4$):

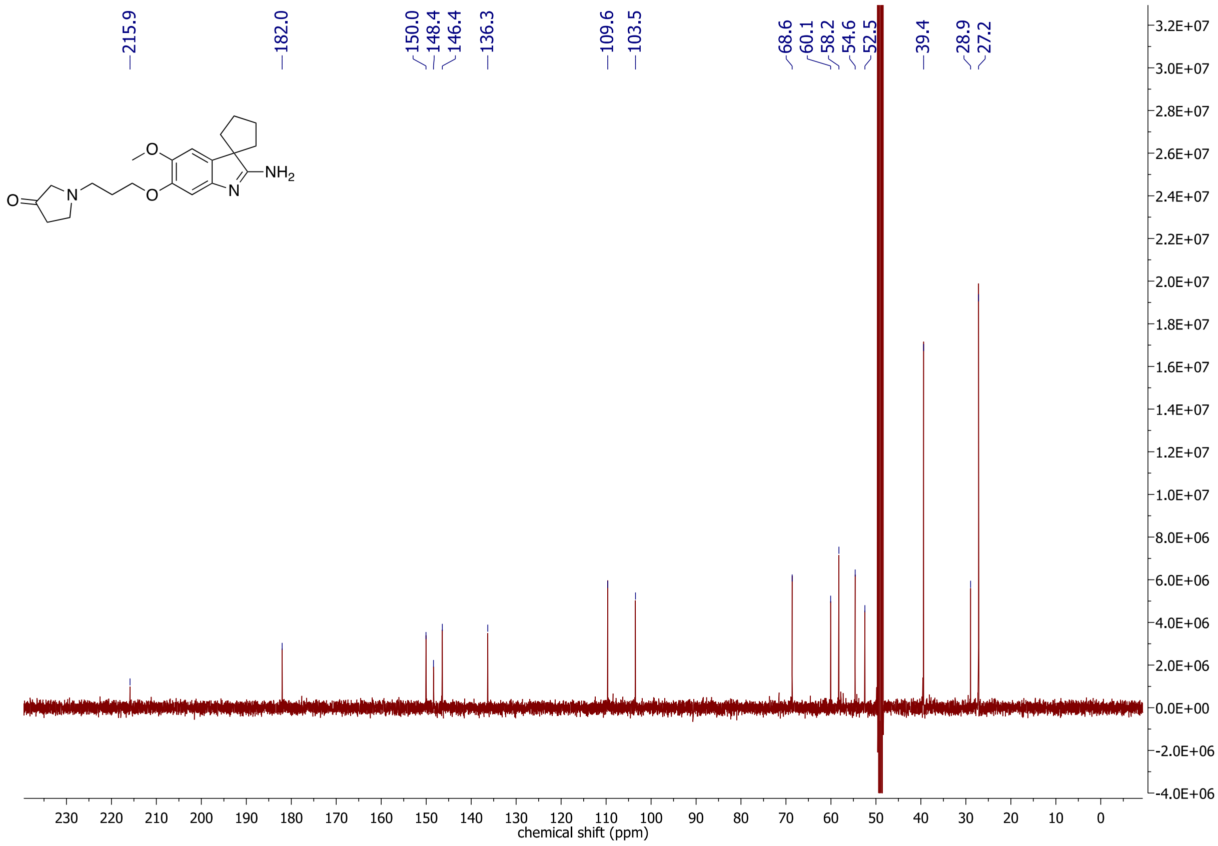

5'-Methoxy-6'-(4-(pyrrolidin-1-yl)butoxy)spiro[cyclohexane-1,3'-indol]-2'-amine (1c)

^{1}H NMR (400 MHz, $CDCl_3$):

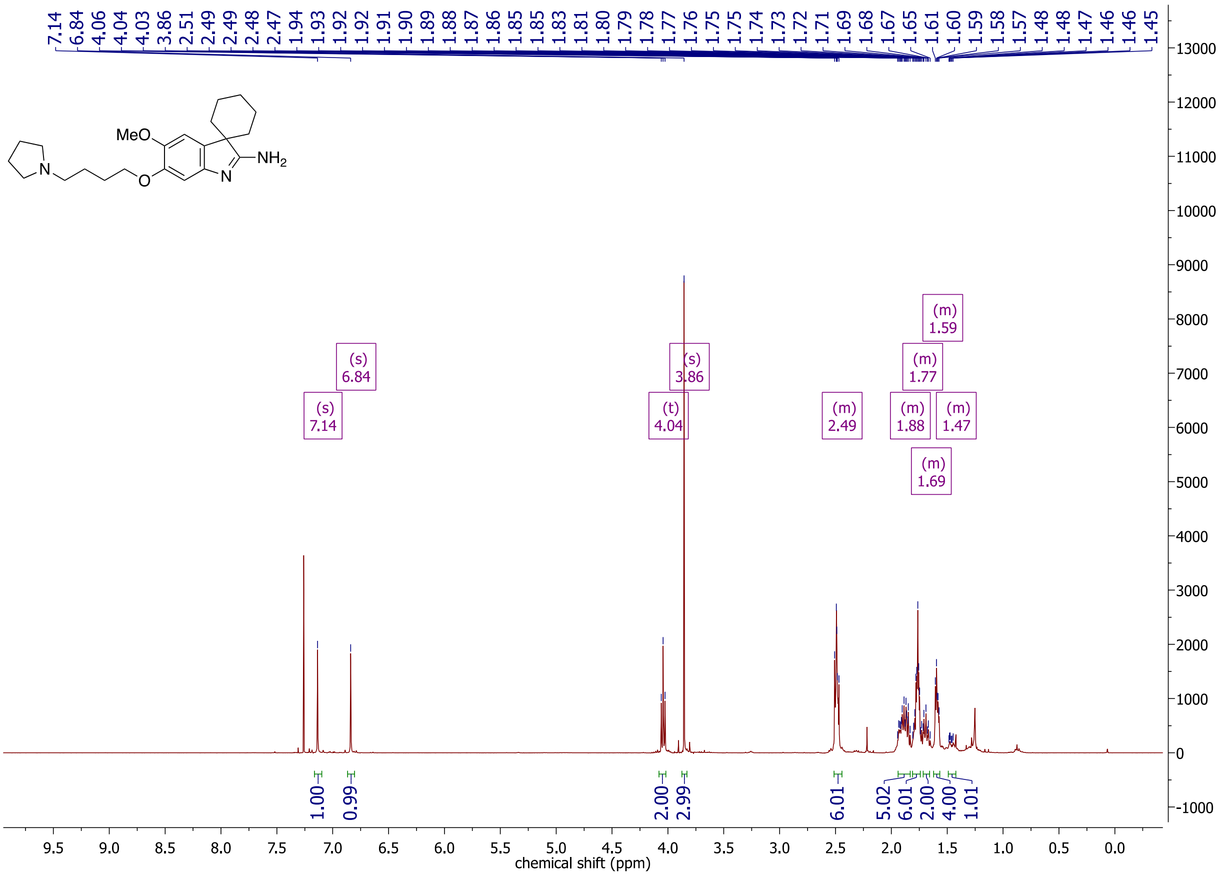

^{13}C NMR (101 MHz, $CDCl_3$):

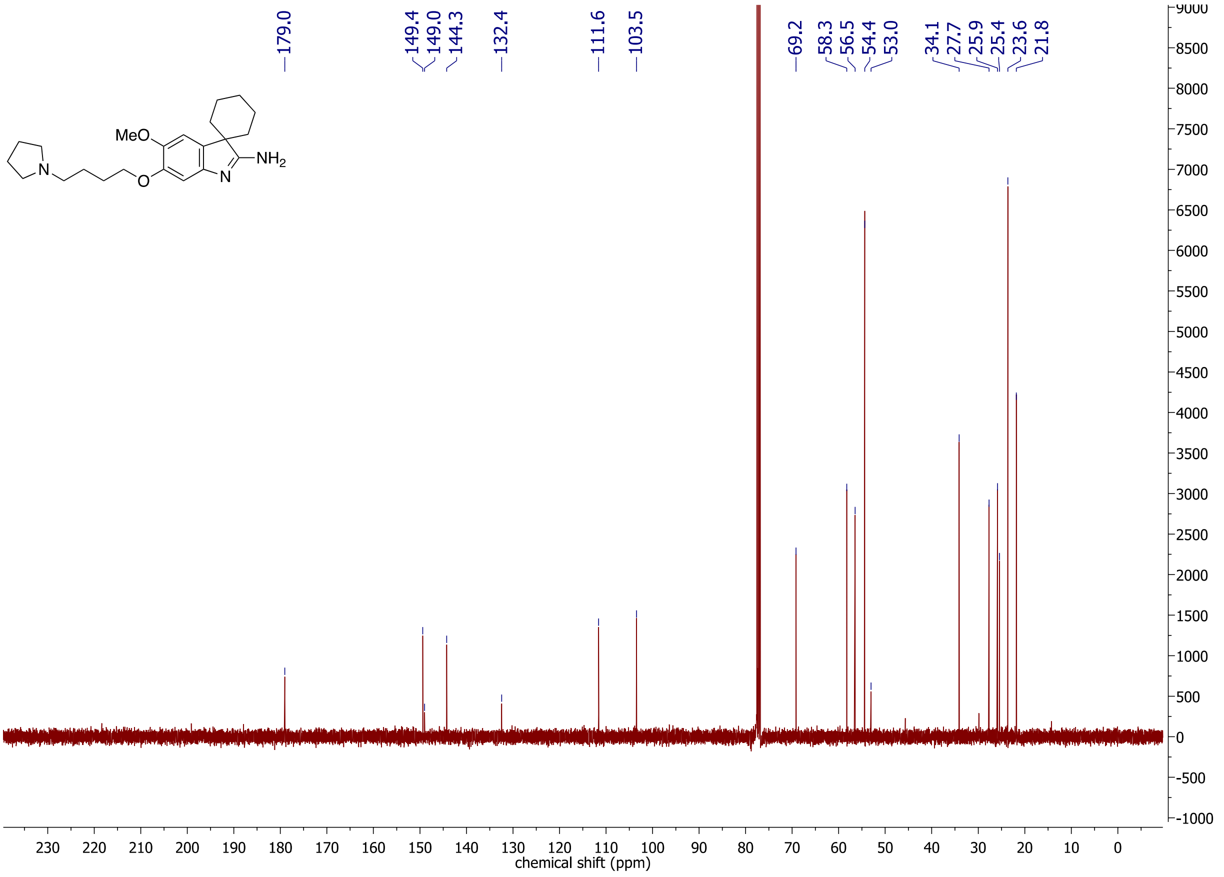

5'-Methoxy-6'-(2-(pyrrolidin-1-yl)ethoxy)spiro[cyclohexane-1,3'-indol]-2'-amine (1d)

^{1}H NMR (500 MHz, $CDCl_3$):

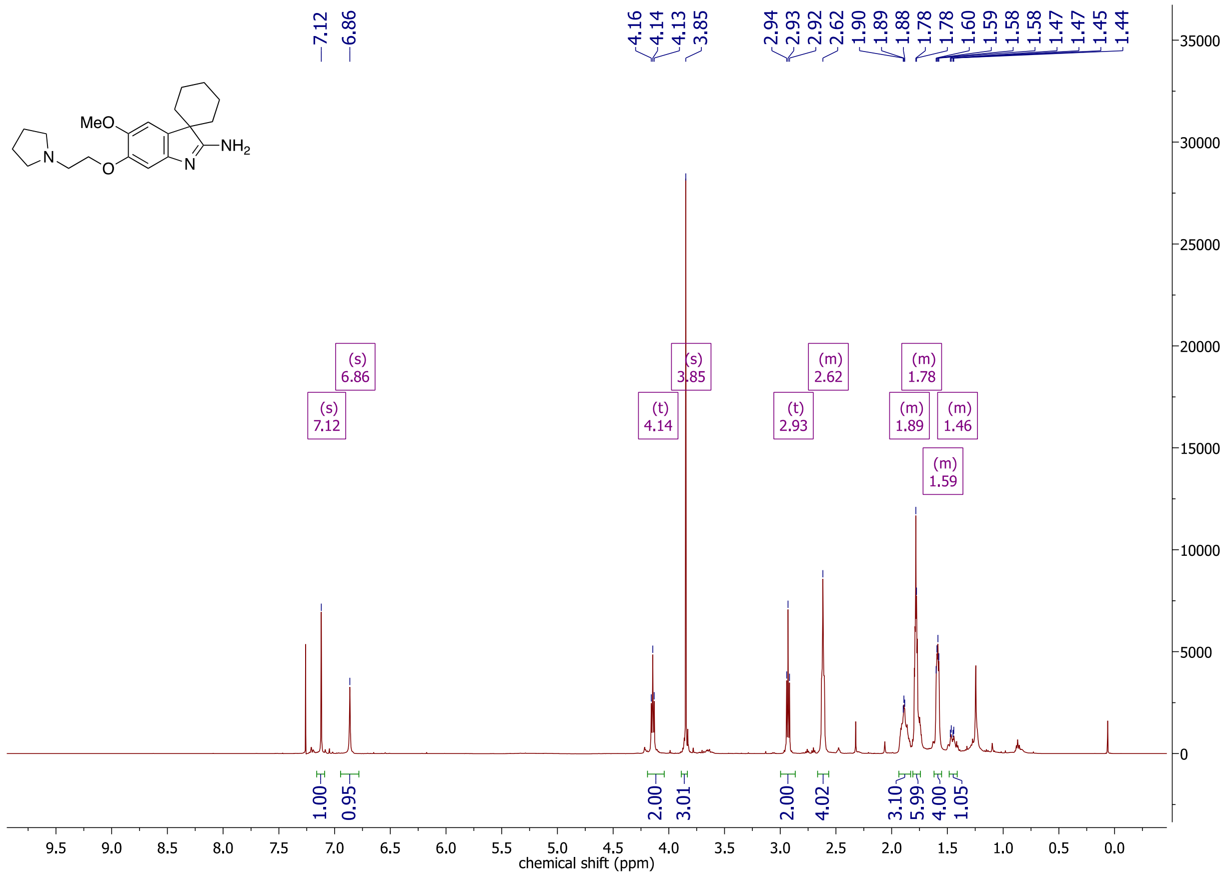

^{13}C NMR (126 MHz, $CDCl_3$):

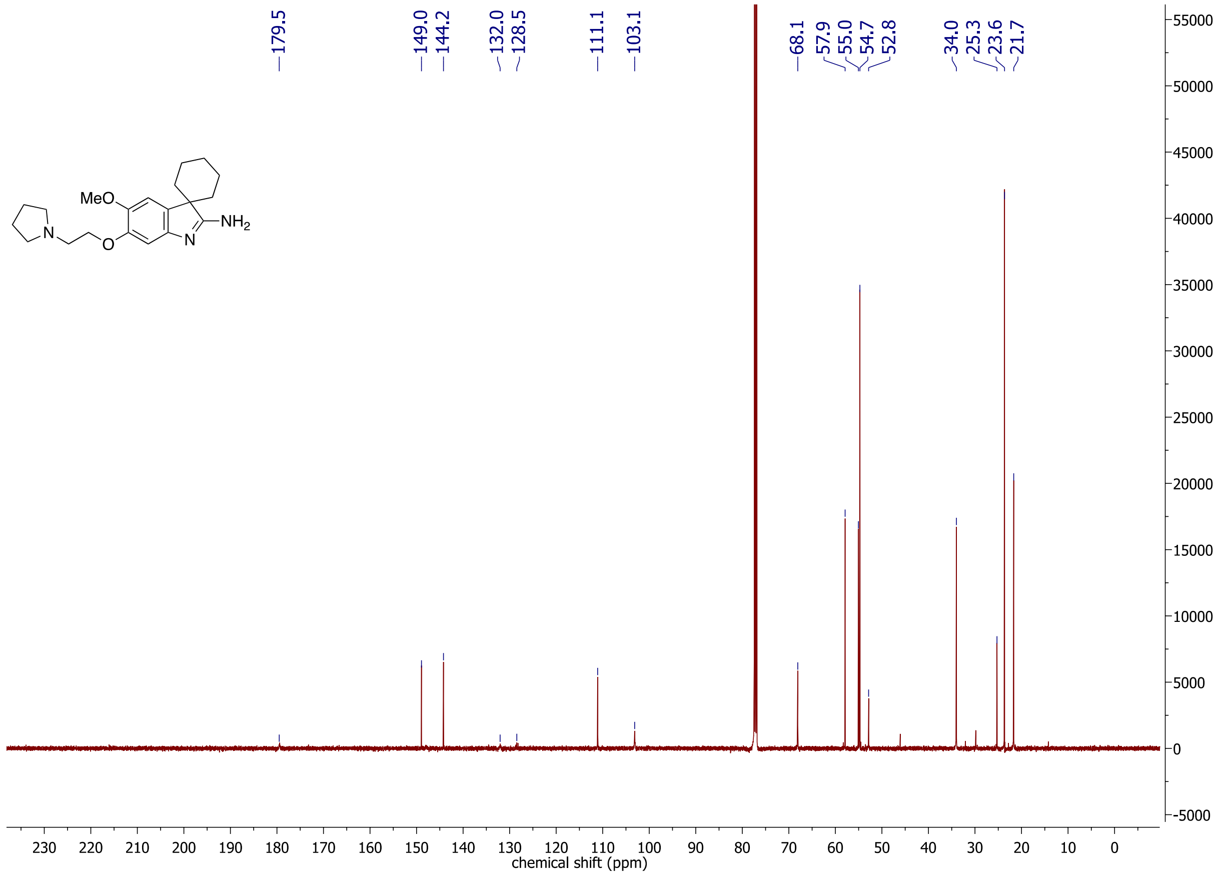

5'-Methoxy-6'-(2-(pyrrolidin-1-yl)ethoxy)spiro[cyclobutane-1,3'-indol]-2'-amine (1e)

^{1}H NMR (400 MHz, $CDCl_3$):

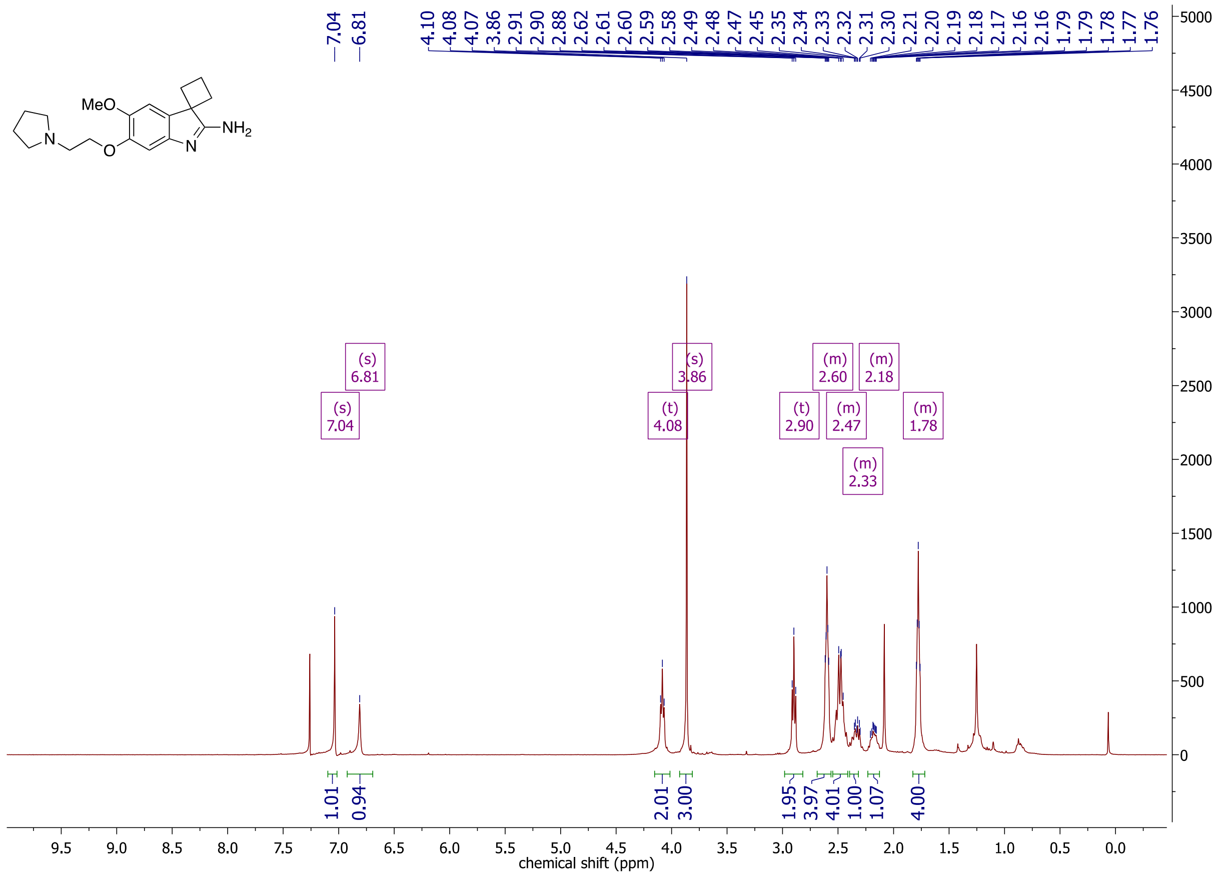

^{13}C NMR (101 MHz, $CDCl_3$):

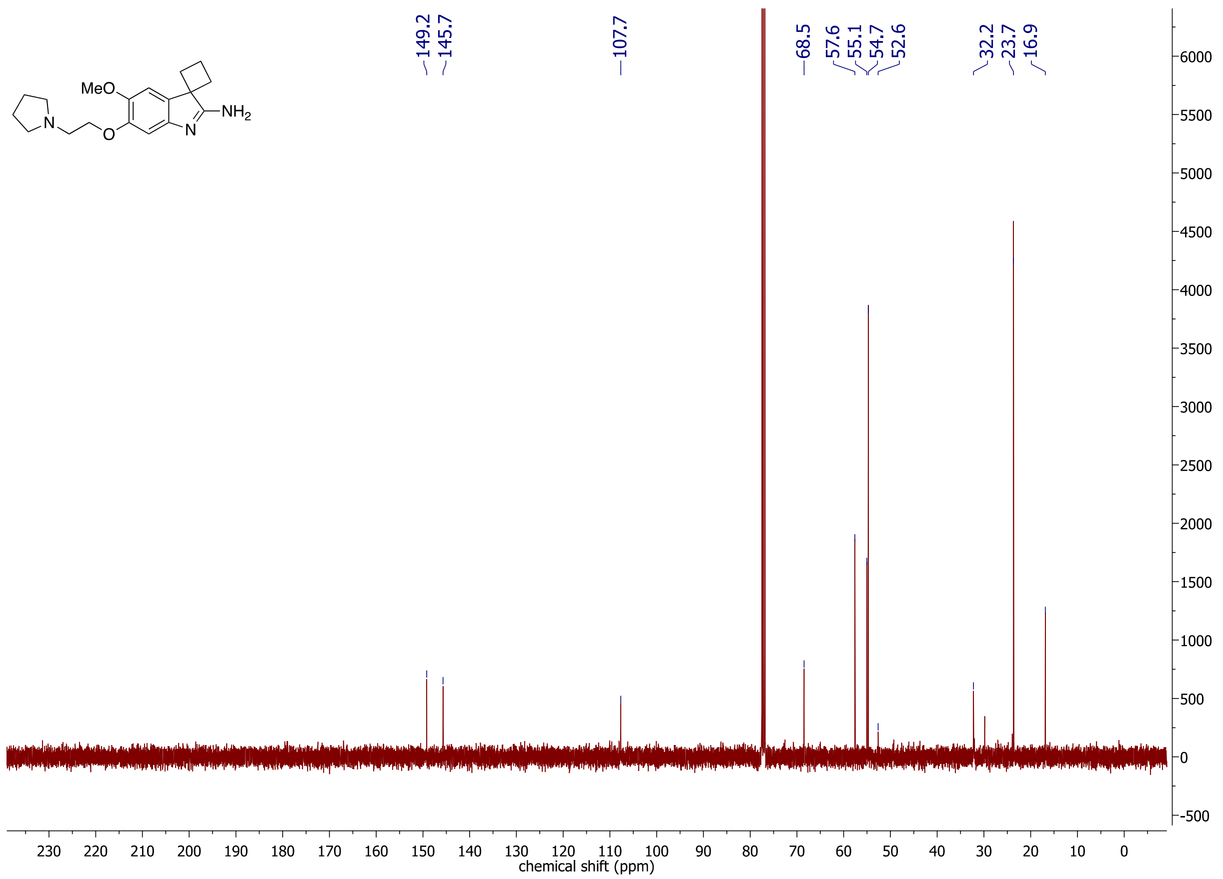

5'-Methoxy-6'-(3-(pyrrolidin-1-yl)propoxy)spiro[cyclopentane-1,3'-indol]-2'-amine (1f)

^{1}H NMR (500 MHz, $CDCl_3$):

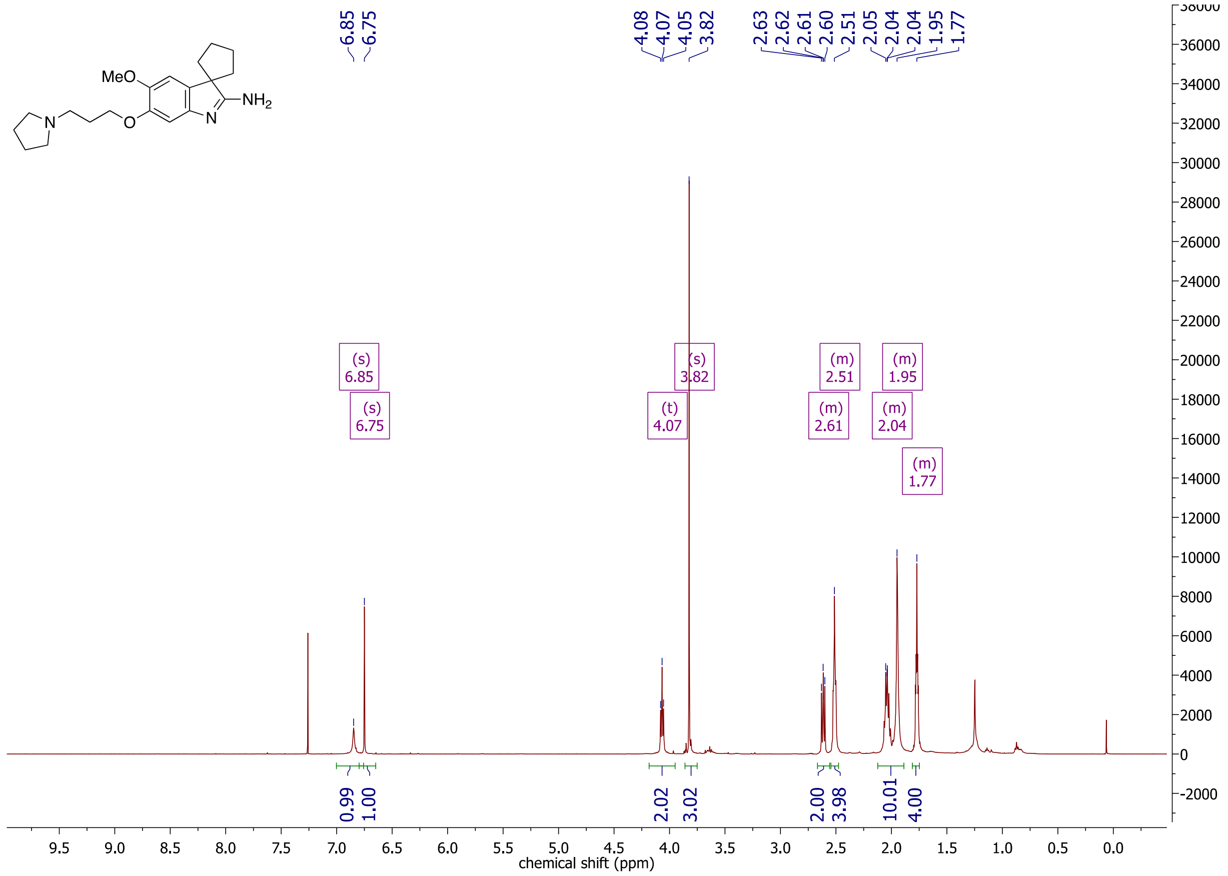

^{13}C NMR (126 MHz, $CDCl_3$):

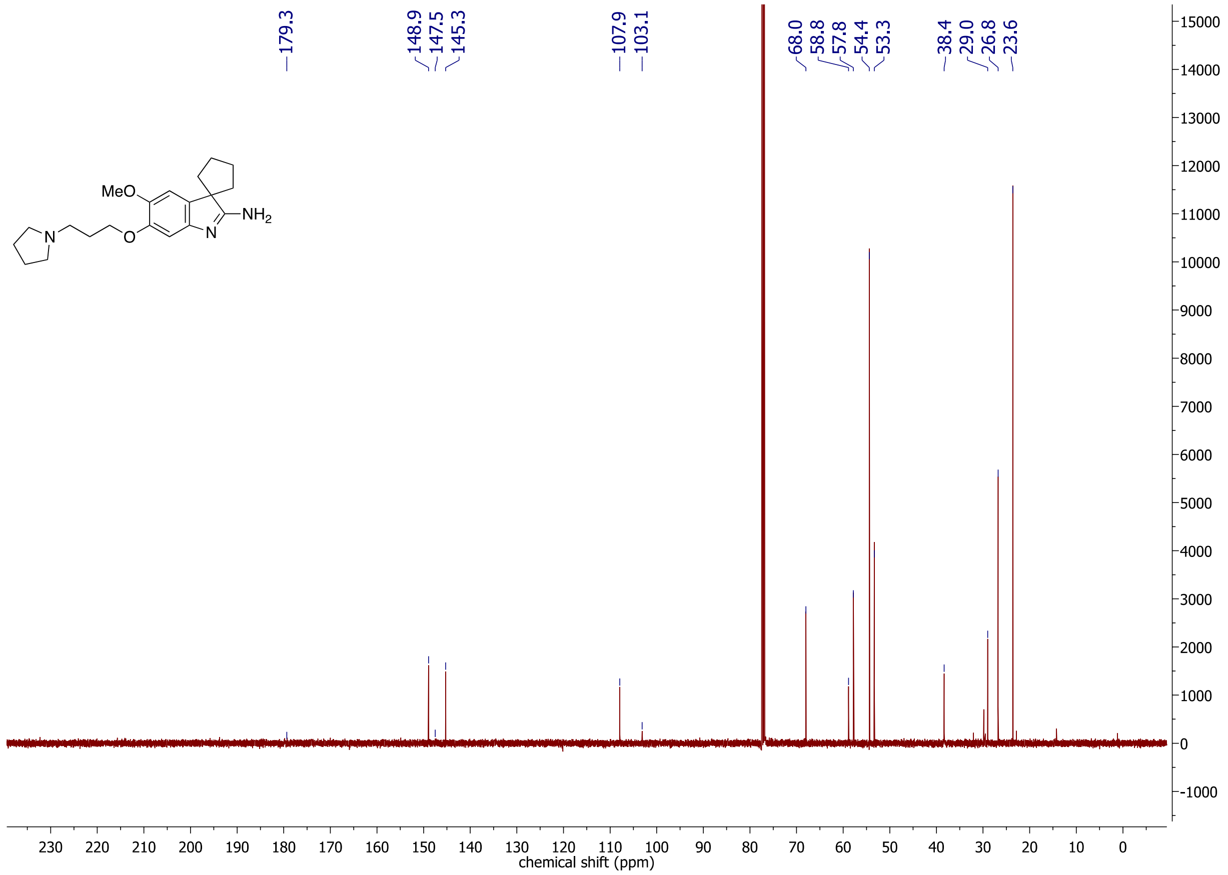

5'-Ethoxy-6'-(3-(pyrrolidin-1-yl)propoxy)spiro[cyclopentane-1,3'-indol]-2'-amine (1g)

^{1}H NMR (400 MHz, $CDCl_3$):

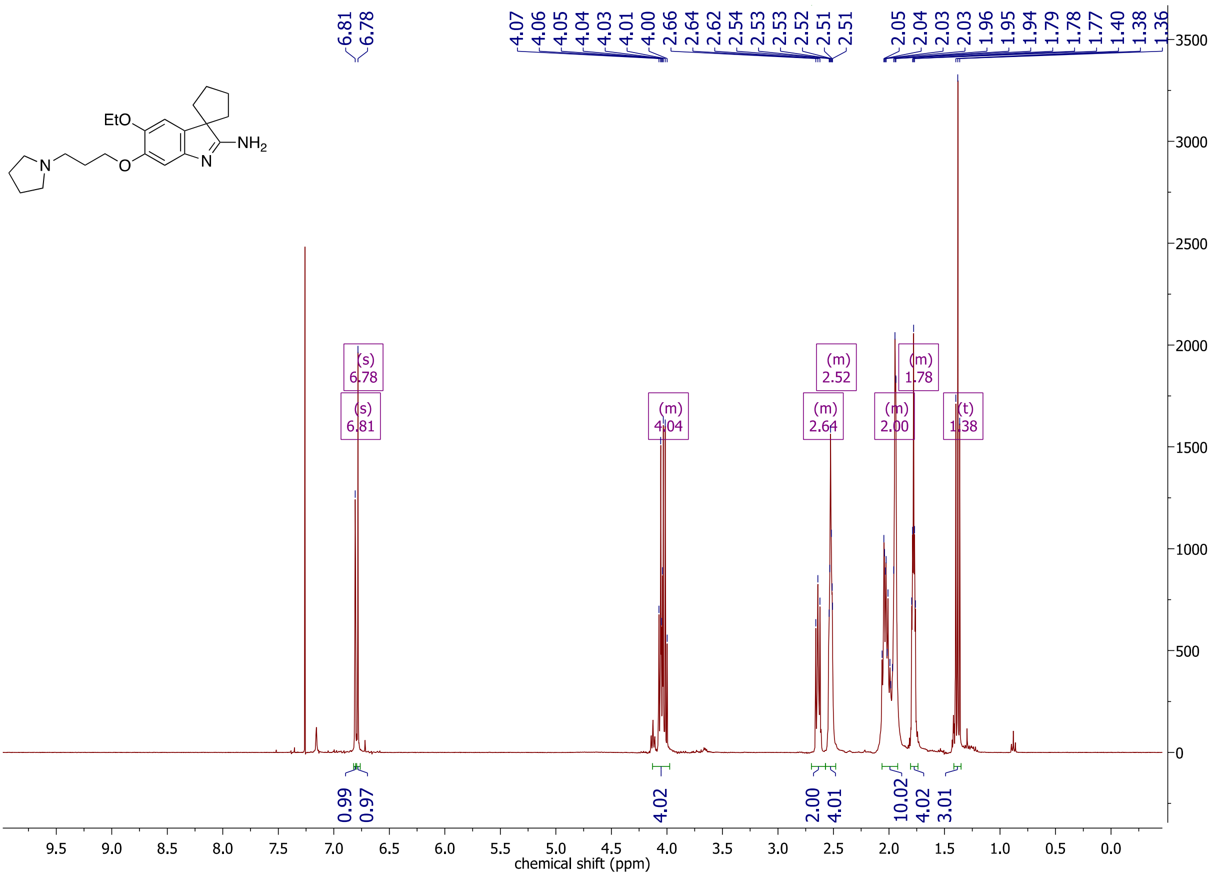

^{13}C NMR (101 MHz, $CDCl_3$):

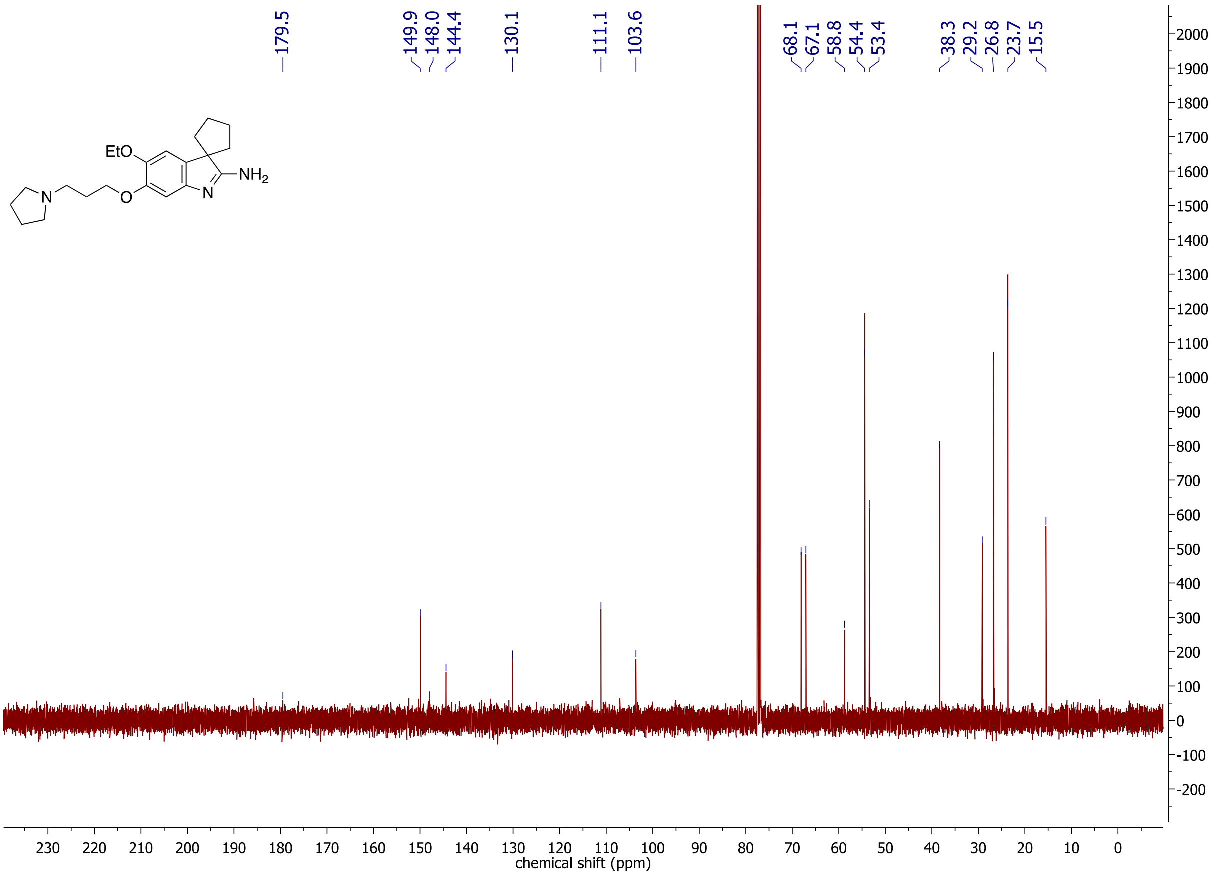

2'-Amino-5'-methoxy-6'-(3-(pyrrolidin-1-yl)propoxy)spiro[cyclopentane-1,3'-indole]-4'-carbonitrile (1h)

^{1}H NMR (400 MHz, $CDCl_3$):

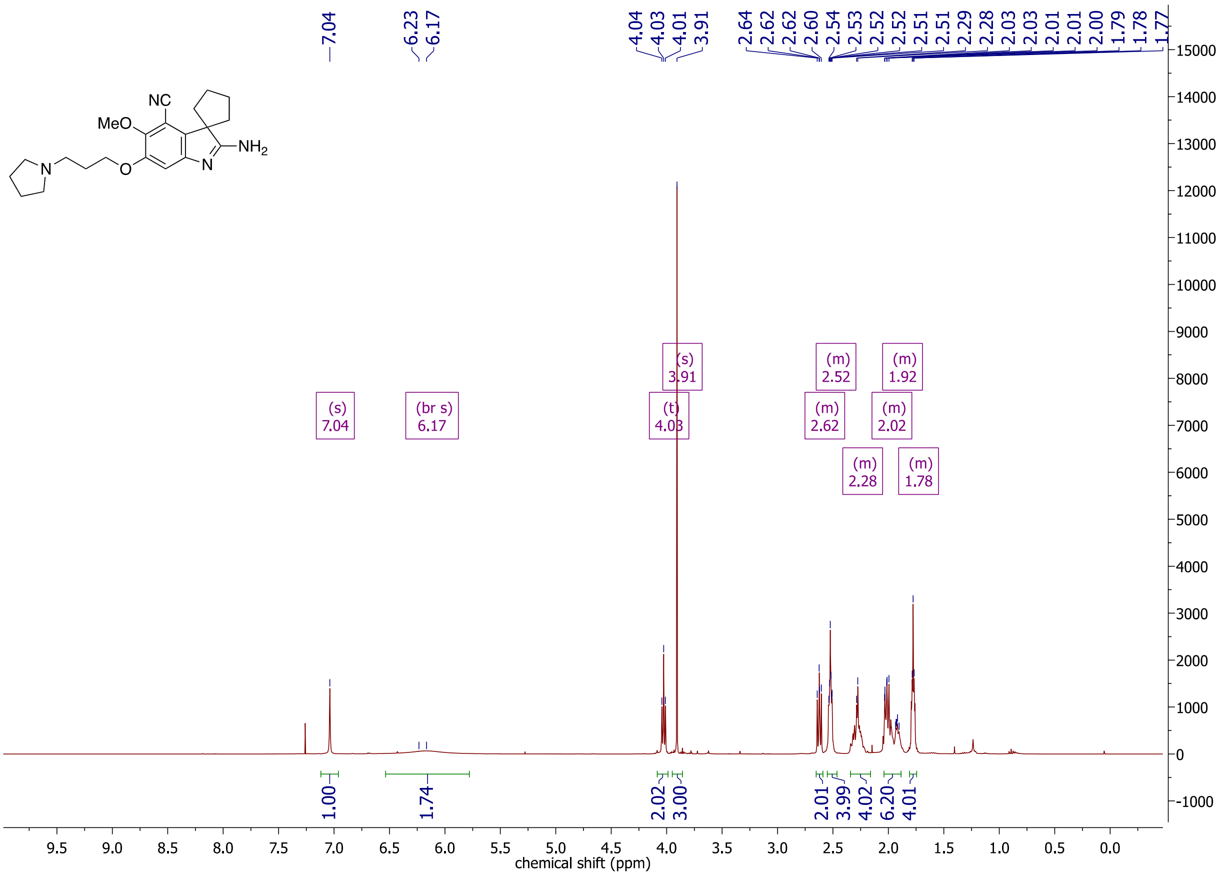

^{13}C NMR (101 MHz, $CDCl_3$):

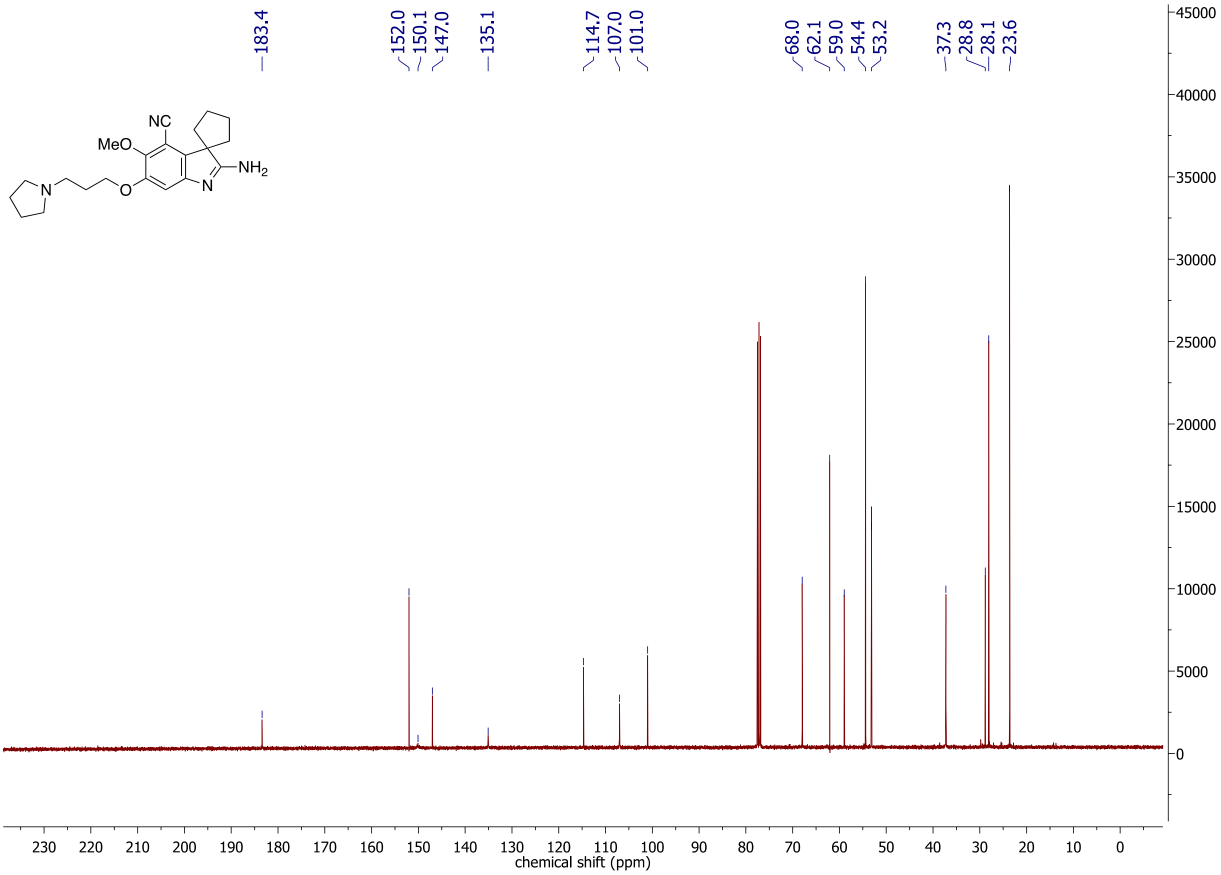

4'-Chloro-5'-methoxy-6'-(3-(pyrrolidin-1-yl)propoxy)spiro[cyclopentane-1,3'-indol]-2'-amine (1i)

^{1}H NMR (400 MHz, $CDCl_3$):

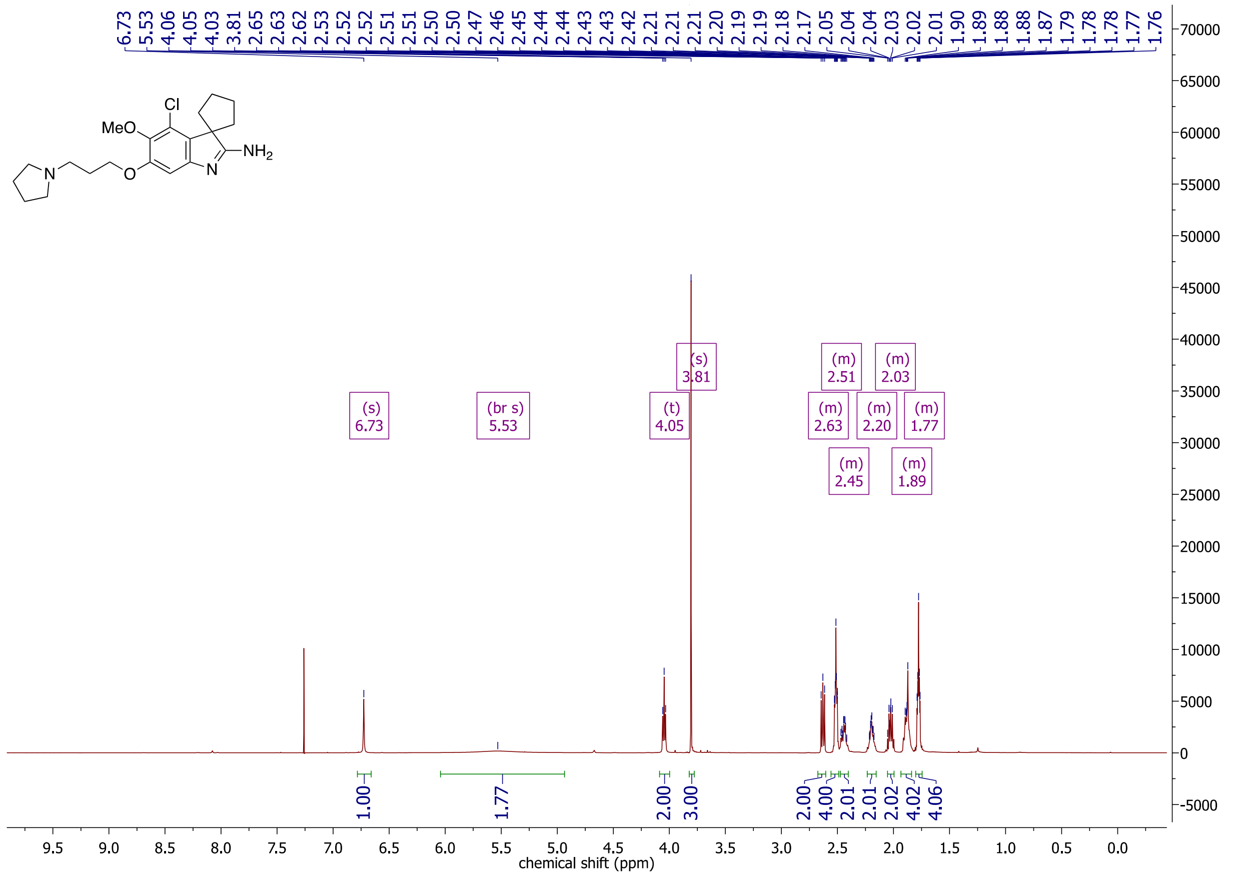

^{13}C NMR (101 MHz, $CDCl_3$):

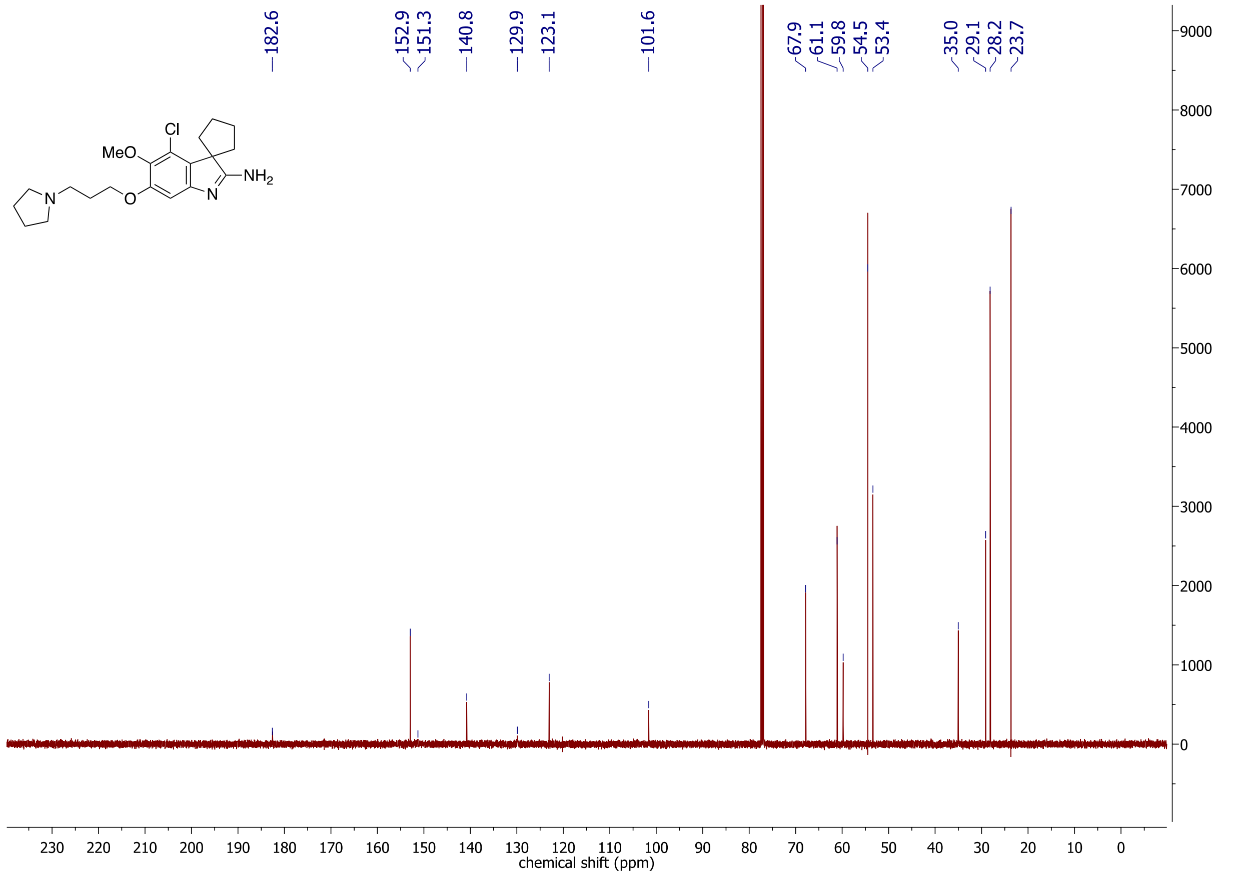

4',5'-Dimethoxy-6'-(3-(pyrrolidin-1-yl)propoxy)spiro[cyclopentane-1,3'-indol]-2'-amine (1j)

^{1}H NMR (400 MHz, $CDCl_3$):

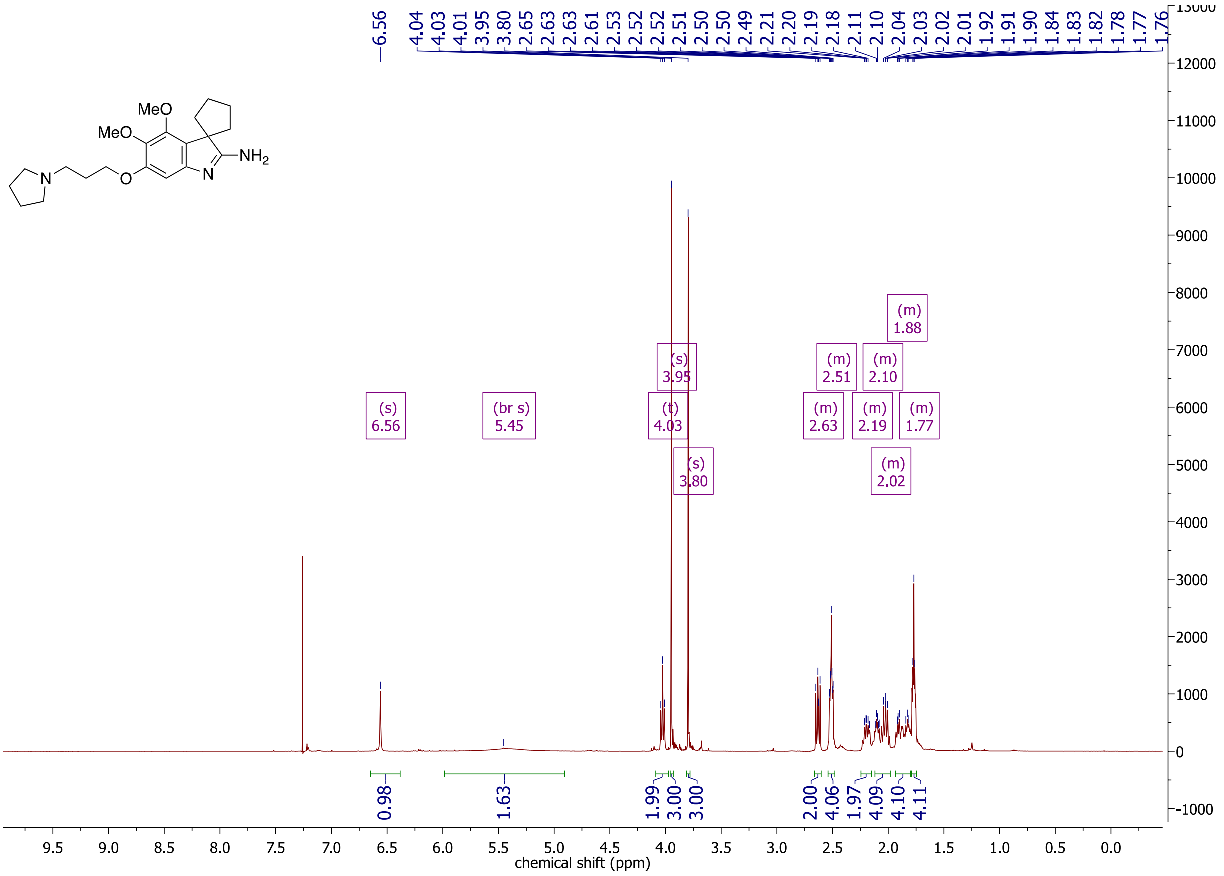

^{13}C NMR (101 MHz, $CDCl_3$):

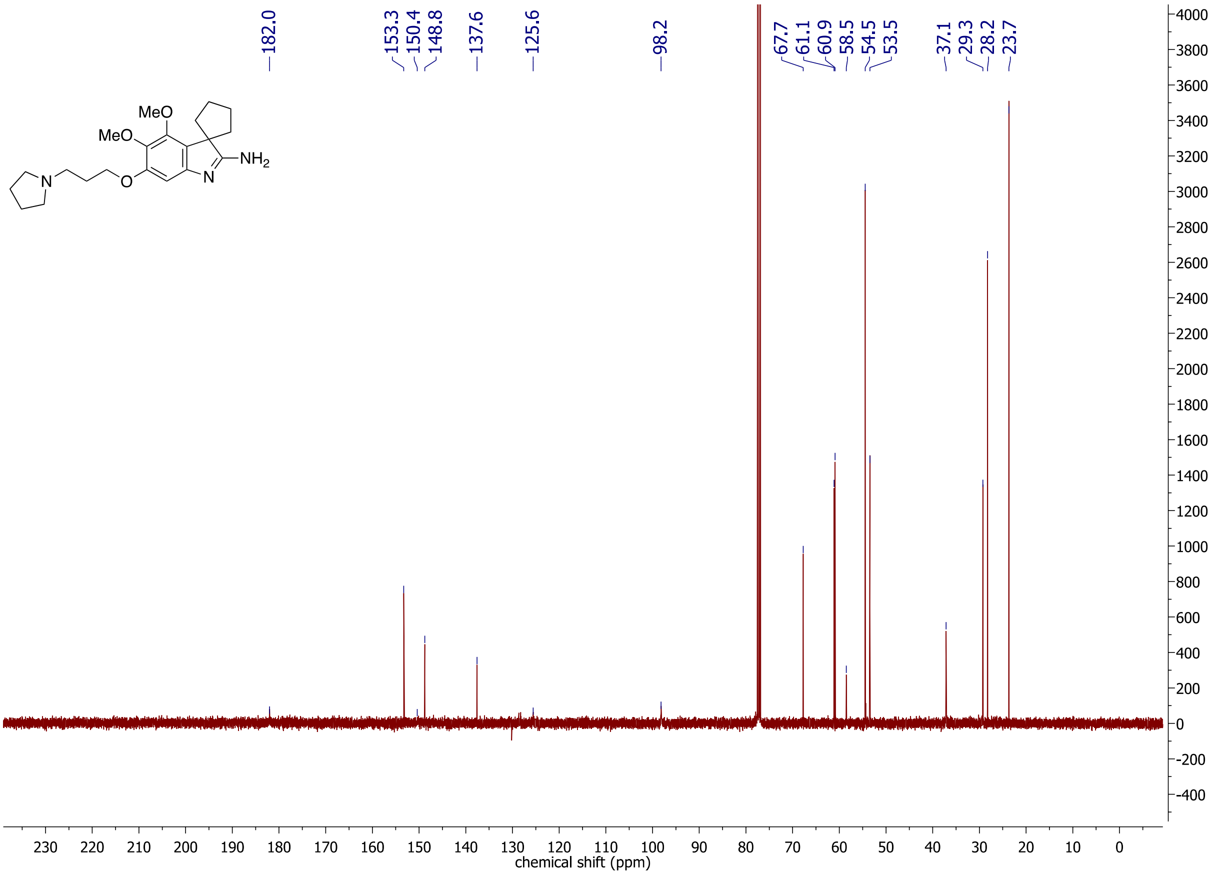

1-(2'-Amino-5'-methoxy-6'-(3-(pyrrolidin-1-yl)propoxy)spiro[cyclopentane-1,3'-indol]-4'-yl)ethan-1-one (1k)

¹H NMR (400 MHz, $CDCl_3$):

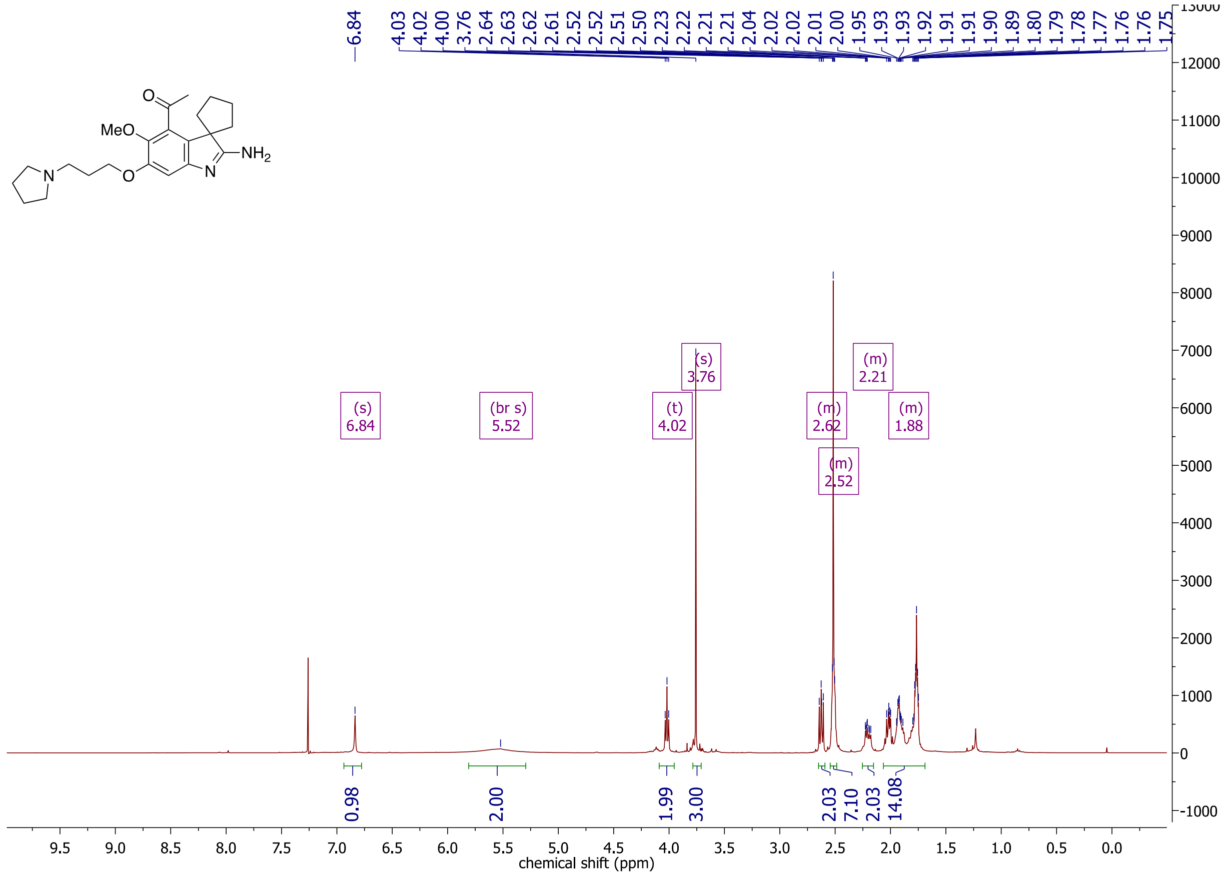

¹³C NMR (101 MHz, $CDCl_3$):

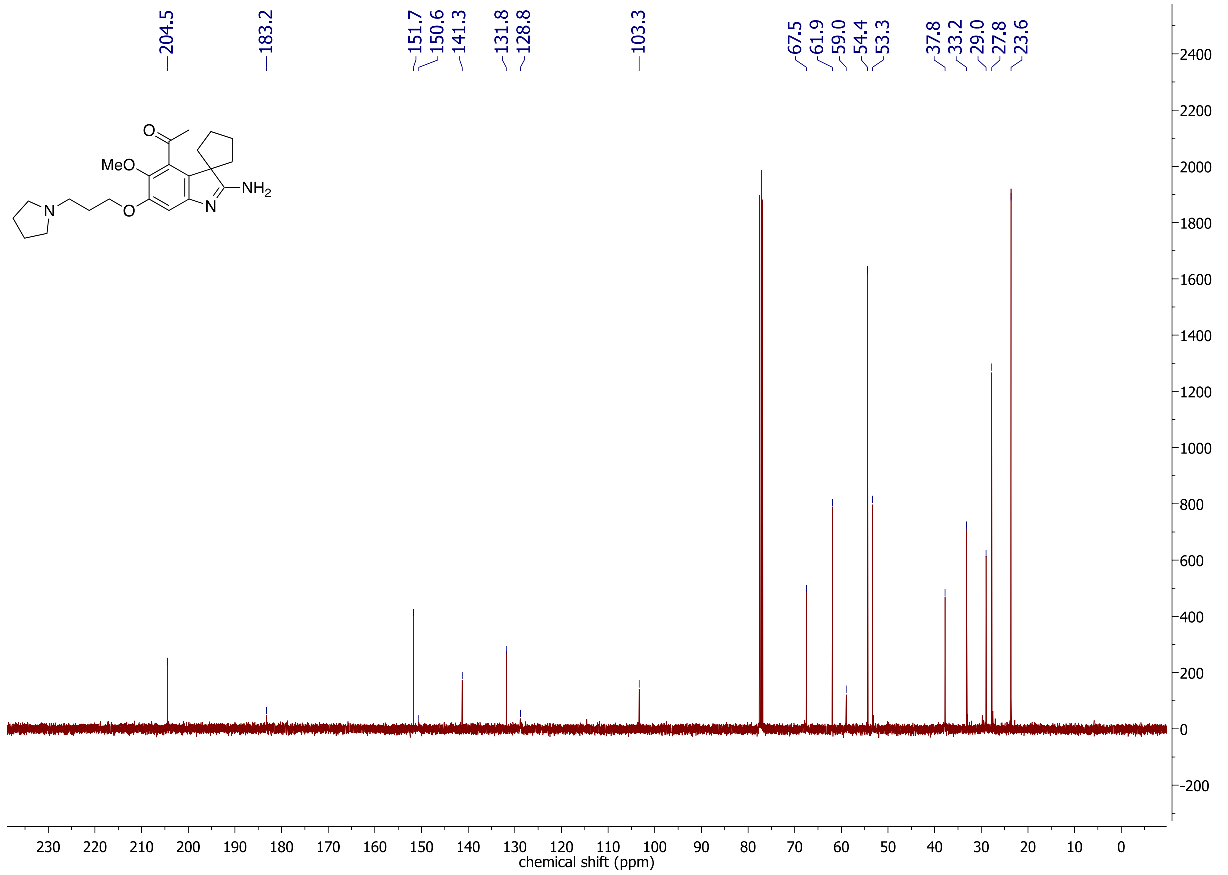

5'-Ethoxy-6'-(3-(pyrrolidin-1-yl)propoxy)spiro[cyclohexane-1,3'-indol]-2'-amine (1l)

^{1}H NMR (400 MHz, $CDCl_3$):

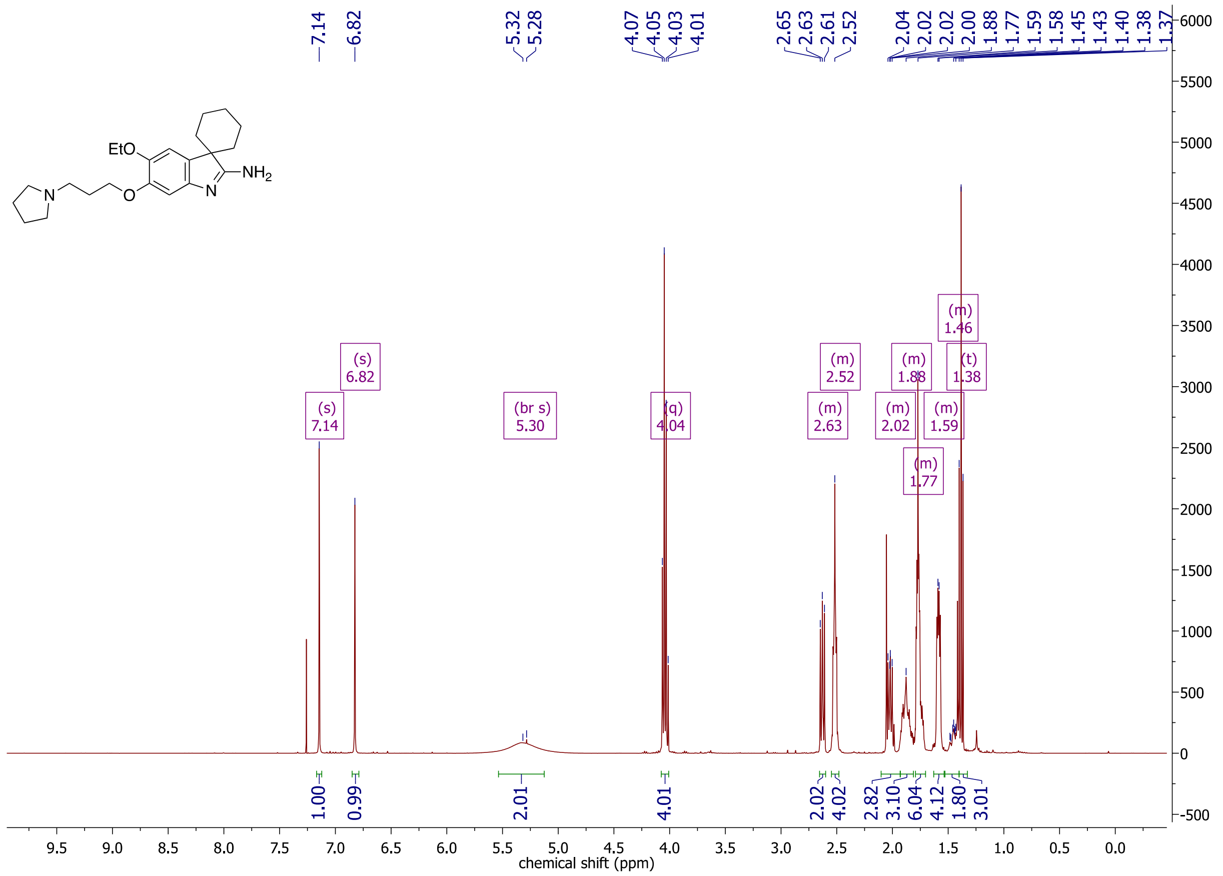

^{13}C NMR (101 MHz, $CDCl_3$):

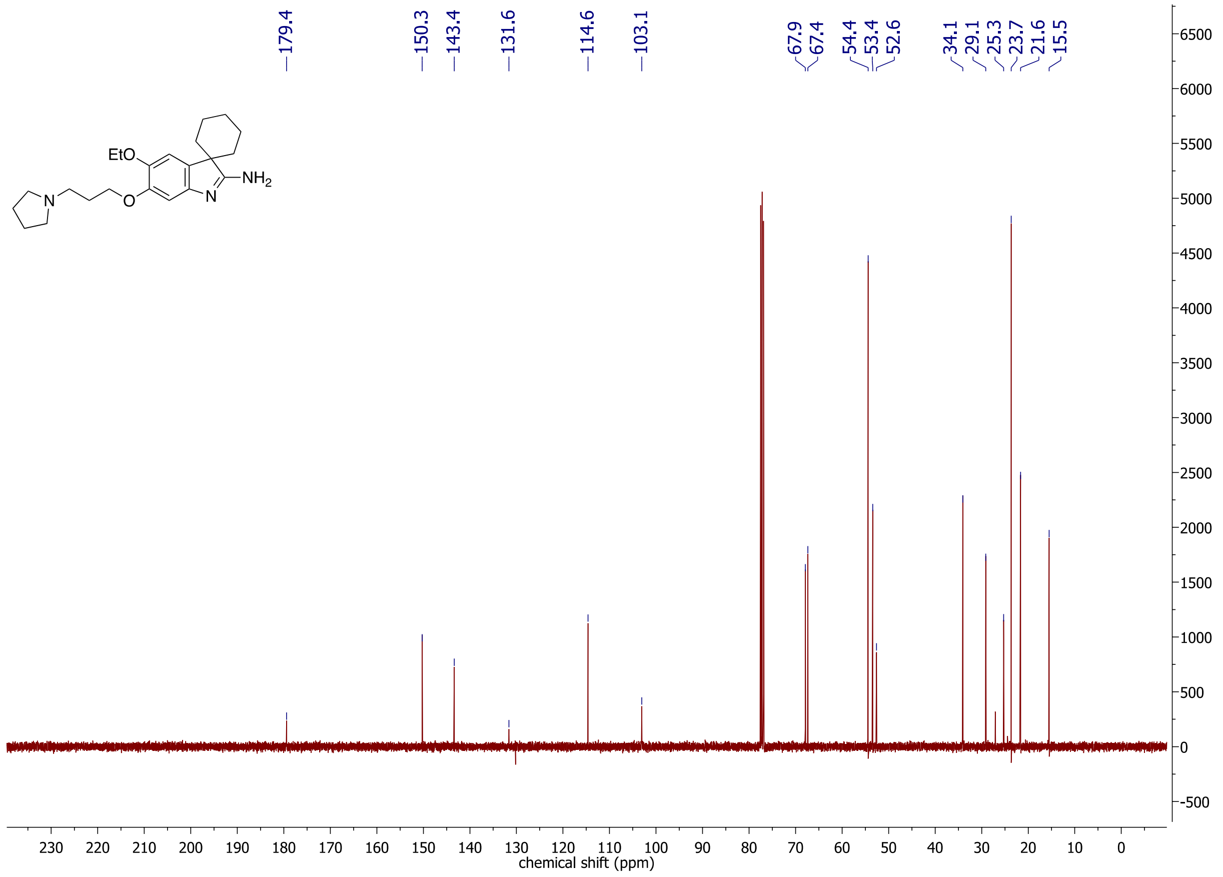

5'-Isopropoxy-6'-(3-(pyrrolidin-1-yl)propoxy)spiro[cyclobutane-1,3'-indol]-2'-amine (1m)

^{1}H NMR (400 MHz, $CDCl_3$):

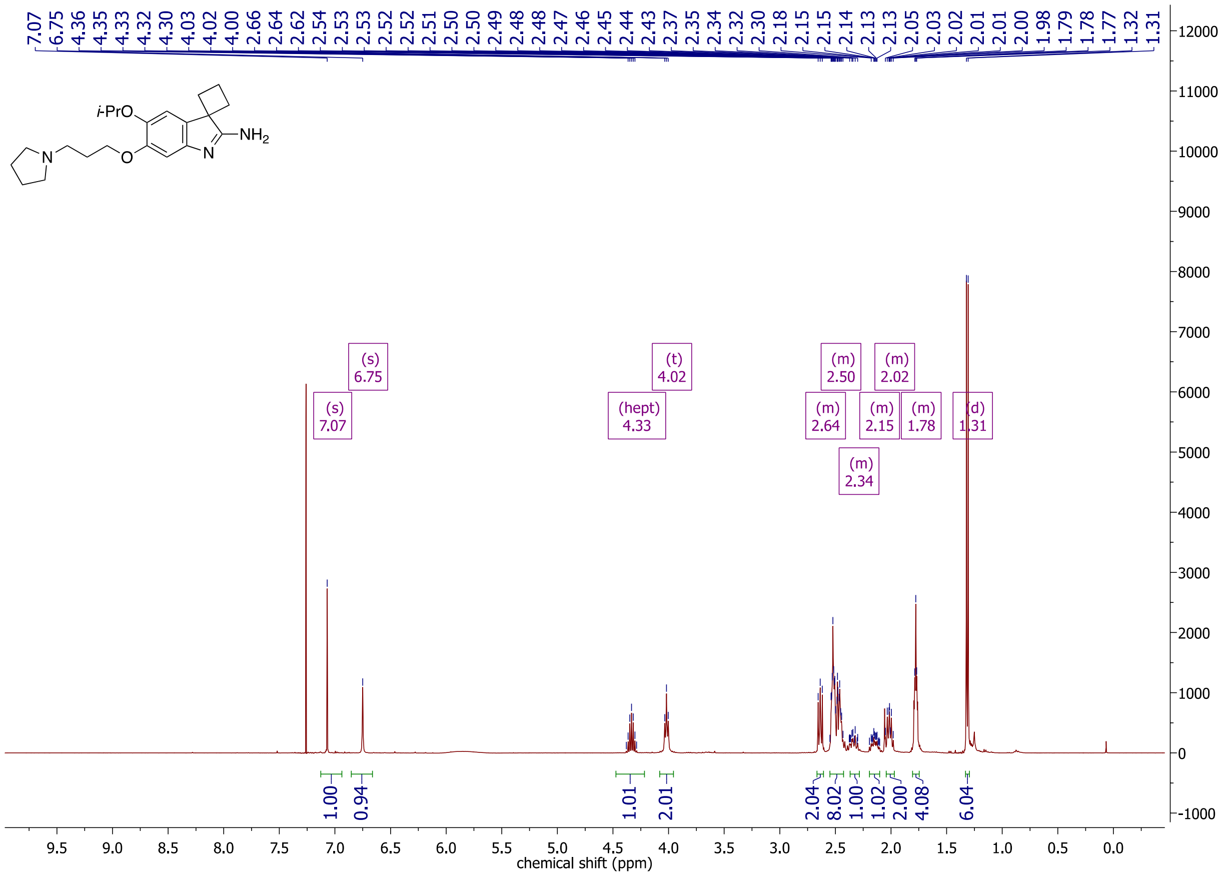

^{13}C NMR (101 MHz, $CDCl_3$):

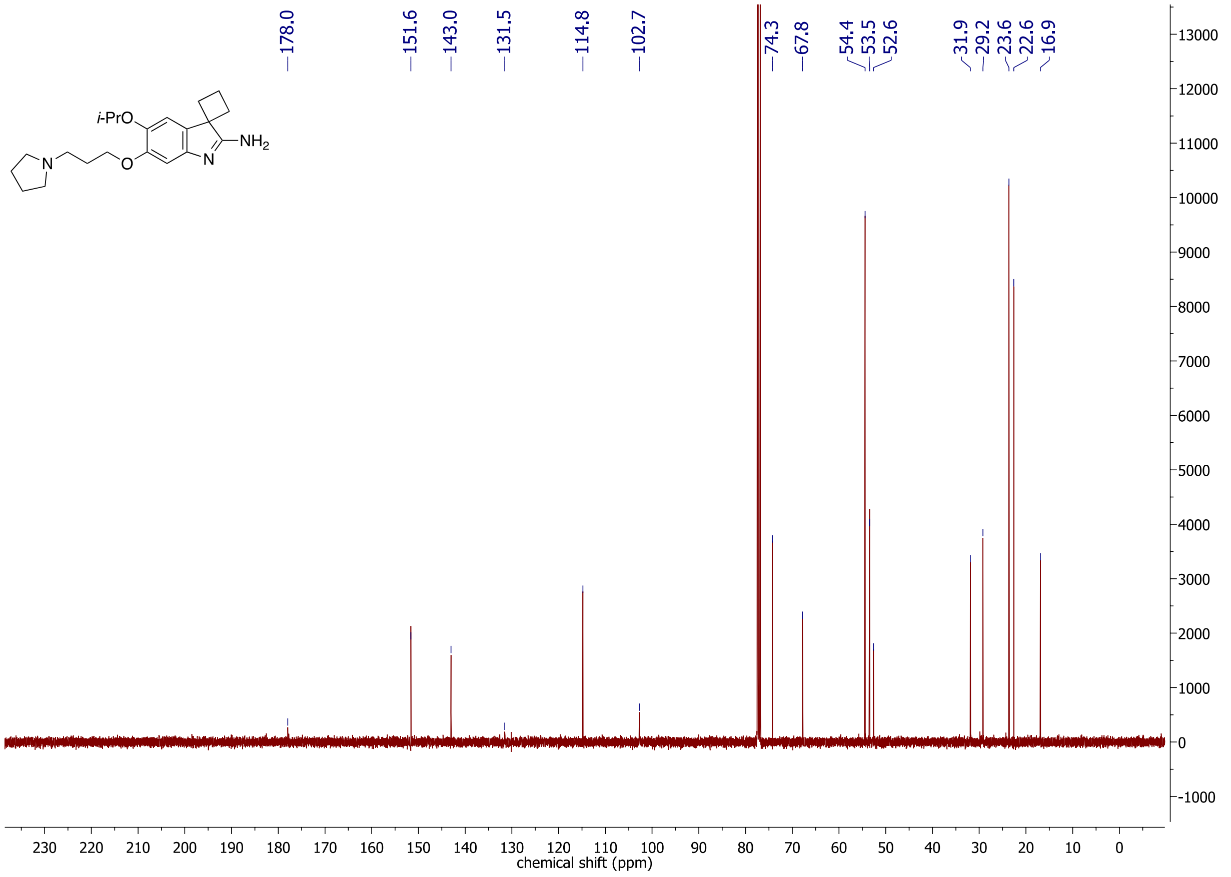

5'-Methoxy-6'-(3-(pyrrolidin-1-yl)propoxy)spiro[cyclohexane-1,3'-indol]-2'-amine (1n)

^{1}H NMR (400 MHz, CDCl3):

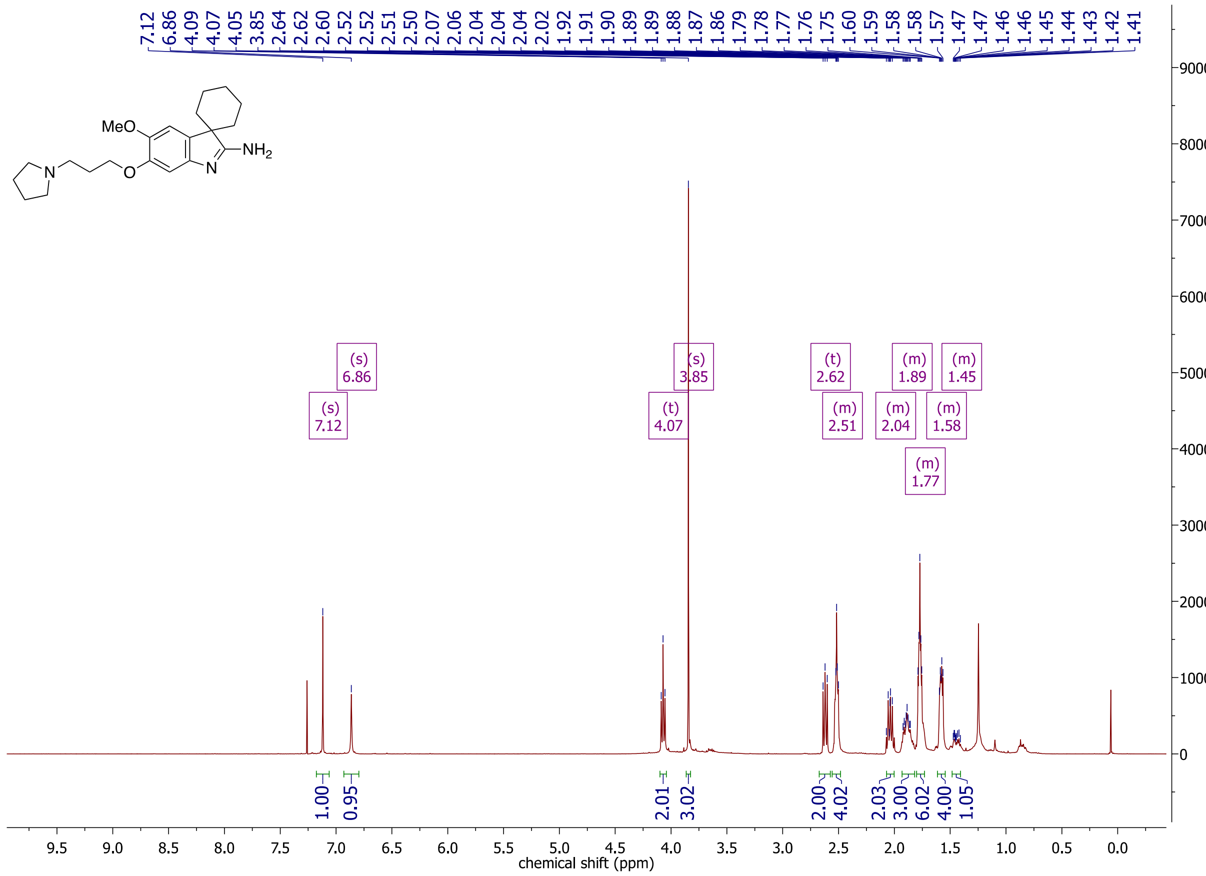

^{13}C NMR (101 MHz, CDCl3):

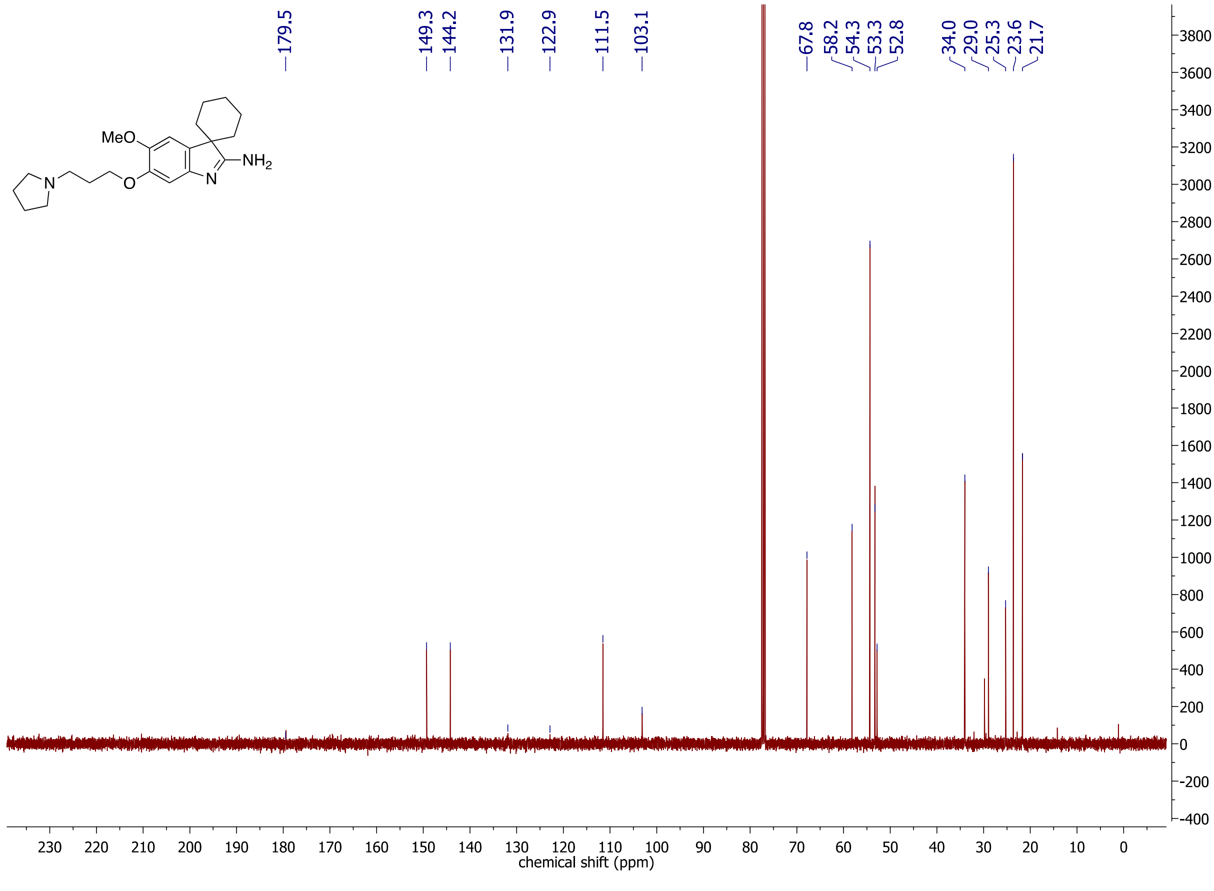

3,3-Diethyl-5-methoxy-6-(3-(pyrrolidin-1-yl)propoxy)-3*H*-indol-2-amine (1o)

^{1}H NMR (500 MHz, $CDCl_3$):

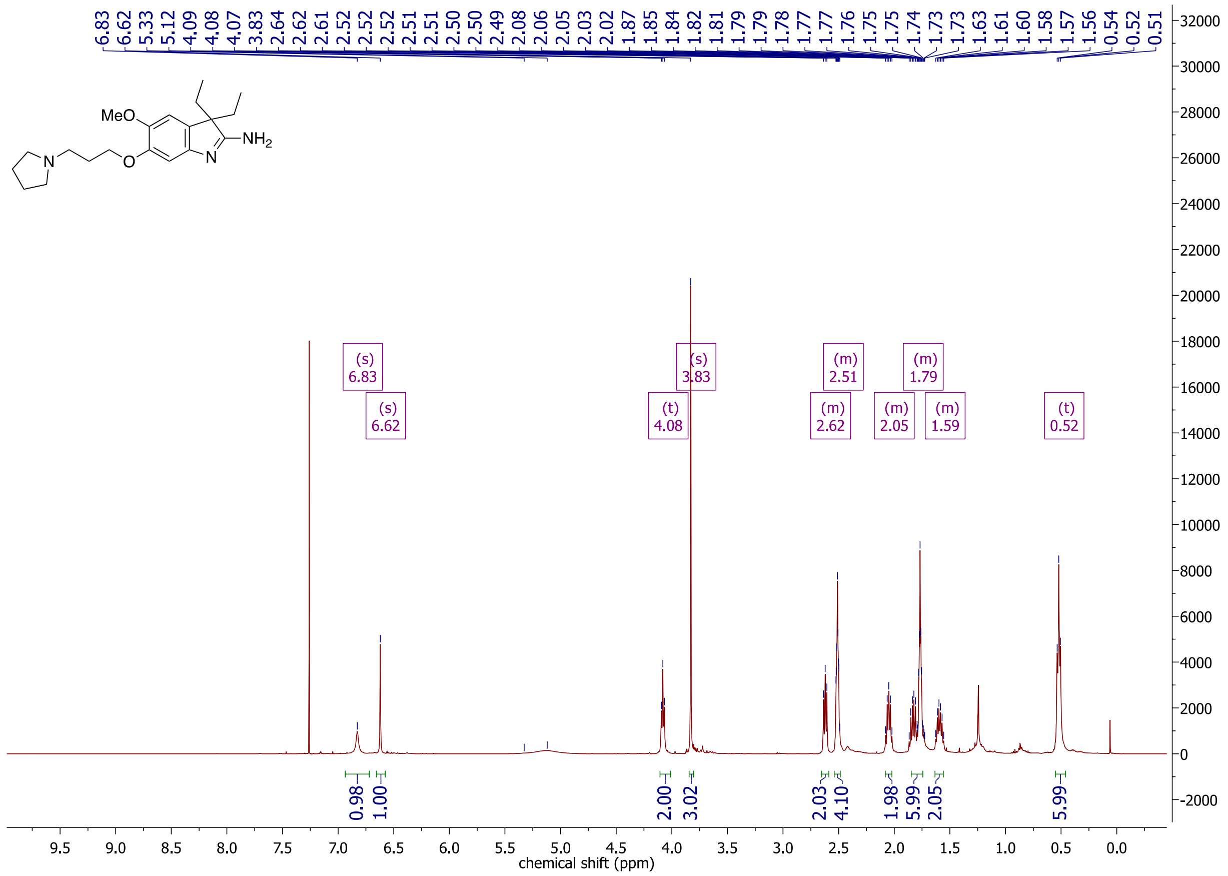

^{13}C NMR (126 MHz, $CDCl_3$):

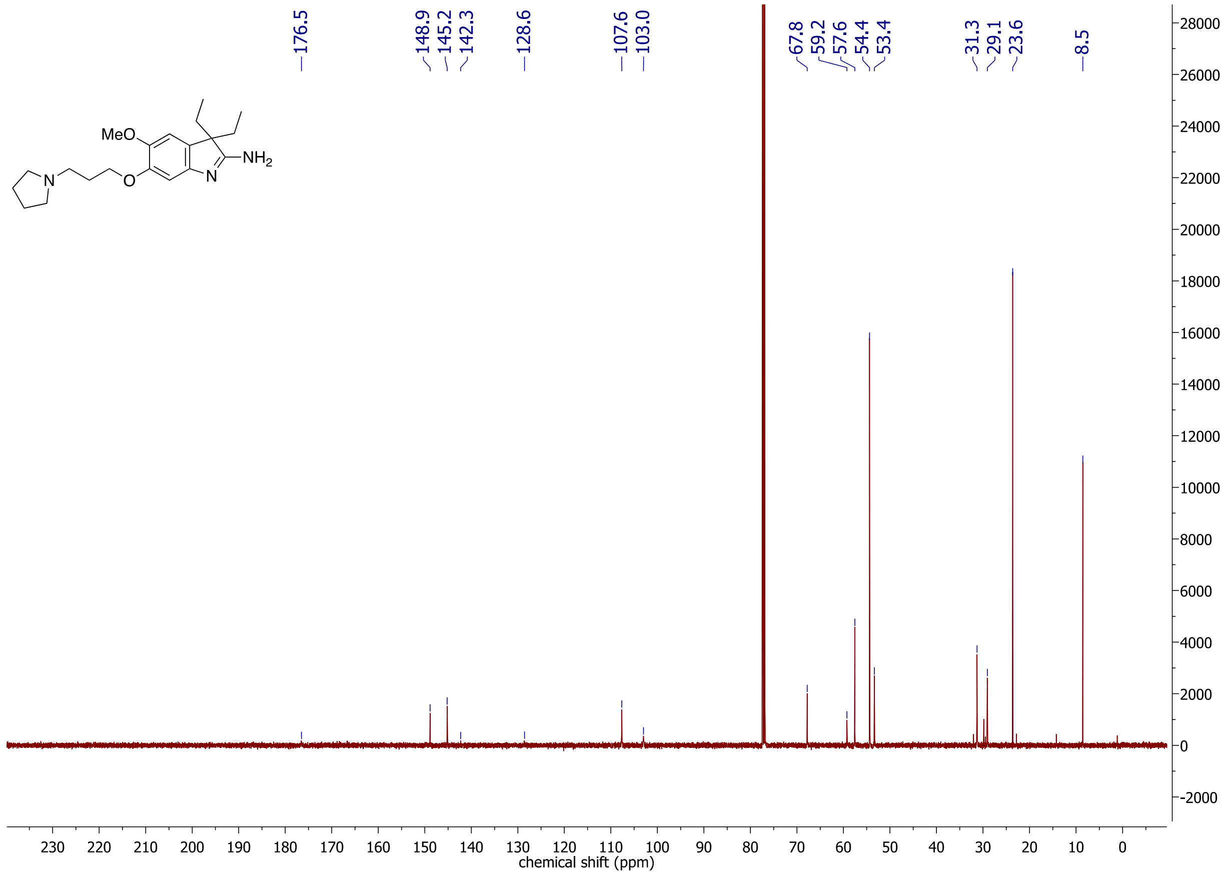

5-Methoxy-3,3-dimethyl-6-(3-(pyrrolidin-1-yl)propoxy)-3*H*-indol-2-amine (1p)

^{1}H NMR (400 MHz, $CDCl_3$):

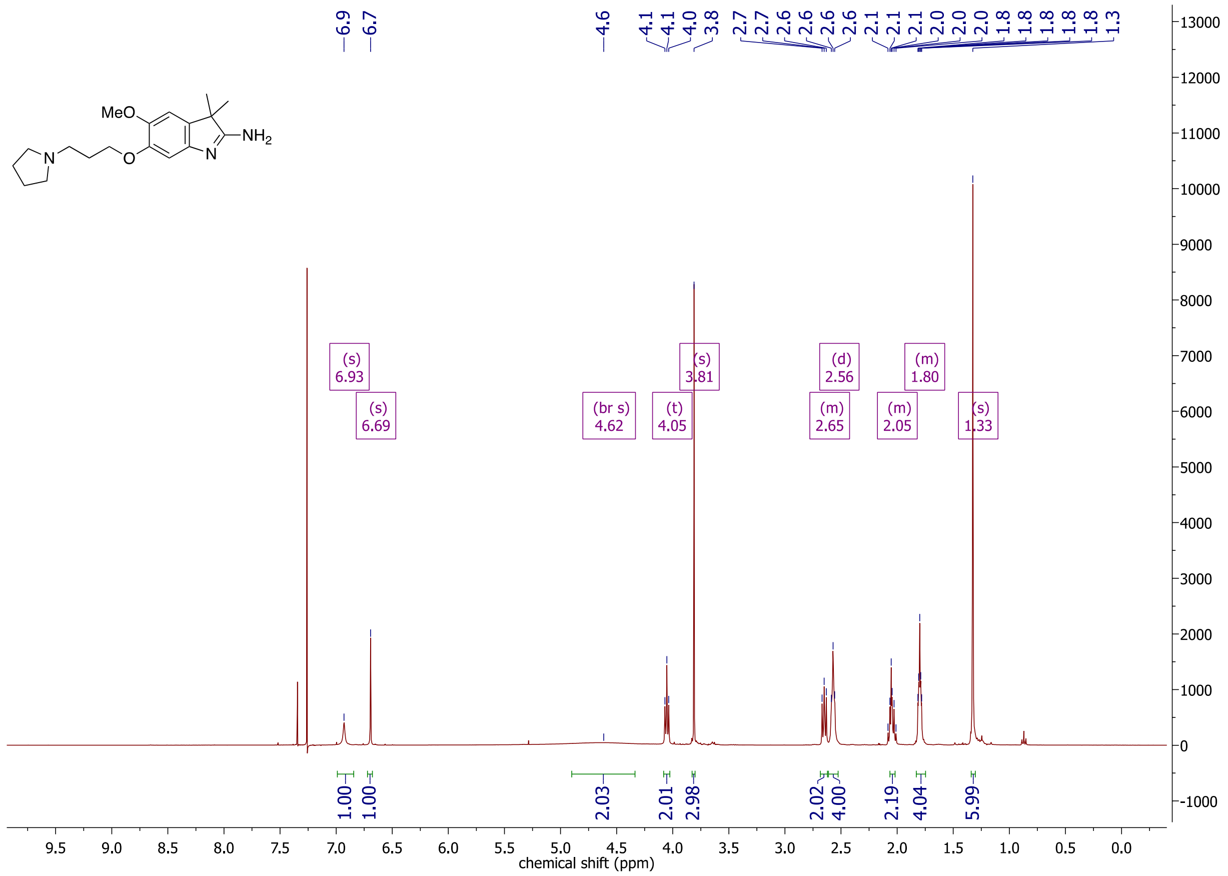

^{13}C NMR (101 MHz, $CDCl_3$):

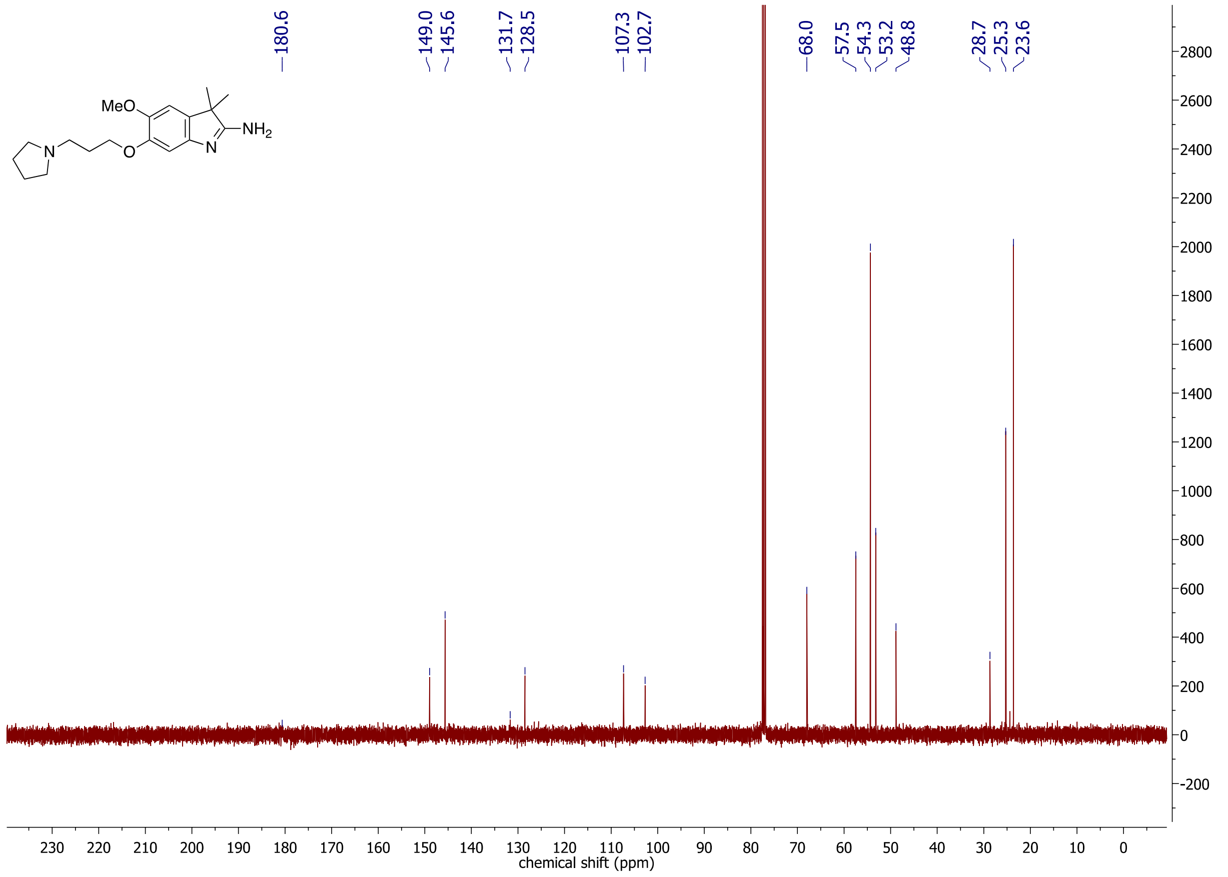

3-Ethyl-5-methoxy-3-methyl-6-(3-(pyrrolidin-1-yl)propoxy)-3*H*-indol-2-amine (1r)

^{1}H NMR (400 MHz, $CDCl_3$):

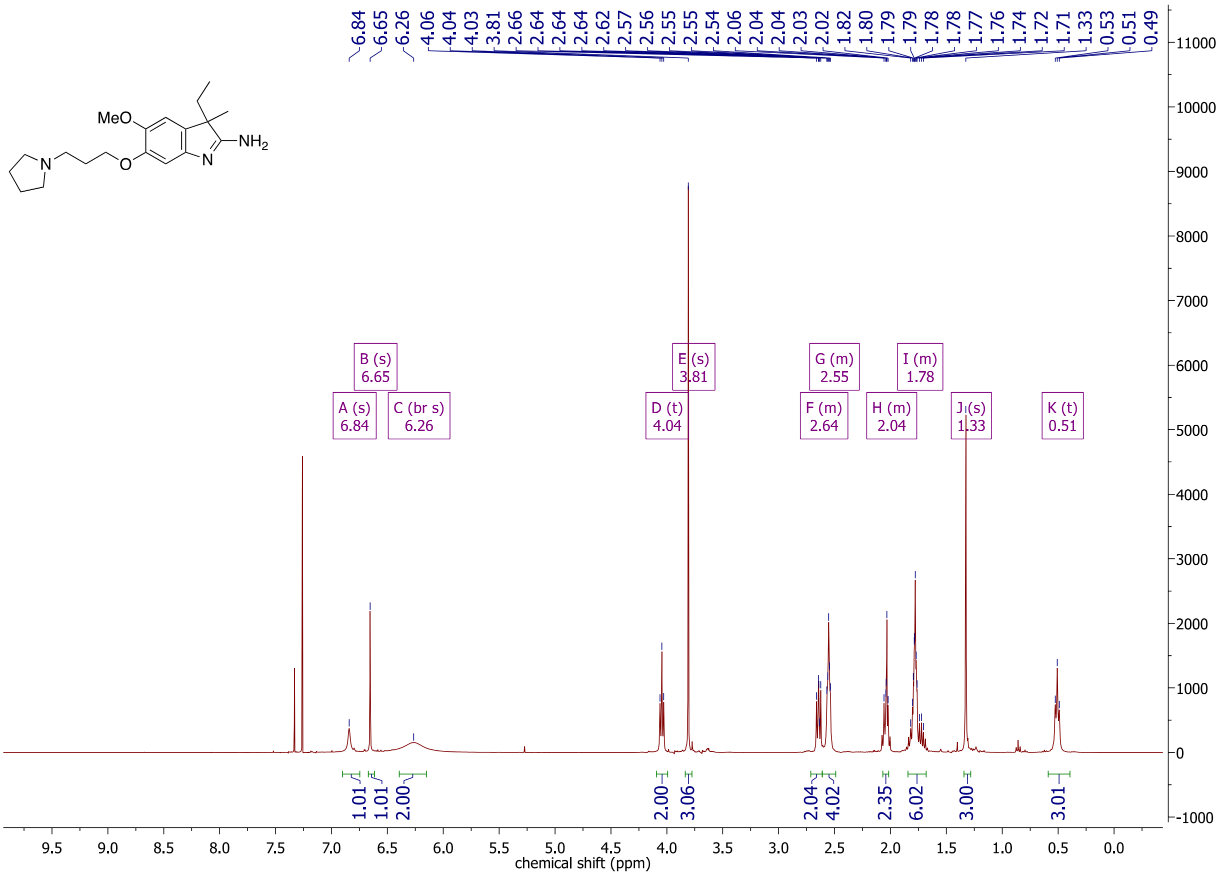

^{13}C NMR (101 MHz, $CDCl_3$):

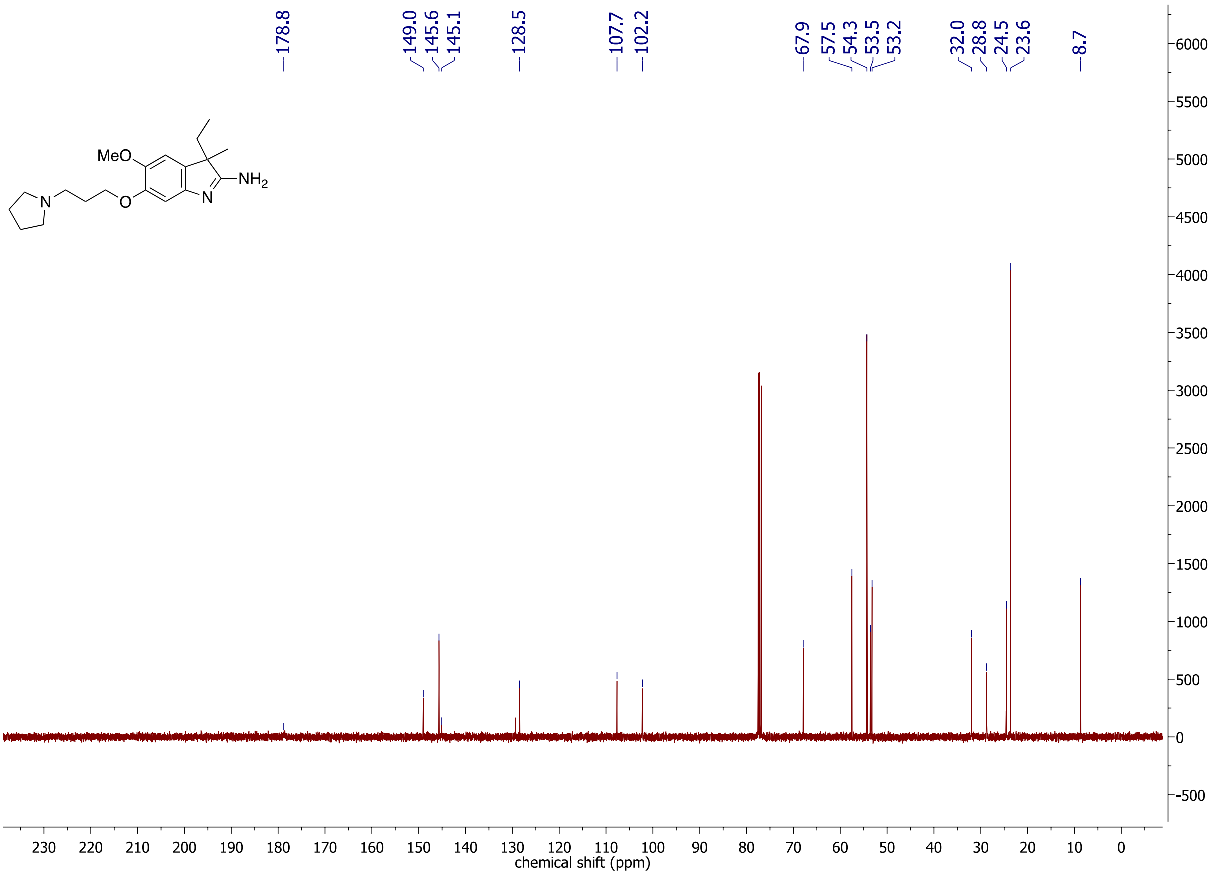

3-Ethyl-6-(3-(isoindolin-2-yl)propoxy)-5-methoxy-3-methyl-3*H*-indol-2-amine (1s)

^{1}H NMR (400 MHz, MeOD-*d*$_4$):

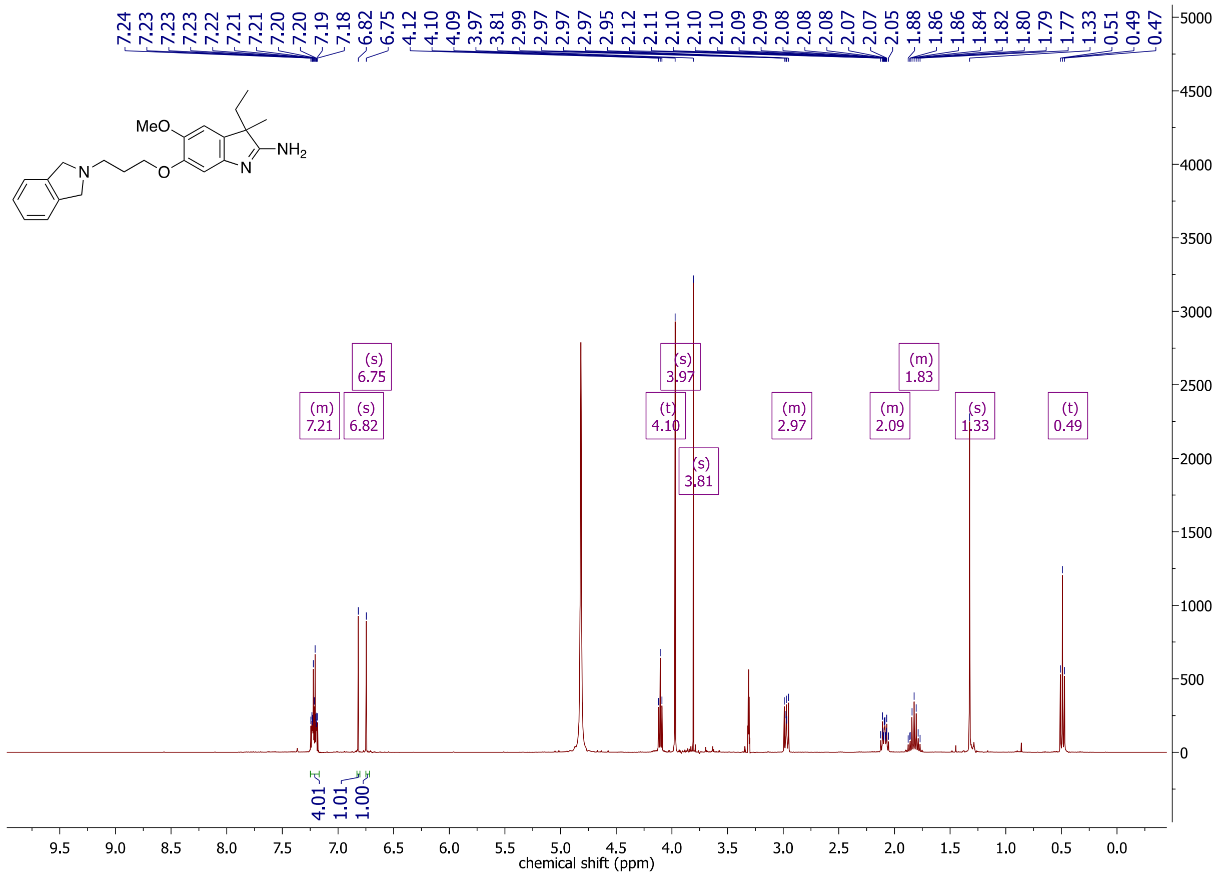

^{13}C NMR (101 MHz, MeOD-*d*$_4$):

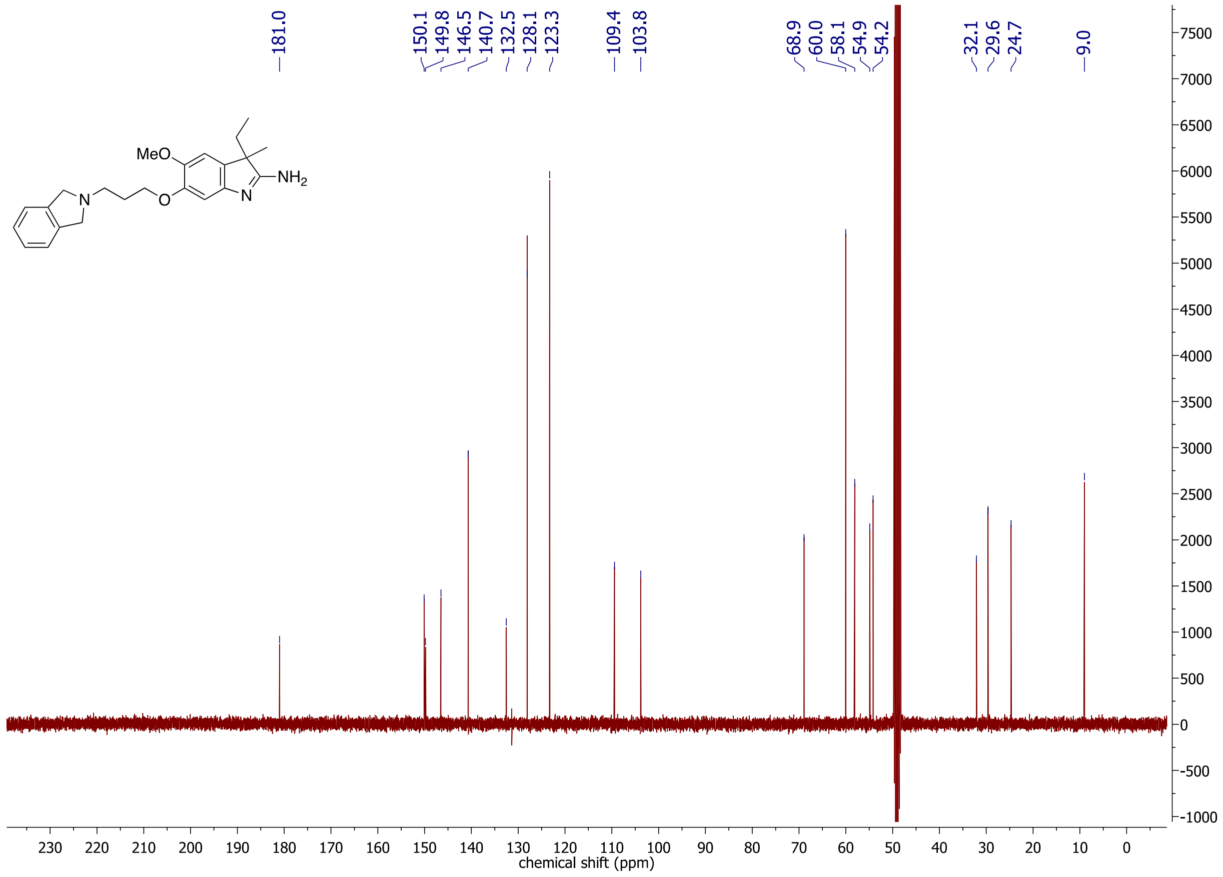

3-Ethyl-5-methoxy-3-methyl-6-(3-((*R*)-3-methylpyrrolidin-1-yl)propoxy)-3H-indol-2-amine (1t)

[1]**H NMR** (400 MHz, MeOD-d_4):

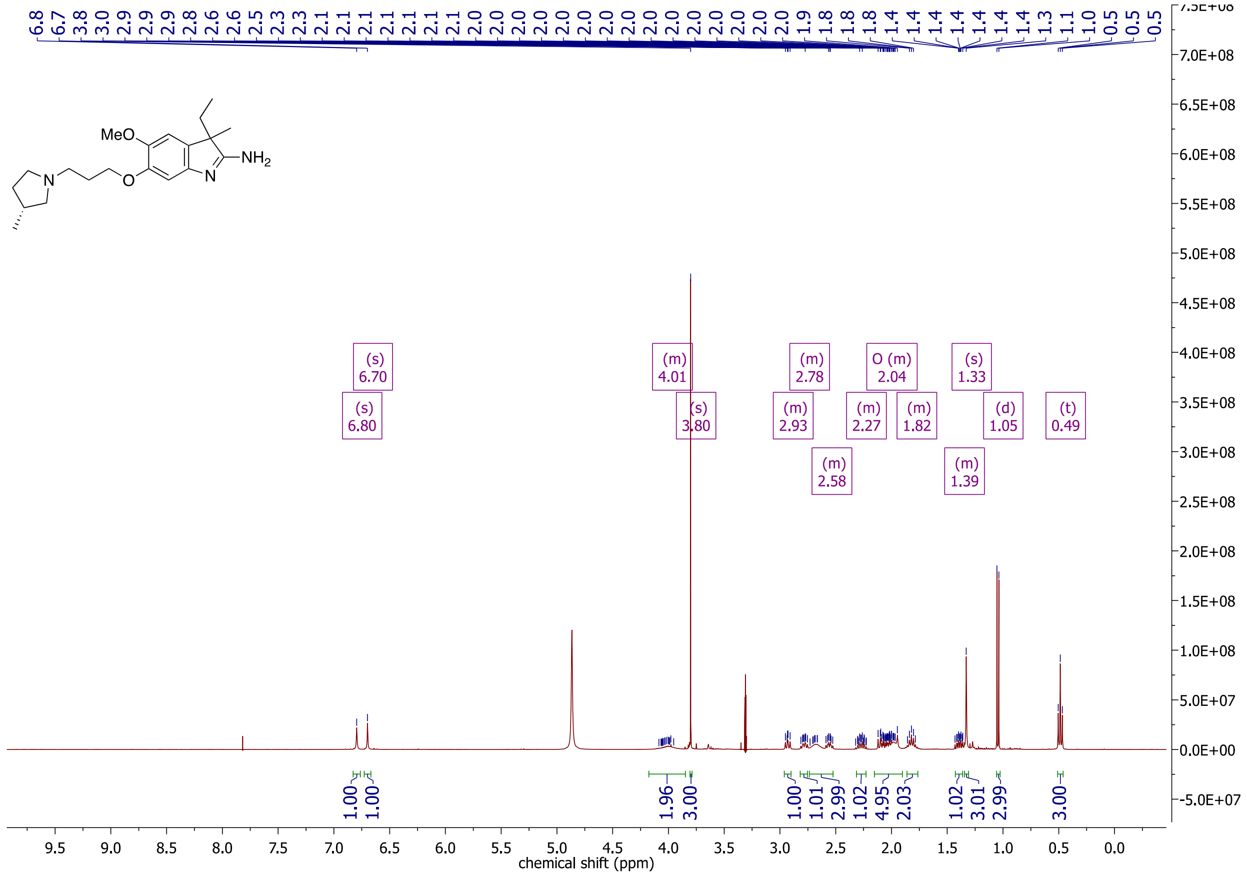

[13]**C NMR** (101 MHz, MeOD-d_4):

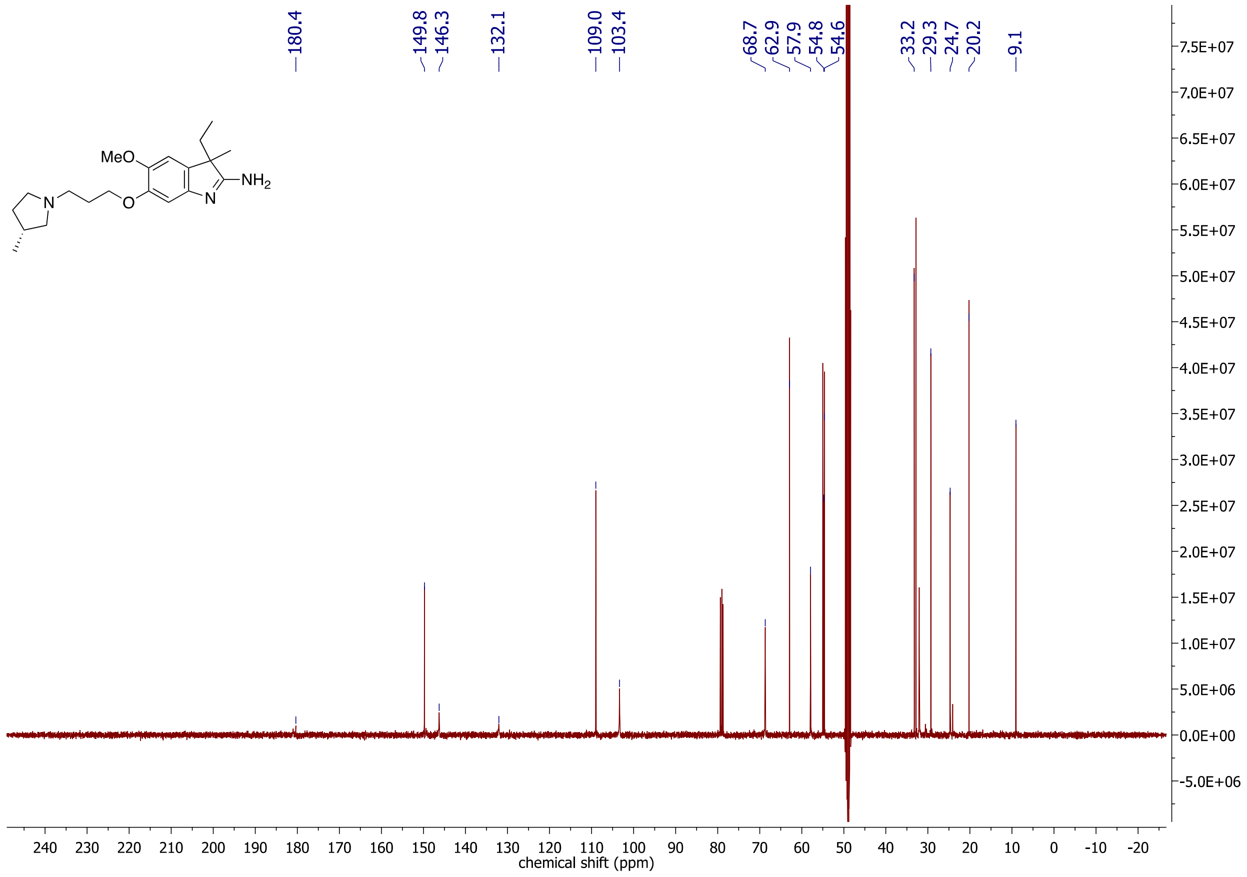

(1-(3-((2'-Amino-5'-methoxyspiro[cyclopentane-1,3'-indol]-6'-yl)oxy)propyl)pyrrolidin-3-yl)methanol (1u)

^{1}H NMR (500 MHz, MeOD-d_4):

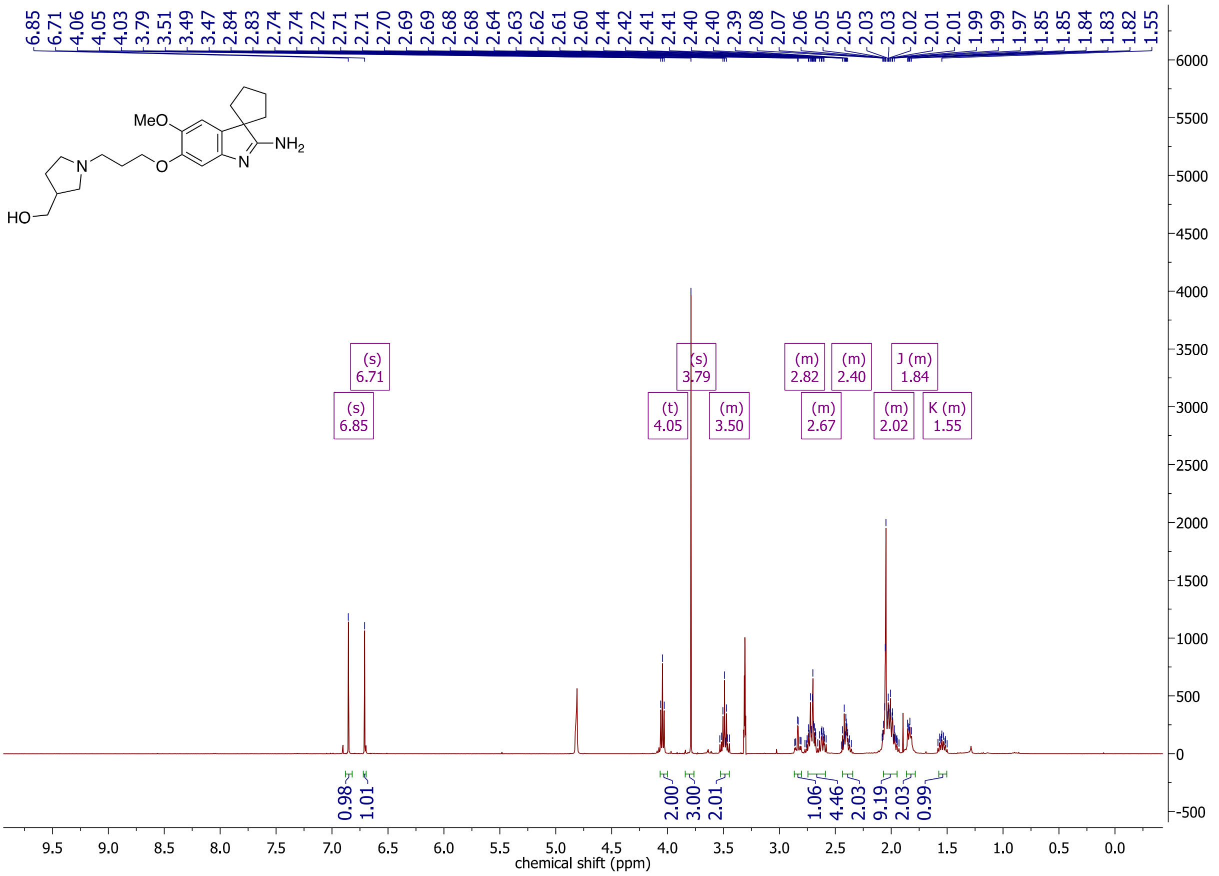

^{13}C NMR (126 MHz, MeOD-d_4):

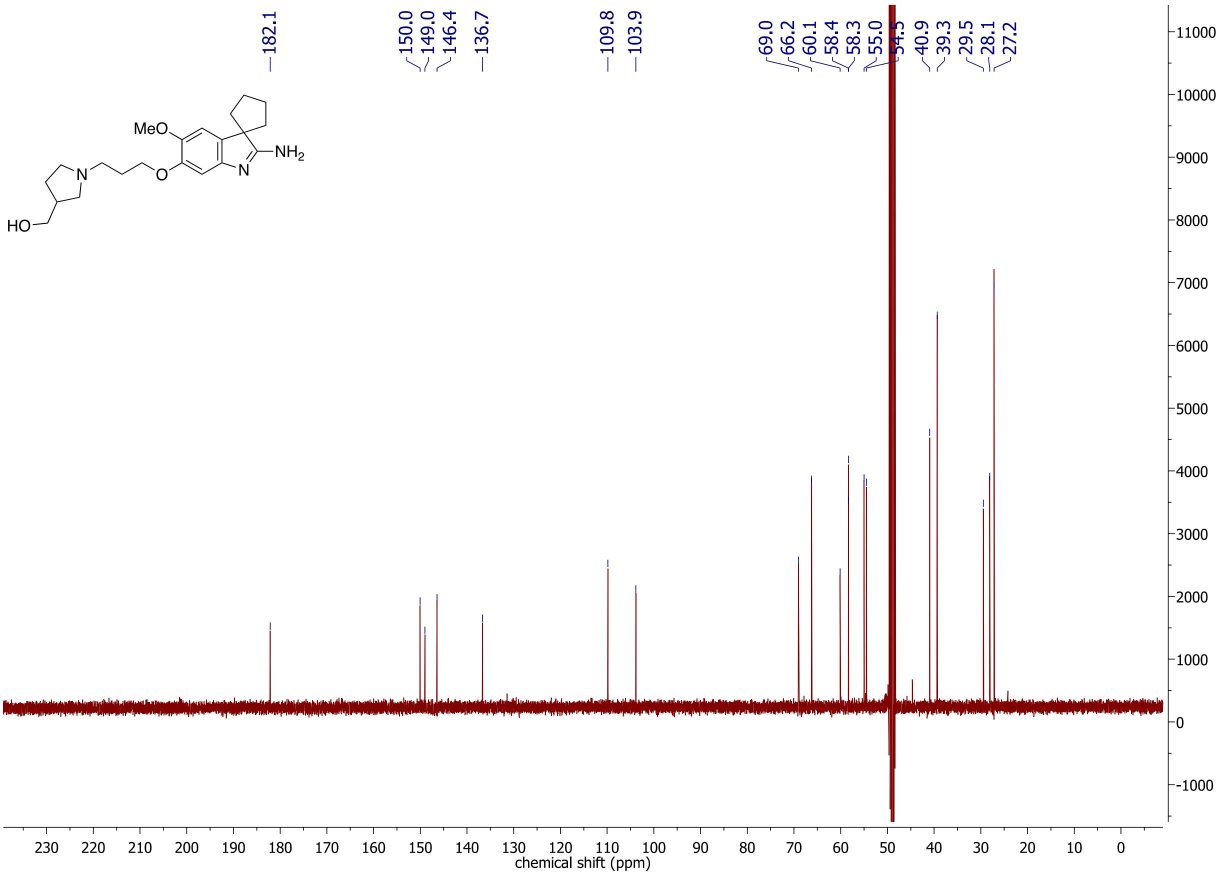

Bibliography

1. Nimgirawath S, Udomputtimekakul P, Taechowisan T, Wanbanjob A, Shen Y. First total syntheses of (+/-)-isopiline, (+/-)-preocoteine, (+/-)-oureguattidine and (+/-)-3-methoxynordomesticine and the biological activities of (+/-)-3-methoxynordomesticine. Chem Pharm Bull (Tokyo). 2009;57(4):368-76.
2. Nakamura M, Kajita D, Matsumoto Y, Hashimoto Y. Design and synthesis of silicon-containing tubulin polymerization inhibitors: replacement of the ethylene moiety of combretastatin A-4 with a silicon linker. Bioorg Med Chem. 2013;21(23):7381-91.
3. Qin H, Xu Z, Cui Y, Jia Y. Total synthesis of (+/-)-decursivine and (+/-)-serotobenine: a Witkop photocyclization/elimination/O-Michael addition cascade approach. Angew Chem Int Ed Engl. 2011;50(19):4447-9.
4. Badarau E, Dilly S, Wouters J, Seutin V, Liegeois JF. Chemical modifications of the N-methyl-laudanosine scaffold point to new directions for SK channels exploration. Bioorg Med Chem Lett. 2014;24(24):5616-20.
5. Imbri D, Tauber J, Opatz T. A high-yielding modular access to the lamellarins: synthesis of lamellarin G trimethyl ether, lamellarin eta and dihydrolamellarin eta. Chemistry. 2013;19(45):15080-3.
6. Ross ST, Franz RG, Wilson JW, Hahn RA, Sarau HM. Synthesis, renal vasodilator and dopamine-sensitive adenylate cyclase activities of O-Methyl derivatives of 6-chloro-2,3,4,5-tetrahydro-1-(4-hydroxyphenyl)-1H-3-benzazepin-7,8-diol (SK&F 82526). Journal of Heterocyclic Chemistry. 1986;23(6):1805-14.
7. Couladouros EA, Soufli IC, Moutsos VI, Chadha RK. Total Synthesis of Combretastatins D. Chemistry – A European Journal. 1998;4(1):33-43.
